# Health of people experiencing co-occurring homelessness, imprisonment, substance use, sex work and/or severe mental illness in high-income countries: a systematic review and meta-analysis

**DOI:** 10.1136/jech-2020-215975

**Published:** 2021-04-23

**Authors:** Emily J. Tweed, Rachel M. Thomson, Dan Lewer, Colin Sumpter, Amir Kirolos, Paul M. Southworth, Amrit Kaur Purba, Robert W. Aldridge, Andrew Hayward, Alistair Story, Stephen W. Hwang, Srinivasa Vittal Katikireddi

**Affiliations:** 1 MRC/CSO Social and Public Health Sciences Unit, University of Glasgow Institute of Health and Wellbeing, Glasgow, UK; 2 Collaborative Centre for Inclusion Health, University College London, London, UK; 3 Department of Public Health, NHS Forth Valley, Stirling, UK; 4 Department of Clinical Infection, Microbiology & Immunology, Institute of Infection, Veterinary & Ecological Sciences, University of Liverpool, Liverpool, UK; 5 The University of Edinburgh Usher Institute of Population Health Sciences and Informatics, Edinburgh, UK; 6 Department of Public Health, NHS Dumfries and Galloway, Dumfries, UK; 7 Institute of Health Informatics, University College London, London, UK; 8 Find and Treat Service, University College London Hospitals NHS Foundation Trust, London, UK; 9 Centre for Urban Health Solutions, St. Michael’s Hospital, Toronto, Ontario, Canada; 10 Department of Medicine, University of Toronto, Toronto, Ontario, Canada

**Keywords:** health inequalities, homelessness, drug misuse, mental health

## Abstract

**Background:**

People affected by homelessness, imprisonment, substance use, sex work or severe mental illness experience substantial excess ill health and premature death. Though these experiences often co-occur, health outcomes associated with their overlap have not previously been reviewed. We synthesised existing evidence on mortality, morbidity, self-rated health and quality of life among people affected by more than one of these experiences.

**Methods:**

In this systematic review and meta-analysis, we searched Medline, Embase, and PsycINFO for peer-reviewed English-language observational studies from high-income countries published between 1 January 1998 and 11 June 2018. Two authors undertook independent screening, with risk of bias assessed using a modified Newcastle-Ottawa Scale. Findings were summarised by narrative synthesis and random-effect meta-analysis.

**Results:**

From 15 976 citations, 2517 studies underwent full-text screening, and 444 were included. The most common exposure combinations were imprisonment/substance use (31% of data points) and severe mental illness/substance use (27%); only 1% reported outcomes associated with more than two exposures. Infections were the most common outcomes studied, with blood-borne viruses accounting for 31% of all data points. Multiple exposures were associated with poorer outcomes in 80% of data points included (sign test for effect direction, p<0.001). Meta-analysis suggested increased all-cause mortality among people with multiple versus fewer exposures (HR: 1.57 and 95% CI: 1.38 to 1.77), though heterogeneity was high.

**Conclusion:**

People affected by multiple exclusionary processes experience profound health inequalities, though there are important gaps in the research landscape. Addressing the health needs of these populations is likely to require co-ordinated action across multiple sectors, such as healthcare, criminal justice, drug treatment, housing and social security.

**PROSPERO registration number:**

CRD42018097189.

## Introduction

Social exclusion can be defined as the processes by which some individuals or social groups are deprived of resources, rights or opportunities to participate in the activities and relationships available to most people in society.^1 2^ Homelessness, imprisonment, substance use, sex work and severe mental illness (SMI) are experiences commonly associated with social exclusion, which often co-occur.^3^ The magnitude of this overlap varies between contexts, but as an example, recent studies from the UK estimate that approximately 1·5 per thousand people experience homelessness, justice involvement and problem substance use in a given year.^4 5^

People affected by any one of these experiences are known to have much higher rates of ill health and premature death than the rest of the population.^3^ For instance, a previous review undertaken by our team found that standardised mortality ratios among people with experience of homelessness, imprisonment, substance use or sex work compared with the general population were between 8 and 12.^3^ That review examined health outcomes associated with these experiences individually and did not investigate their co-occurrence.

There is good reason to hypothesise that multiple forms of exclusion may be associated with poorer health. First, intersectionality approaches have highlighted how overlapping forms of disadvantage can interact to influence an individual’s social experience and therefore their health.^6^ Second, some forms of multiple exclusions appear to be associated with adverse outcomes, whereas for others, the evidence is mixed. For instance, among people released from prison, substance use—but not psychiatric history—is a consistent risk factor for mortality.^7 8^

To our knowledge, the association between multiple forms of exclusion and health outcomes has not previously been reviewed. Understanding this association is increasingly important given rising rates of homelessness, imprisonment and drug-related harms across a number of high-income countries.^9–12^ We aimed to synthesise evidence from high-income countries of the association between lifetime exposure to more than one of the following: homelessness, imprisonment, substance use, commercial sex work or SMI and the following outcomes:

1. All-cause and cause-specific mortality.
2. Morbidity.
3. Self-rated health or quality of life.

We chose to consider SMI as an additional exposure to those included in our previous review, as in some contexts it overlaps substantially with the other experiences of interest^13–15^ and is often associated with both social exclusion^16^ and premature morbidity and mortality.^17^

## Methods

This systematic review and meta-analysis were conducted according to a protocol registered with PROSPERO in advance of study initiation.

### Populations of interest

Exposures of interest in this review were chosen on the grounds that they are among the most extreme and most commonly co-occurring forms of social exclusion in high-income countries: their impacts are likely to be less variable over time and place than experiences, which are more closely allied to individual identity, such as ethnicity, migration or sexual minority status. This choice was also informed by continuity with a previous review undertaken by our team^3^ and a forthcoming cohort study drawing on administrative data sources.

Study participants comprised people with a lifetime history of more than one of the following:

- Homelessness (including people who are rough sleeping or unstably/marginally housed).
- Imprisonment.
- Substance use (other than alcohol, cannabis or image/performance-enhancing drugs).
- Sex work (including transactional sexual relationships, ie, sex in exchange for food, accommodation and drugs).
- SMI, defined as schizophrenia spectrum disorders, other psychotic disorders and/or bipolar disorder or according to the primary study’s definition of “serious” or “severe” mental illness.

Alcohol was not included in the exposure definition for substance use, as the legality and ubiquity of alcohol in many high-income countries mean that its use is less stigmatised and less closely associated with social exclusion.^18^ Cannabis was similarly excluded given the legalisation or decriminalisation of its use in many high-income countries.^19^ Where a study referred to substance use without distinguishing between these categories, studies were included.

Studies were excluded if participants were recruited from secondary healthcare settings or on the basis of specific health conditions or healthcare utilisation (other than for SMI or substance use). The comparator group was defined as people with fewer or none of the exposures of interest. Given the number of studies retrieved, it was decided at the full-text stage to exclude data points without any comparison group.

### Outcomes

The outcomes of interest were mortality (all-cause or cause-specific, categorised according to International Classification of Diseases 10th revision (ICD-10) chapters); morbidity (based on clinical diagnosis, hospitalisation, validated diagnostic tool or self-report, categorised according to ICD-10); and quality of life, health-related quality of life and self-rated health (based on formal measures such as SF-36 (36 Item Short-Form Survey) or EQ-5D). We excluded outcomes relating to the perinatal period, health behaviours, engagement with preventative health services and prognosis or treatment success. Outcomes that clearly preceded exposures of interest (eg, disorders of early childhood) were not eligible. Both absolute and relative outcome measures were eligible for inclusion.

### Study design

Eligible study designs were cross-sectional, case–control and cohort studies and baseline data from interventional studies. Systematic reviews were eligible for inclusion if they had a clearly specified review question, reported a search strategy including more than one database and used explicit inclusion criteria to select studies.

### Publication characteristics

Given that the relationship between the exposures and outcomes of interest is at least partly dependent on context (eg, social policy, healthcare provision, public attitudes and other factors), we restricted our search to studies published in the last two decades (from 01 January 1998 to 6 November 2018) and in the English language from high-income countries (World Bank classification),^20^ to maximise the relevance of the evidence retrieved to current policy and practice in those countries. Conference papers, theses, correspondence and editorials or other commentary were excluded.

### Searches, screening and data extraction

Medline, Embase and PsycINFO were searched on 6 November 2018 using a search strategy developed with an information specialist and detailed in online supplemental appendix 1. Screening was undertaken in Covidence, following automatic and manual deduplication. All title/abstracts were screened for inclusion by ET and independently by a second reviewer. A 20% sample of the resulting full texts underwent double screening; the remainder was single-screened by ET. The kappa statistic for the double-screened sample was 0.93.

10.1136/jech-2020-215975.supp2 Supplementary data

Data extraction of studies eligible for inclusion was undertaken by ET and checked independently by a second reviewer using a standardised form, available in online supplemental appendix 1. We contacted authors where eligible data points were identified but not published in an extractable format (eg, graphical presentation only). Risk of bias (RoB) was assessed independently by ET and a second reviewer at the outcome level, using an adapted version of the Newcastle-Ottawa Scale for non-randomised studies (online supplemental appendix 1). Screening conflicts and discrepancies in data extraction or RoB assessment were resolved by discussion and, where necessary, the adjudication of a third reviewer (SVK).

For completeness, where systematic reviews were identified, we reviewed these to identify any original studies not included in the original searches, which appeared to report relevant outcomes for >more than one exposure group in combination.^21^ These underwent title and abstract screening followed by full-text review as per studies retrieved by searches: of 104 potentially eligible studies identified in this way, only seven met inclusion criteria and were included in the final analysis.

Studies were reviewed to identify duplicate data where results from a single research study were presented in separate publications: where this occurred, the study with the largest or most representative sample size was included. Potentially overlapping data points (eg, reporting both absolute and relative measures for the same population and outcome or reporting the same outcome for non-mutually exclusive substance use subgroups) were deduplicated using the criteria outlined in online supplemental appendix 1.

### Data synthesis

Narrative synthesis of findings was undertaken according to a prespecified protocol, summarising the characteristics of included studies: the range, direction, and size of associations reported; the results of RoB assessment; and key gaps in the literature.^22^ Effect direction plots were created as a visual aid to synthesis and sign tests used to test the null hypothesis of equivalent outcomes between multiple excluded groups and comparators.^23^ All visualisations were created in R V.3.6.3 using the packages *ggplot2* and *rworldmap*.

Meta-analyses were undertaken to compare multiple with fewer exposures in order to explore the overarching review question of whether multiple exclusions is associated with poorer health. We anticipated substantial heterogeneity and therefore planned in advance to use random-effect models, using the *metan* command in Stata V.15. These were carried out separately by effect measure as most studies reported only point estimates without absolute numbers, precluding synthesis across multiple effect measures. Funnel plots were used to visually assess the potential of publication bias for key outcomes reported in the manuscript.

## Results

**Figure 1:**
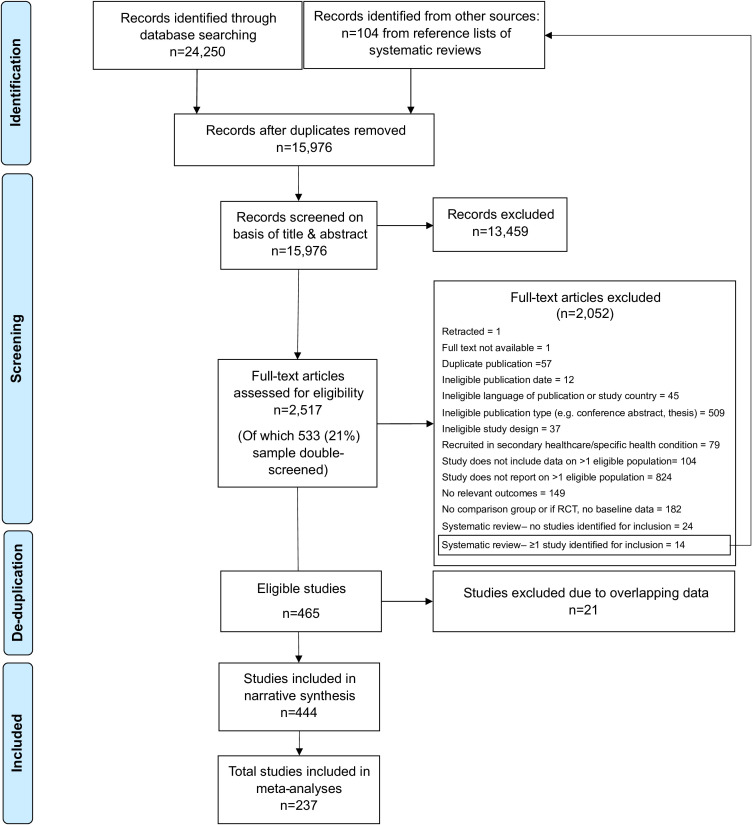
PRISMA flow chart.

Figure 1 shows the study selection figure. After deduplication, a total of 444 studies were included, yielding 1480 data points (ie, effect estimates for a unique population and comparator combination) in total. Details of included studies are in online supplemental appendix 2.

Thirty countries were represented (online supplemental figure A3.1 in appendix 3), though the majority of studies were carried out in the USA (n=164; 37%), Canada (n=57; 13%), the UK (n=41; 9%) or Australia (n=39; 9%). Most studies were cross-sectional (n=327; 74%); only 23% studies (n=103) reported longitudinal data. With regard to RoB, 63% of data points (n=932/1480) were assessed as having low RoB, though this varied by study design (online supplemental table A3.1 in appendix 3).

**Figure 2:**
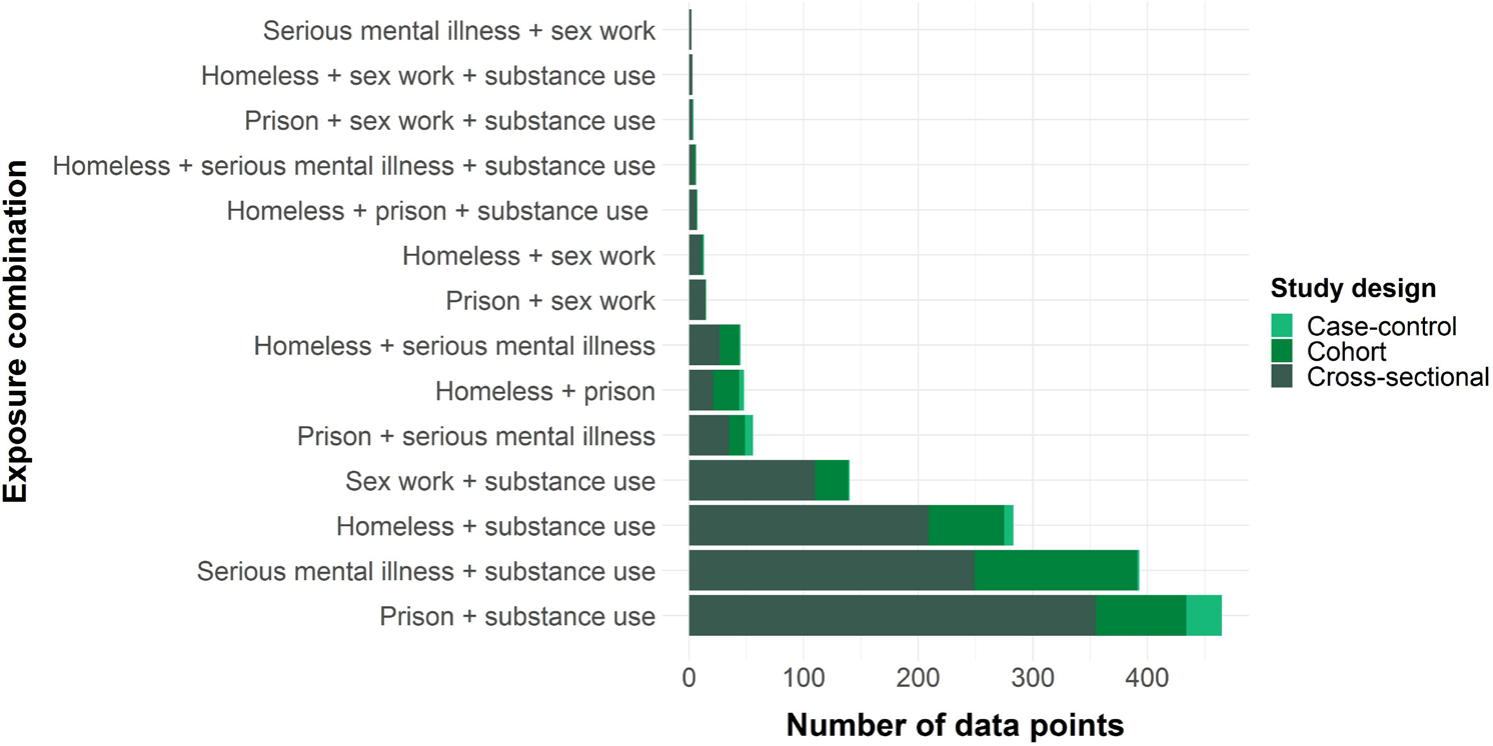
Number of data points by exposure combination and study design.

The most common exposure combinations were imprisonment/substance use and SMI/substance use, accounting for 31% (n=465) and 27% (n=393) of data points, respectively, followed by homelessness/substance use (19%; n=283). Only four of the possible 10 combinations of three exposures had any available data points; no data points were identified relating to four or more exposures (figure 2).

With regard to outcomes, 77% (n=1139) data points are related to morbidity, 16% (n=239) to mortality and 7% (n=101) to self-reported health or quality of life. The most common ICD-10 chapters were infections (chapter 1: 40%; n=587), mental and behavioural disorders (chapter 5: 16%; n=236) and external causes (chapters 19 and 20 combined: 15%; n=227).

**Figure 3:**
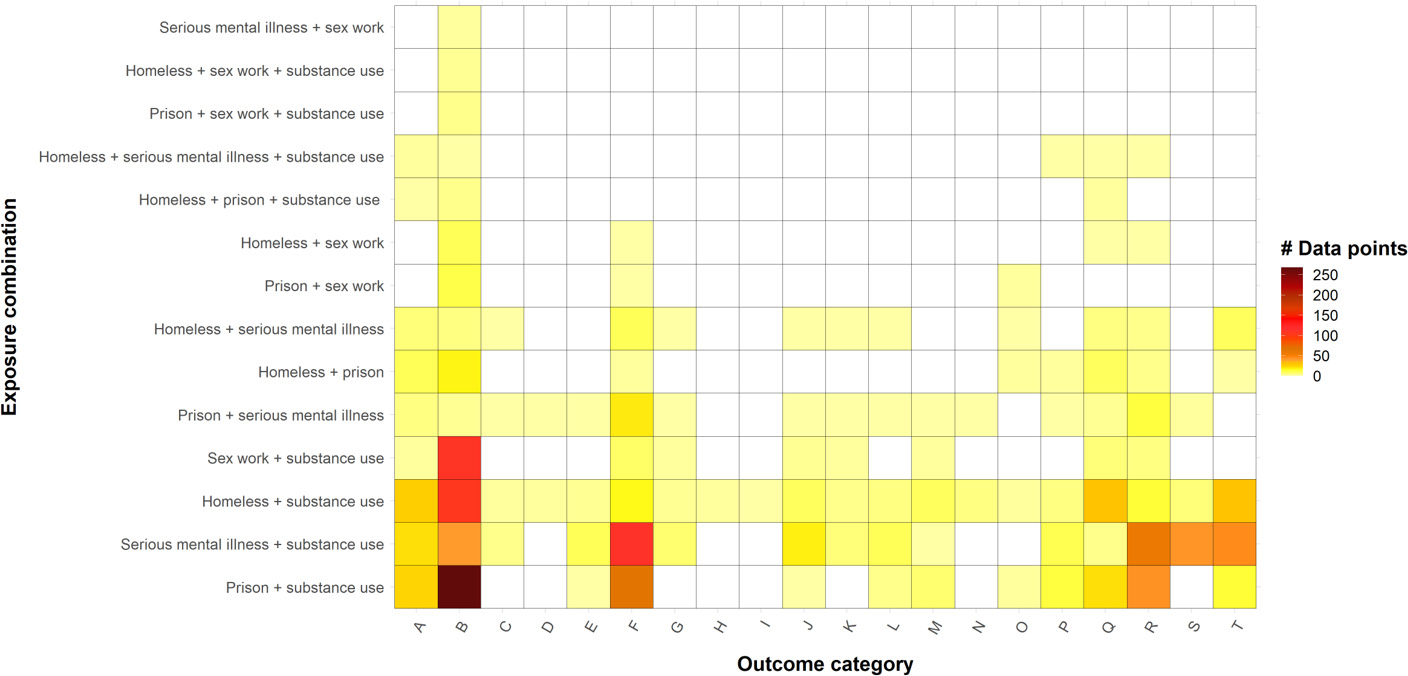
Heat map showing distribution of data points by exposure combination and outcome category.

Figure 3 shows the distribution of data points by exposure combination and outcome category (organised by ICD-10 chapter). It illustrates the dominance of a limited number of outcome types—above all, infectious diseases—and the lack of any data for some exposure combinations on common conditions such as circulatory, respiratory and metabolic disorders. Across all exposure combinations, blood-borne viruses (BBV) accounted for 31% (n=456) of total data points (online supplemental figure A3.2 in appendix 3).

Overall, 80% (n=1190/1480) of data points showed an association between multiple exposures and poorer health outcomes: after restricting to studies at low RoB, this rose to 86% (n=801/932). Sign testing of effect direction gave p values of <0.001 in both cases, providing evidence to reject the null hypothesis of no association.

In presenting the results of our narrative and quantitative synthesis, we focus on all-cause mortality; cause-specific mortality and morbidity from infections, external causes, and non-communicable diseases (NCDs); and self-rated health and quality of life. Effect direction plots and meta-analysis results for other outcomes are shown in online supplemental appendix 3 and 4, respectively. Heterogeneity was high in most meta-analyses undertaken and did not appear to be explained by assessed RoB.

**Figure 4:**
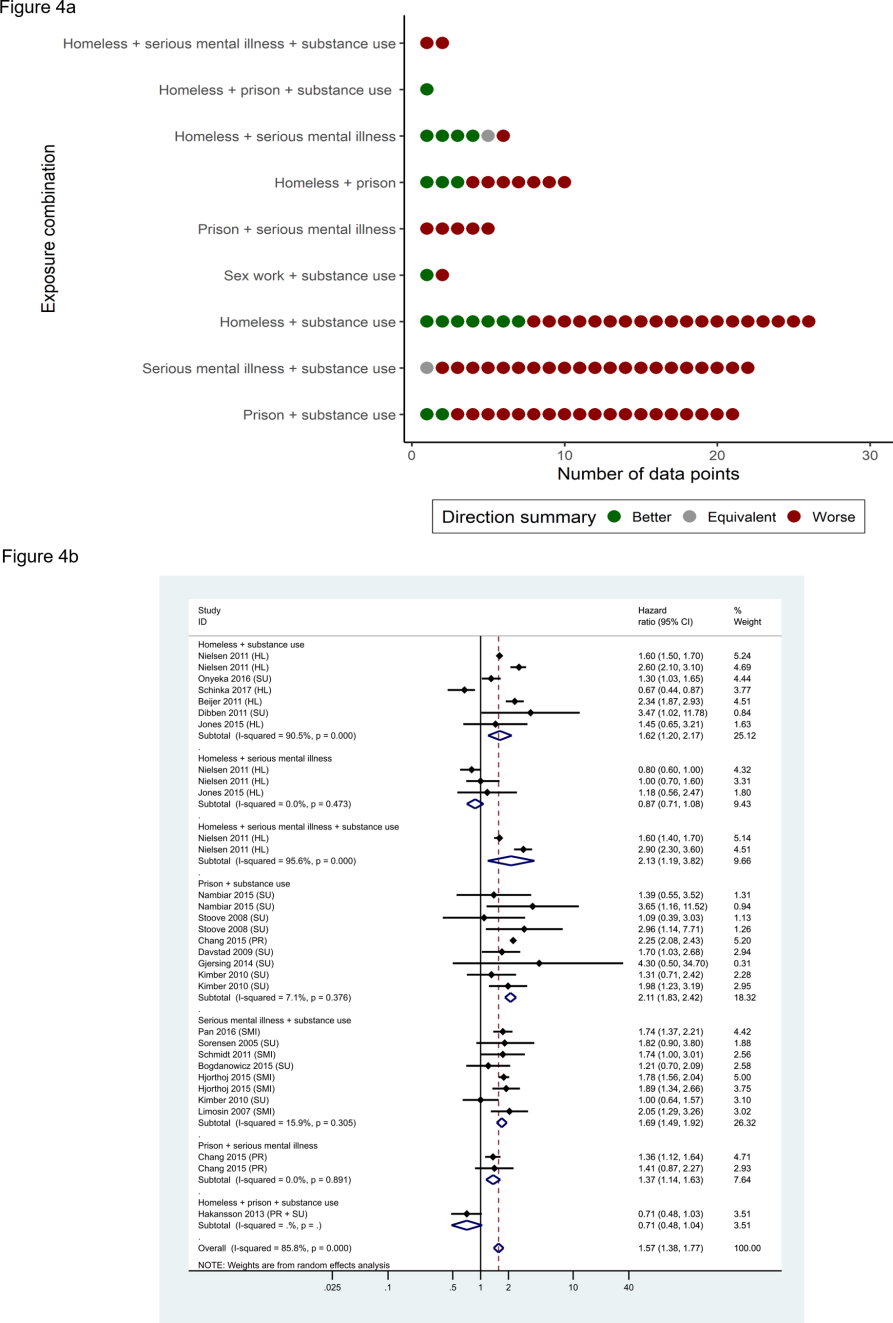
(A) Summary effect direction plot for all-cause mortality. (B). Forest plot for meta-analysis of data points reporting all-cause mortality using HRs, by exposure combination.

With regard to all-cause mortality, 79% (n=75/95) data points showed an association between multiple exposures and increased risk (sign test for effect direction <0.001; figure 4A). Figure 4B shows the pooled results for studies reporting HRs, the most commonly reported effect measure. The pooled point estimate of 1·57 (95% CI: 1·38 to 1·77) was similar to those obtained for other effect measures, and exclusion of studies at high RoB did not materially affect the results (online supplemental appendix 4).

**Figure 5:**
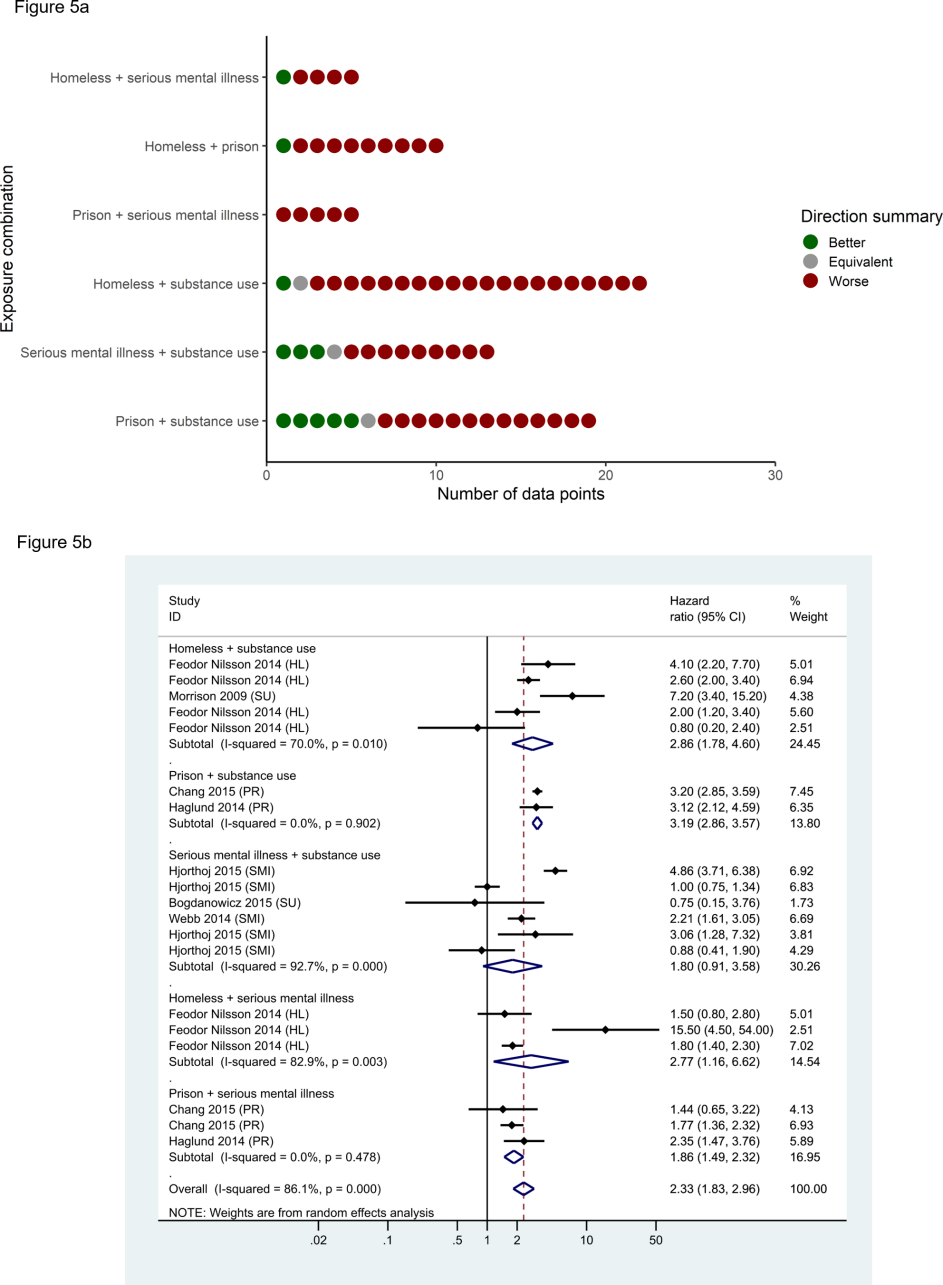
(A) Summary effect direction plot for external mortality (ICD-10 chapters 19 and 20) (B). Forest plot for meta-analysis of data points reporting external-cause mortality using HRs, by exposure combination.

For external-cause mortality (ICD-10 chapters 19 and 20), again, the majority (81%; n=60/74) of data points suggested greater risk among those with multiple versus fewer exposures (sign test for effect direction p<0.001; figure 5A). Pooled measures indicated a stronger association than for all-cause mortality, evident across all exposure combinations (figure 5B). Again, results were similar across effect measures and after stratification by assessed RoB (online supplemental appendix 4). Similarly, with regard to BBV prevalence, 87% (n=394/452) data points showed an association between multiple exposures and higher prevalence (sign test for effect direction p<0.001; online supplemental appendix 3). Pooled measures indicated a strong association including after stratification by assessed RoB (online supplemental appendix 4).

Fewer data points were available for NCDs: effect direction plots and sign testing did not identify an association between multiple exclusions and NCDs overall (online supplemental appendix 3), though the burden of respiratory disease did appear to be significantly higher (sign test p=0.016). Variation in outcome measures and time periods meant that meta-analyses were small and potentially underpowered but showed similar results (online supplemental appendix 4).

With regard to self-rated health and quality of life, 71% (n=71/100) data points for this outcome type suggested poorer outcomes among people experiencing multiple exclusions (sign test p<0.001), but this proportion varied by exposure combinations (online supplemental appendix 3). Meta-analysis of these outcomes was not possible due to variation in the instruments used and limitations in reporting.

Relatively few data points were available for gender-stratified analyses. Exploratory analyses did not suggest a consistent difference between genders in associations between multiple exclusions and health outcomes (online supplemental appendix 4).

## Discussion

Our systematic review demonstrates that existing evidence on the association between multiple exclusions and health is dominated by cross-sectional studies examining a limited number of exposure combinations and outcomes. In particular, we found there is a predominance of studies on infectious disease, mental illness and external causes of morbidity and mortality. Few studies have examined combinations involving sex work or more than two of these experiences. Results of our narrative and quantitative synthesis suggested that multiple exclusions are associated with increased all-cause and external-cause mortality, as well as higher prevalence of BBV. For NCDs, few data points were available, and associations varied by NCD type, exposure combination and outcome measure.

The skew of previous research towards specific exposure combinations and outcomes means that available evidence may not reflect the population overlap between these experiences or conditions causing the greatest burden of ill health. For instance, multiple exclusions appear to be associated with a higher risk of some NCDs, which may translate into a substantial population burden, yet these conditions were relatively understudied. These populations may therefore be further disadvantaged by evidence gaps on potentially important health needs.

Nonetheless, the available data demonstrate stark health inequalities. An estimated, 57% greater hazard of mortality associated with multiple exclusions, beyond the eightfold to 12-fold differential seen between people with any one of such experience and the rest of the population,^3^ suggests extreme health disadvantage among these populations. The findings of excess risk of infections, and of comorbidities such as respiratory disease, are especially noteworthy in the context of the current COVID-19 pandemic.

The mechanisms by which intersecting forms of social exclusion may influence health are likely to be complex. Multiple exclusions may worsen health through multiplicative or additive risks or even improve it by enabling access to services with beneficial effects. Alternatively, the combination of these experiences may pose no additional risk (particularly where background risk is already high) or merely represent a marker for other forms of cumulative adversity with effects on health, such as extreme poverty.

The strengths of this review include its comprehensive scope, systematic and transparent approach and use of best practice guidelines for narrative synthesis.^22^ To our knowledge, no other review to date has attempted to synthesise the evidence of health outcomes associated with multiple forms of social exclusion in this way.

However, a number of limitations to our review should be noted. Given the large number of studies identified, we were unable to explore diversity within exposure categories in detail: for instance, homelessness encompasses a spectrum of housing exclusion from rough sleeping to ‘sofa surfing’. The nature of these experiences—and their relationship with health—is also likely to vary across contexts with different welfare regimes, healthcare systems and legislative approaches: this may further contribute to heterogeneity and merits more detailed investigation.

Another potential limitation is the risk of publication bias. Inspection of funnel plots suggested potential for small-study effects (online supplemental appendix 5), though this may be explained by true heterogeneity or methodological weaknesses of smaller studies.

Our findings cannot be used to draw conclusions about the causal effects of multiple exclusionary experiences, since few of the original studies used designs appropriate to causal inference, and to enhance comparability, our data extraction focused on minimally adjusted measures. Further work is required to establish the extent and nature of potential causal mechanisms.

Nonetheless, descriptive epidemiology can provide insights into ways to mitigate observed health inequalities. For instance, the high rates of external-cause mortality identified here suggest an important role for overdose, suicide and accident prevention interventions in justice settings, temporary accommodation and mental healthcare. Existing services and policies tend to be narrowly focused on single experiences: a phenomenon particularly well documented in mental healthcare, where people with substance use problems are often excluded from services.^24^ Our results suggest a more integrated approach may be warranted. Descriptive epidemiology can also provide baseline data for evaluating policy and service changes with the potential to impact health.^25 26^

Future research in this area would benefit from being informed by conceptual and empirical understandings of multiple exclusions: for instance, by prioritising combinations that are most common or associated with poorest outcomes. There is also a need for more longitudinal research examining more than two overlapping experiences and for a greater focus on the potential burden of NCDs.

However, there are also important opportunities for action on the available evidence. While people affected by multiple exclusions represent a relatively small group within society, the extreme health inequalities identified here mean that their experiences and needs should be an important consideration within healthcare systems, public health and public policy more broadly. This is especially pertinent during the global COVID-19 pandemic, in which these populations face a ‘perfect storm’ of clinical and social vulnerability.^27–29^

## Conclusion

Evidence to date suggests that people affected by multiple exclusionary processes experience profound health inequalities, though there are also important gaps in the research landscape. In particular, there is a need for studies examining a broader range of exposure combinations and outcomes, especially NCDs, and exploring possible causal mechanisms. In the meantime, addressing the health needs of these populations is likely to require co-ordinated action across multiple sectors, such as healthcare, criminal justice, drug treatment, housing and social security.

**What is already known on this subject**

- Previous studies have shown that people experiencing homelessness, imprisonment, sex work, substance use or serious mental illness experience high levels of ill health and premature death.
- However, these experiences are known to overlap substantially in the population, a phenomenon variously referred to as multiple exclusions, severe and multiple disadvantage and multiple and complex needs. There is empirical and theoretical evidence to suggest that this overlap may matter for health.
- The association between more than one of these experiences and health outcomes has not previously been systematically reviewed.

**What this study adds**

- Our synthesis of existing evidence suggests that people affected by more than one of these exclusionary experiences have substantially poorer health outcomes—in terms of mortality, morbidity and self-rated health/quality of life.
- This review has also identified important gaps in the literature. Future research in this field should prioritise longitudinal designs examining a broader range of combinations and outcomes, particularly non-communicable diseases, and exploring heterogeneity within exposures.
- While in population terms the number of people affected by multiple exclusionary processes may be relatively small, they appear to experience extreme health inequalities. Addressing these inequalities is likely to require co-ordinated action across multiple sectors.

10.1136/jech-2020-215975.supp1 Supplementary data

## Data Availability

Data are available on reasonable request. The datasets used and/or analysed during the current study are available from the corresponding author on request. Protocol available from PROSPERO—CRD42018097189.
