# Supplementary material for: Health of people experiencing co-occurring homelessness, imprisonment, substance use, sex work and/or severe mental illness in high-income countries: a systematic review and meta-analysis
Source: J Epidemiol Community Health. 2021 Apr 23;75(10):1010–8. doi: 10.1136/jech-2020-215975 (PMC8458085; doi:10.1136/jech-2020-215975)

BMJ Publishing Group Limited (BMJ) disclaims all liability and responsibility arising from any reliance placed on this supplemental material which has been supplied by the author(s)

Appendix 3. Results – narrative synthesis

Figure A3.1. Map of geographical distribution of studies. Ineligible countries are shown in grey;tudies undertaken in >1 country are not shown on the map but included in all other analyses.

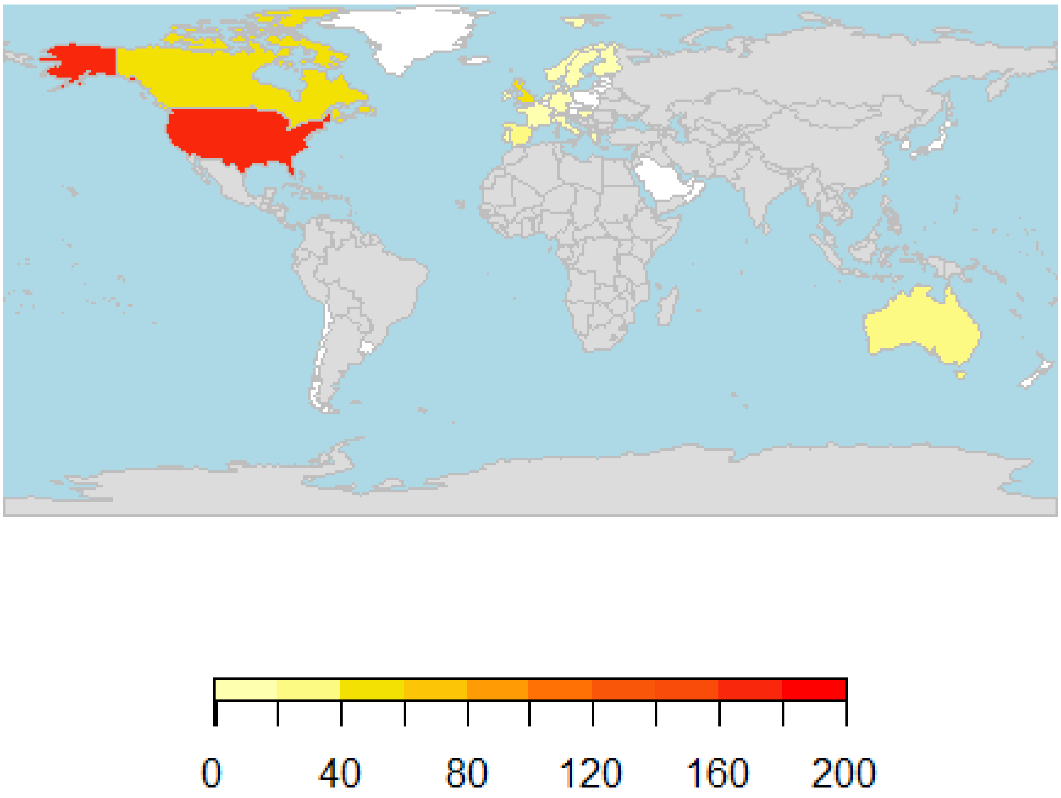

Table A3.1. Risk of bias of included data points, by study design.

Study design	Total points available	Threshold for low risk of bias	Data points scored as low risk (%)	Total data points
Cross-sectional	5	≥4	573 (54.8)	1,045
Case-control	7	≥5	43 (76.8)	56
Cohort	8	≥6	316 (83.8)	379
Total	-	-	932 (63.0)	1,480

Figure A3.2. Number of data points by exposure combination and outcome type: blood-borne virus, other infection, or non-infection-related.

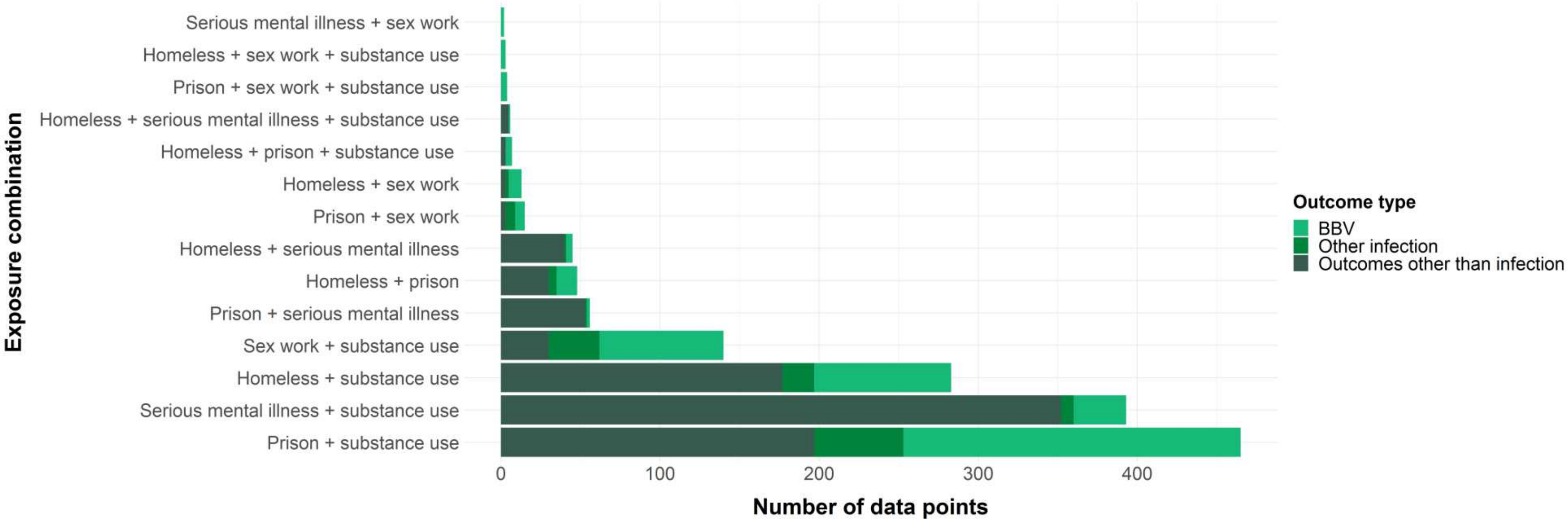

Due to space constraints, exposure combinations for which no data points were identified are not shown.

Figure A3.3. Summary effect direction plot for certain infectious and parasitic diseases (ICD-10 chapter 1)

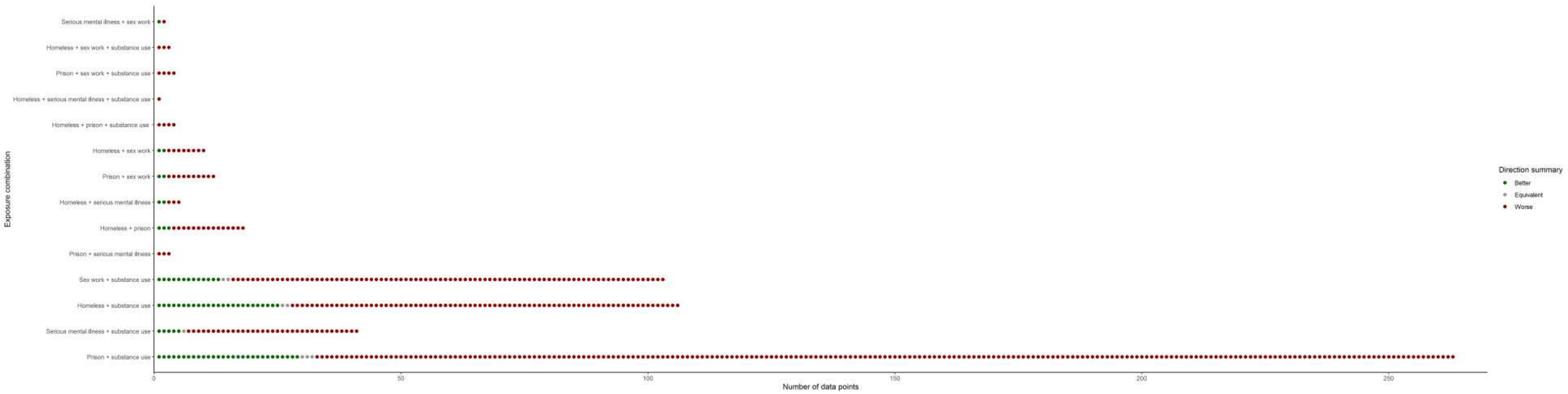

One-tailed sign test for effect direction (poorer outcomes among those with multiple vs fewer exposures): p<0.001

Figure A3.4. Summary effect direction plot for neoplasms (ICD-10 chapter 2)

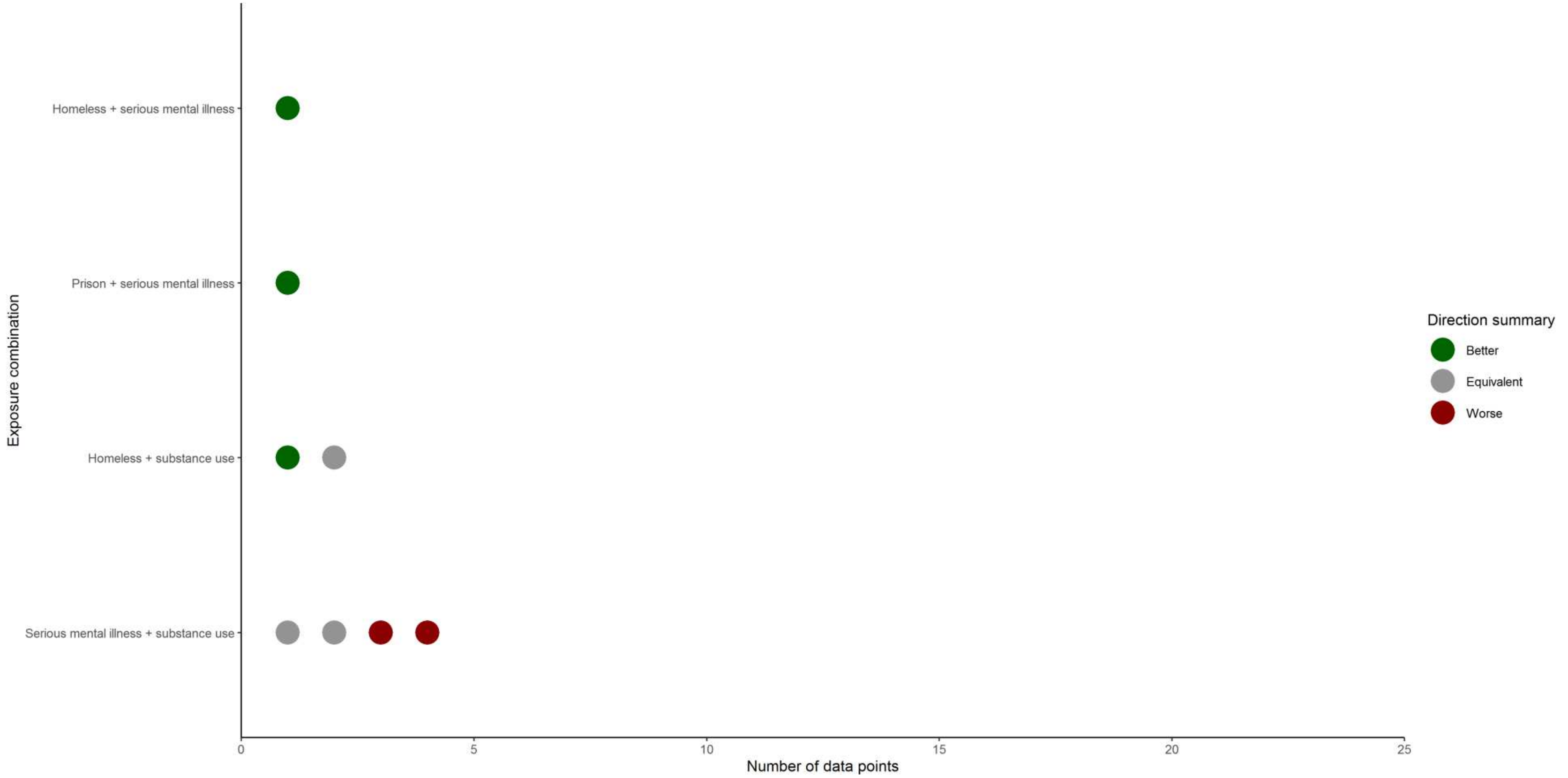

One-tailed sign test for effect direction (poorer outcomes among those with multiple vs fewer exposures): p=0.813

Figure A3.5. Summary effect direction plot for diseases of the blood and blood-forming organs and certain disorders involving the immune mechanism (ICD-10 chapter 3)

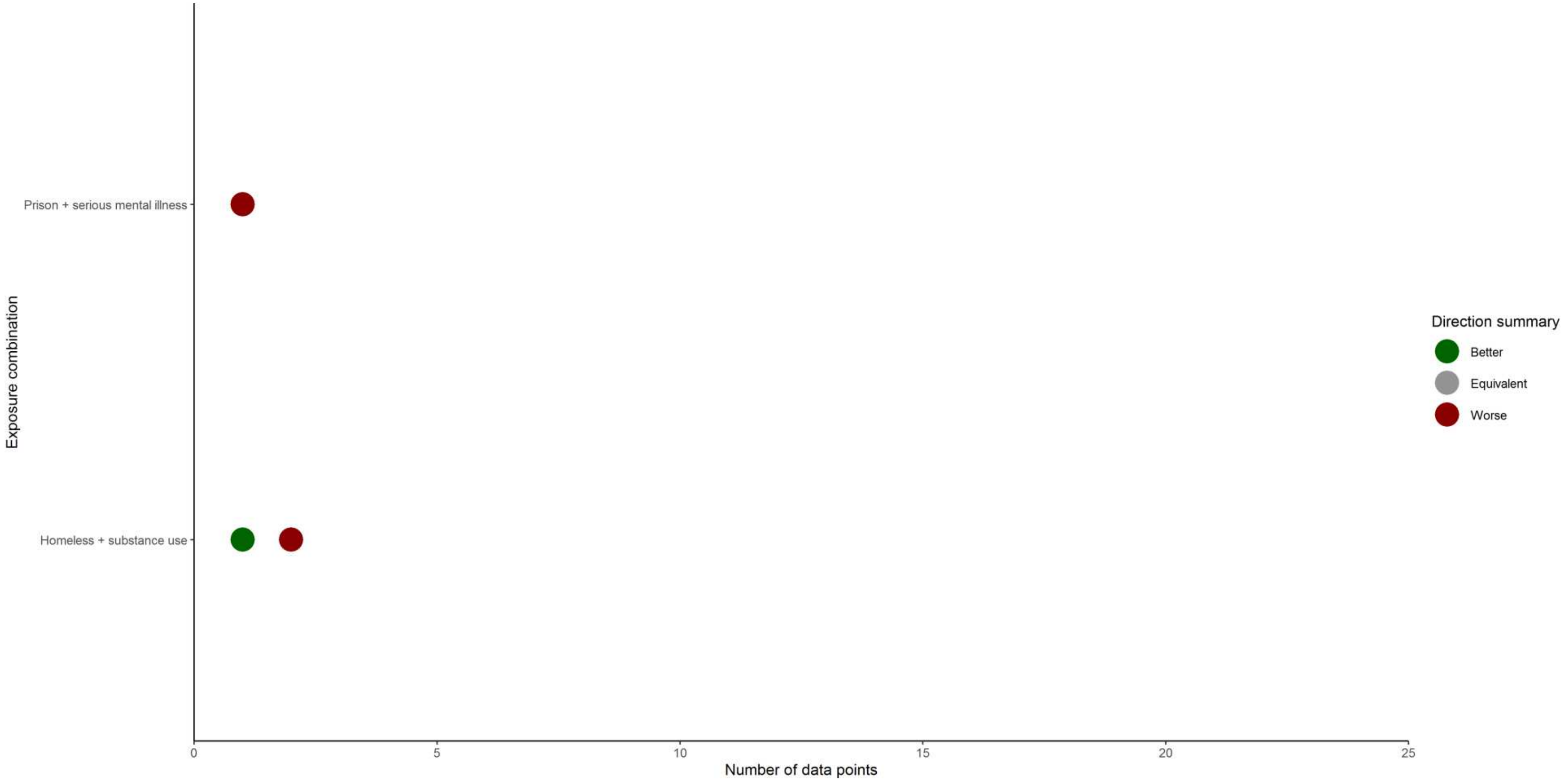

One-tailed sign test for effect direction (poorer outcomes among those with multiple vs fewer exposures): p=0.500

Figure A3.6. Summary effect direction plot for endocrine, nutritional and metabolic diseases (ICD-10 chapter 4)

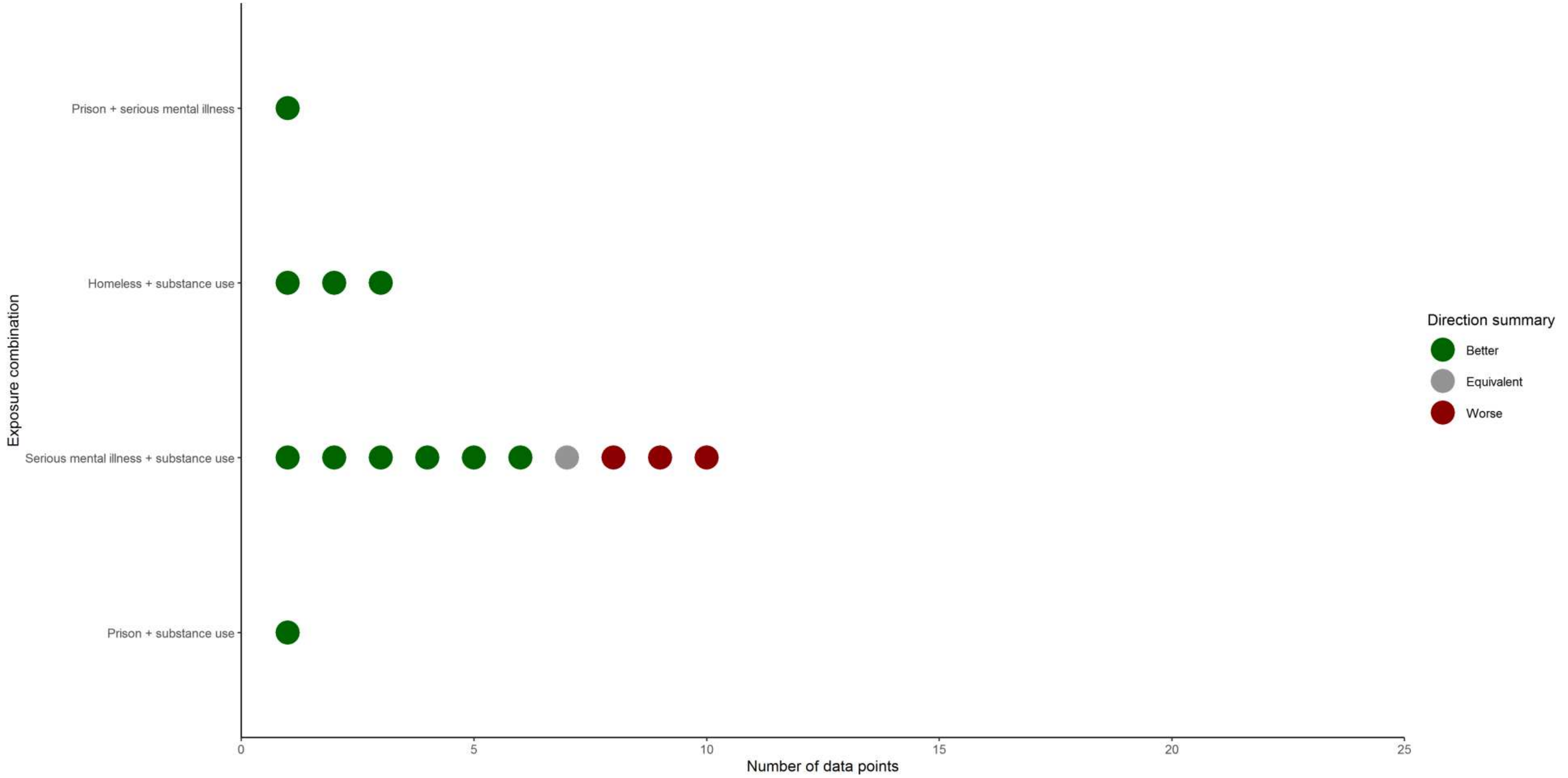

One-tailed sign test for effect direction (poorer outcomes among those with multiple vs fewer exposures): p=0.994

Figure A3.7. Summary effect direction plot for mental and behavioural disorders (ICD-10 chapter 5)

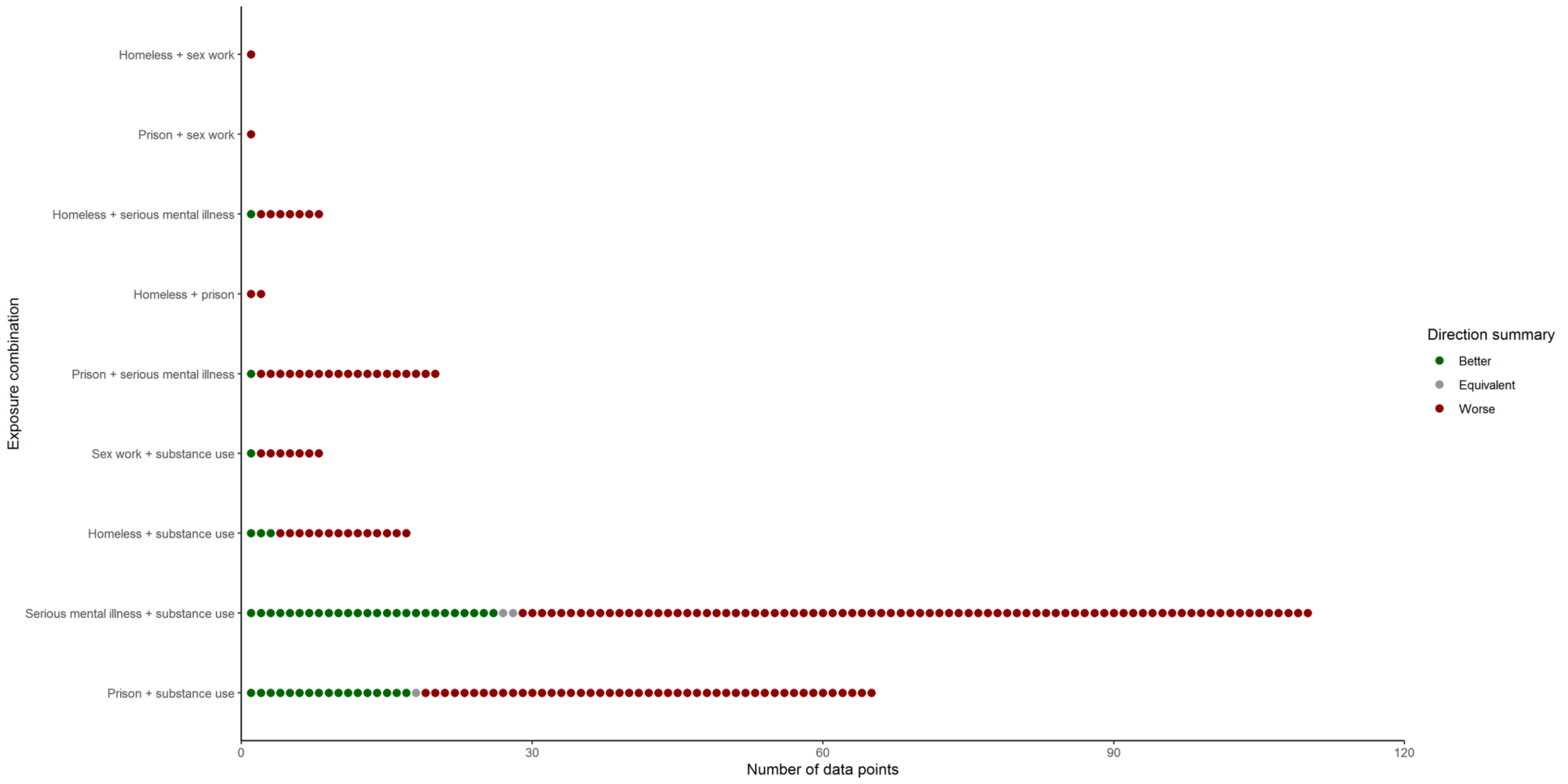

One-tailed sign test for effect direction (poorer outcomes among those with multiple vs fewer exposures): p<0.001

Figure A3.8. Summary effect direction plot for diseases of the nervous system (ICD-10 chapter 6)

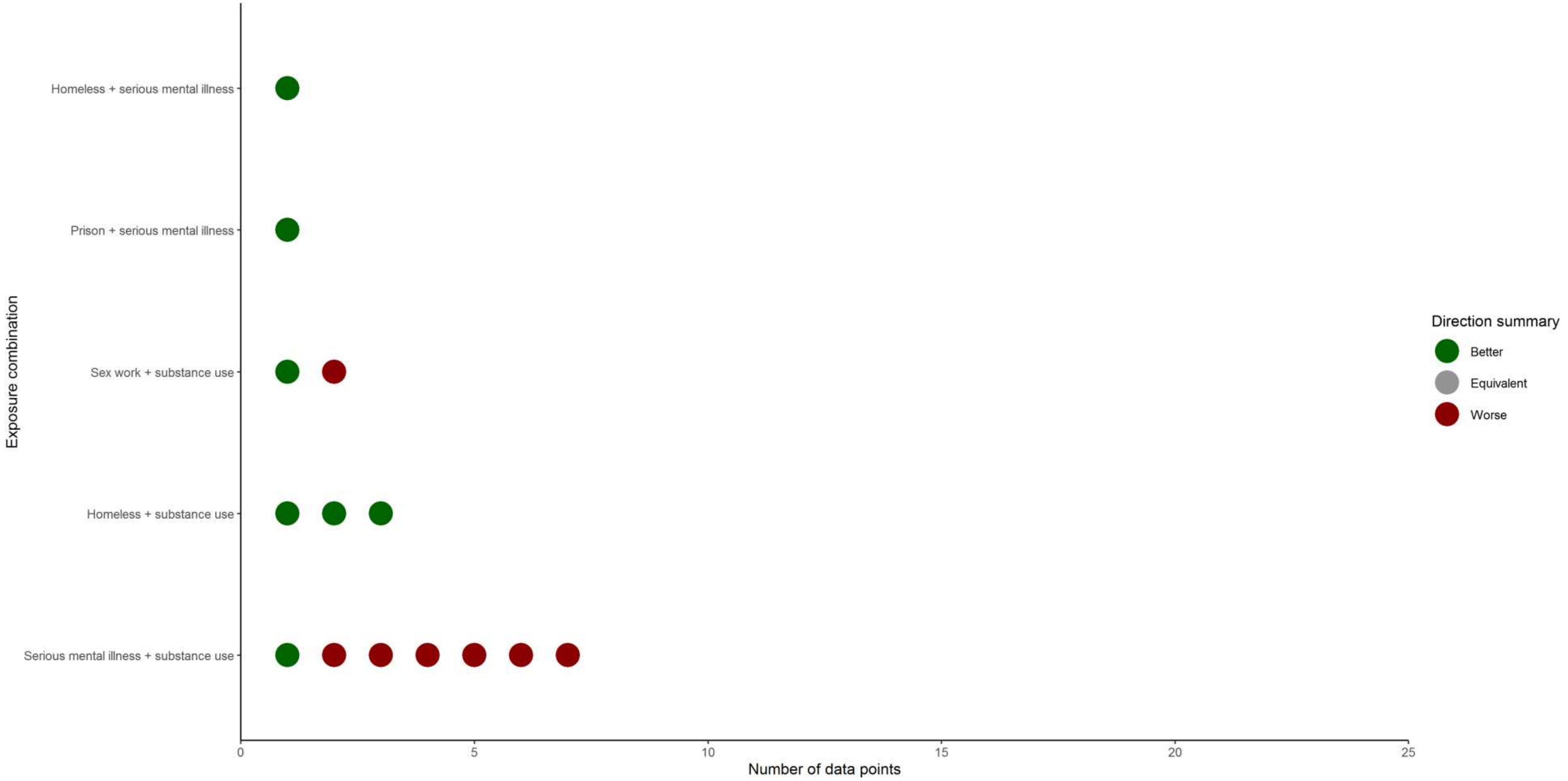

One-tailed sign test for effect direction (poorer outcomes among those with multiple vs fewer exposures): p=0.605

Figure A3.9. Summary effect direction plot for diseases of the eye and adnexa (ICD-10 chapter 7)

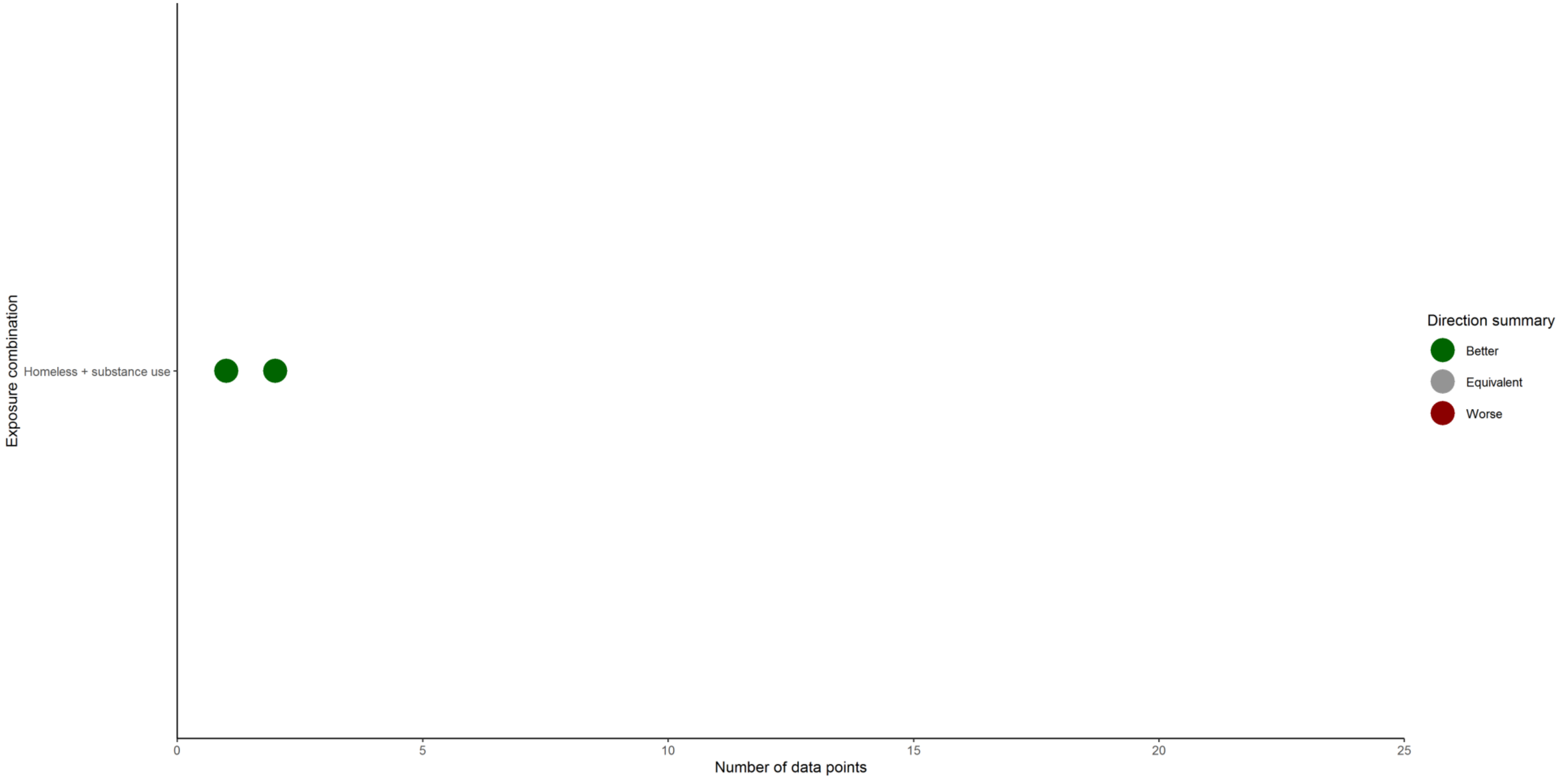

One-tailed sign test for effect direction (poorer outcomes among those with multiple vs fewer exposures): p=1.000

Figure A3.10. Summary effect direction plot for diseases of the ear and mastoid process (ICD-10 chapter 8)

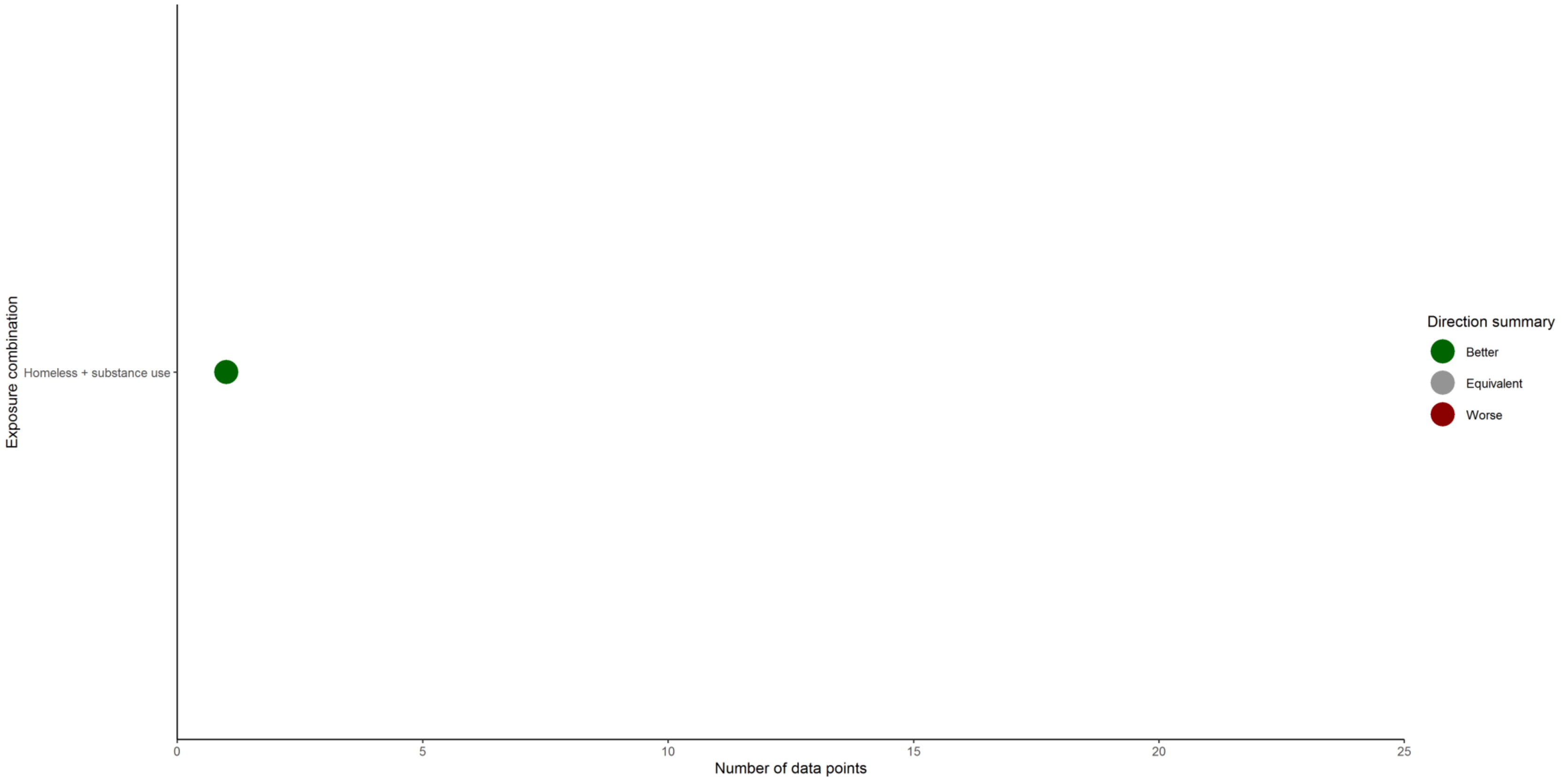

One-tailed sign test for effect direction (poorer outcomes among those with multiple vs fewer exposures): p=1.000

Figure A3.11. Summary effect direction plot for diseases of the circulatory system (ICD-10 chapter 9)

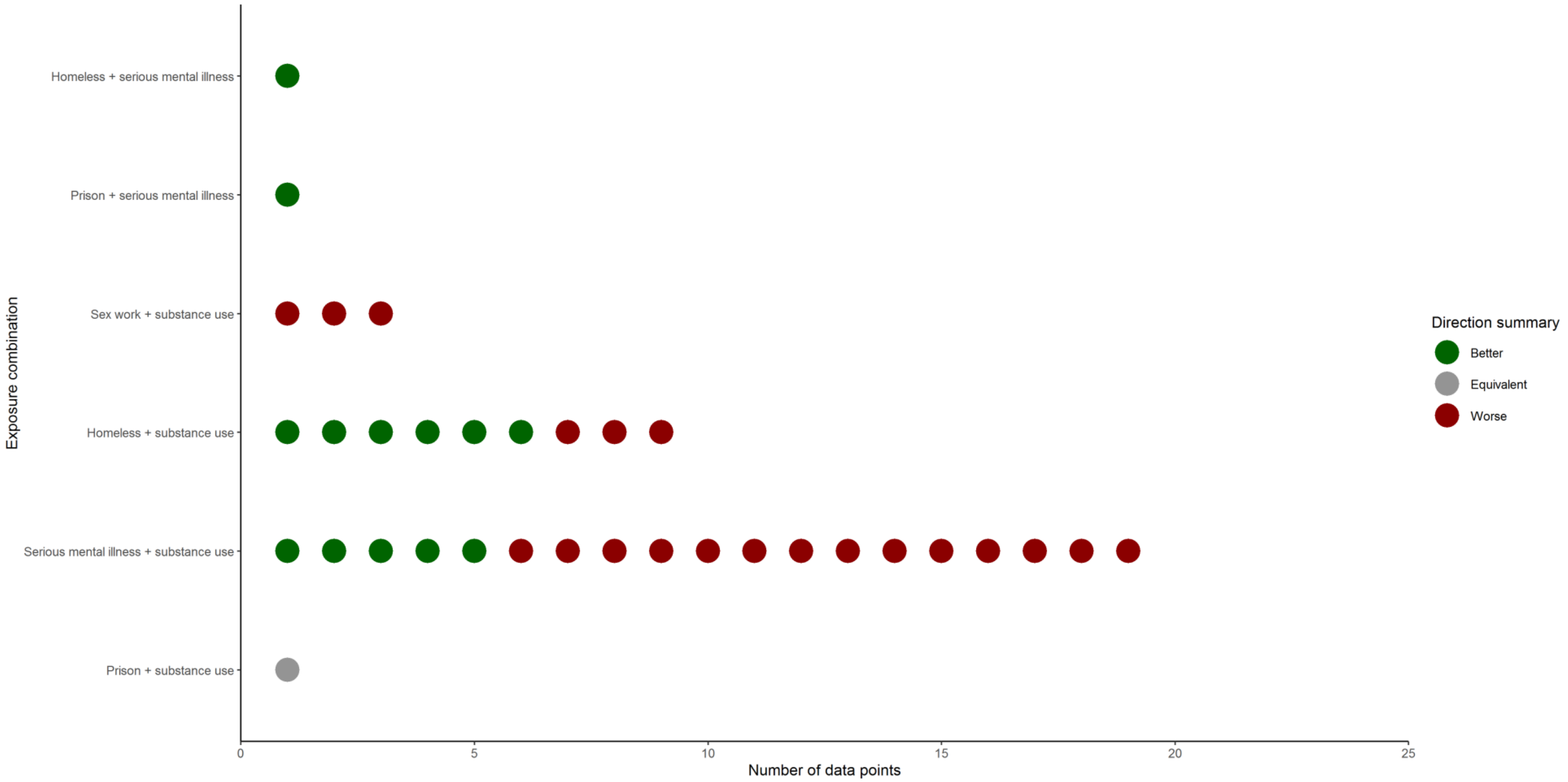

One-tailed sign test for effect direction (poorer outcomes among those with multiple vs fewer exposures): p=0.148

Figure A3.12. Summary effect direction plot for diseases of the respiratory system (ICD-10 chapter 10)

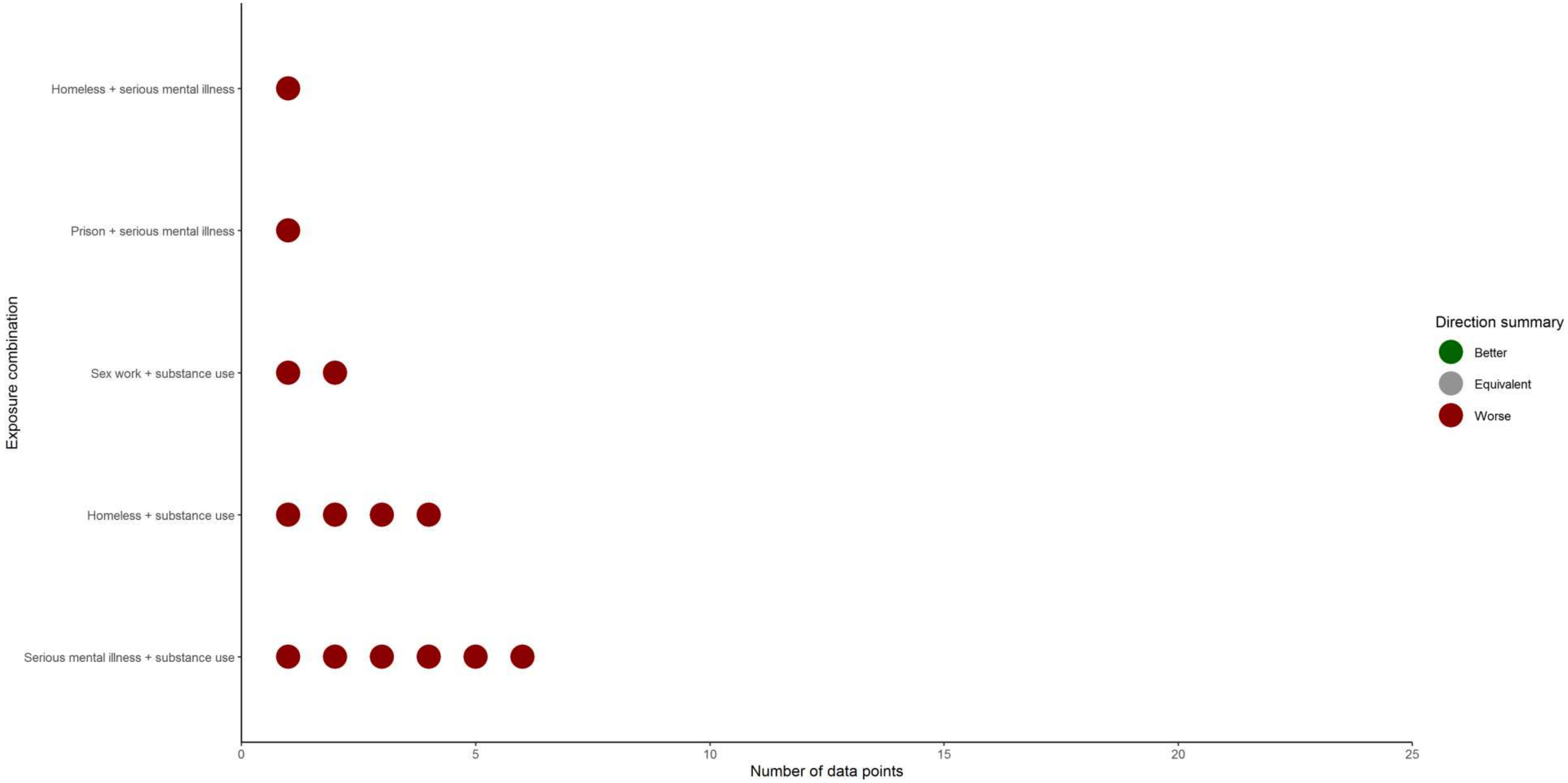

One-tailed sign test for effect direction (poorer outcomes among those with multiple vs fewer exposures): p<0.001

Figure A3.13. Summary effect direction plot for diseases of the digestive system (ICD-10 chapter 11)

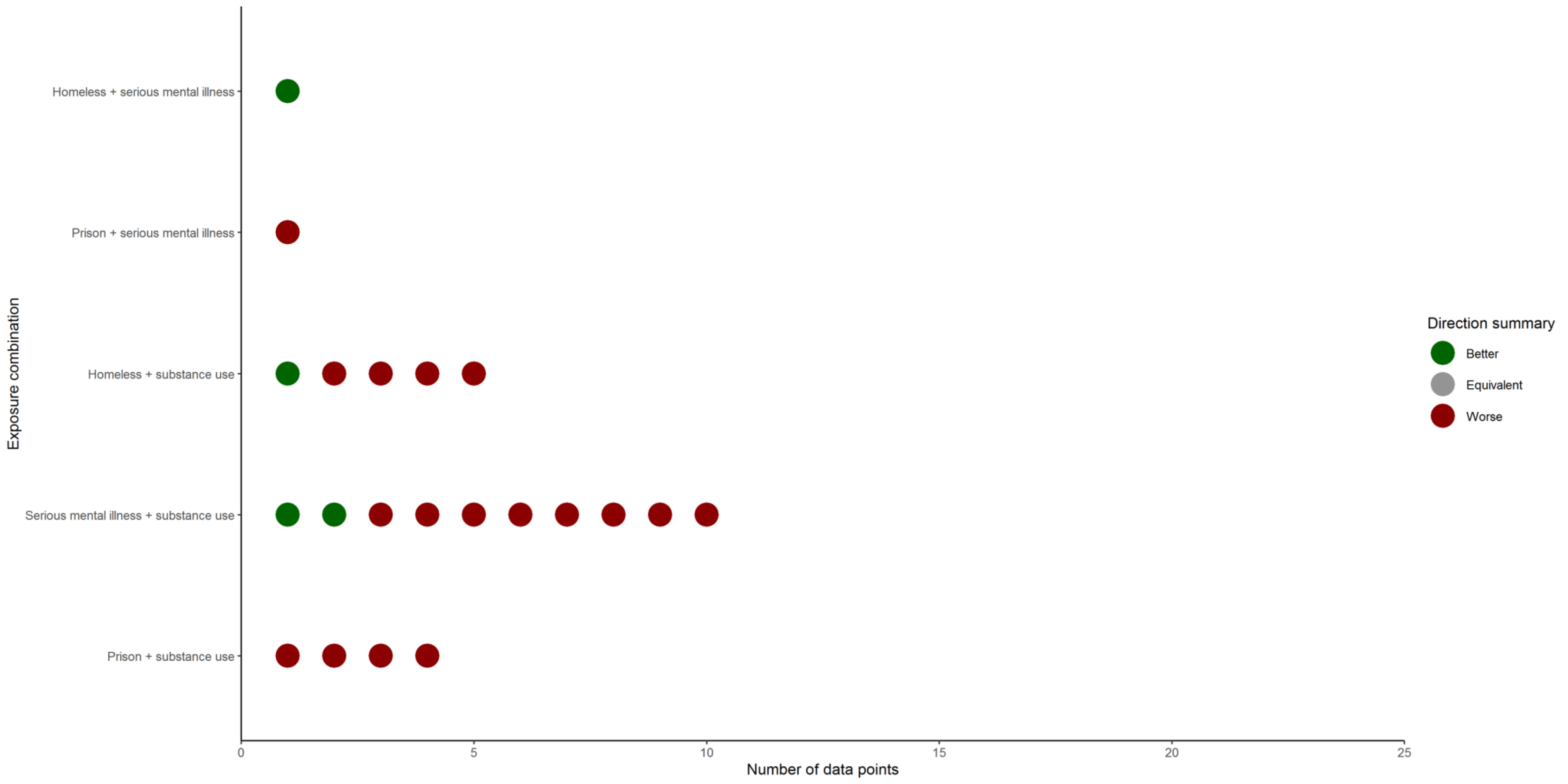

One-tailed sign test for effect direction (poorer outcomes among those with multiple vs fewer exposures): p=0.004

Figure A3.14. Summary effect direction plot for diseases of the skin and subcutaneous tissue (ICD-10 chapter 12)

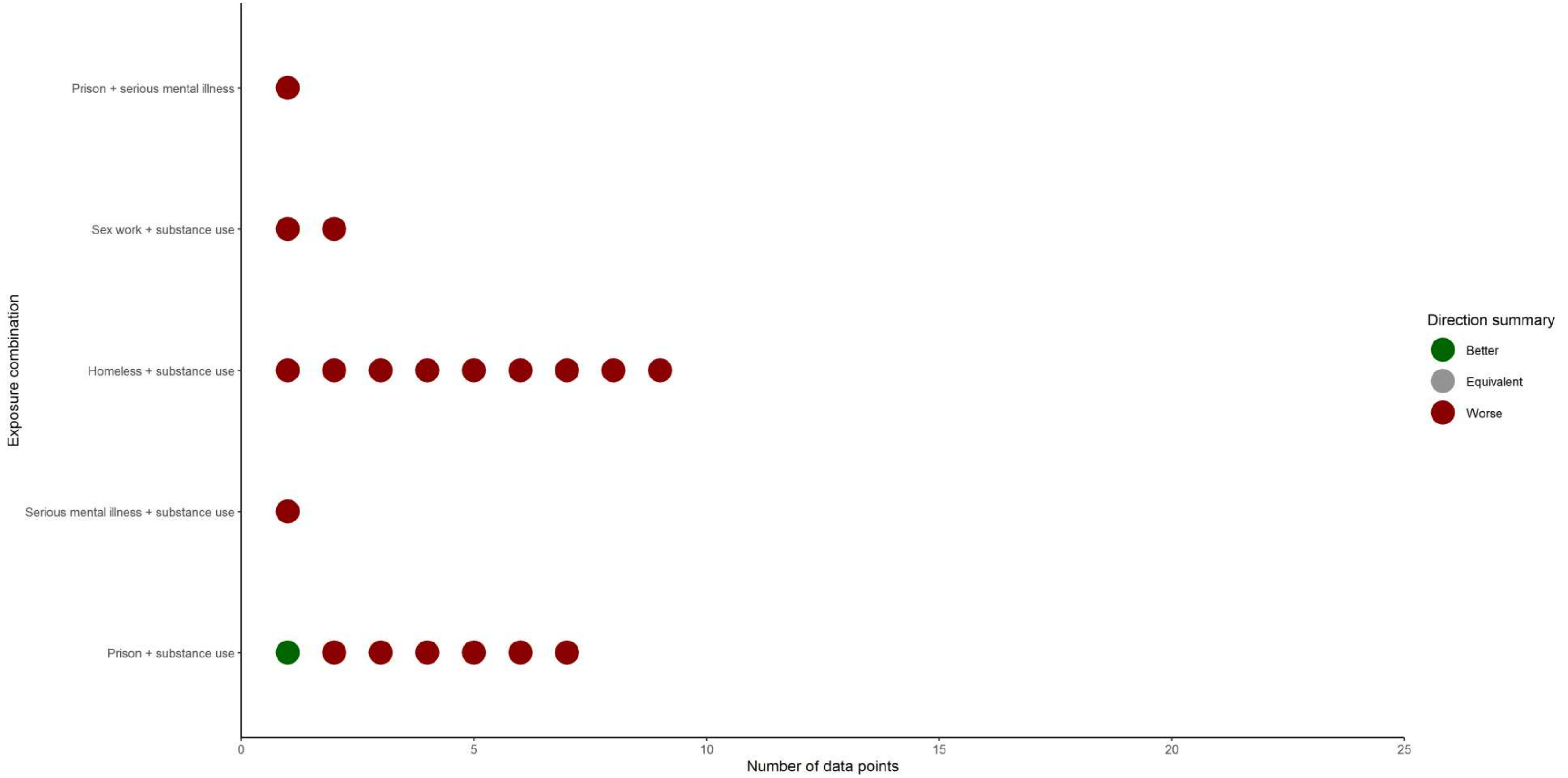

One-tailed sign test for effect direction (poorer outcomes among those with multiple vs fewer exposures): p<0.001

Figure A3.15. Summary effect direction plot for diseases of the musculoskeletal system and connective tissue (ICD-10 chapter 13)

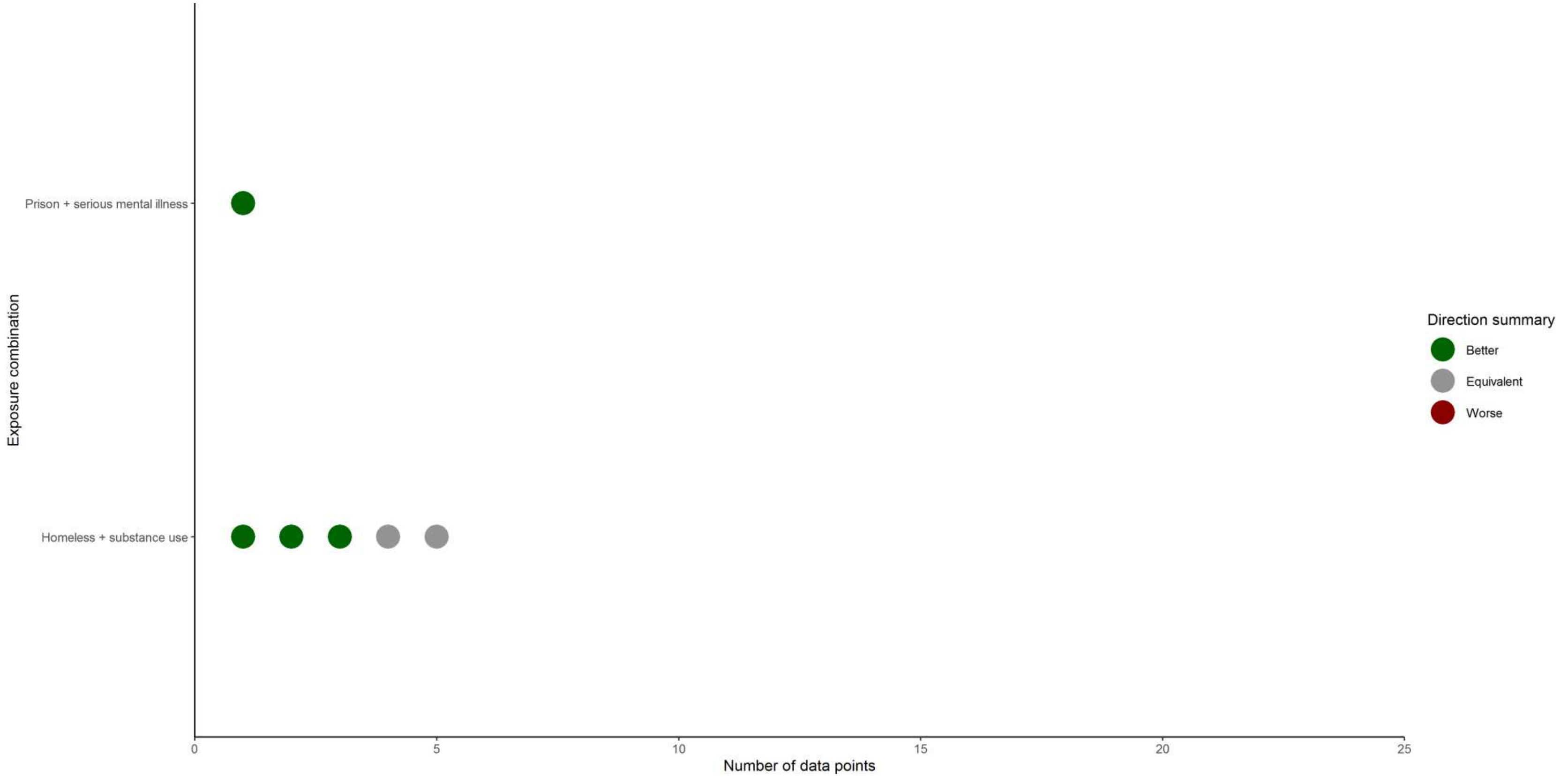

One-tailed sign test for effect direction (poorer outcomes among those with multiple vs fewer exposures): p=1.000

Figure A3.16. Summary effect direction plot for diseases of the genitourinary system (ICD-10 chapter 14)

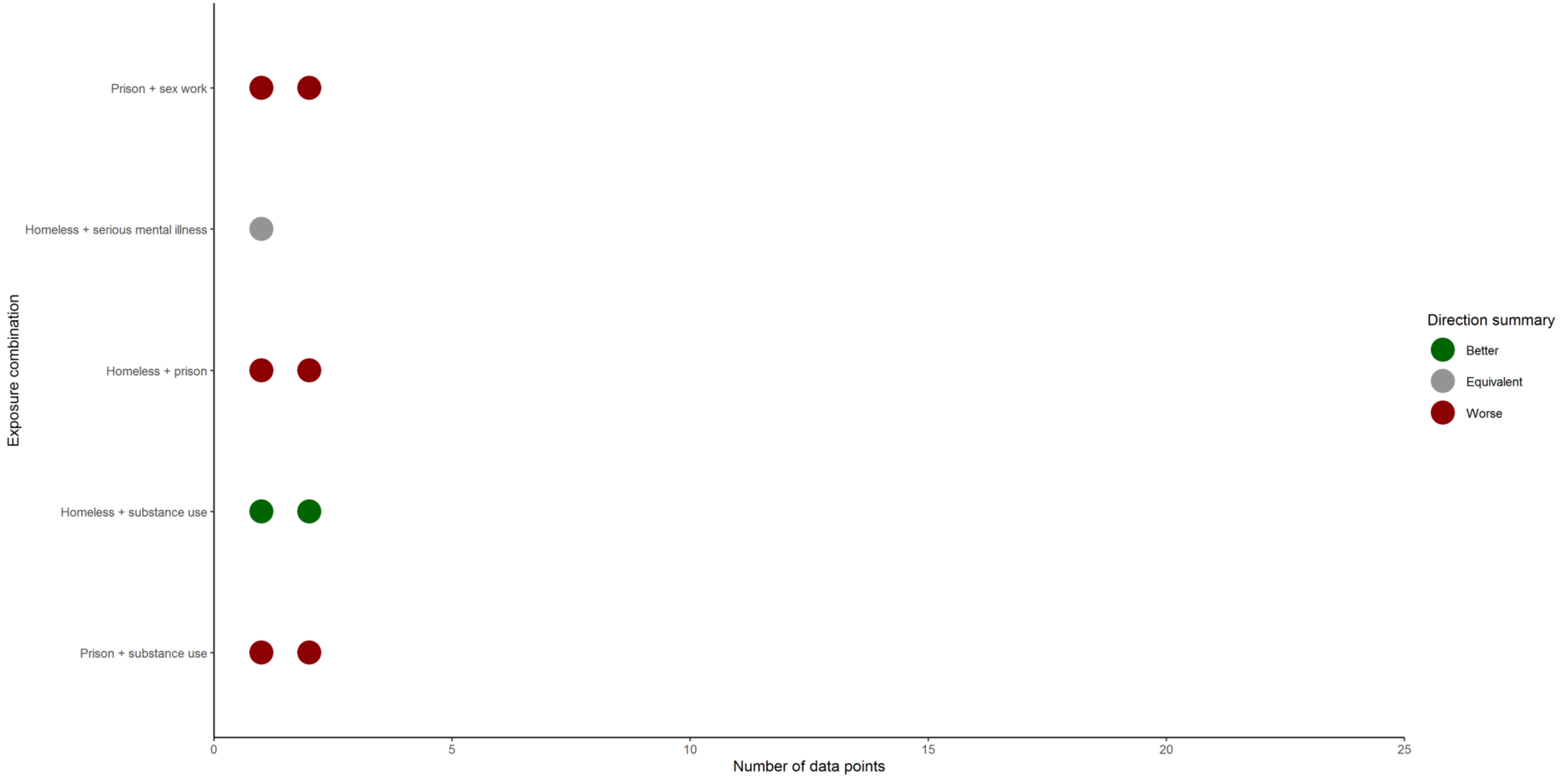

One-tailed sign test for effect direction (poorer outcomes among those with multiple vs fewer exposures): p=0.145

Figure A3.17. Summary effect direction plot for symptoms, signs, and abnormal clinical and laboratory findings not elsewhere classified (ICD-10 chapter 18)

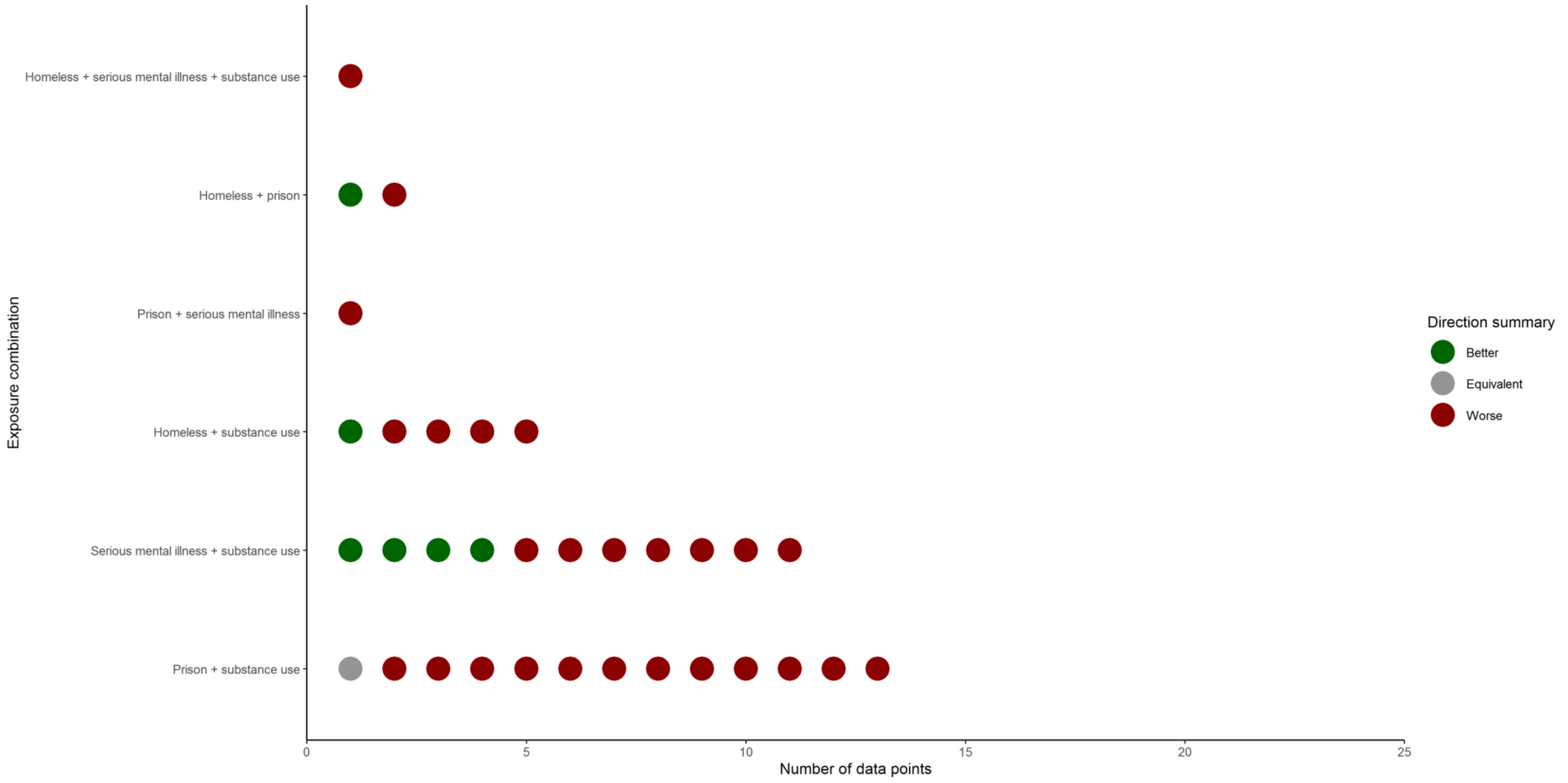

One-tailed sign test for effect direction (poorer outcomes among those with multiple vs fewer exposures): p<0.001

Figure A3.18. Summary effect direction plot for injury, poisoning and certain other consequences of external causes (ICD-10 chapter 19)

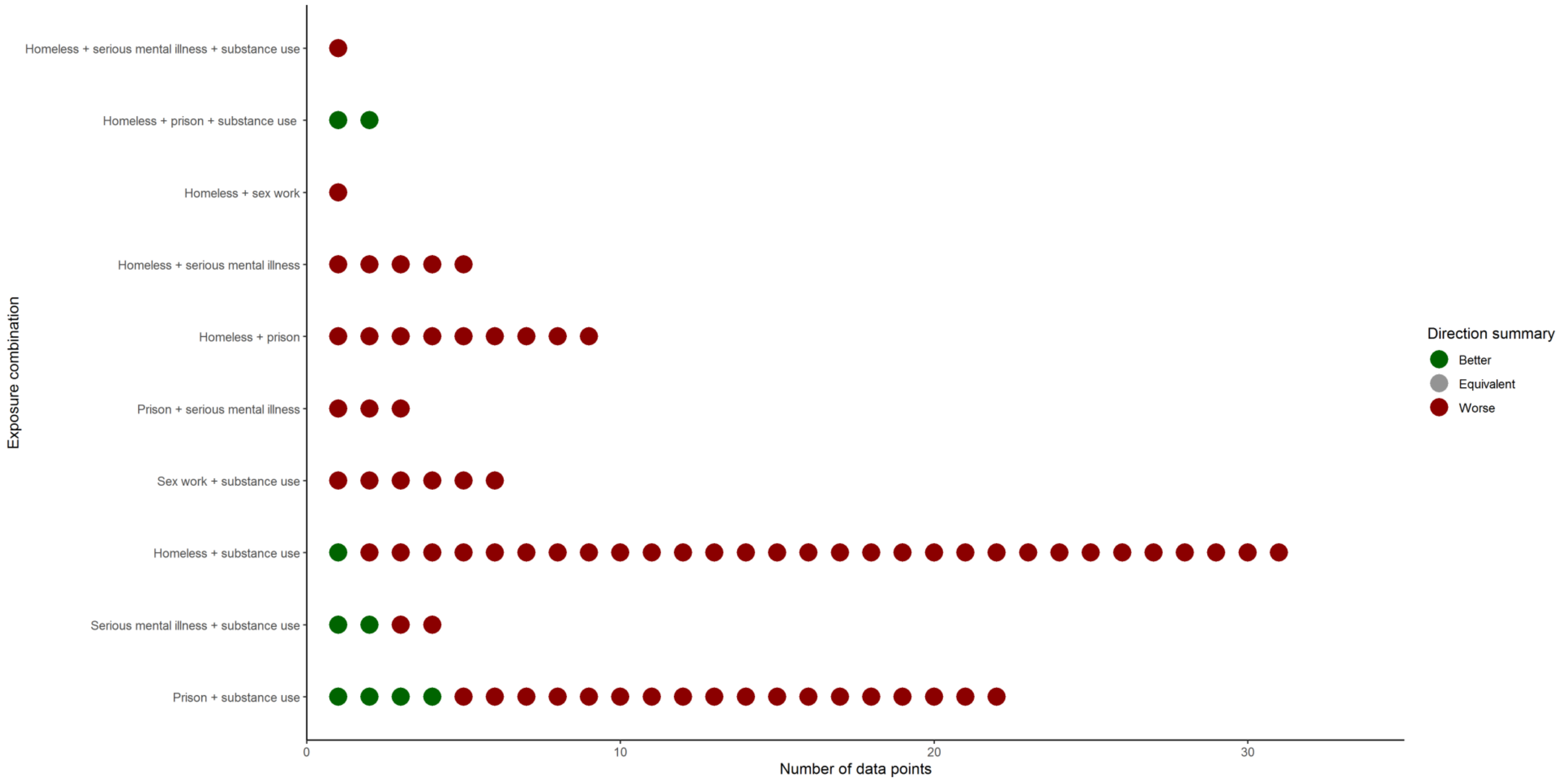

One-tailed sign test for effect direction (poorer outcomes among those with multiple vs fewer exposures): p<0.001

Figure A3.19. Summary effect direction plot for external causes of morbidity and mortality (ICD-10 chapter 20)

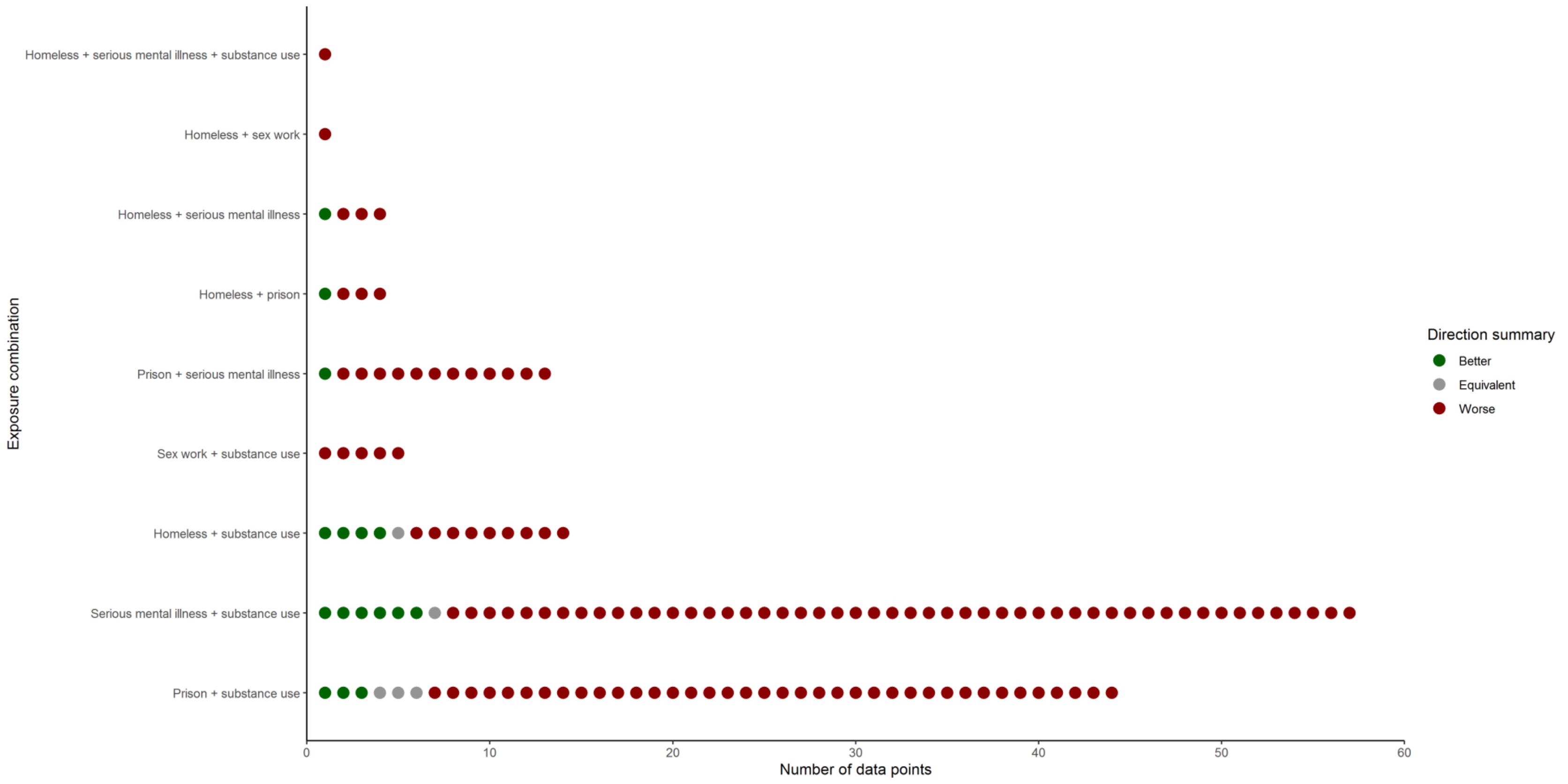

One-tailed sign test for effect direction (poorer outcomes among those with multiple vs fewer exposures): $p<0.001$

Figure A3.20. Summary effect direction plot for self-rated health or quality of life

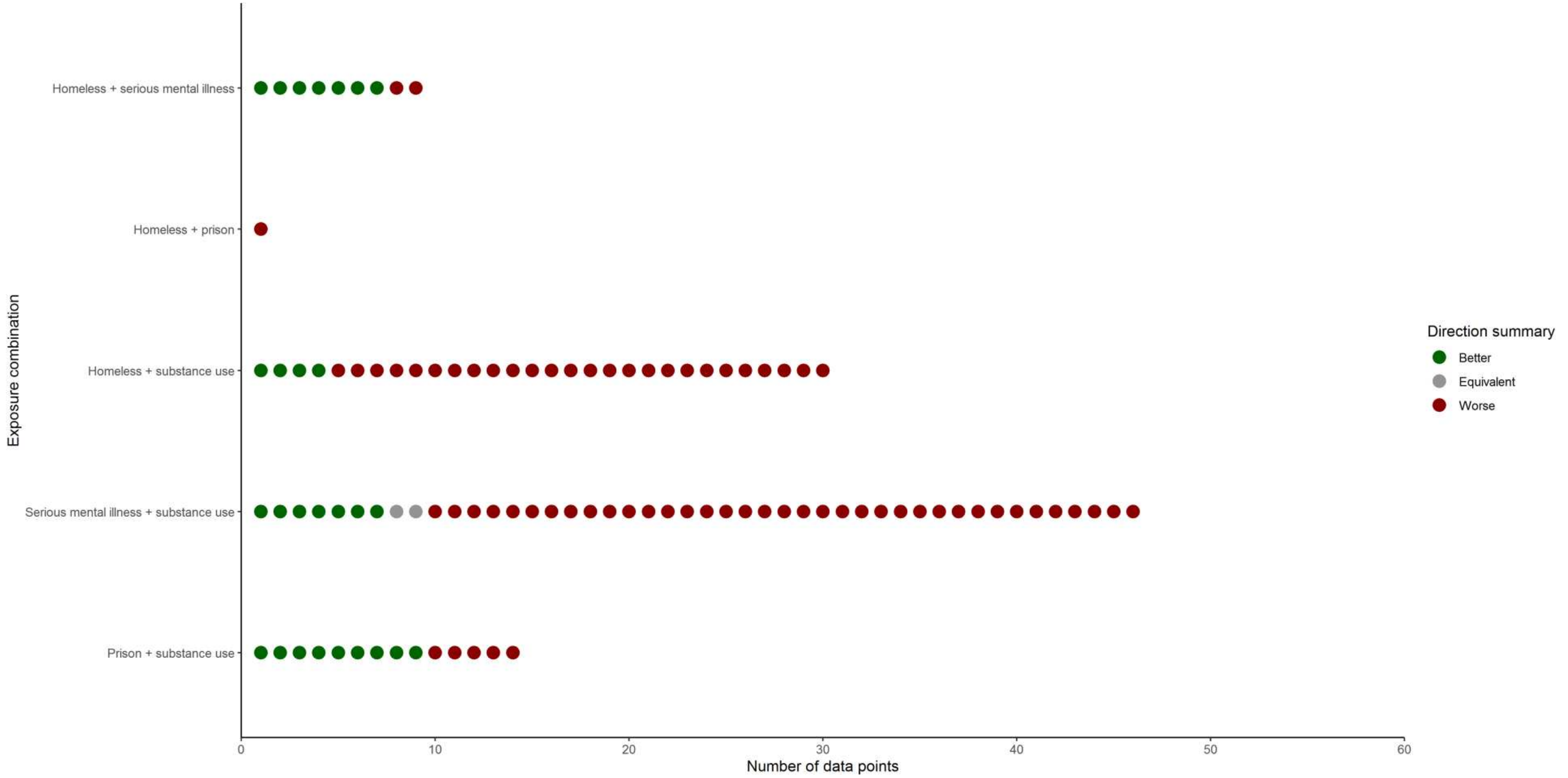

One-tailed sign test for effect direction (poorer outcomes among those with multiple vs fewer exposures): p<0.001

Figure A3.21. Summary effect direction plot for blood-borne viruses

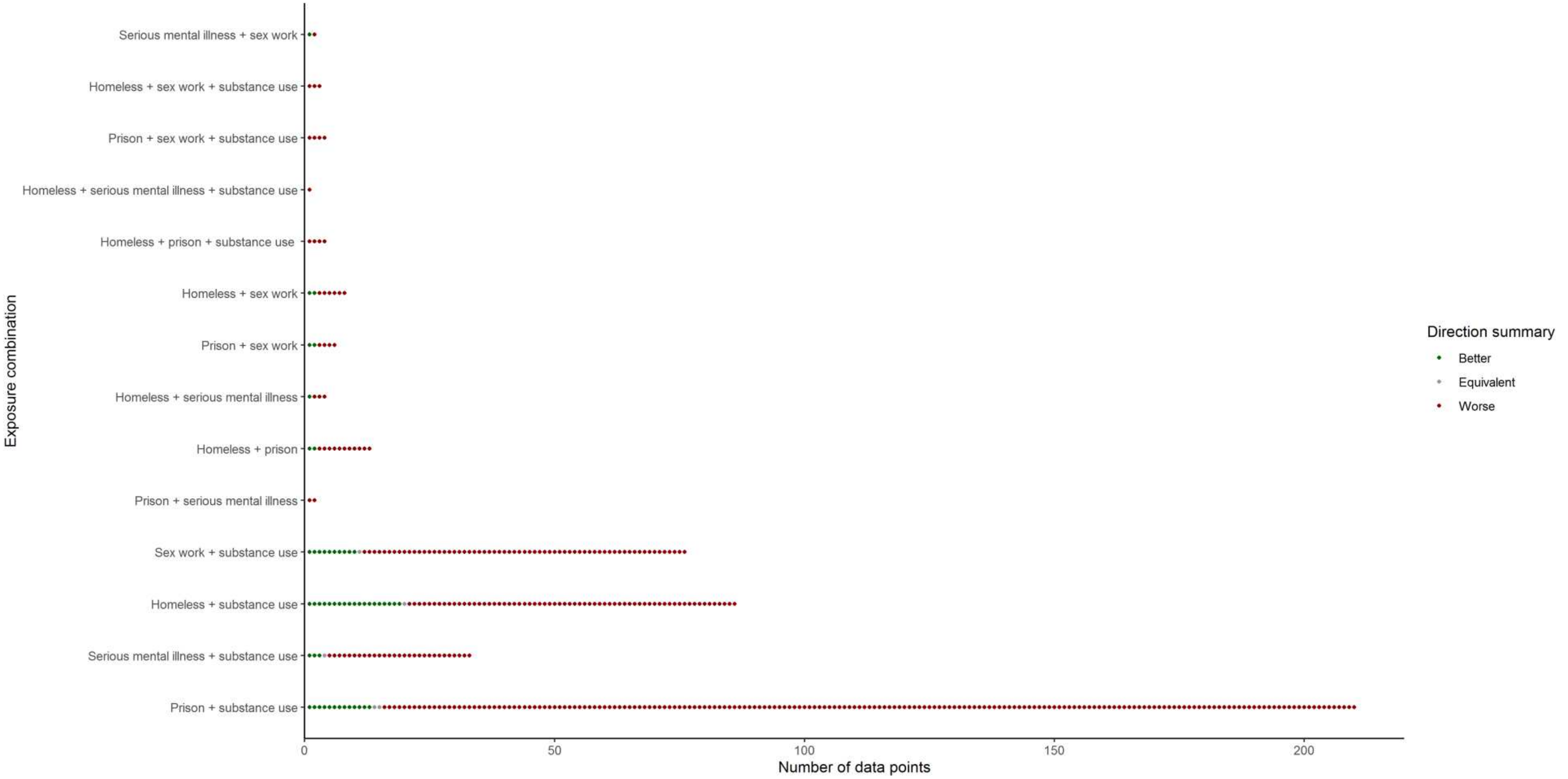

One-tailed sign test for effect direction (poorer outcomes among those with multiple vs fewer exposures): p<0.001

Figure A3.22. Summary effect direction plot for non-communicable diseases (comprising neoplasms, cardiovascular and cardiometabolic disease, chronic respiratory disease, and diabetes)

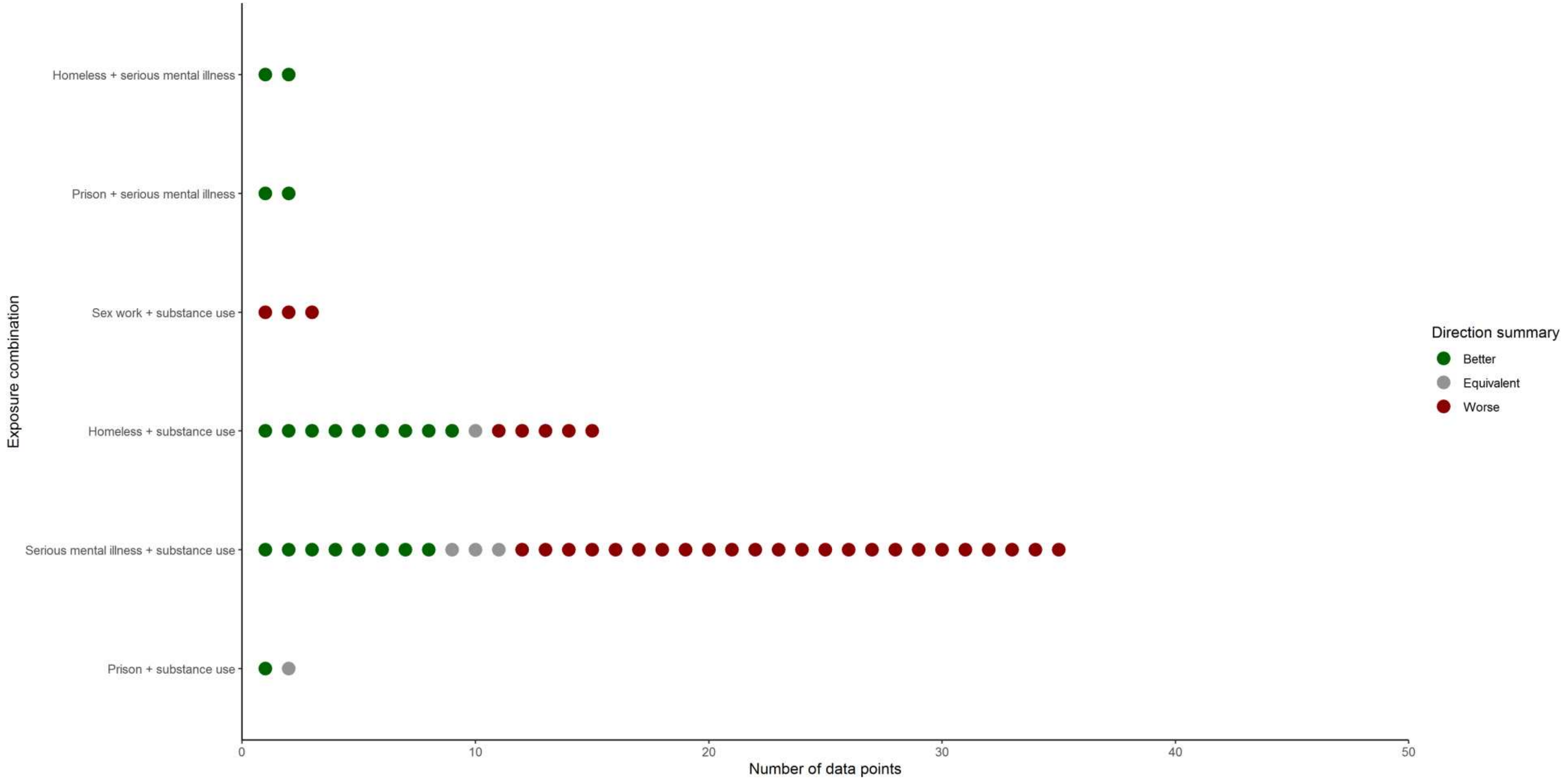

One-tailed sign test for effect direction (poorer outcomes among those with multiple vs fewer exposures): p=0.110

Appendix 4. Results – meta-analysis

Table 4.1. List of outcomes for which sufficient data were available to undertake meta-analysis, and whether feasible to stratify meta-analyses by risk of bias and gender.

ICD-10 chapter	Outcome	Outcome measure(s)	Stratification by risk of bias?	Stratification by gender?
1-20	All-cause mortality	Hazard ratio<br>Odds ratio<br>Rate ratio<br>Risk ratio	Y<br>Y<br>Y<br>N	Y<br>Y<br>Y<br>N
1	Hepatitis A	Lifetime prevalence	Y	N
1	Hepatitis B (anti-HBc positive)	Lifetime prevalence	Y	Y
1	Hepatitis C	Lifetime prevalence	Y	Y
1	Hepatitis E	Lifetime prevalence	N	N
1	HIV	Lifetime prevalence	Y	Y
1	Latent tuberculosis	Lifetime prevalence	N	N
1	HPV infection	Lifetime prevalence	N	N
1	Sexually transmitted infections	Lifetime prevalence<br>Current prevalence<br>Incident - hazard ratio<br>Incident - odds ratio<br>Incident – relative risk	N<br>N<br>N<br>N<br>Y	N<br>Y<br>N<br>N<br>N
**5**	Attention deficit hyperactivity disorder	Lifetime prevalence	N	N
**5**	Alcohol disorders	Lifetime prevalence<br>Past year prevalence	N<br>N	N<br>Y
**5**	Personality disorders	Lifetime prevalence	N	Y
**5**	Anxiety disorders	Lifetime prevalence<br>Past year prevalence	Y<br>N	N<br>N
**5**	Depressive disorders	Lifetime prevalence<br>Past year prevalence<br>Past week prevalence<br>Current prevalence	N<br>N<br>N<br>N	N<br>Y<br>N<br>N
**5**	Dysthymia	Lifetime prevalence	N	N
**5**	Obsessive compulsive disorder	Lifetime prevalence	Y	N
**5**	Post-traumatic stress disorder	Lifetime prevalence<br>Current prevalence	N<br>N	N<br>N
**5**	Pathological gambling	Lifetime prevalence	Y	N
**12**	Skin and soft tissue infections	Past year prevalence<br>Past six months prevalence<br>Incident – odds ratios	N<br>N<br>N	N<br>N<br>N
**14**	Amenorrhoea	Current prevalence	N	N
**14**	Irregular menstrual cycle	Current prevalence	N	N
**19**	Non-fatal drug overdose	Lifetime prevalence<br>Past 18 month prevalence<br>Past 12 month prevalence<br>Past 6 month prevalence<br>Past month prevalence<br>Incident – hazard ratio	Y<br>N<br>Y<br>Y<br>N<br>N	N<br>N<br>N<br>N<br>N<br>N
**19**	Drug overdose mortality	Hazard ratio<br>Odds ratio<br>Relative risk	N<br>Y<br>N	N<br>N<br>N
**19**	Head injury	Lifetime prevalence	N	N
**19**	Brain injury	Lifetime prevalence	Y	N
**19**	Injury mortality	Hazard ratio	N	Y
**20**	Deliberate self-harm	Lifetime prevalence<br>Past month prevalence	Y<br>Y	Y<br>Y
**20**	Accident mortality	Subhazard ratio	N	N
**20**	Suicide mortality	Hazard ratio<br>Odds ratio<br>Incidence rate ratio	N<br>Y<br>N	Y<br>N<br>N
**19 & 20**	External causes of mortality combined	Hazard ratios	Y	Y
	Non-communicable diseases	Lifetime prevalence<br>Past year prevalence<br>Current prevalence	Y<br>N<br>Y	N<br>Y<br>N
	Non-communicable disease mortality	Hazard ratio<br>Cumulative mortality<br>Rate ratio	N<br>N<br>N	N<br>N<br>Y

Figure A4.1. Forest plot for meta-analysis of studies reporting hazard ratios for all-cause mortality among people with multiple versus fewer exposures, by exposure combination – also shown in main text

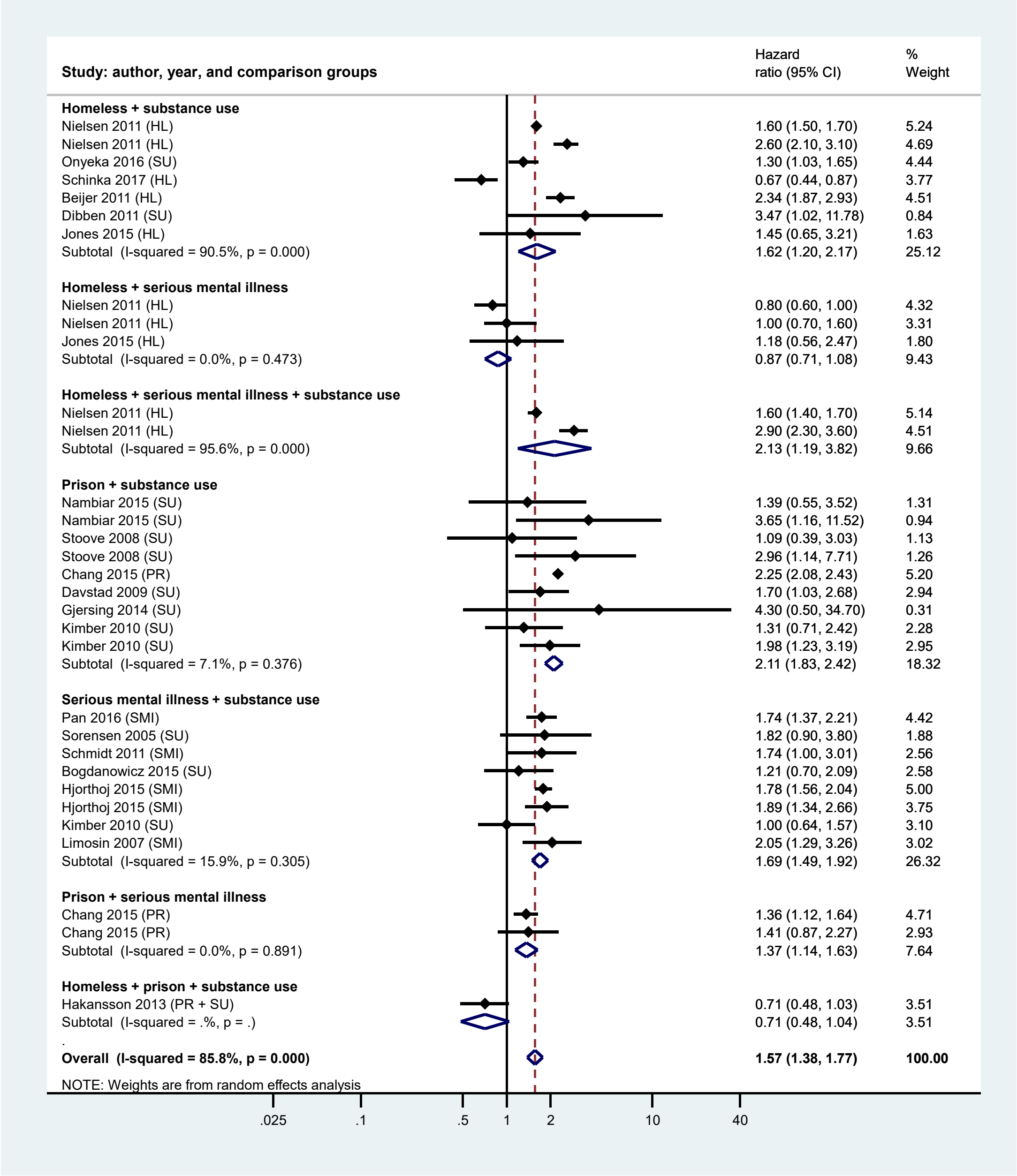

Figure A4.2. Forest plot for meta-analysis of studies reporting hazard ratios for all-cause mortality among people with multiple versus fewer exposures, by risk of bias

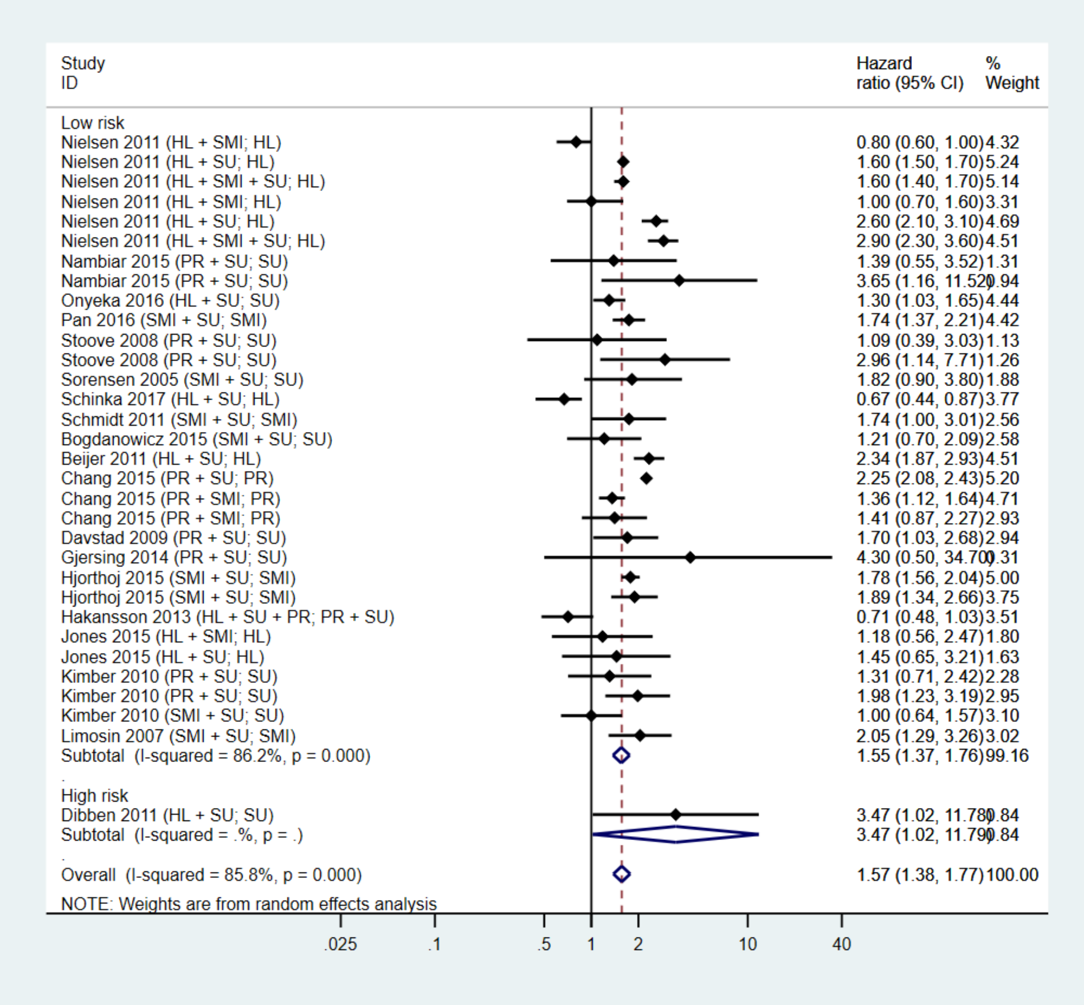

Figure A4.3. Forest plot for meta-analysis of studies reporting hazard ratios for all-cause mortality among people with multiple versus fewer exposures, by gender (where reported separately)

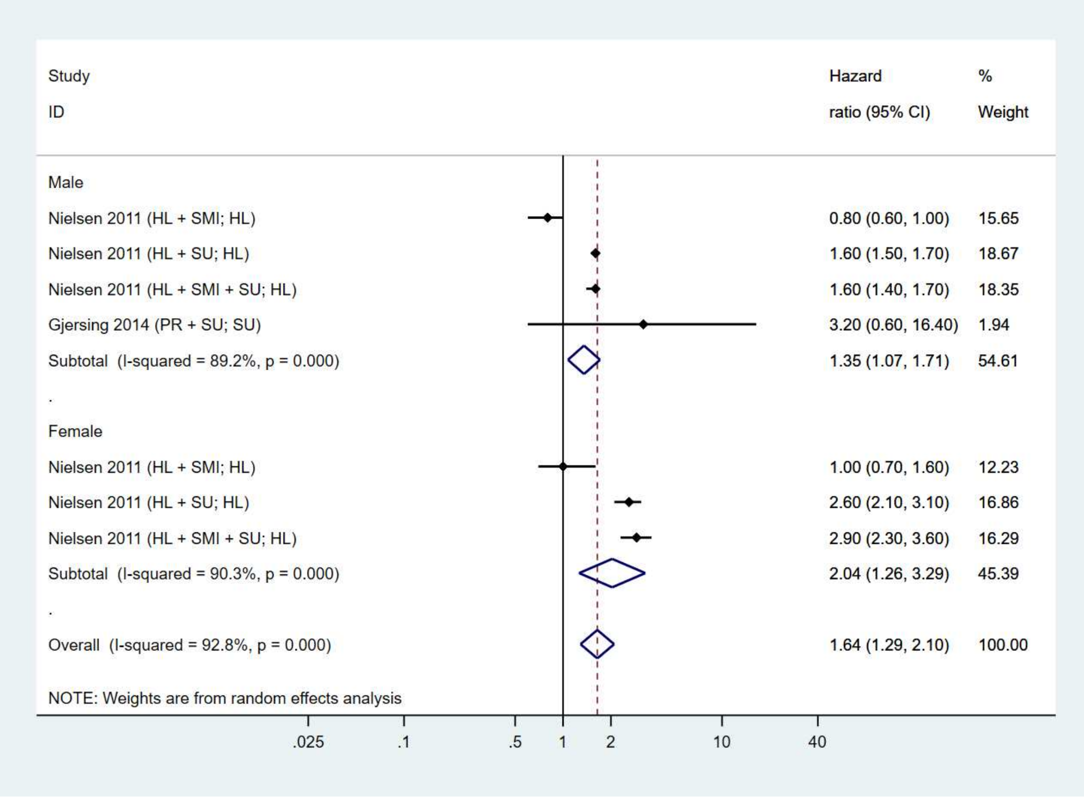

Figure A4.4. Forest plot for meta-analysis of studies reporting odds ratios for all-cause mortality among people with multiple versus fewer exposures, by exposure combination

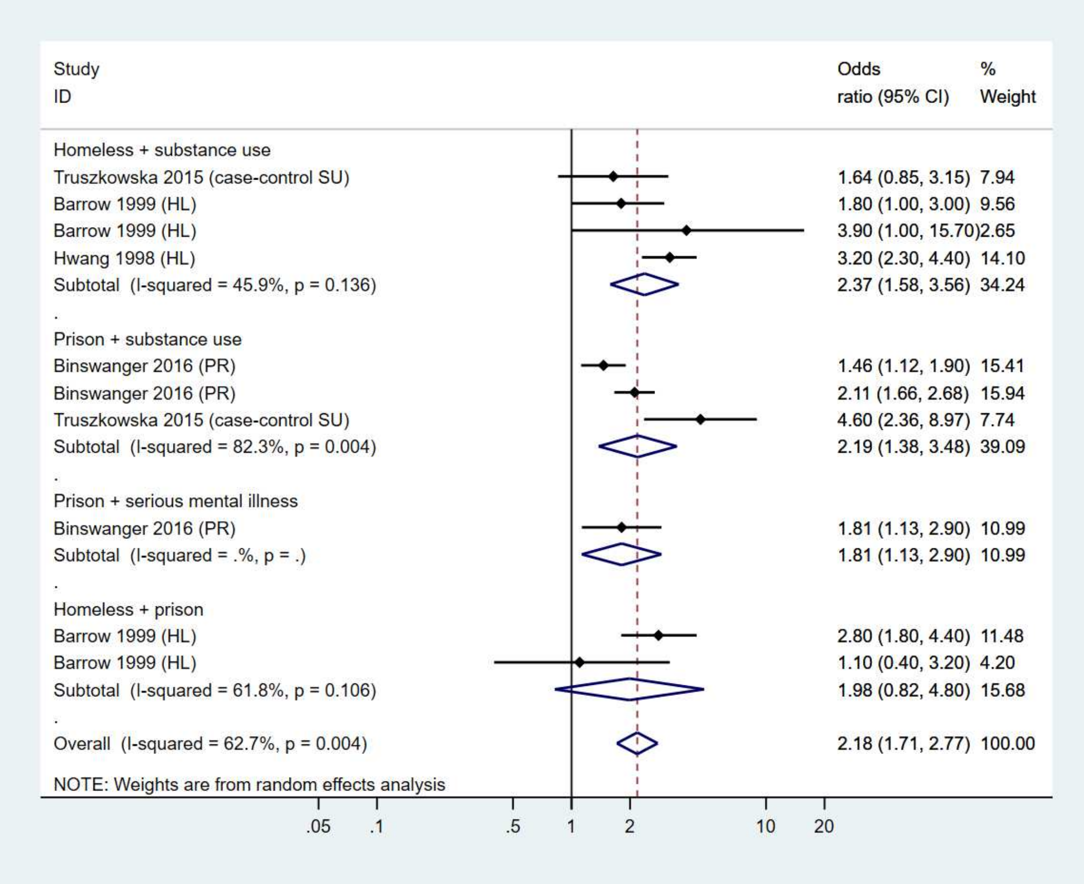

Figure A4.5. Forest plot for meta-analysis of studies reporting odds ratios for all-cause mortality among people with multiple versus fewer exposures, by risk of bias

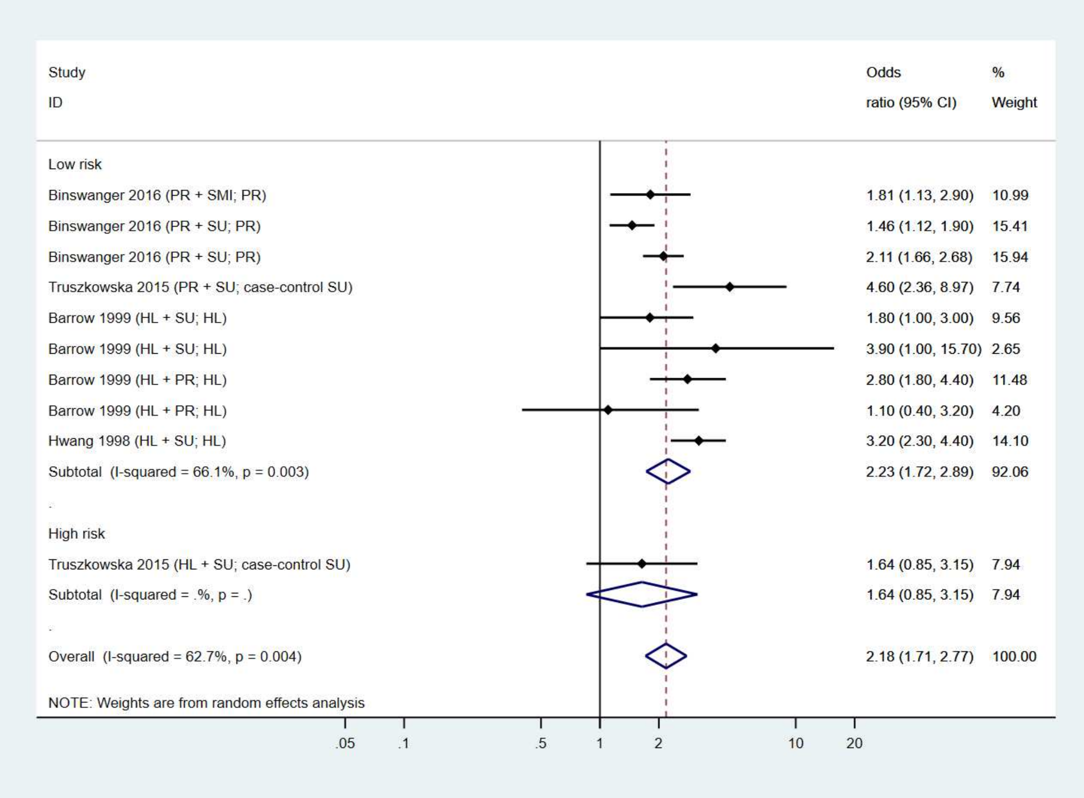

Figure A4.6. Forest plot for meta-analysis of studies reporting odds ratios for all-cause mortality among people with multiple versus fewer exposures, by gender (where reported separately)

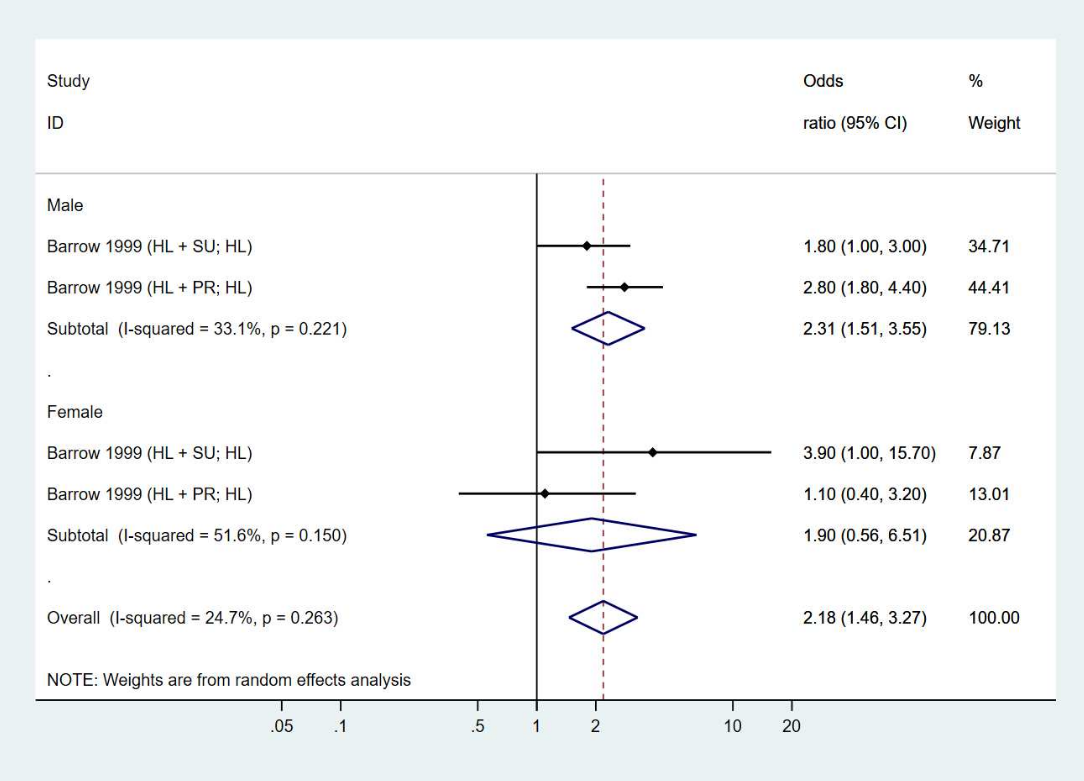

Figure A4.7. Forest plot for meta-analysis of studies reporting rate ratios for all-cause mortality among people with multiple versus fewer exposures, by exposure combination

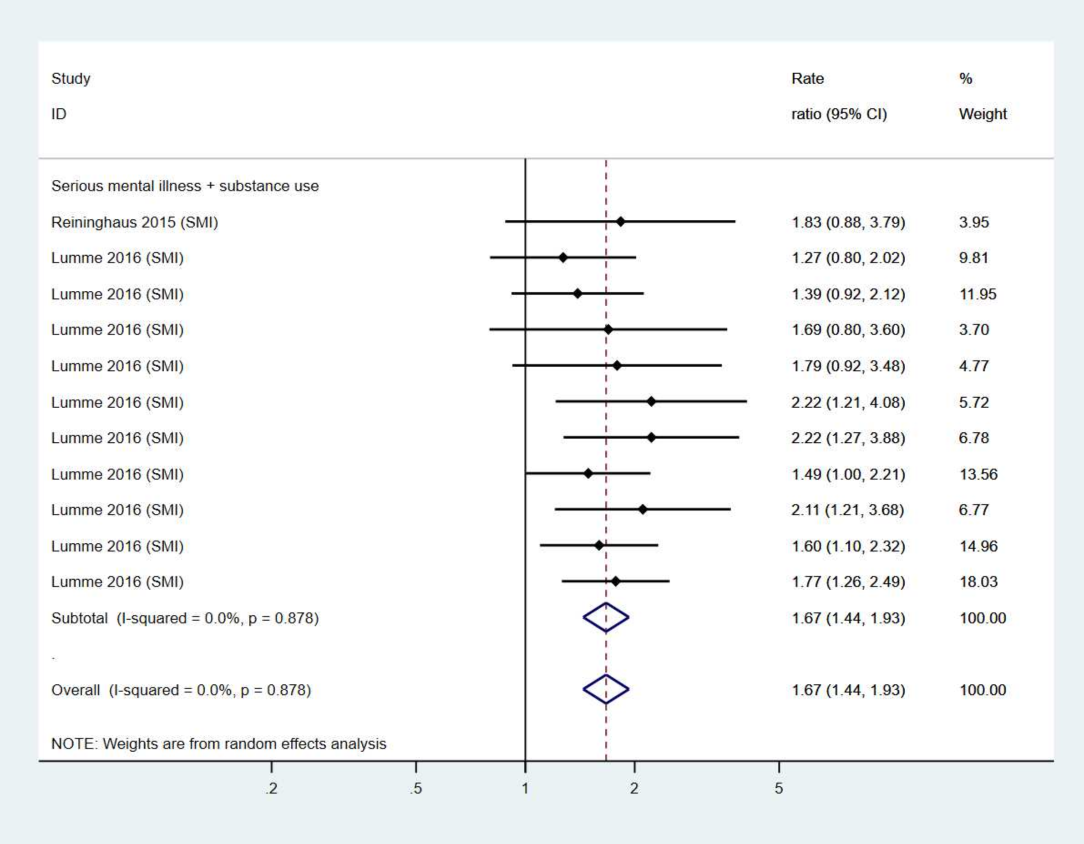

Figure A4.8. Forest plot for meta-analysis of studies reporting rate ratios for all-cause mortality among people with multiple versus fewer exposures, by risk of bias

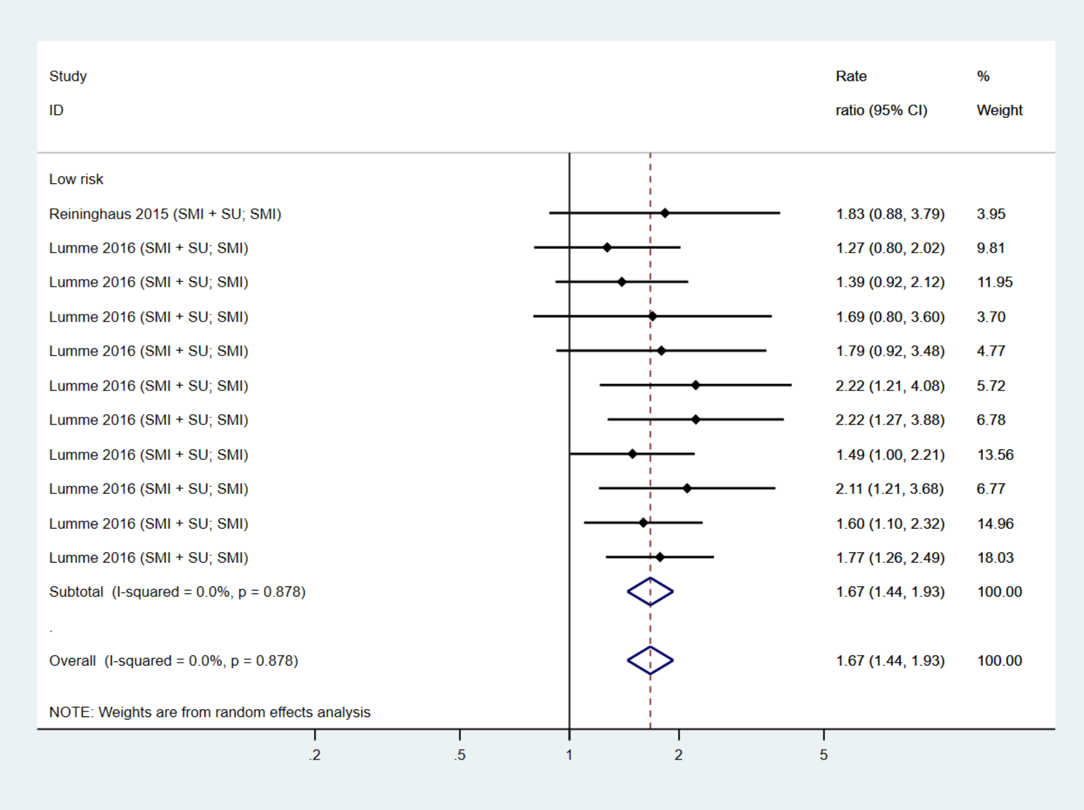

Figure A4.9. Forest plot for meta-analysis of studies reporting rate ratios for all-cause mortality among people with multiple versus fewer exposures, by gender (where reported separately)

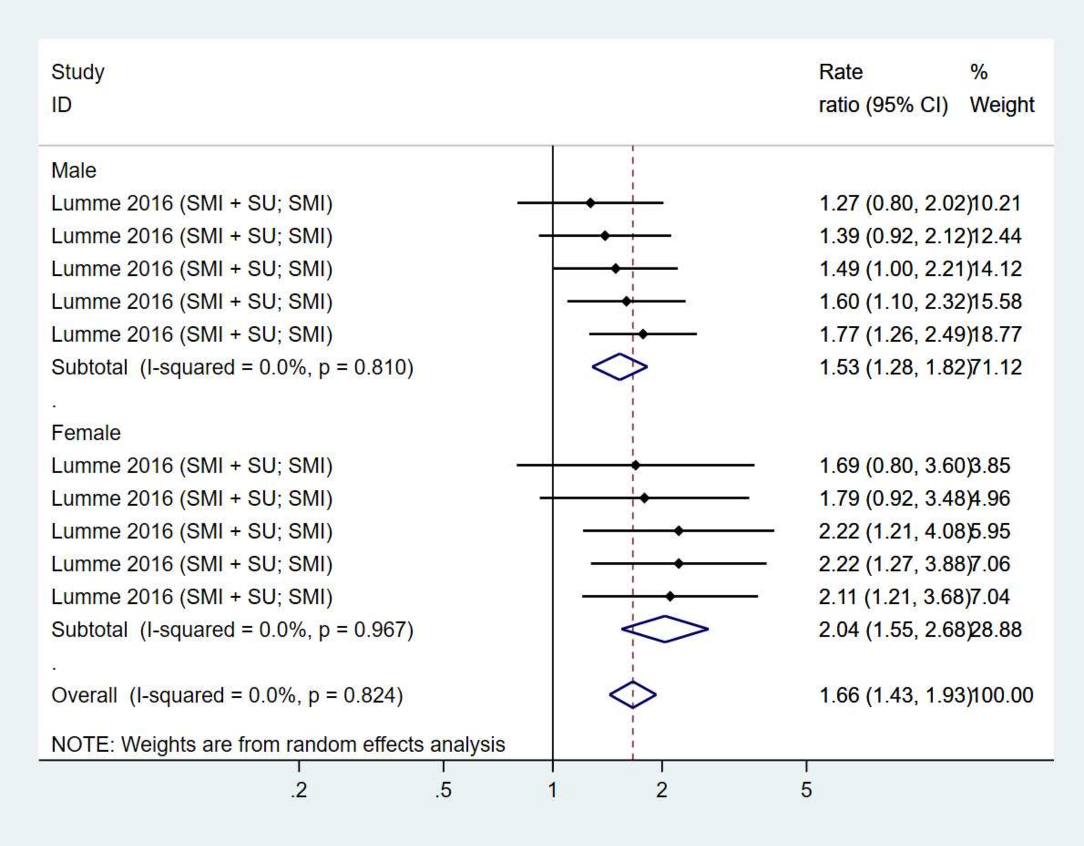

Figure A4.10. Forest plot for meta-analysis of studies reporting risk ratios for all-cause mortality among people with multiple versus fewer exposures, by exposure combination

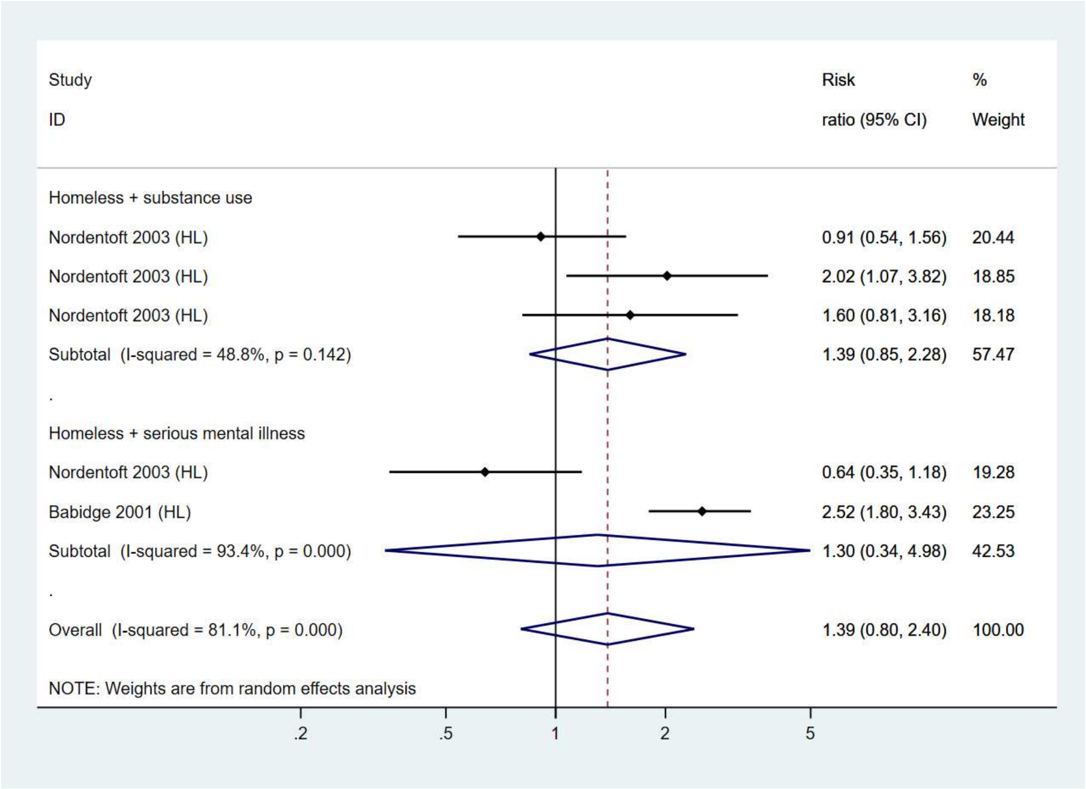

Insufficient data available to permit stratification by gender or risk of bias.

Figure A4.11. Forest plot for meta-analysis of studies reporting lifetime prevalence of hepatitis A infection among people with multiple versus fewer exposures, by exposure combination

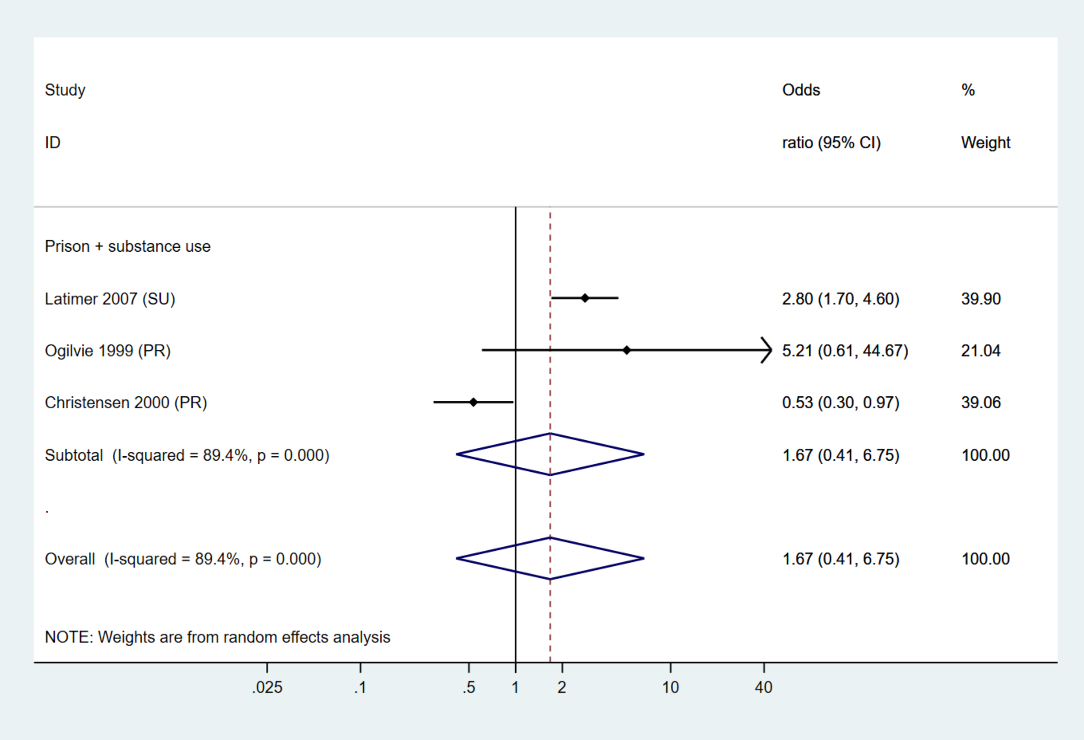

Figure A4.12. Forest plot for meta-analysis of studies reporting lifetime prevalence of hepatitis A infection among people with multiple versus fewer exposures, by risk of bias

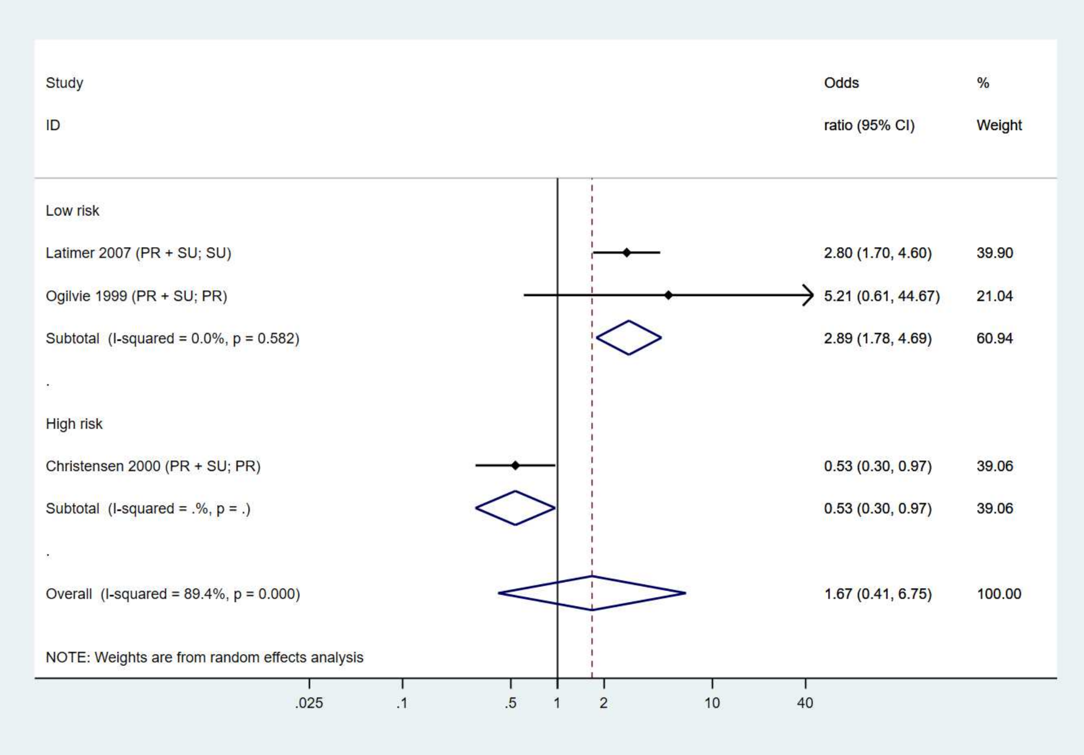

Insufficient data available to permit stratification by gender.

Figure A4.13. Forest plot for meta-analysis of studies reporting lifetime prevalence of hepatitis B virus infection (anti-HBc positive) among people with multiple versus fewer exposures, by exposure combination

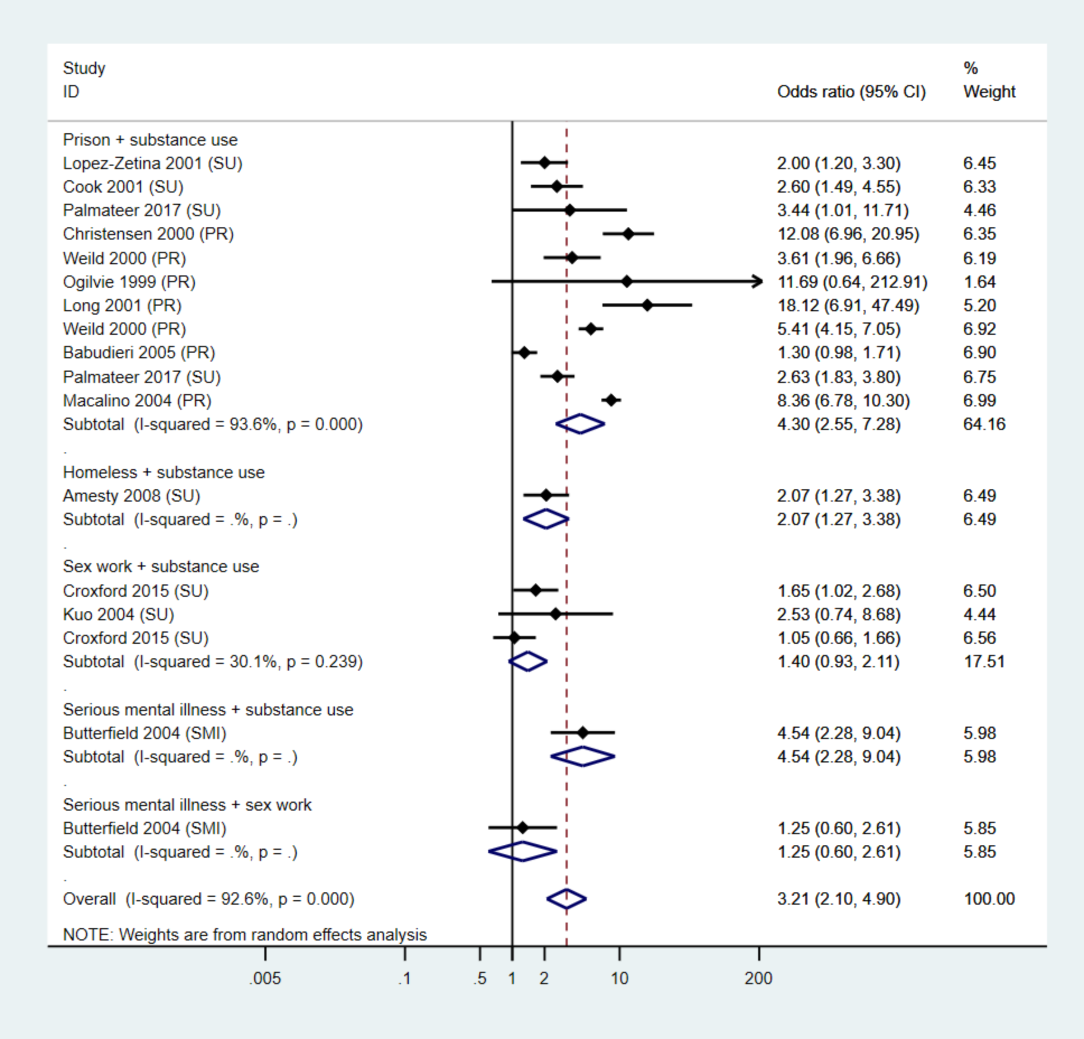

Figure A4.14. Forest plot for meta-analysis of studies reporting lifetime prevalence of hepatitis B virus infection (anti-HBc positive) among people with multiple versus fewer exposures, by risk of bias

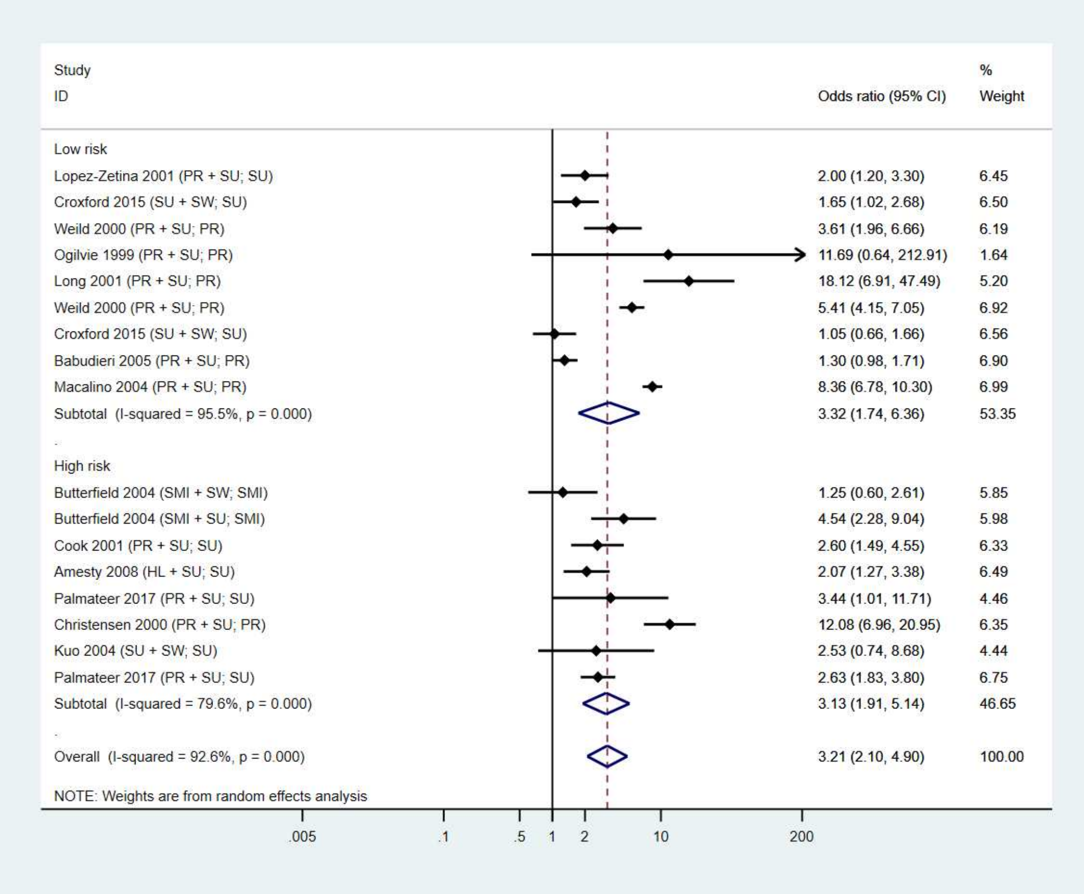

Figure A4.15. Forest plot for meta-analysis of studies reporting lifetime prevalence of hepatitis B virus infection (anti-HBc positive) among people with multiple versus fewer exposures, by gender

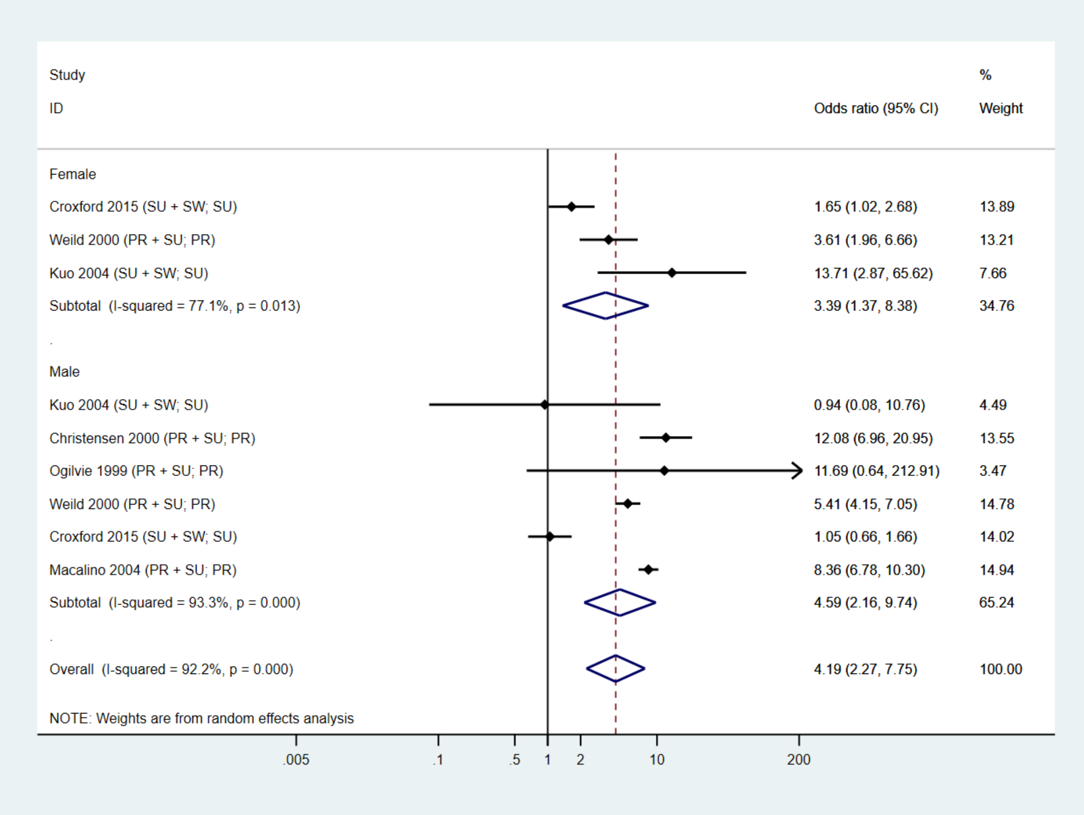

Figure A4.16. Forest plot for meta-analysis of studies reporting lifetime prevalence of hepatitis C virus infection among people with multiple versus fewer exposures, by exposure combination

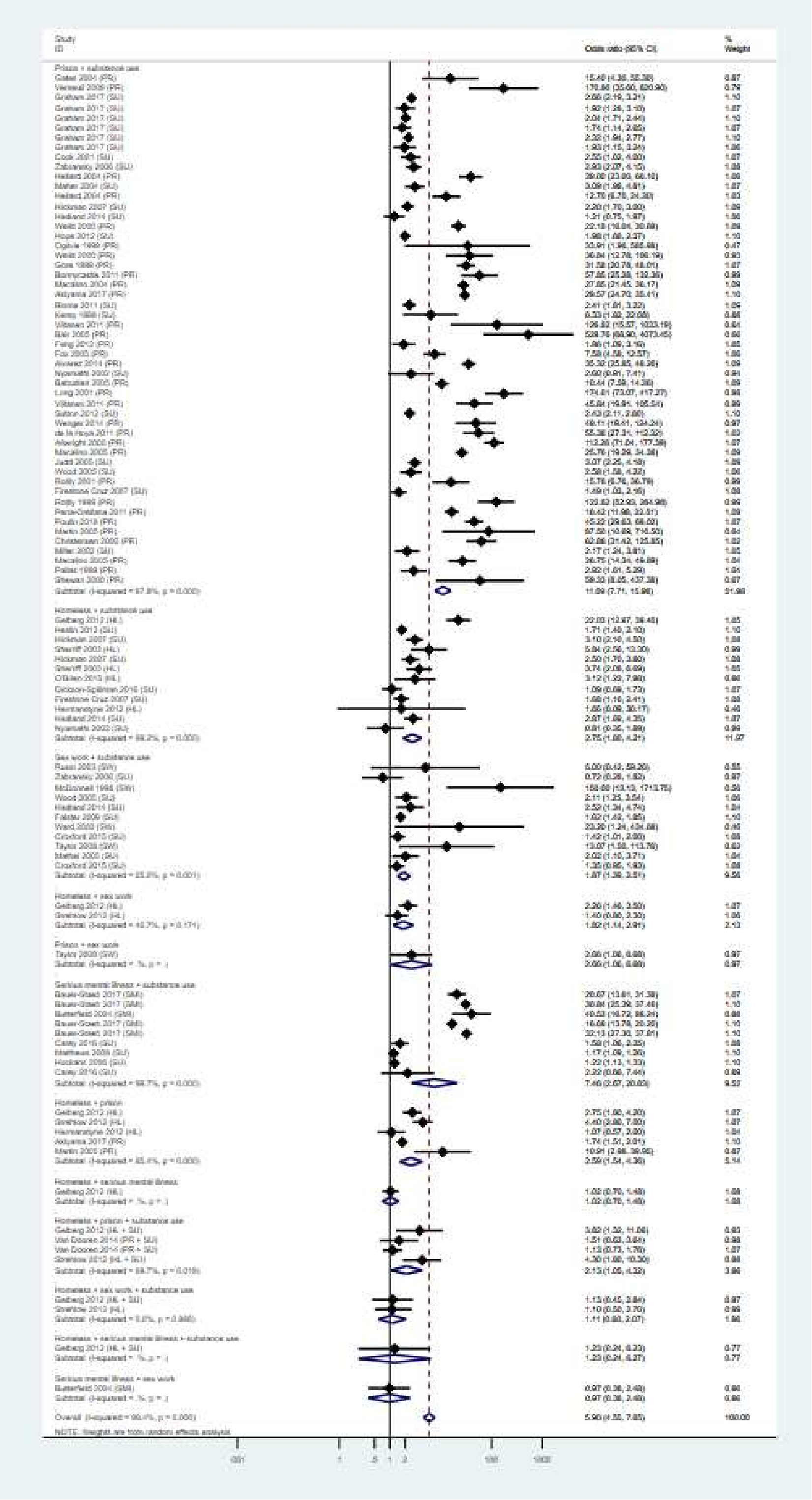

Figure A4.17. Forest plot for meta-analysis of studies reporting lifetime prevalence of hepatitis C virus infection among people with multiple versus fewer exposures, by risk of bias

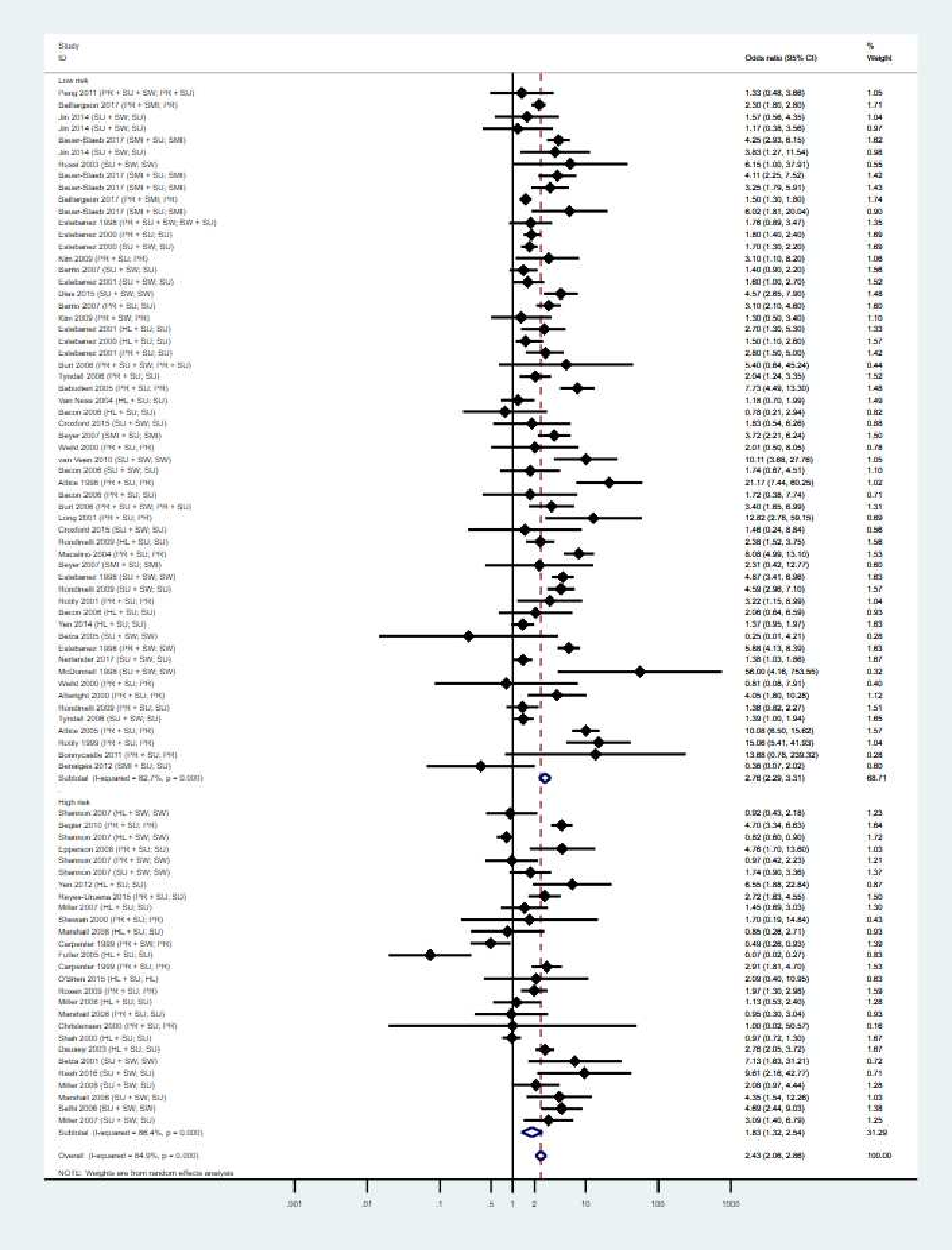

Figure A4.18. Forest plot for meta-analysis of studies reporting lifetime prevalence of hepatitis C virus infection among people with multiple versus fewer exposures, by gender

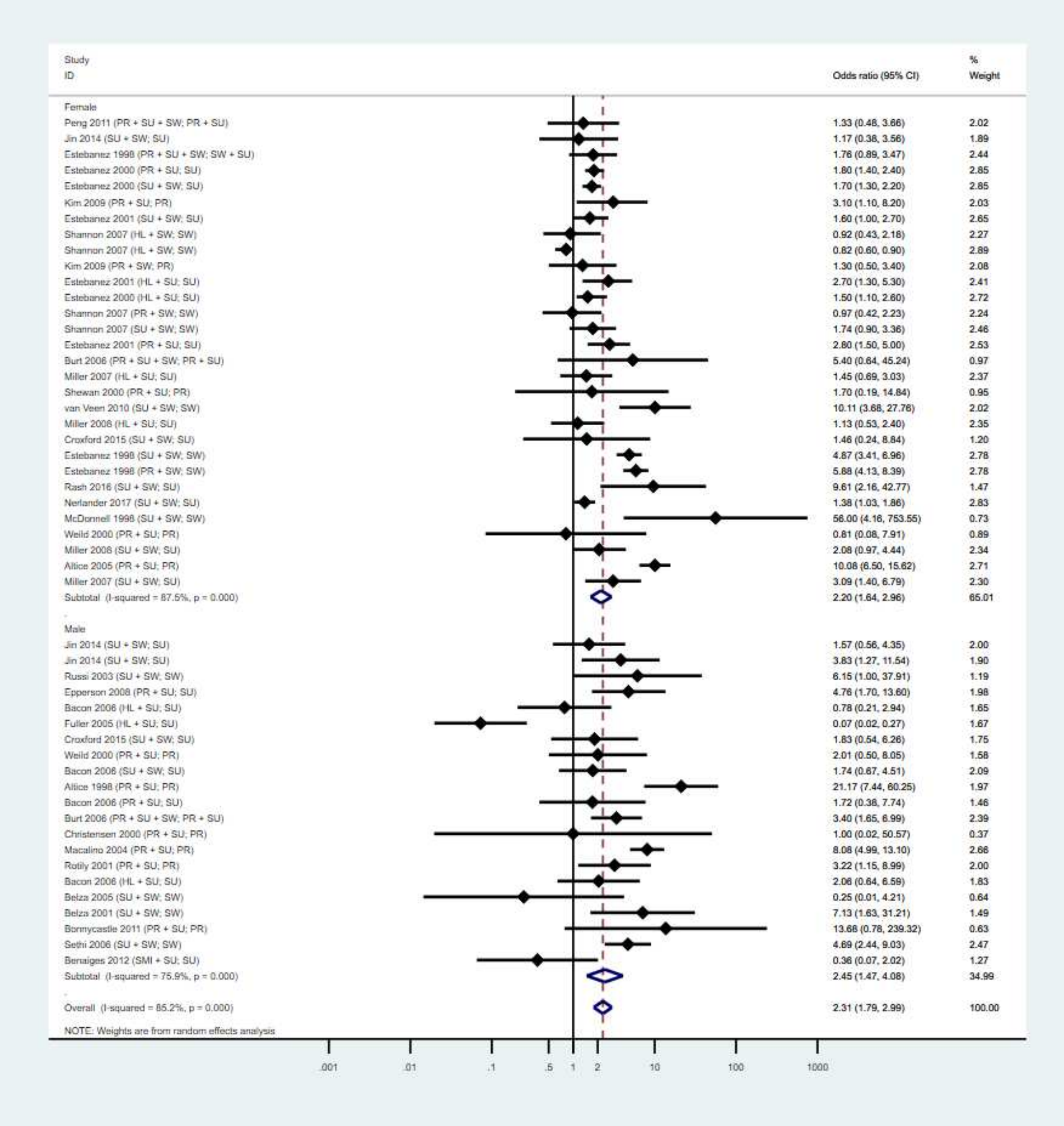

Figure A4.19. Forest plot for meta-analysis of studies reporting lifetime prevalence of hepatitis E infection among people with multiple versus fewer exposures, by exposure combination

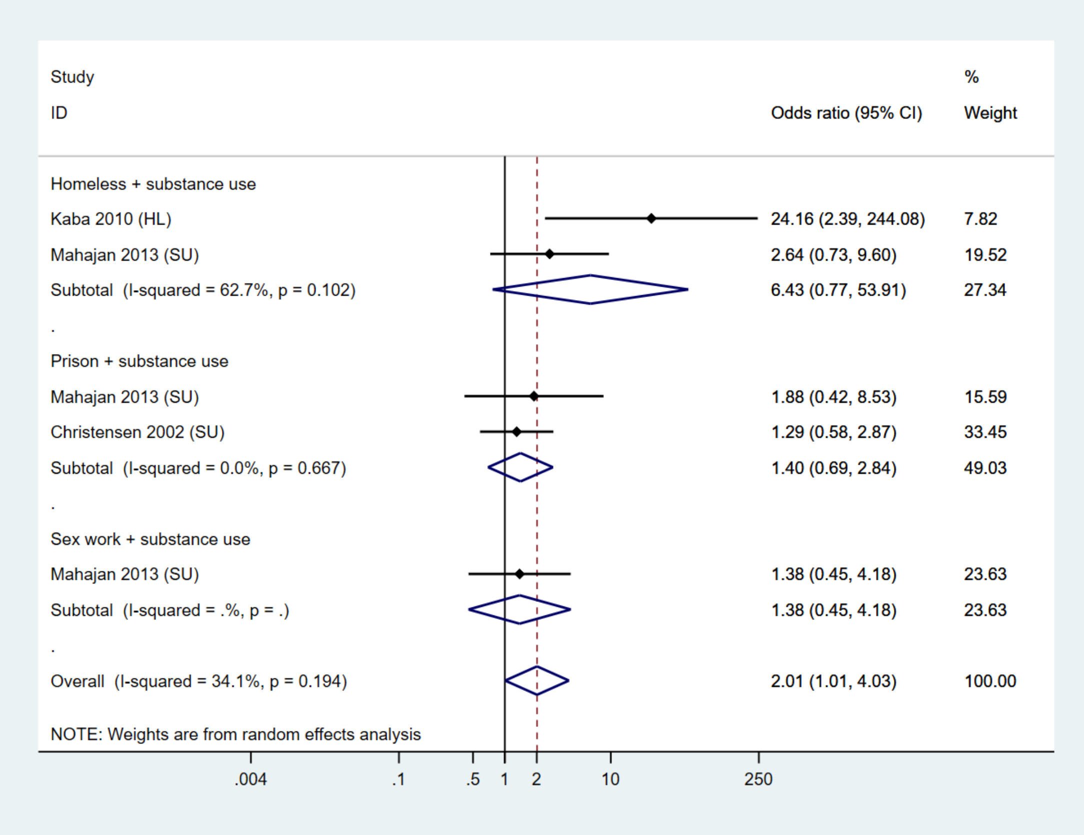

Insufficient data available to permit stratification by risk of bias or gender.

Figure A4.20. Forest plot for meta-analysis of studies reporting lifetime prevalence of HIV infection among people with multiple versus fewer exposures, by exposure combination

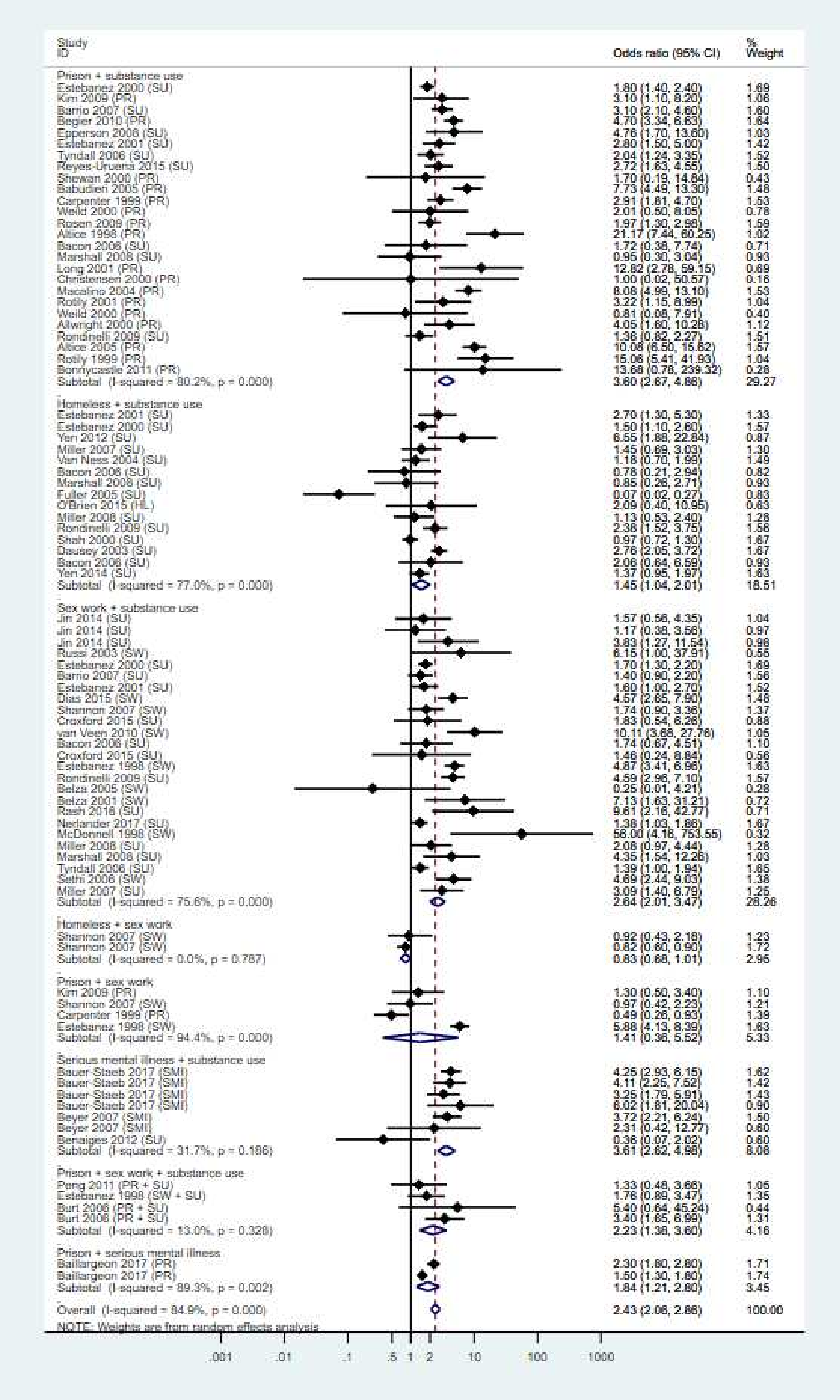

Figure A4.21. Forest plot for meta-analysis of studies reporting lifetime prevalence of HIV infection among people with multiple versus fewer exposures, by risk of bias

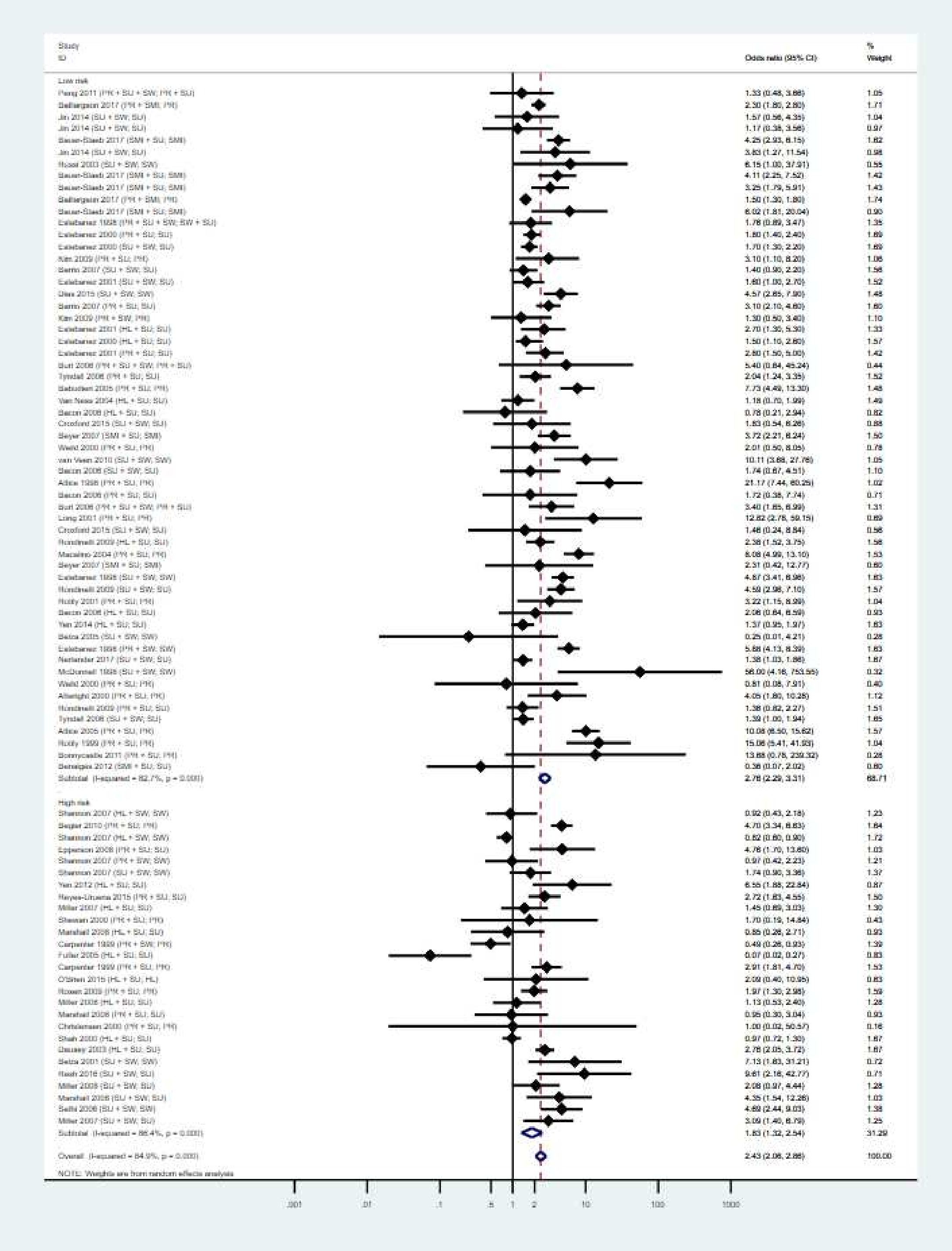

Figure A4.25. Forest plot for meta-analysis of studies reporting lifetime prevalence of HIV infection among people with multiple versus fewer exposures, by gender

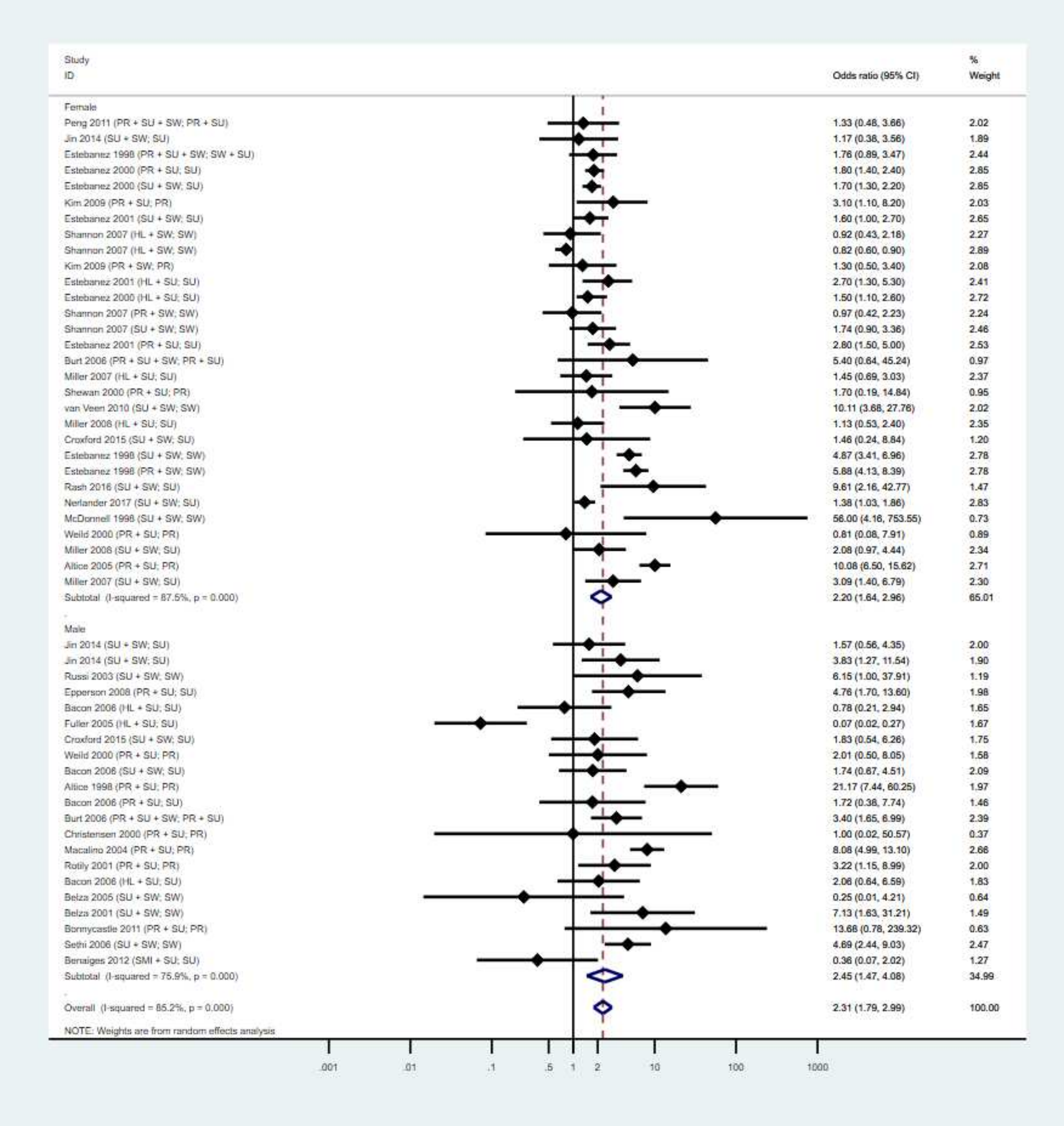

Figure A4.16. Forest plot for meta-analysis of studies reporting lifetime prevalence of latent tuberculosis infection among people with multiple versus fewer exposures, by exposure combination

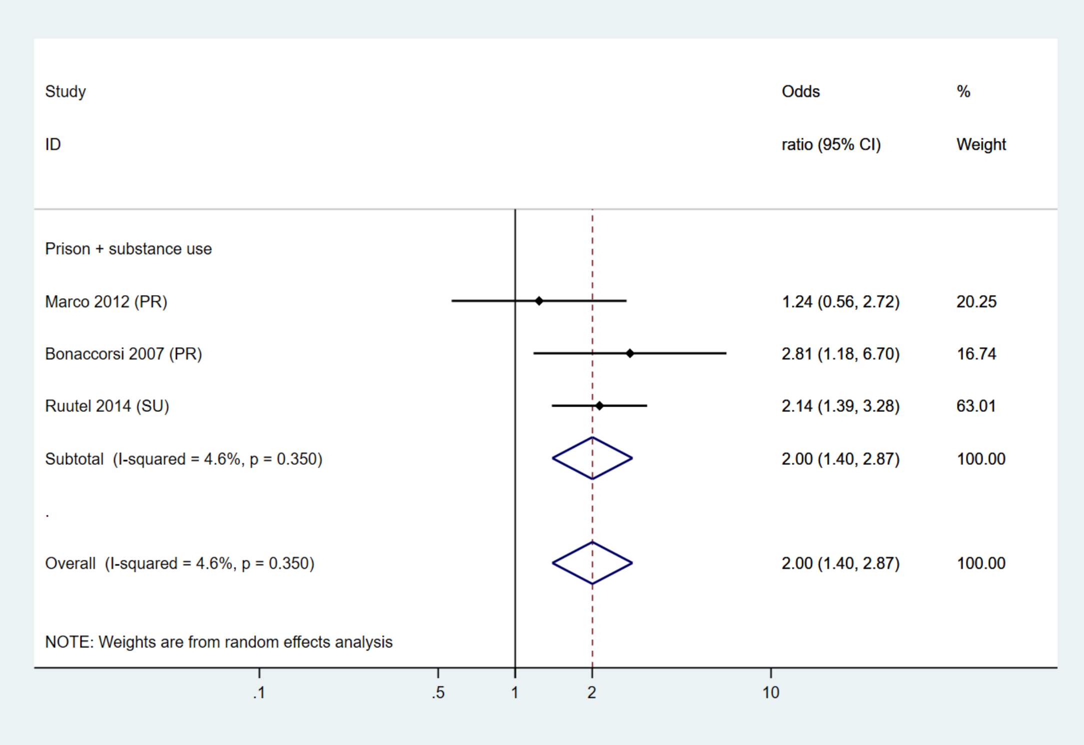

Insufficient data available to permit stratification by risk of bias or gender.

Figure A4.17. Forest plot for meta-analysis of studies reporting current prevalence of human papillomavirus (HPV) infection among people with multiple versus fewer exposures, by exposure combination

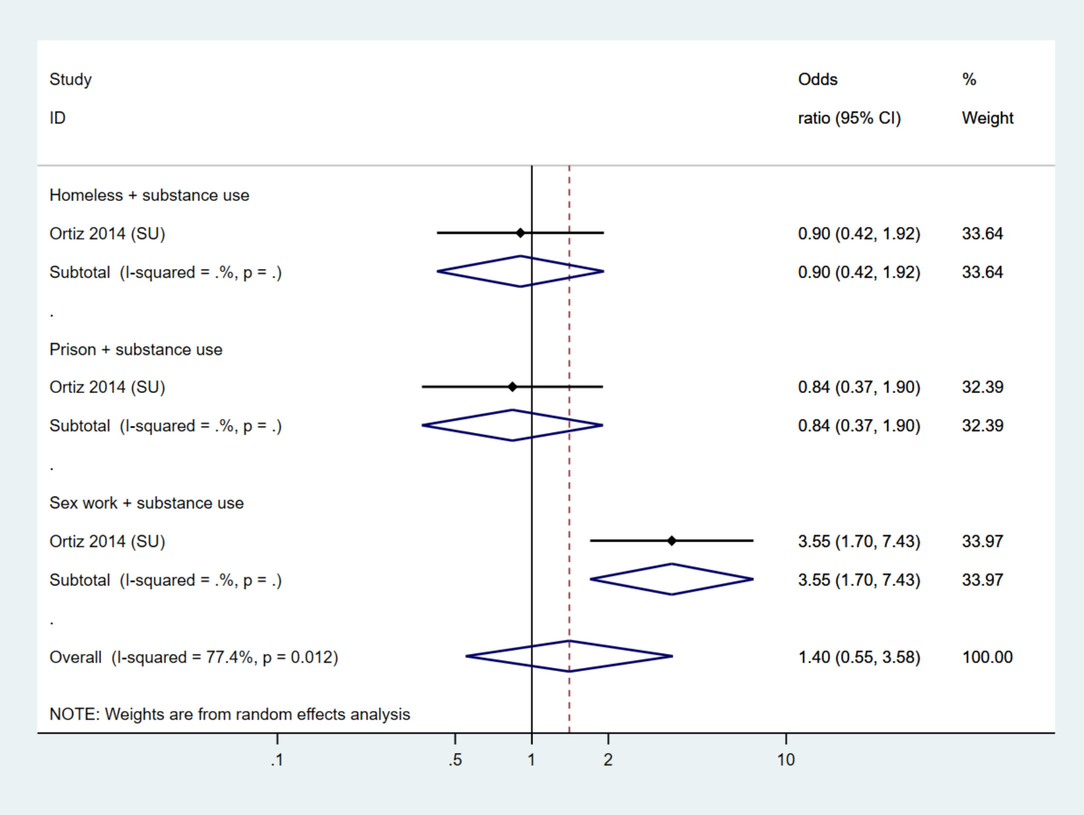

Insufficient data available to permit stratification by risk of bias or gender.

Figure A4.18. Forest plot for meta-analysis of studies reporting lifetime prevalence of sexually transmitted infection among people with multiple versus fewer exposures, by exposure combination

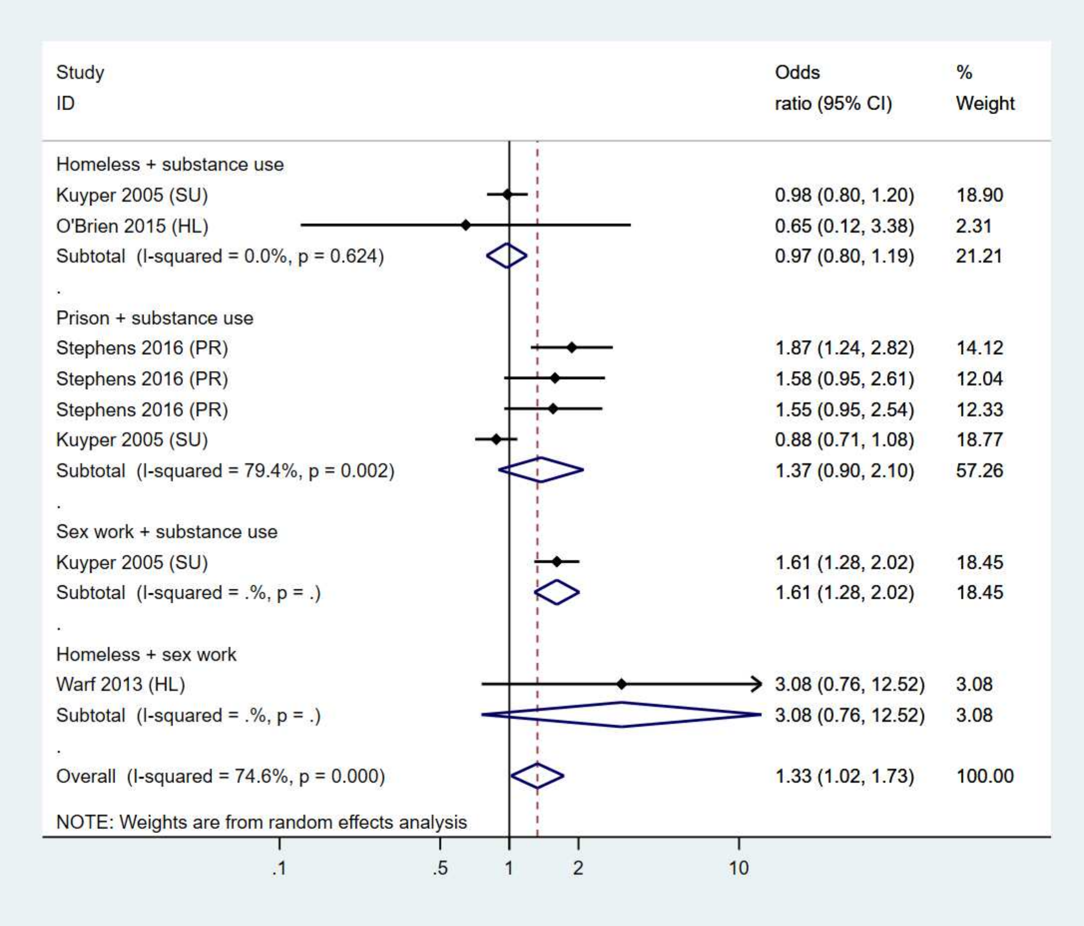

Insufficient data available to permit stratification by risk of bias or gender.

Figure A4.19. Forest plot for meta-analysis of studies reporting prevalence of current sexually transmitted infections among people with multiple versus fewer exposures, by exposure combination

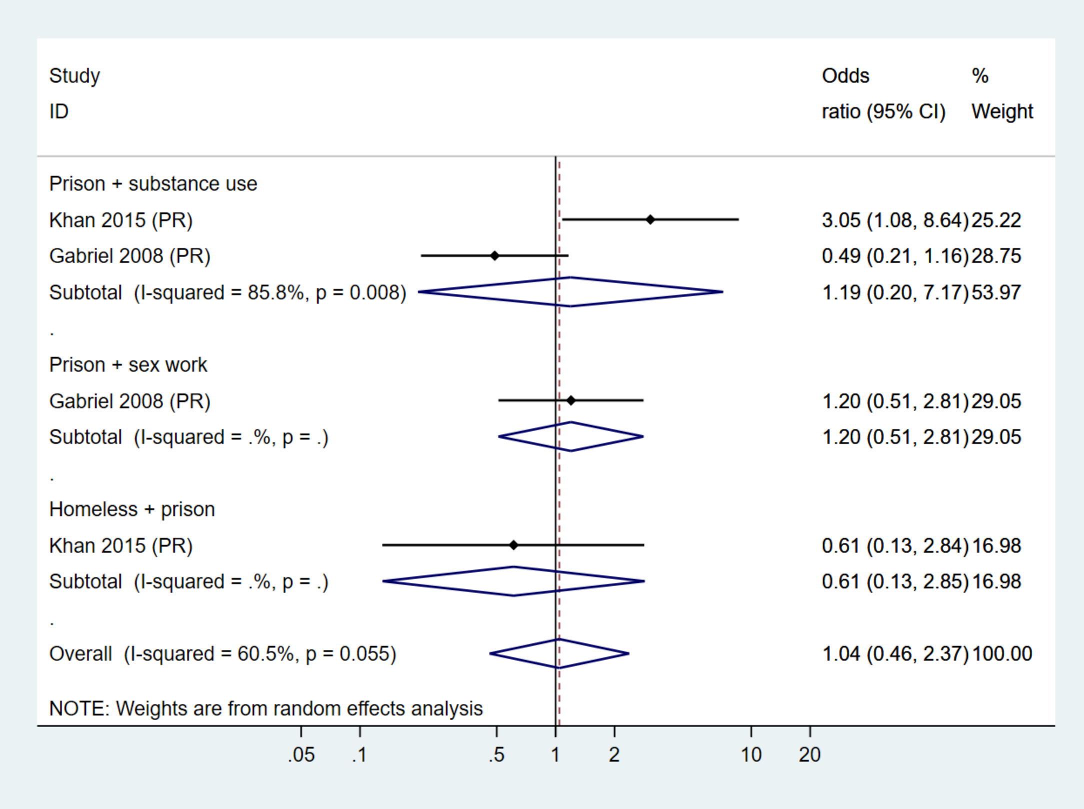

Figure A4.20. Forest plot for meta-analysis of studies reporting prevalence of current sexually transmitted infections among people with multiple versus fewer exposures, by gender (where data reported separately)

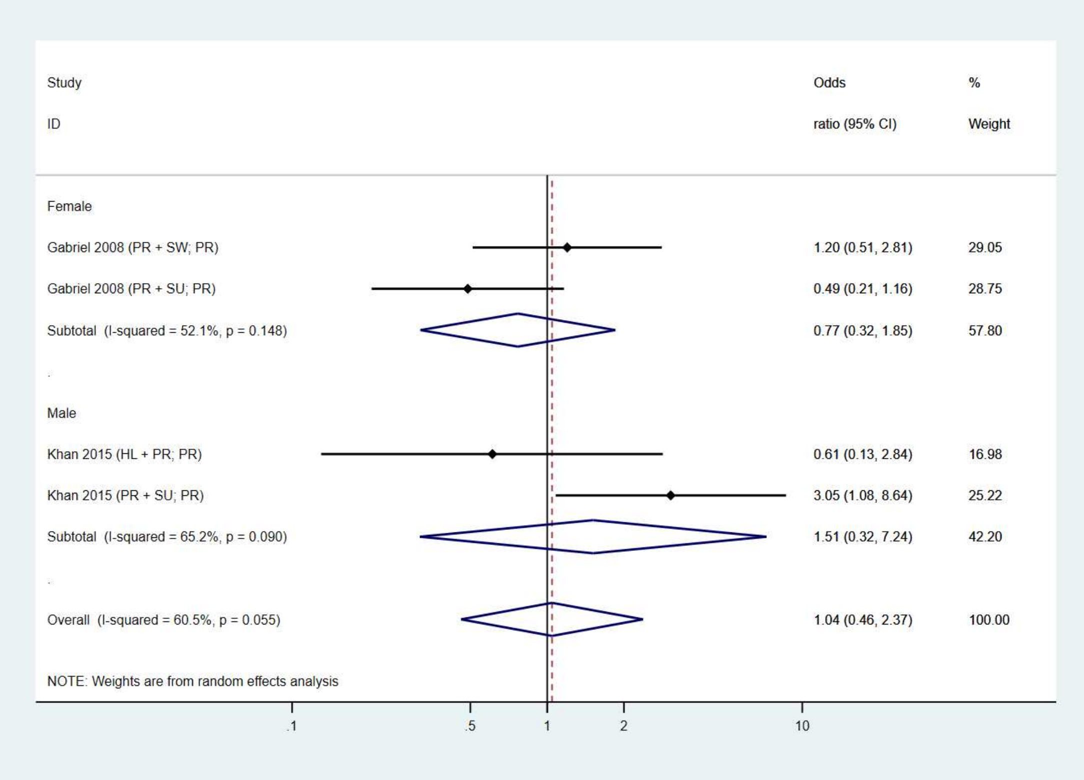

Insufficient data available to permit stratification by risk of bias.

Figure A4.21. Forest plot for meta-analysis of studies reporting hazard ratios for sexually transmitted infections among people with multiple versus fewer exposures, by exposure combination

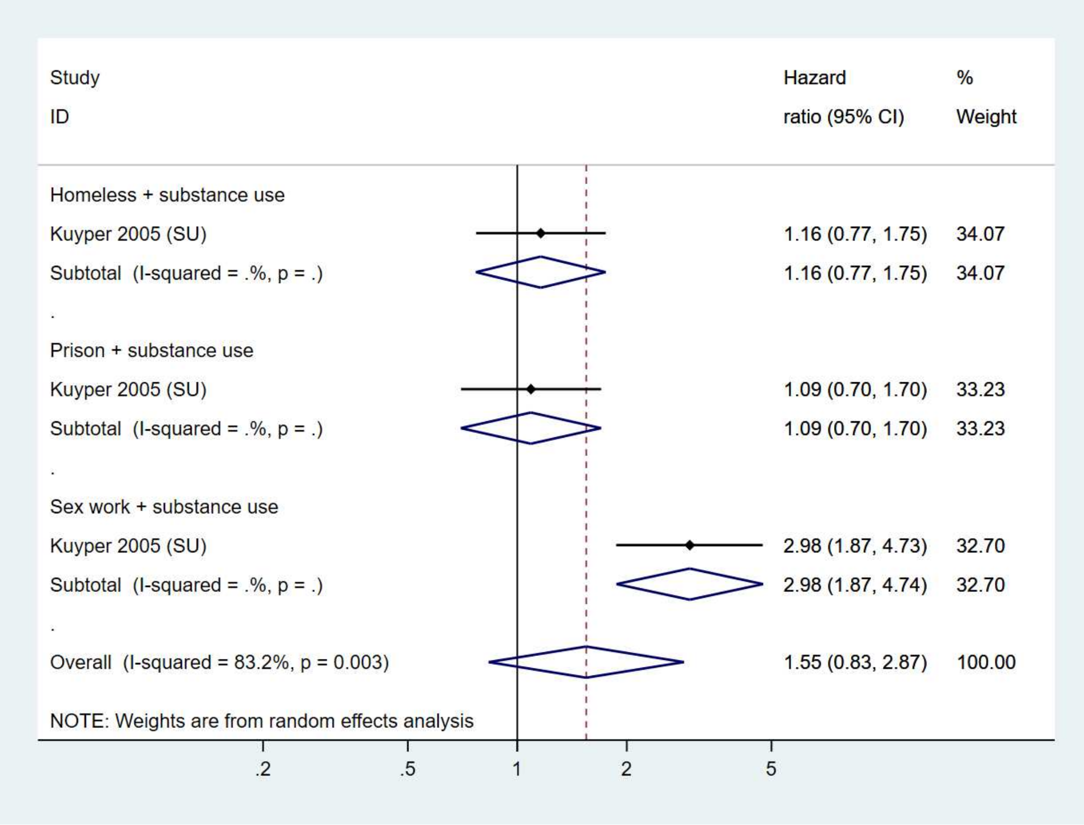

Insufficient data available to permit stratification by risk of bias or gender.

Figure A4.22. Forest plot for meta-analysis of studies reporting relative risk for the incidence of sexually transmitted infections among people with multiple versus fewer exposures, by exposure combination

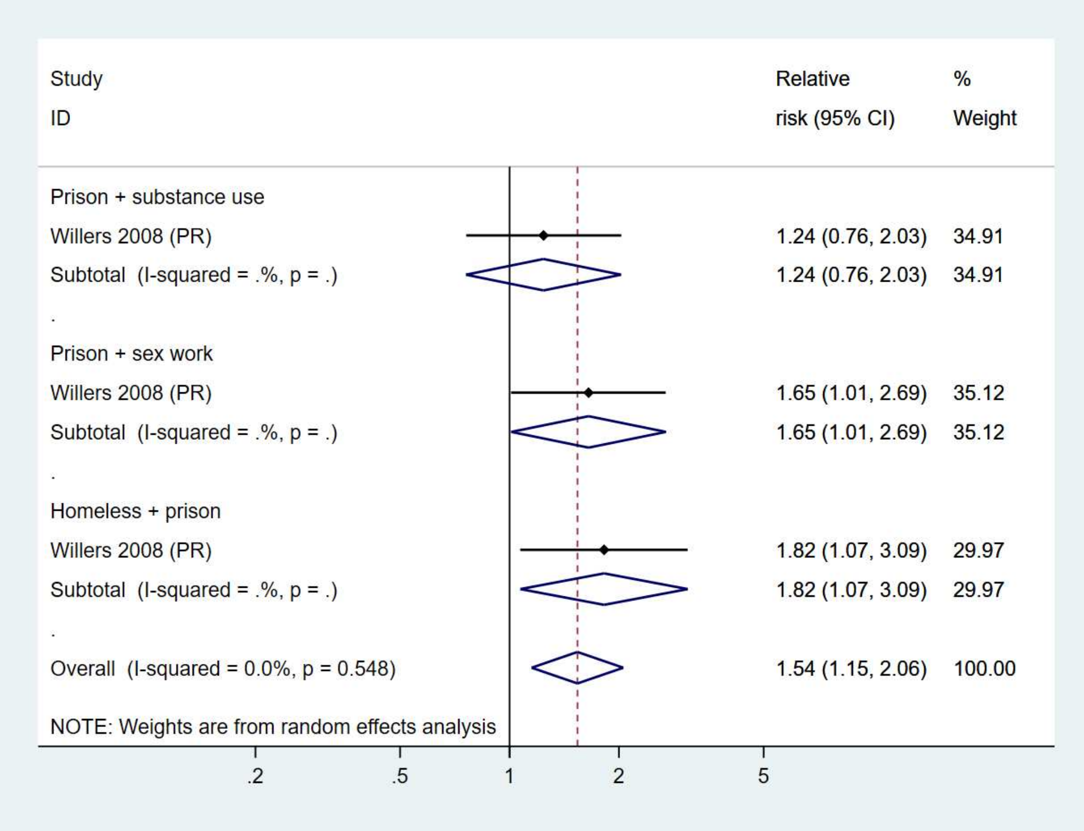

Figure A4.23. Forest plot for meta-analysis of studies reporting relative risk for the incidence of sexually transmitted infections among people with multiple versus fewer exposures, by risk of bias

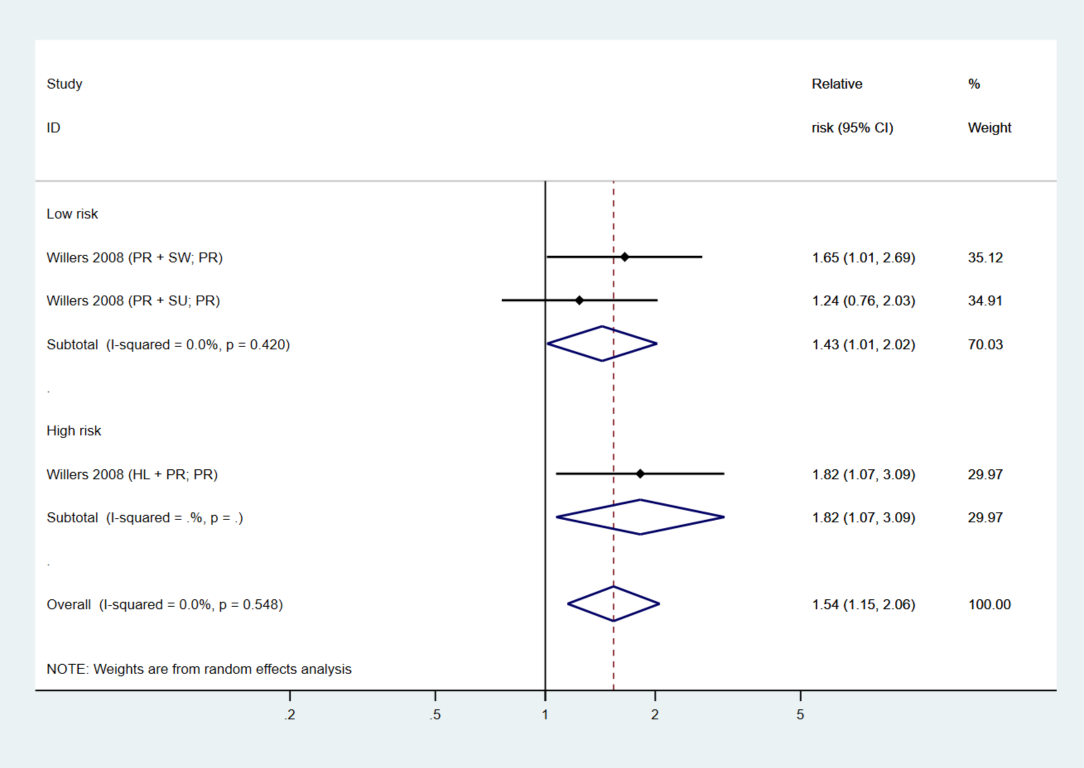

Insufficient data available to permit stratification by gender.

Figure A4.24. Forest plot for meta-analysis of studies reporting lifetime prevalence of attention deficit hyperactivity disorder among people with multiple versus fewer exposures, by exposure combination

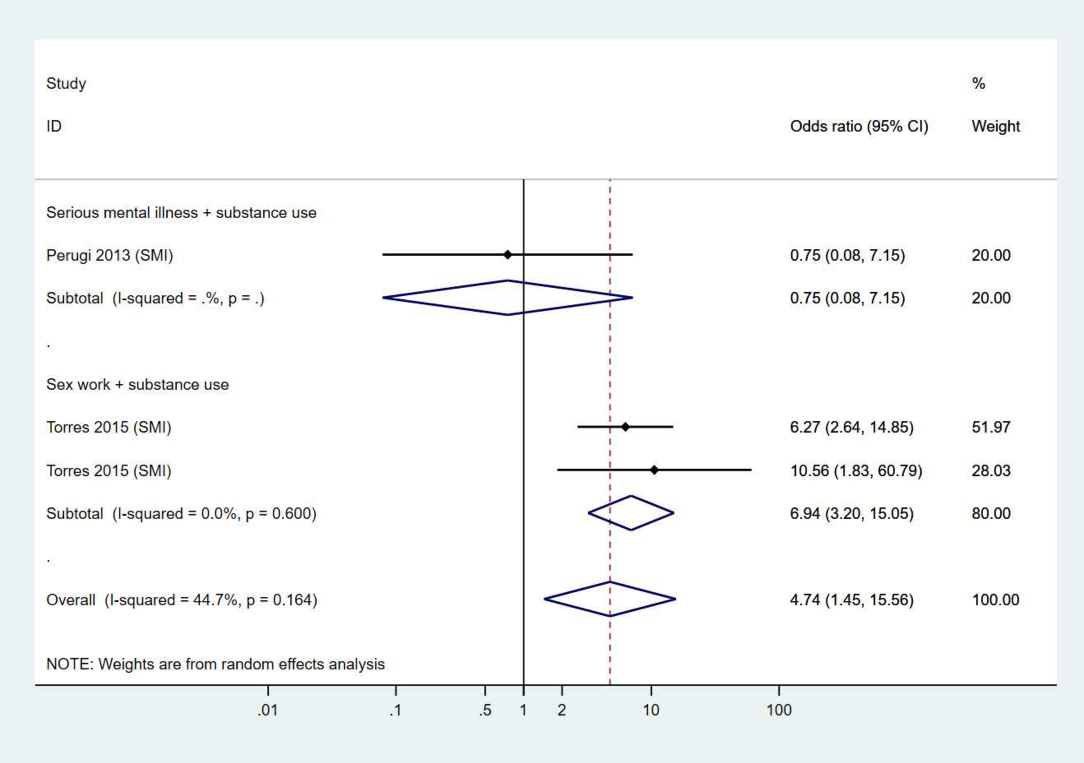

Insufficient data available to permit stratification by risk of bias or gender.

Figure A4.25. Forest plot for meta-analysis of studies reporting lifetime prevalence of mental and behavioural disorders due to use of alcohol among people with multiple versus fewer exposures, by exposure combination

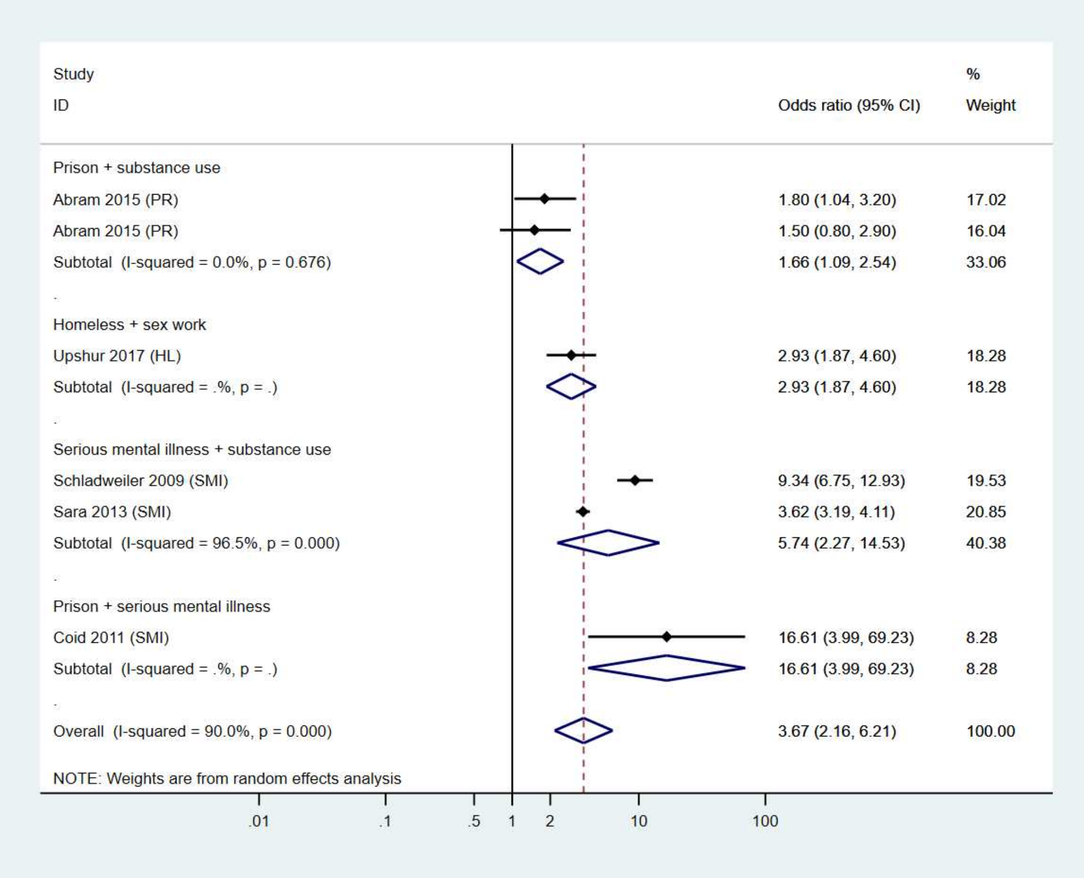

Insufficient data available to permit stratification by risk of bias or gender.

Figure A4.26. Forest plot for meta-analysis of studies reporting past year prevalence of mental and behavioural disorders due to use of alcohol among people with multiple versus fewer exposures, by exposure combination

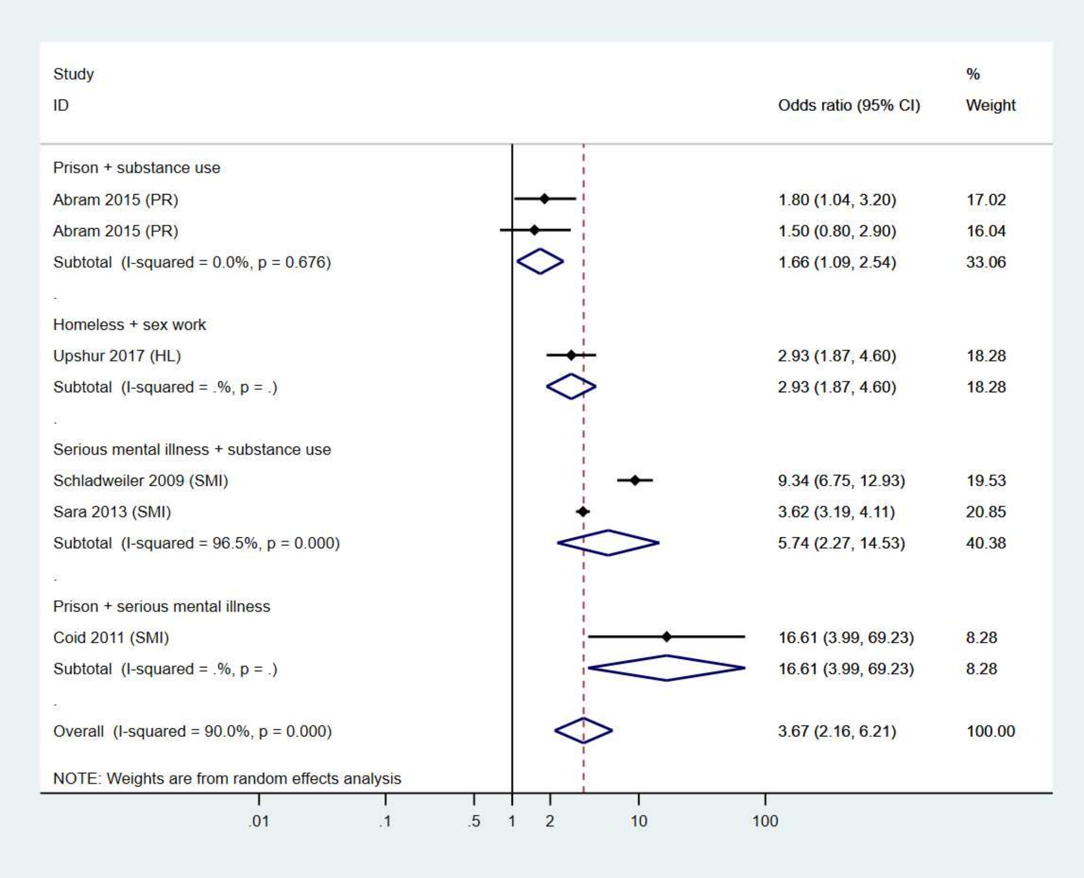

Figure A4.27. Forest plot for meta-analysis of studies reporting past year prevalence of mental and behavioural disorders due to use of alcohol among people with multiple versus fewer exposures, by gender (where reported separately)

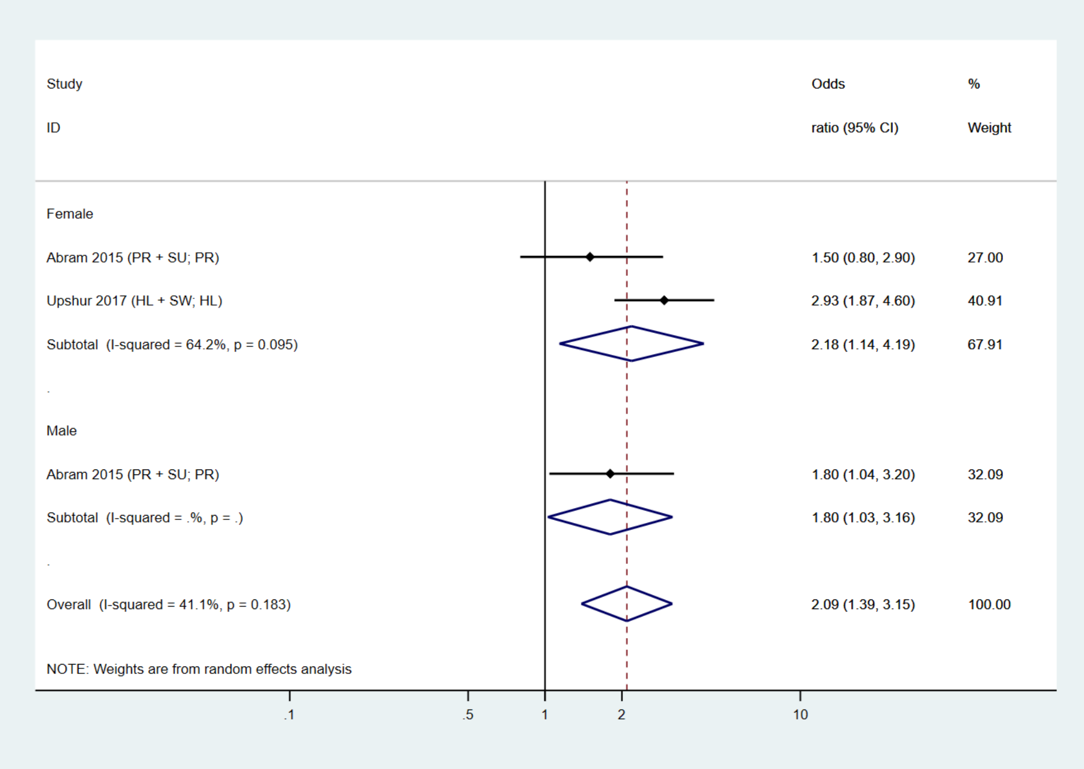

Insufficient data available to permit stratification by risk of bias.

Figure A4.28. Forest plot for meta-analysis of studies reporting lifetime prevalence of personality disorder among people with multiple versus fewer exposures, by exposure combination

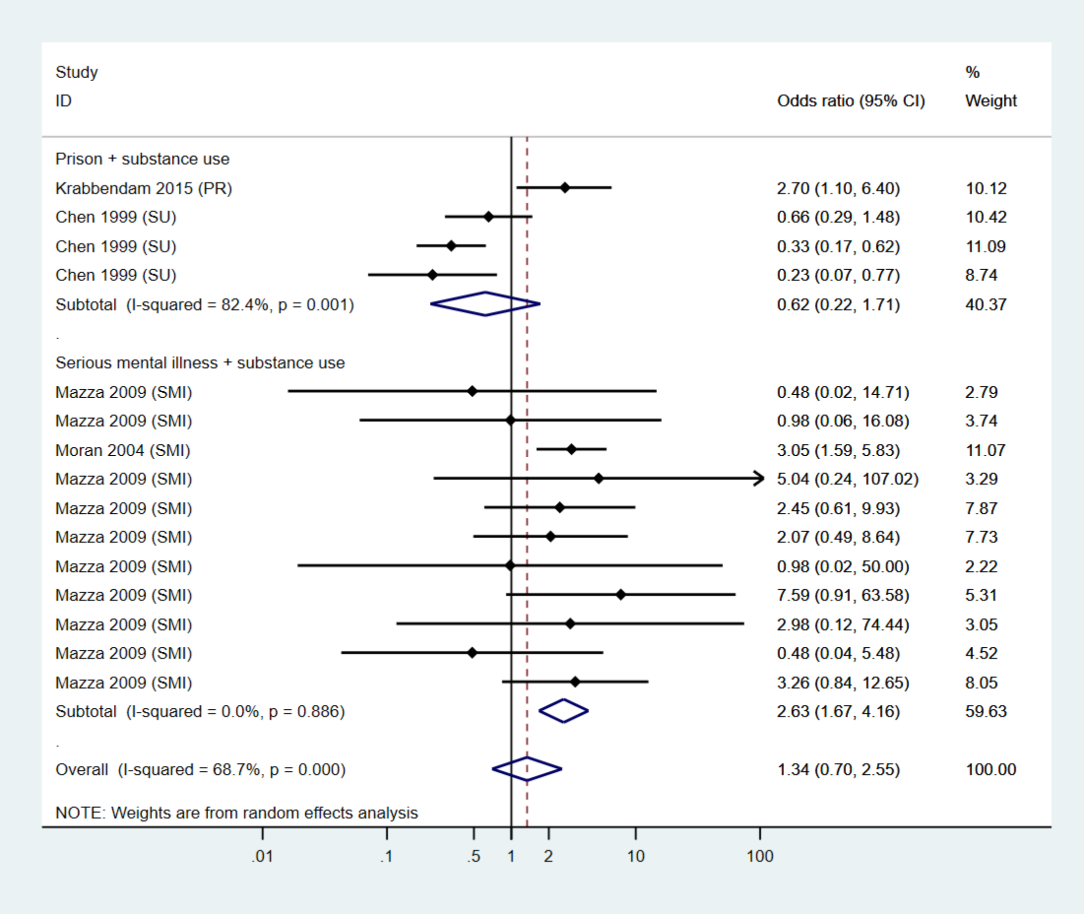

Figure A4.29. Forest plot for meta-analysis of studies reporting lifetime prevalence of personality disorder among people with multiple versus fewer exposures, by gender (where reported separately)

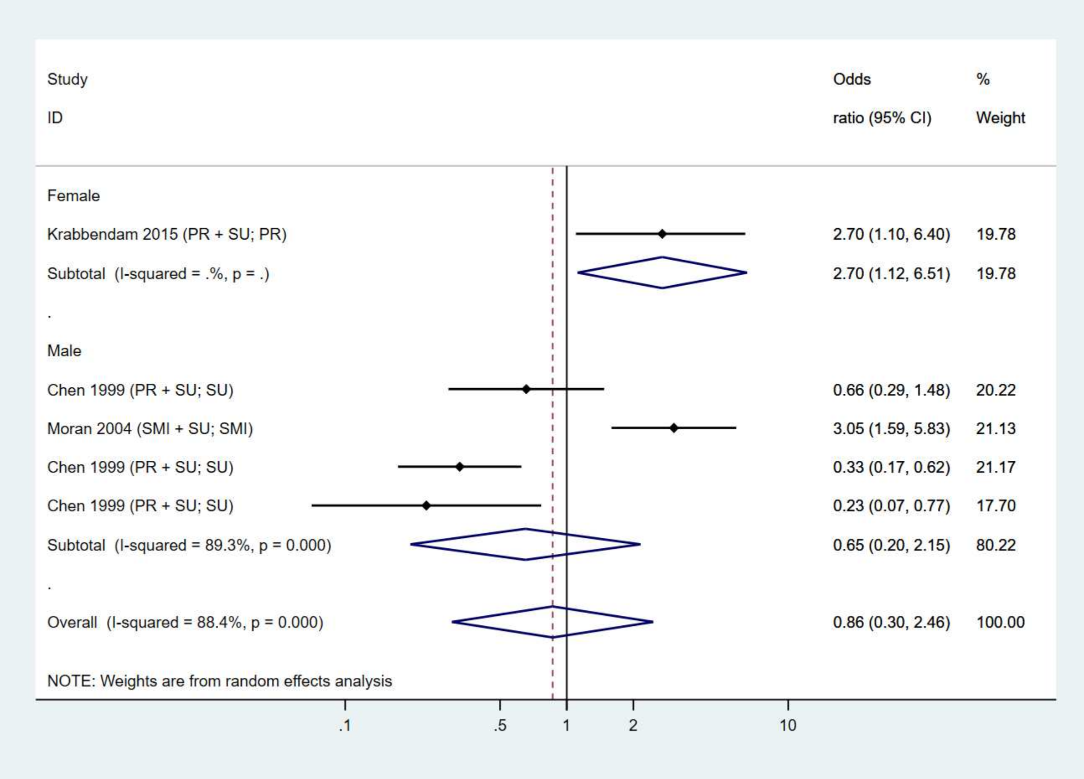

Insufficient data available to permit stratification by risk of bias.

Figure A4.30. Forest plot for meta-analysis of studies reporting lifetime prevalence of anxiety disorders among people with multiple versus fewer exposures, by exposure combination

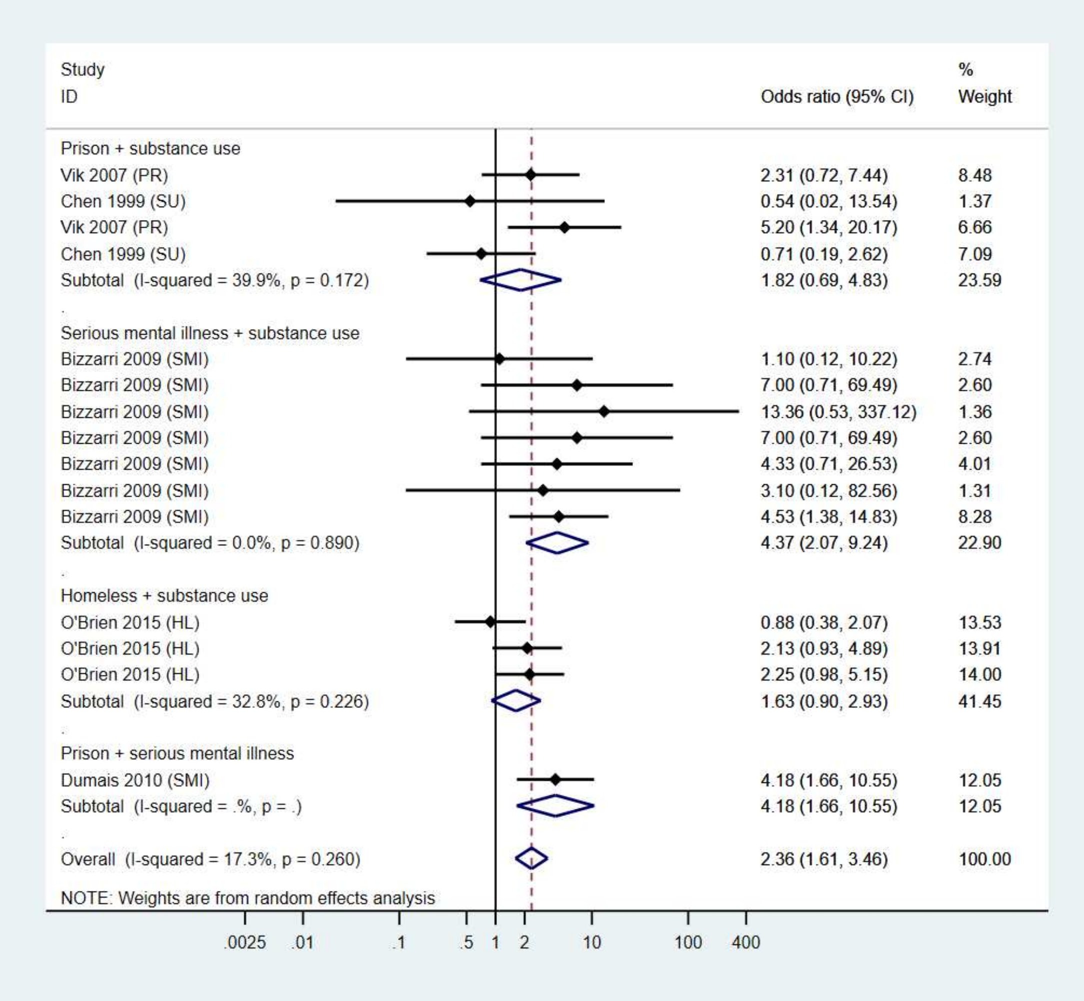

Figure A4.31. Forest plot for meta-analysis of studies reporting lifetime prevalence of anxiety disorders among people with multiple versus fewer exposures, by risk of bias

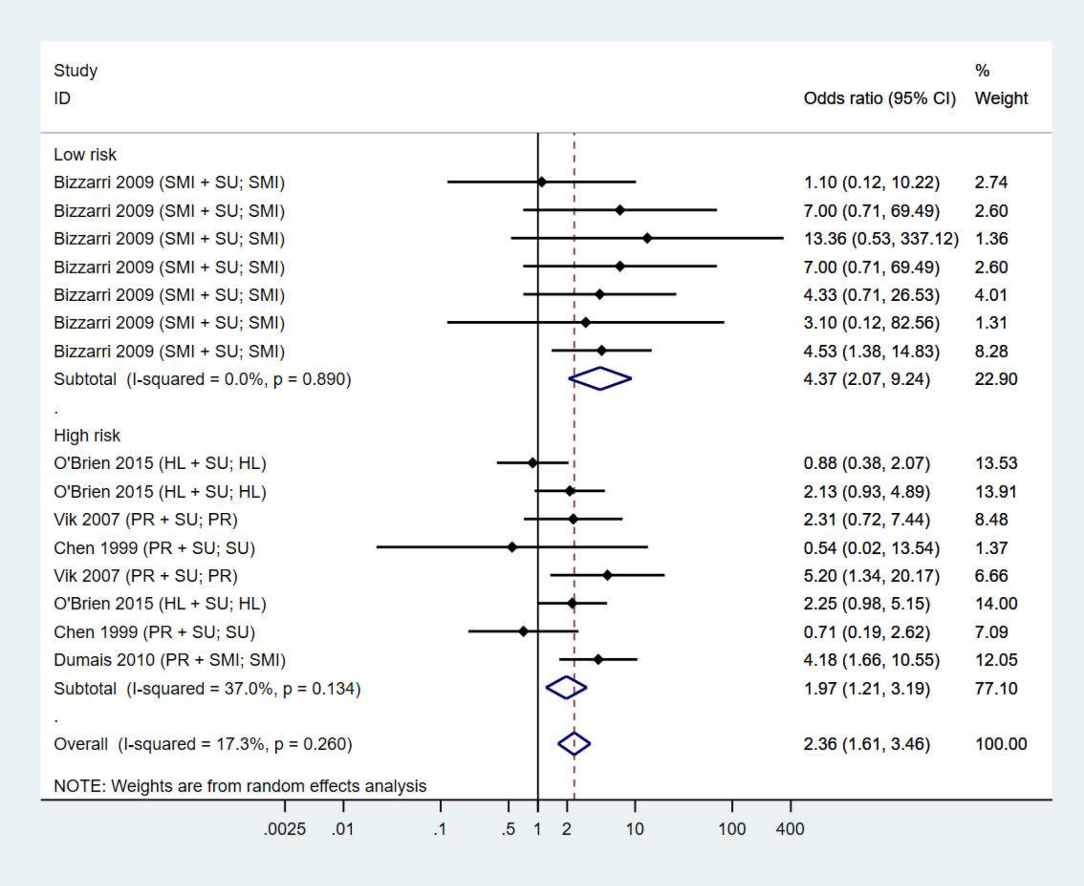

Insufficient data available to permit stratification by gender.

Figure A4.32. Forest plot for meta-analysis of studies reporting past year prevalence of anxiety disorders among people with multiple versus fewer exposures, by exposure combination

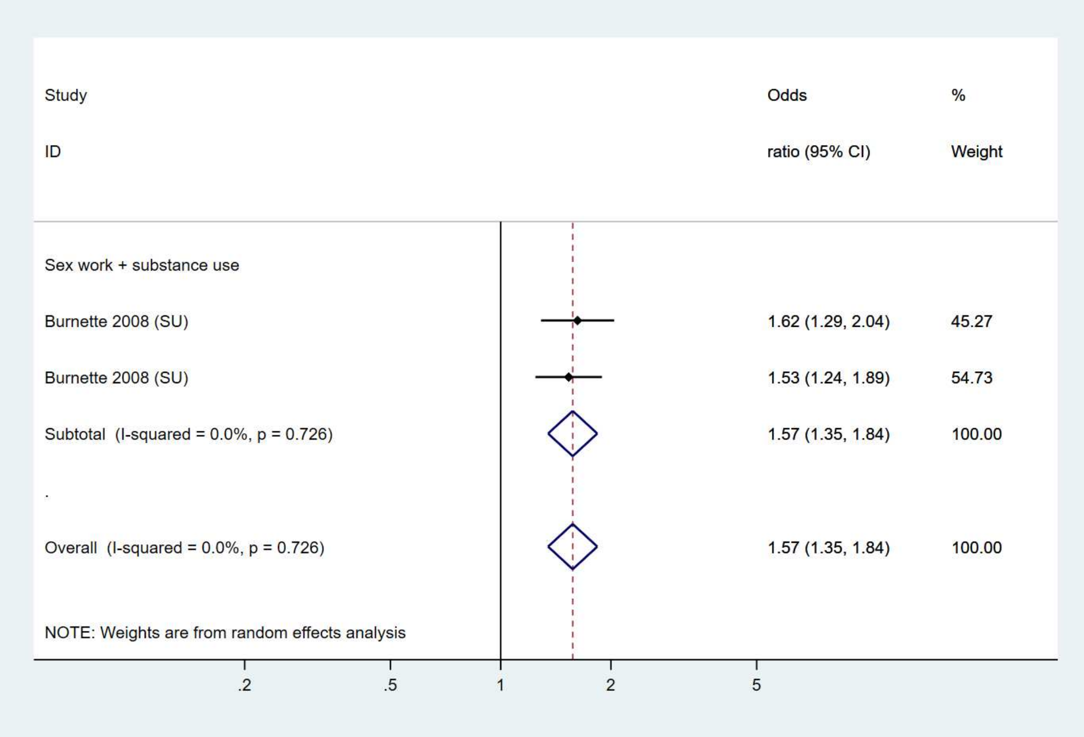

Insufficient data available to permit stratification by gender or risk of bias.

Figure A4.33. Forest plot for meta-analysis of studies reporting lifetime prevalence of depressive disorders among people with multiple versus fewer exposures, by exposure combination

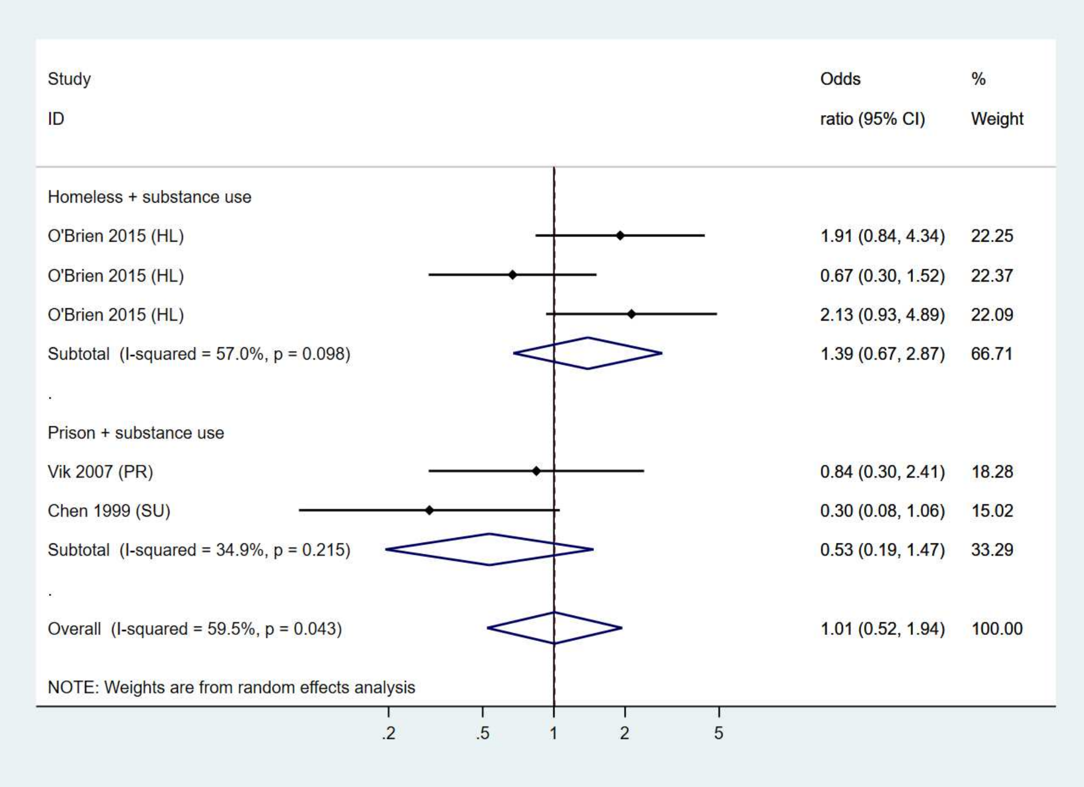

Insufficient data available to permit stratification by gender or risk of bias.

Figure A4.34. Forest plot for meta-analysis of studies reporting past year prevalence of depressive disorders among people with multiple versus fewer exposures, by exposure combination

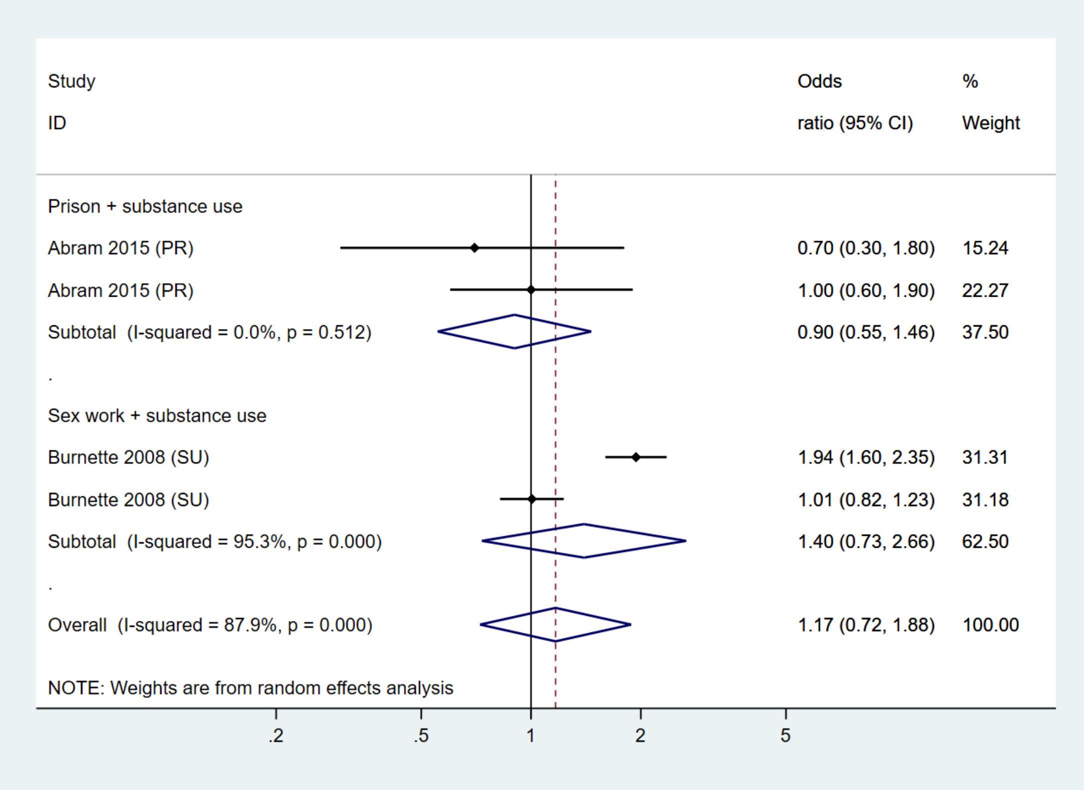

Figure A4.35. Forest plot for meta-analysis of studies reporting past year prevalence of depressive disorders among people with multiple versus fewer exposures, by gender (where reported separately)

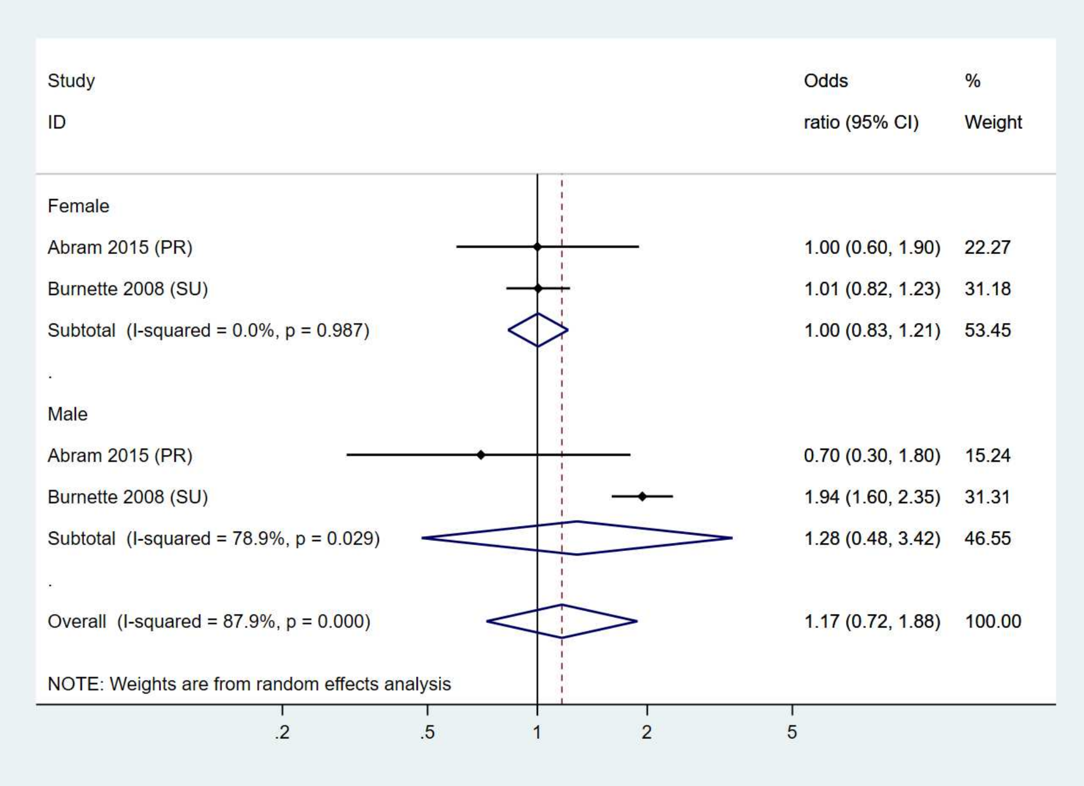

Insufficient data available to permit stratification by risk of bias.

Figure A4.36. Forest plot for meta-analysis of studies reporting past week prevalence of depressive disorders among people with multiple versus fewer exposures, by exposure combination

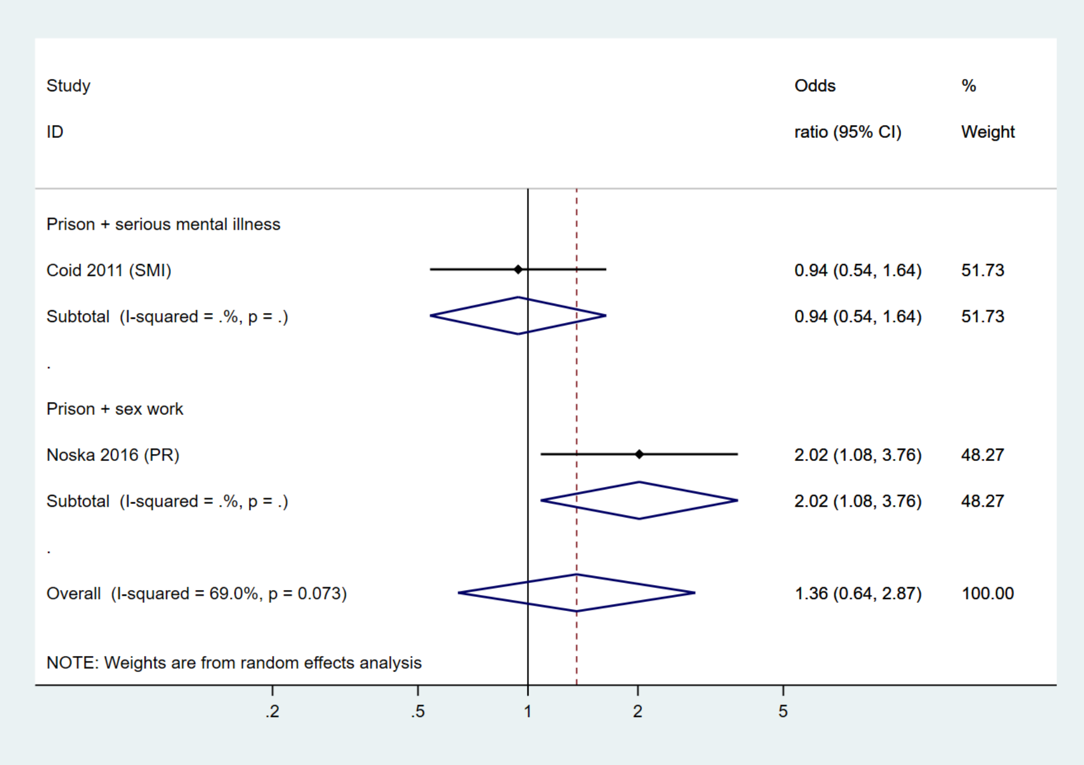

Insufficient data available to permit stratification by gender or risk of bias.

Figure A4.37. Forest plot for meta-analysis of studies reporting current prevalence of depressive disorders among people with multiple versus fewer exposures, by exposure combination

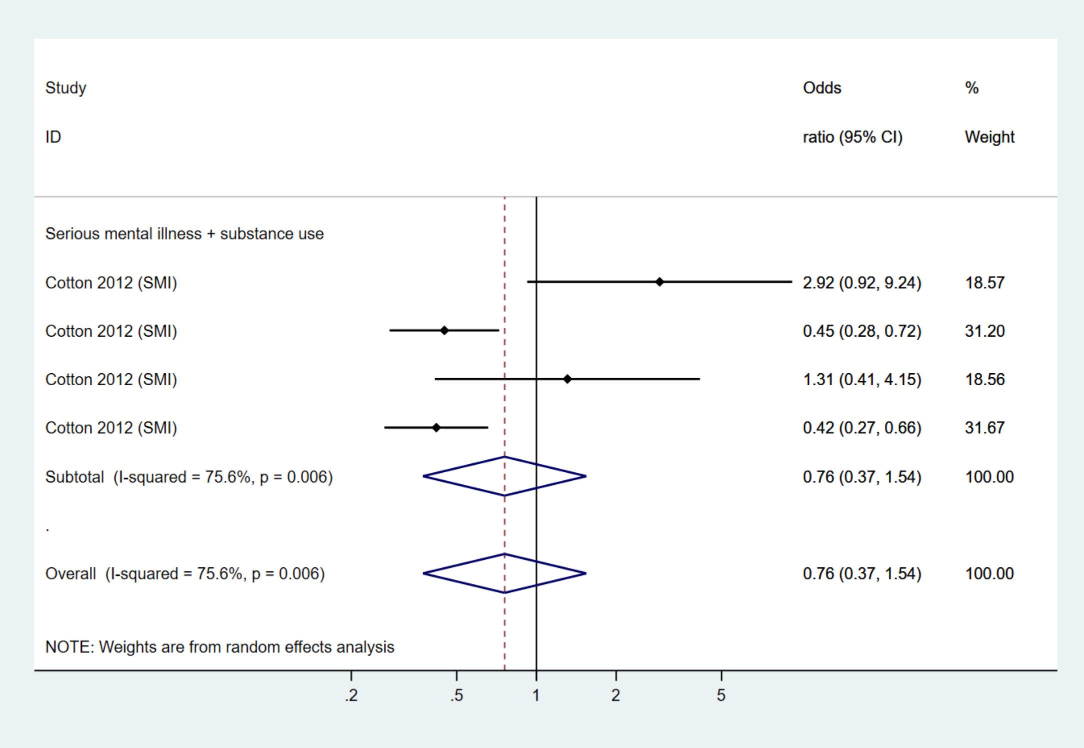

Insufficient data available to permit stratification by gender or risk of bias.

Figure A4.38. Forest plot for meta-analysis of studies reporting lifetime prevalence of dysthymia among people with multiple versus fewer exposures, by exposure combination

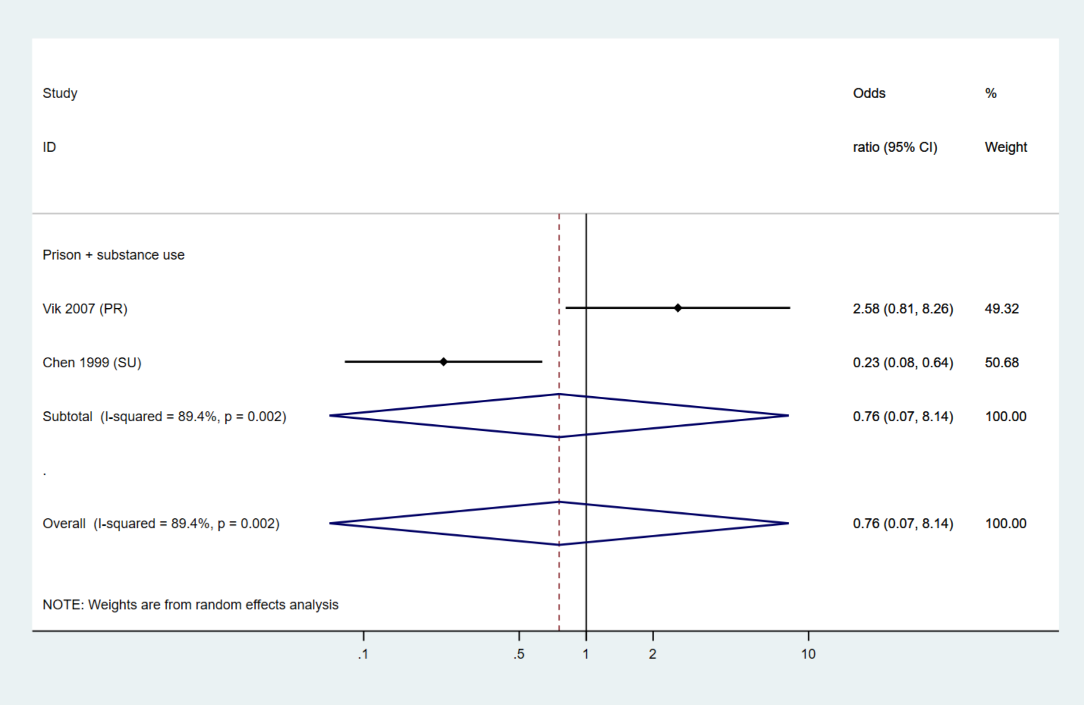

Insufficient data available to permit stratification by gender or risk of bias.

Figure A4.39. Forest plot for meta-analysis of studies reporting lifetime prevalence of obsessive compulsive disorder among people with multiple versus fewer exposures, by exposure combination

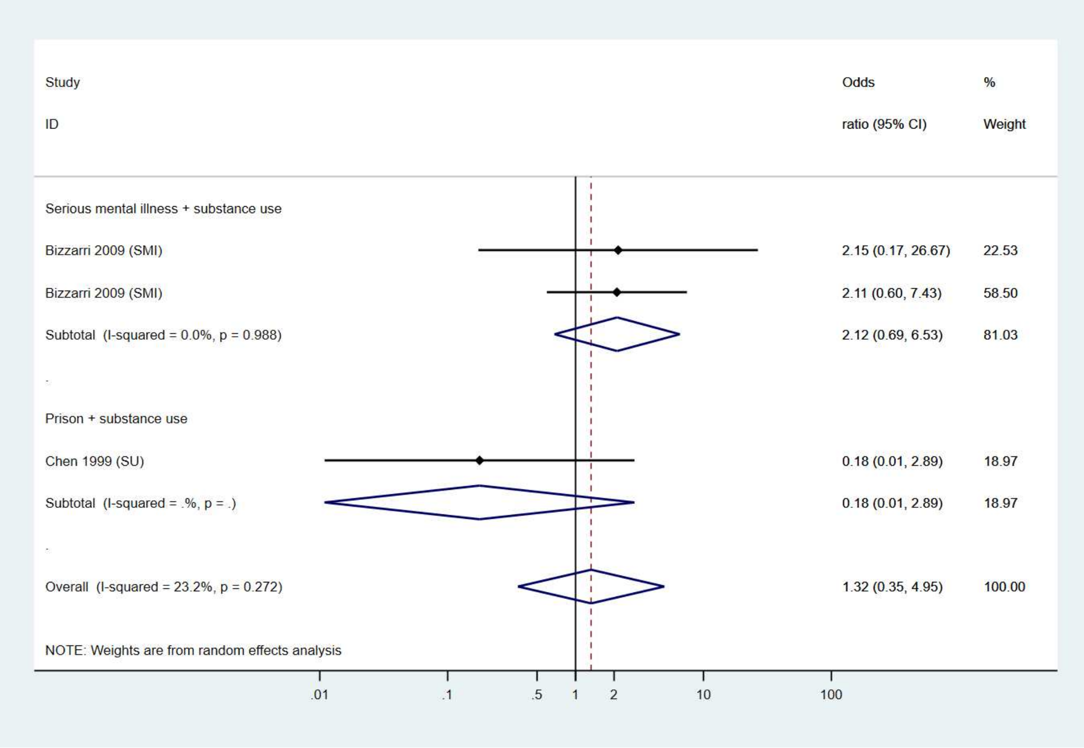

Figure A4.40. Forest plot for meta-analysis of studies reporting lifetime prevalence of obsessive compulsive disorder among people with multiple versus fewer exposures, by risk of bias

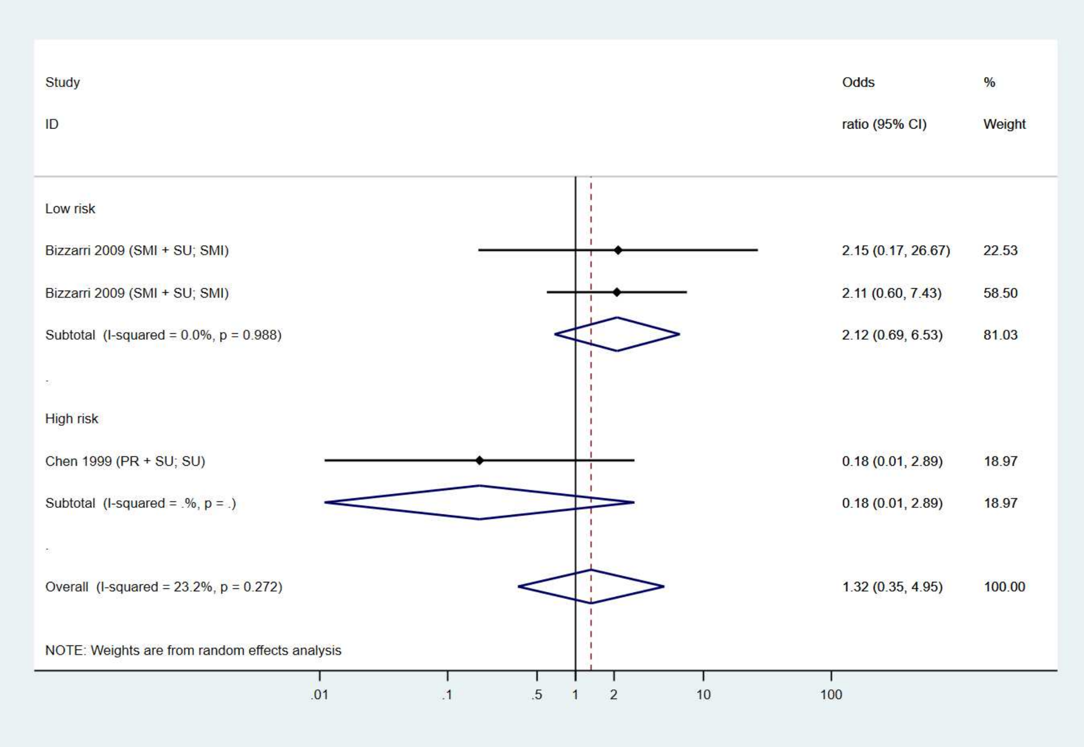

Insufficient data available to permit stratification by gender.

Figure A4.41. Forest plot for meta-analysis of studies reporting lifetime prevalence of post traumatic stress disorder among people with multiple versus fewer exposures, by exposure combination

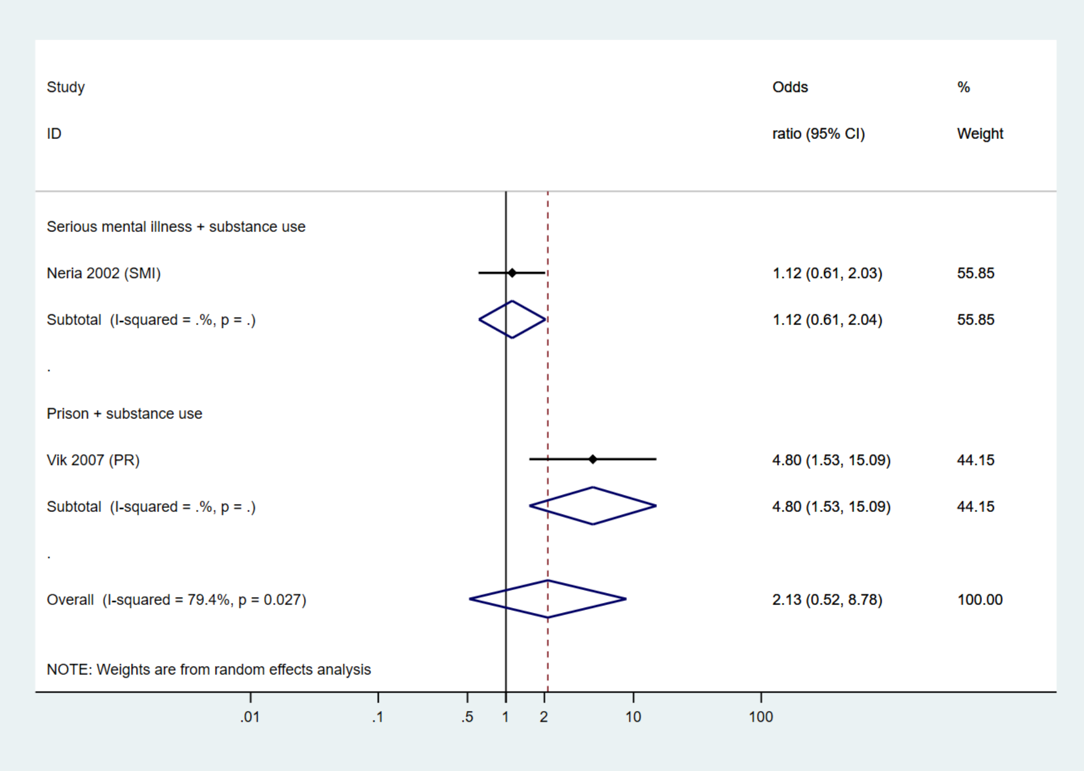

Insufficient data available to permit stratification by gender or risk of bias.

Figure A4.42. Forest plot for meta-analysis of studies reporting current prevalence of post traumatic stress disorder among people with multiple versus fewer exposures, by exposure combination

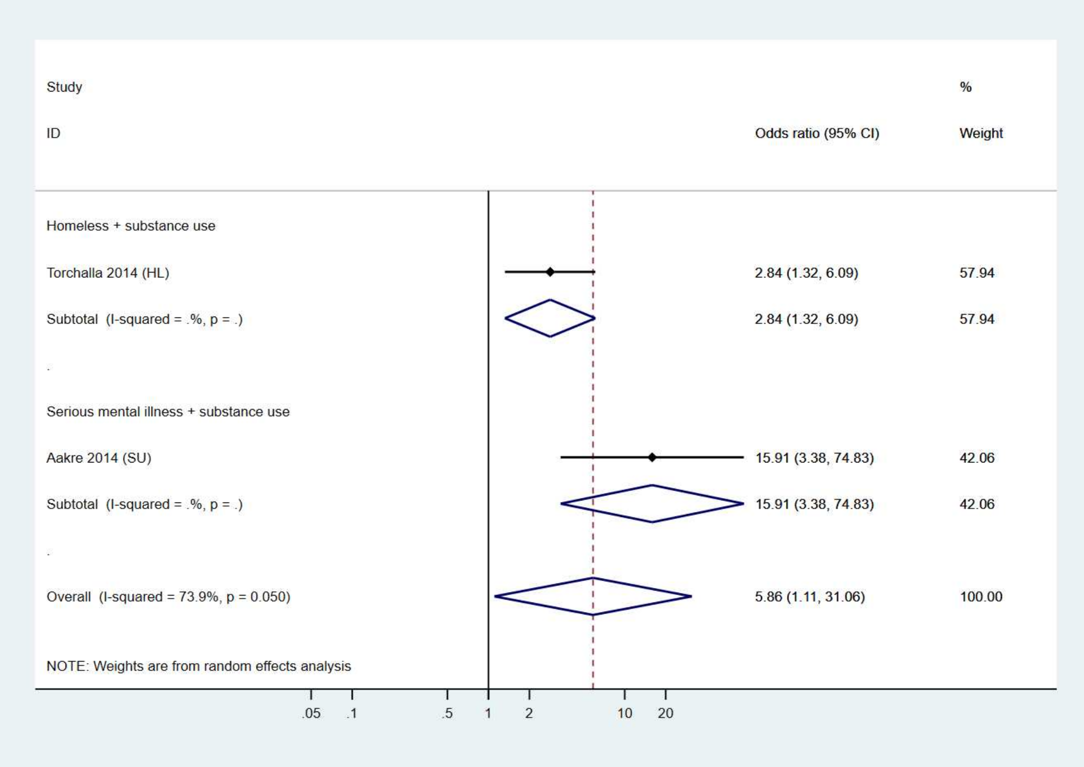

Insufficient data available to permit stratification by gender or risk of bias.

Figure A4.43. Forest plot for meta-analysis of studies reporting current prevalence of pathological gambling among people with multiple versus fewer exposures, by exposure combination

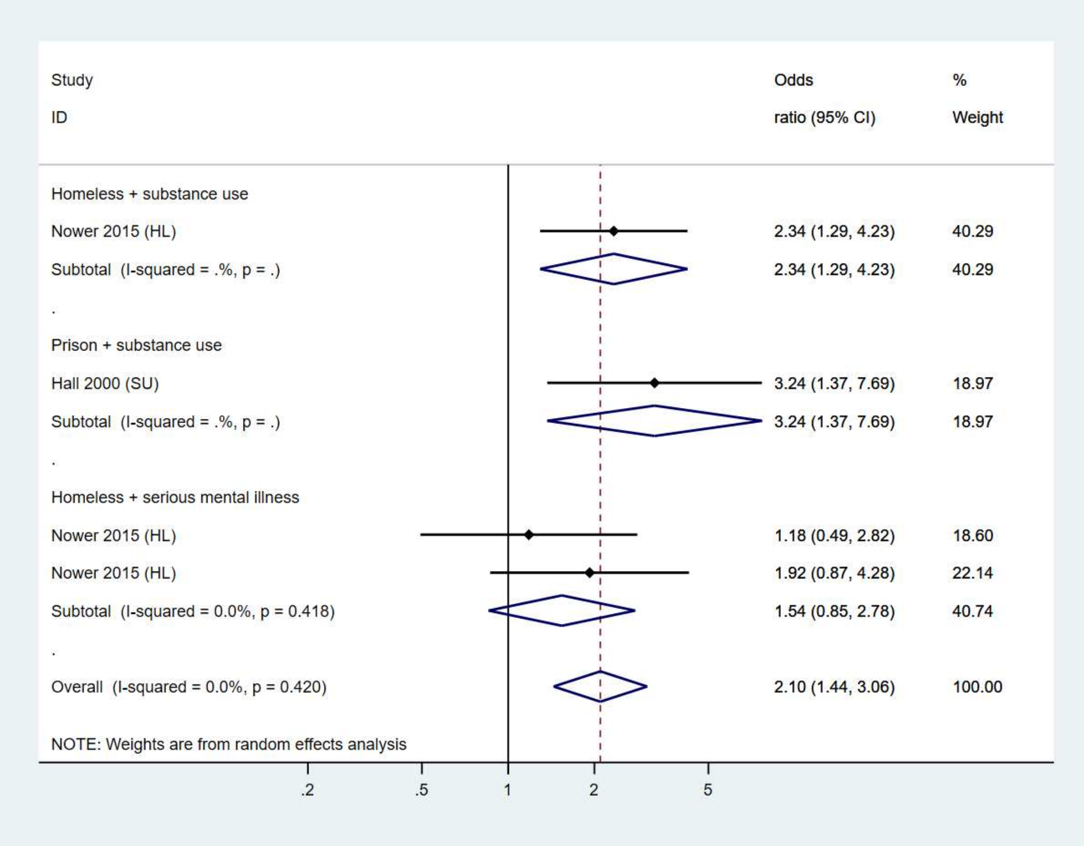

Figure A4.44. Forest plot for meta-analysis of studies reporting current prevalence of pathological gambling among people with multiple versus fewer exposures, by risk of bias

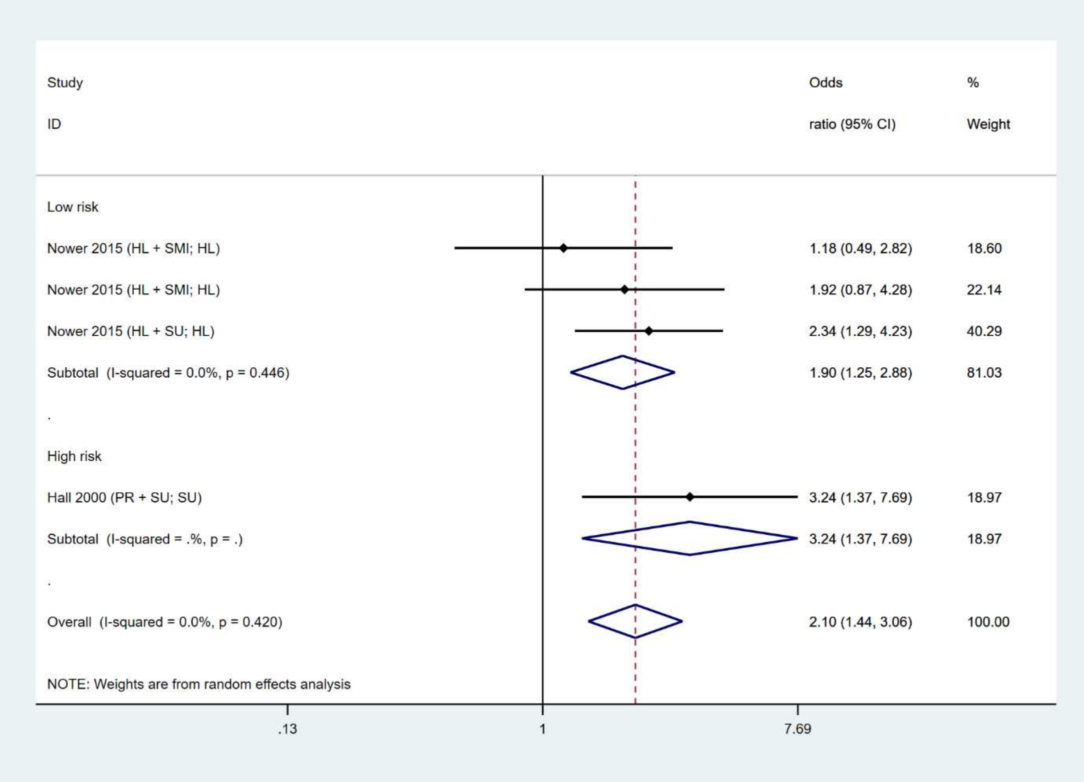

Insufficient data available to permit stratification by gender.

Figure A4.45. Forest plot for meta-analysis of studies reporting past year prevalence of skin and soft tissue infection among people with multiple versus fewer exposures, by exposure combination

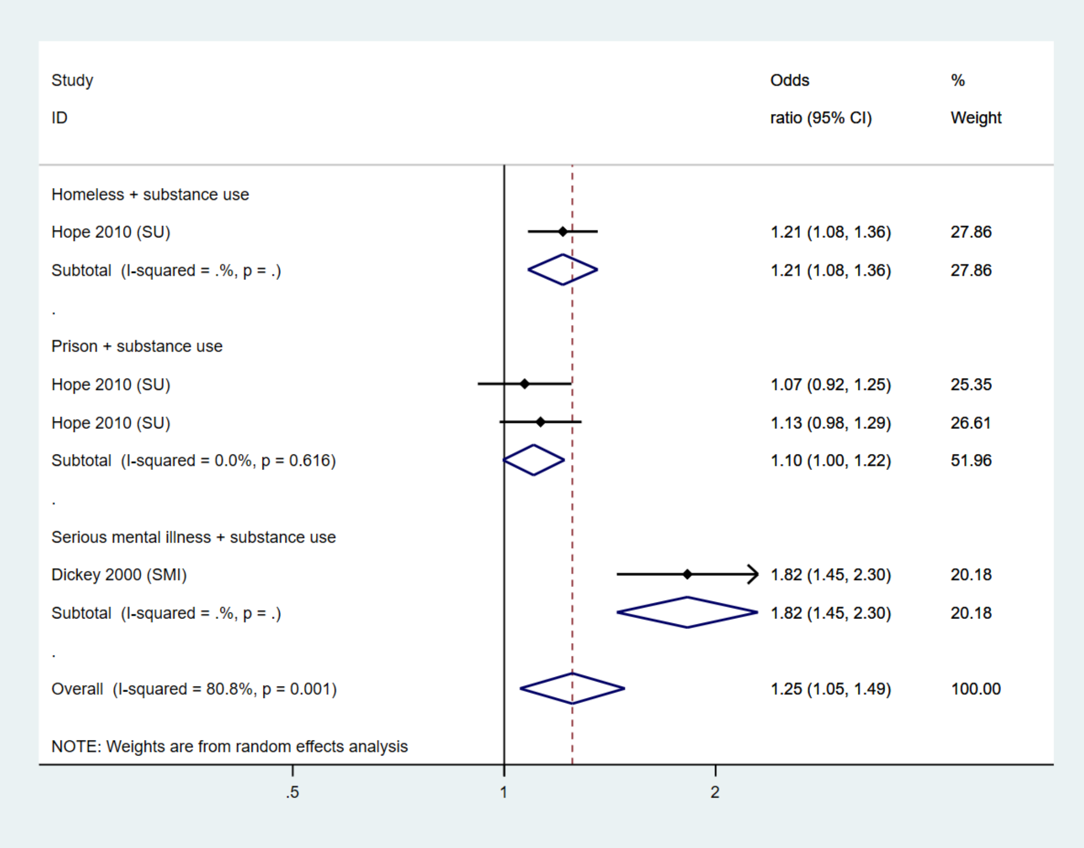

Insufficient data available to permit stratification by gender or risk of bias.

Figure A4.46. Forest plot for meta-analysis of studies reporting past six month prevalence of skin and soft tissue infection among people with multiple versus fewer exposures, by exposure combination

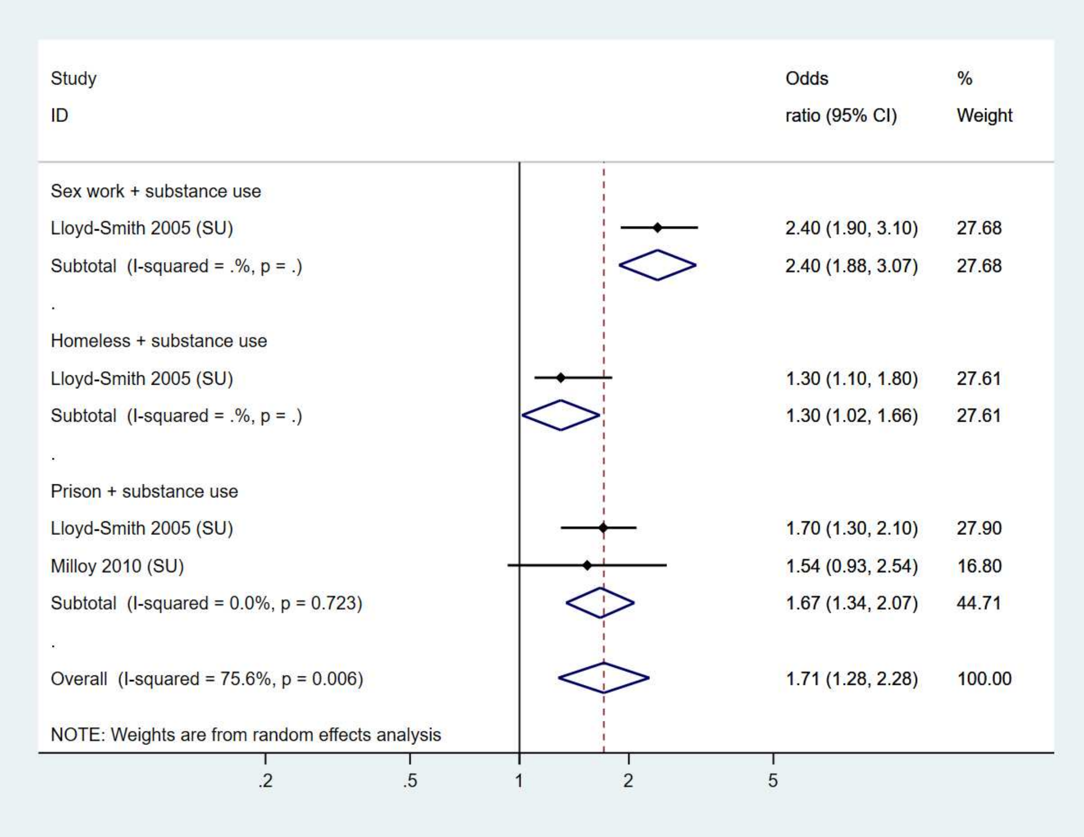

Insufficient data available to permit stratification by gender or risk of bias.

Figure A4.47. Forest plot for meta-analysis of studies reporting incidence of skin and soft tissue infection among people with multiple versus fewer exposures, by exposure combination

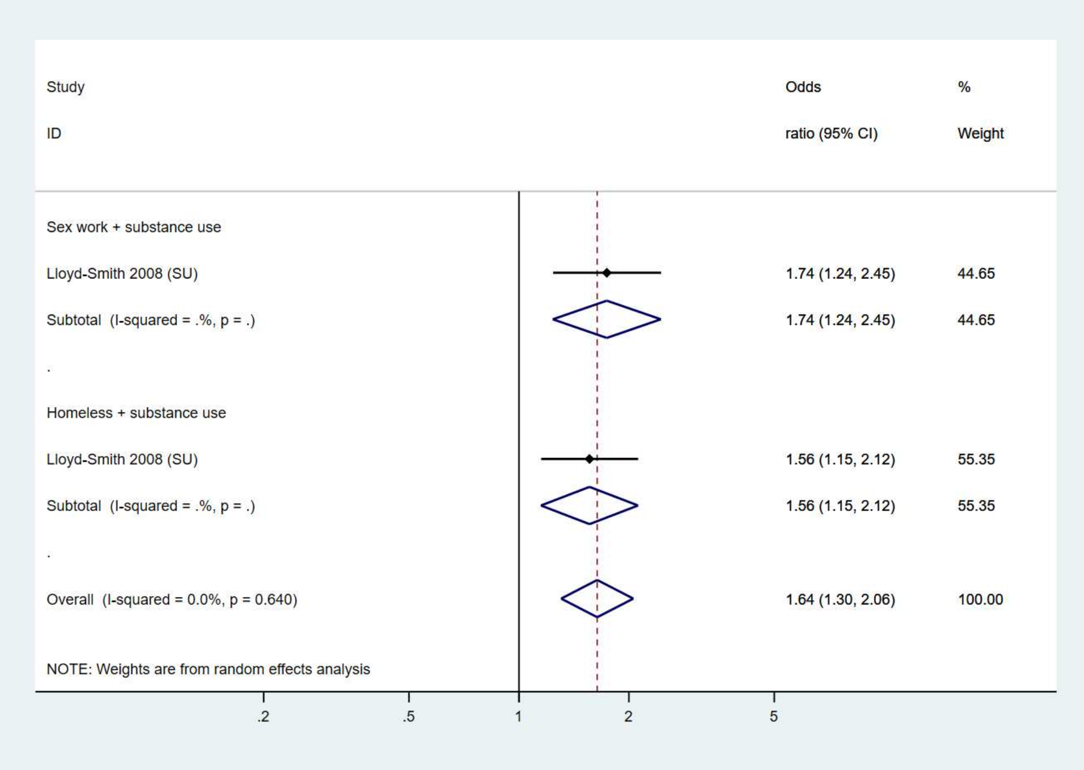

Insufficient data available to permit stratification by gender or risk of bias.

Figure A4.48. Forest plot for meta-analysis of studies reporting current prevalence of amenorrhoea among people with multiple versus fewer exposures, by exposure combination

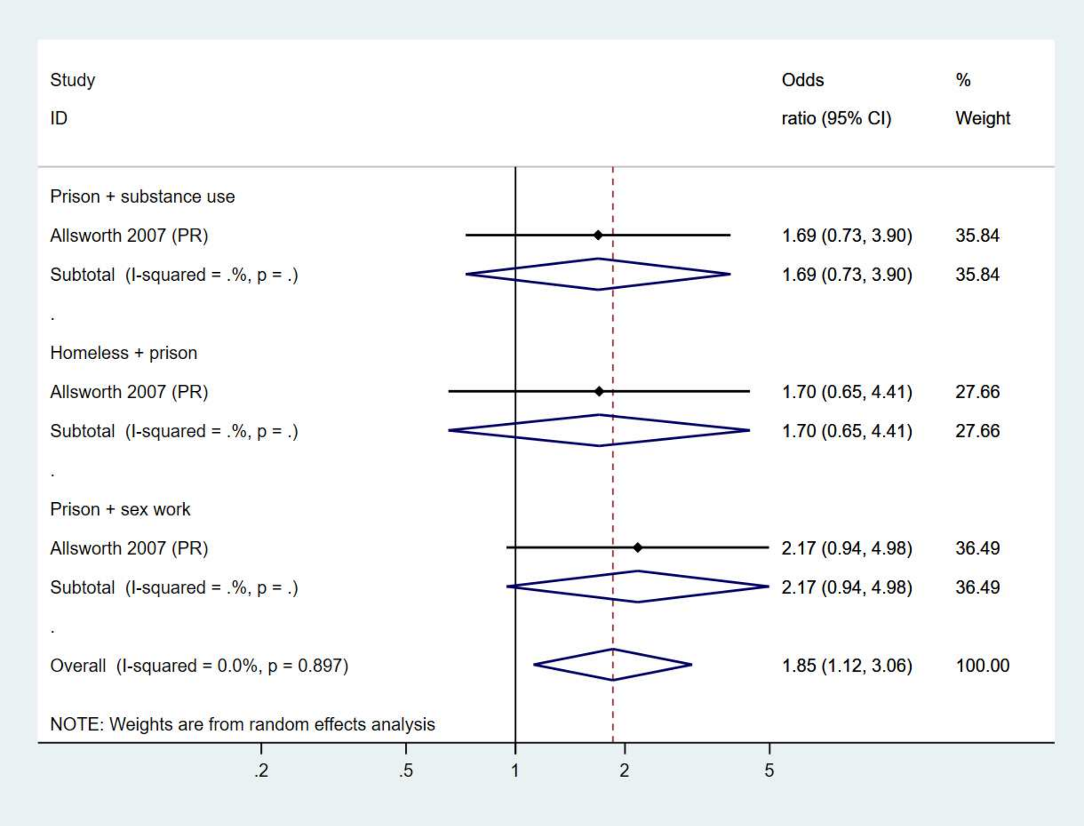

Insufficient data available to permit stratification by risk of bias.

Figure A4.49. Forest plot for meta-analysis of studies reporting current prevalence of irregular menstruation among people with multiple versus fewer exposures, by exposure combination

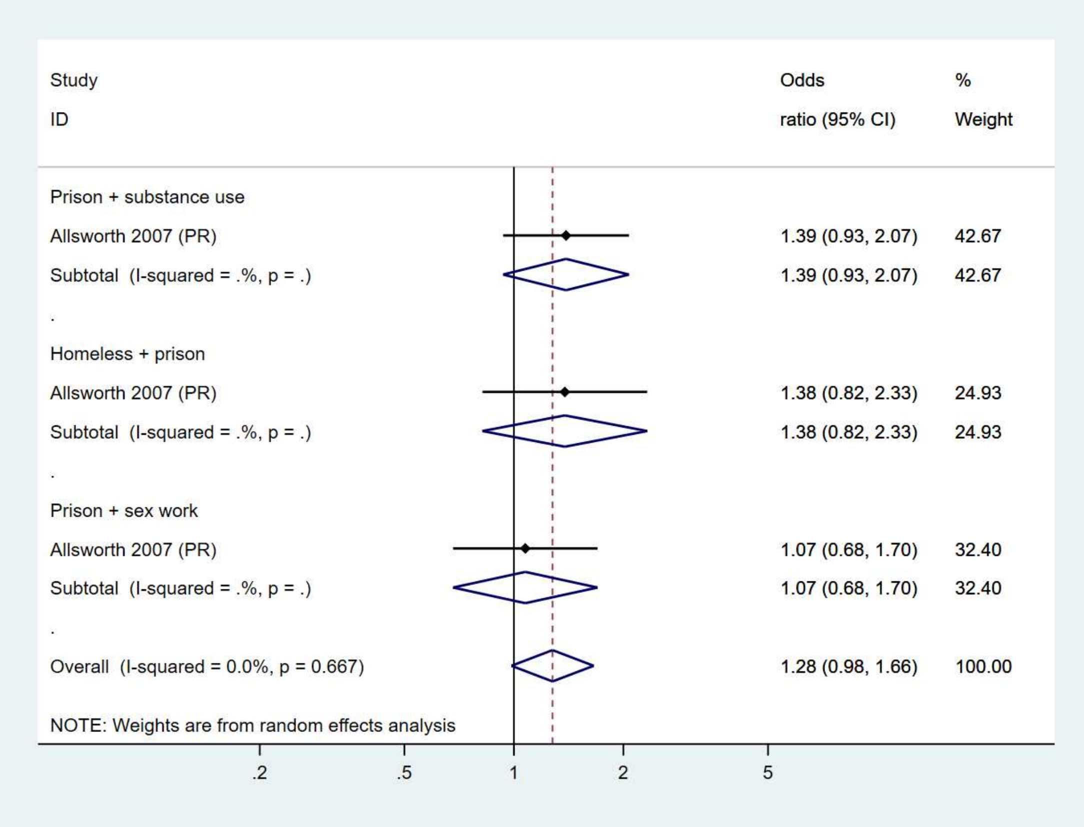

Insufficient data available to permit stratification by risk of bias.

Figure A4.50. Forest plot for meta-analysis of studies reporting lifetime prevalence of non-fatal drug overdose among people with multiple versus fewer exposures, by exposure combination

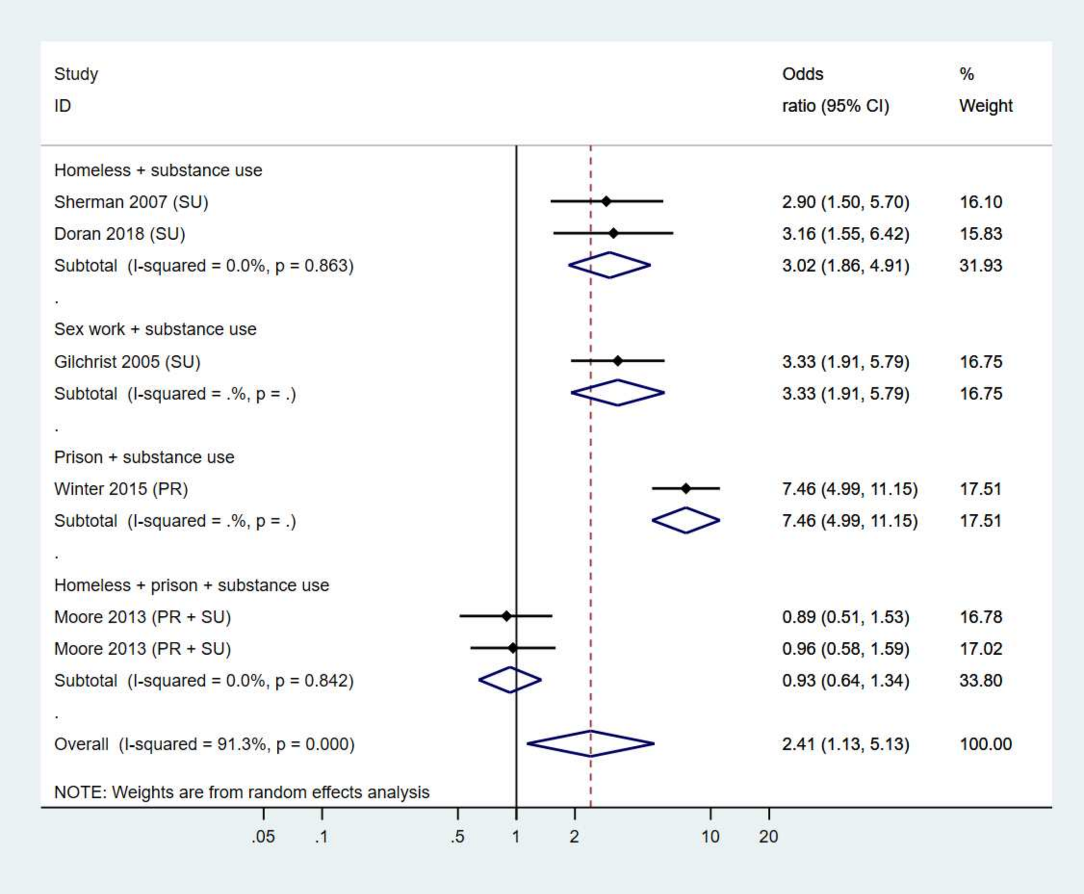

Figure A4.51. Forest plot for meta-analysis of studies reporting lifetime prevalence of non-fatal drug overdose among people with multiple versus fewer exposures, by risk of bias

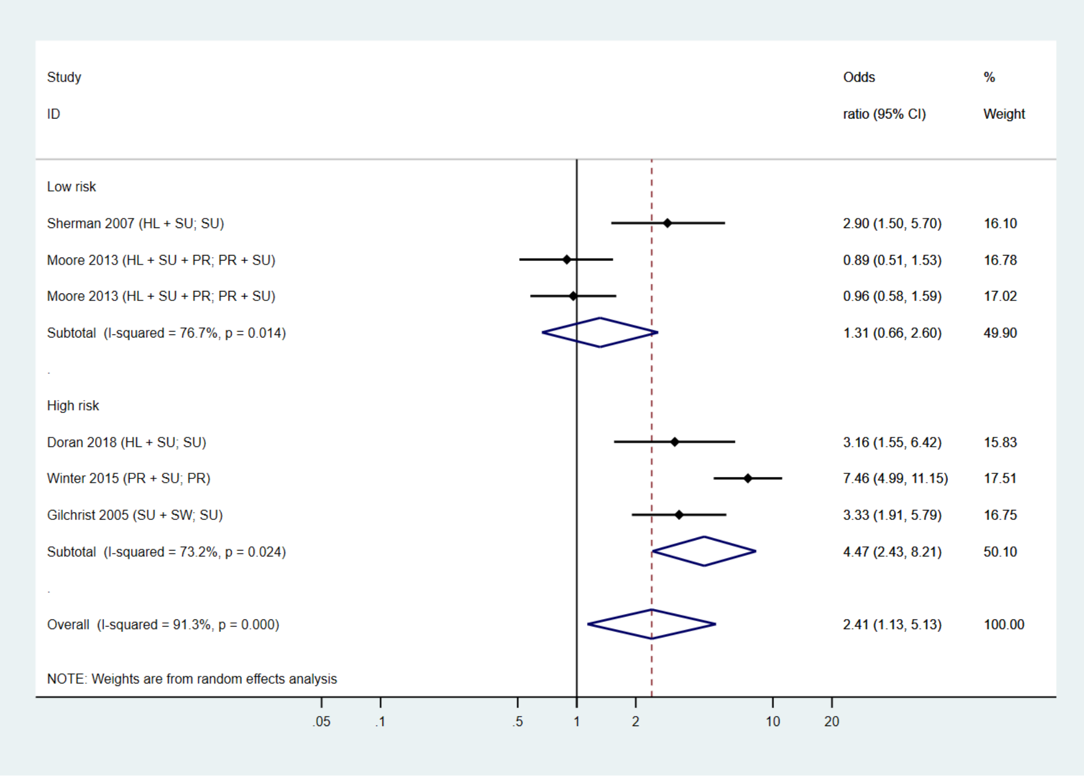

Insufficient data available to permit stratification by gender.

Figure A4.52. Forest plot for meta-analysis of studies reporting past 18 months prevalence of non-fatal drug overdose among people with multiple versus fewer exposures, by exposure combination

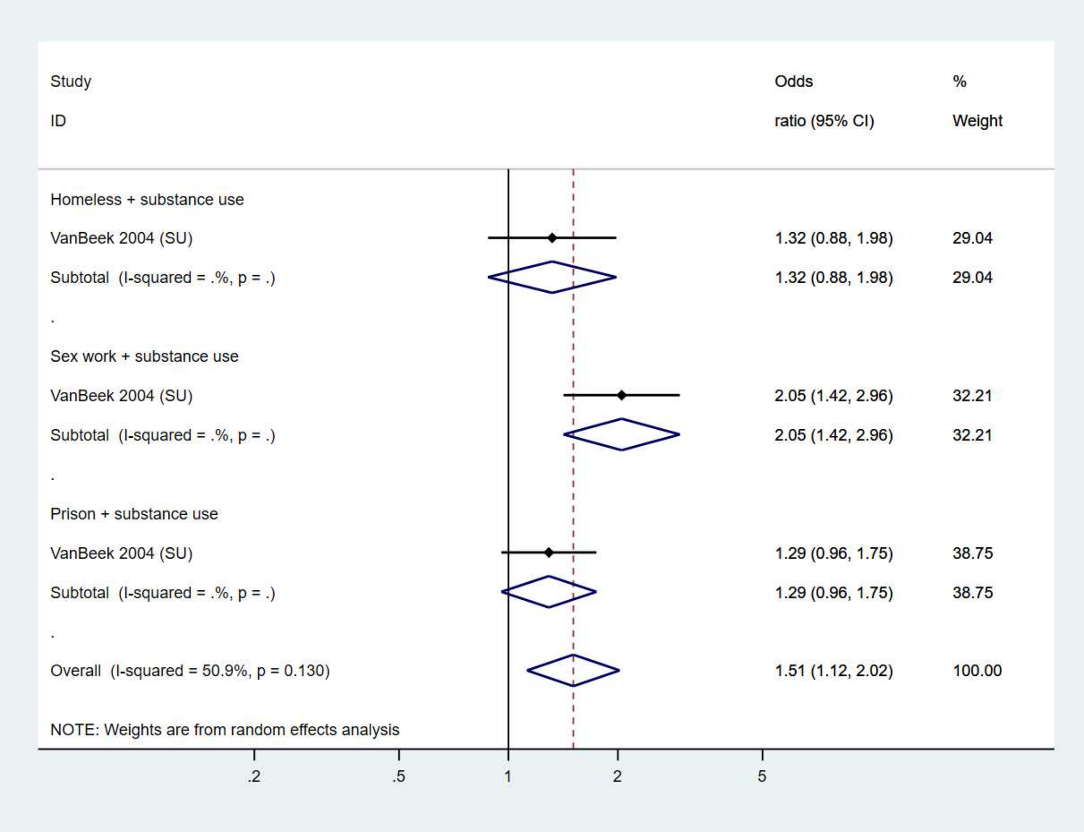

Insufficient data available to permit stratification by risk of bias or gender.

Figure A4.53. Forest plot for meta-analysis of studies reporting past year prevalence of non-fatal drug overdose among people with multiple versus fewer exposures, by exposure combination

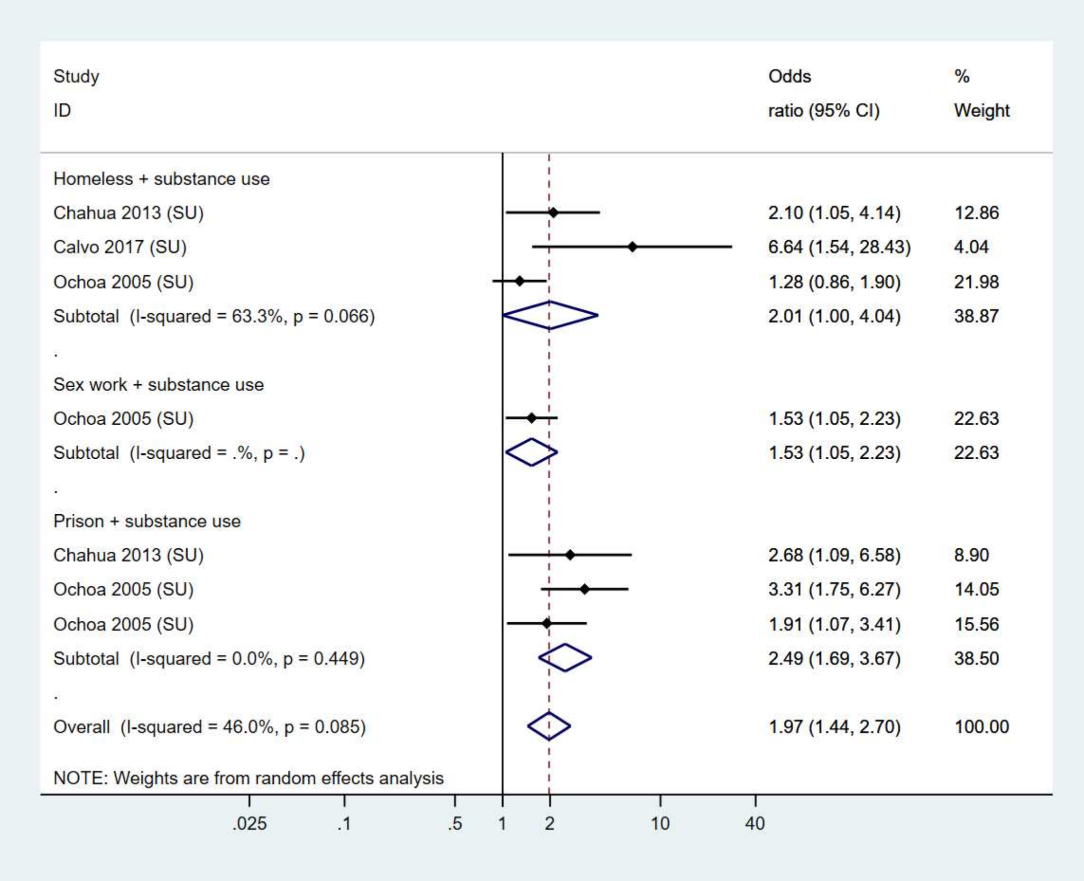

Figure A4.54. Forest plot for meta-analysis of studies reporting past year prevalence of non-fatal drug overdose among people with multiple versus fewer exposures, by risk of bias

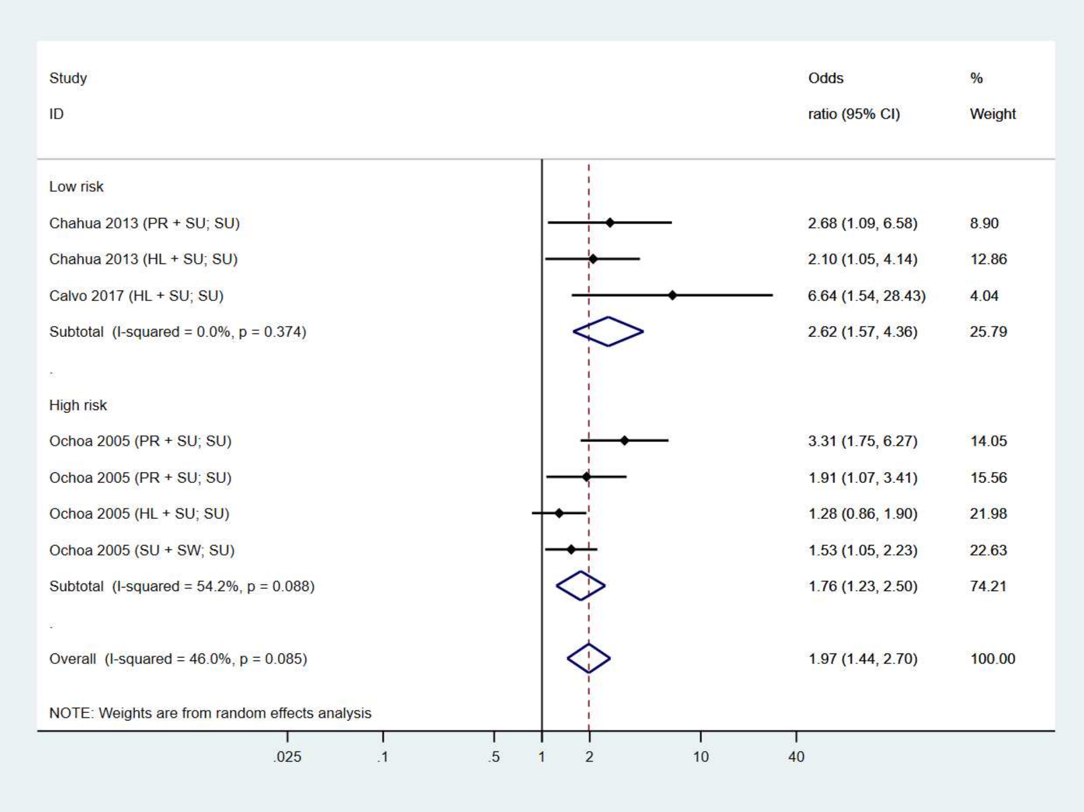

Insufficient data available to permit stratification by gender.

Figure A4.55. Forest plot for meta-analysis of studies reporting six month prevalence of non-fatal drug overdose among people with multiple versus fewer exposures, by exposure combination

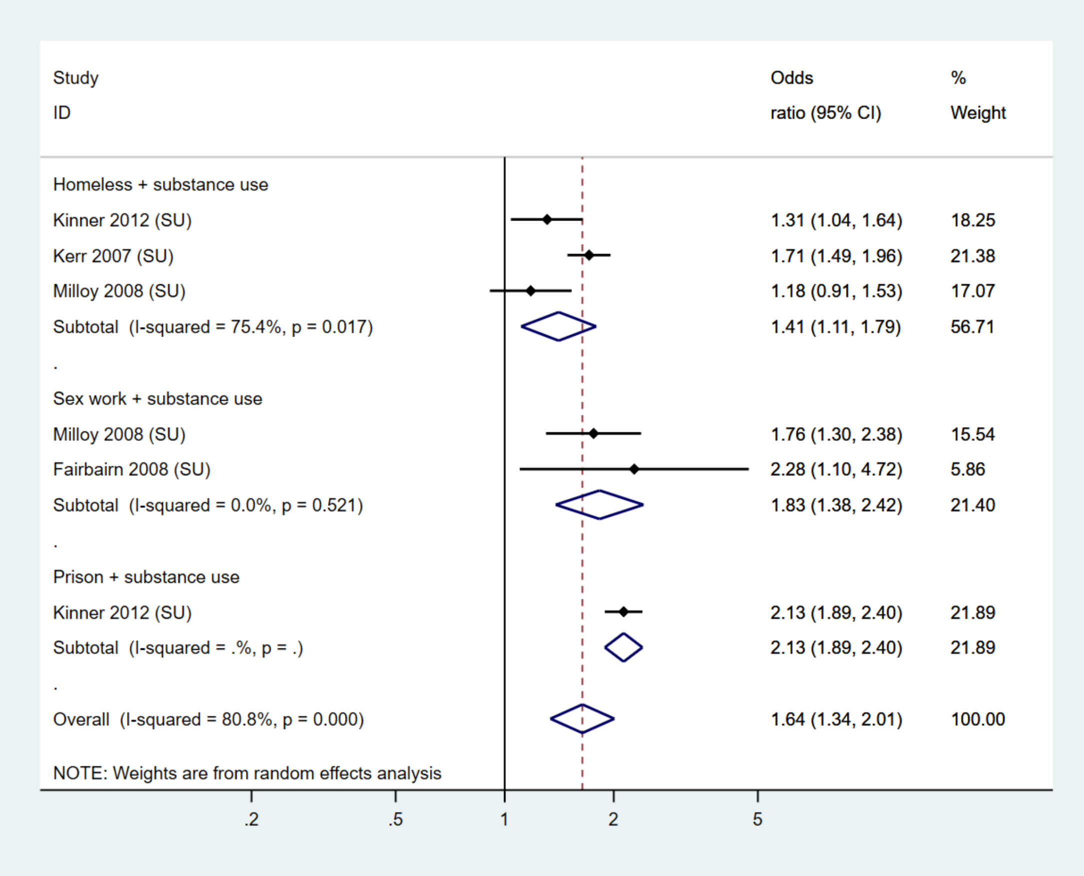

Figure A4.56. Forest plot for meta-analysis of studies reporting six month prevalence of non-fatal drug overdose among people with multiple versus fewer exposures, by risk of bias

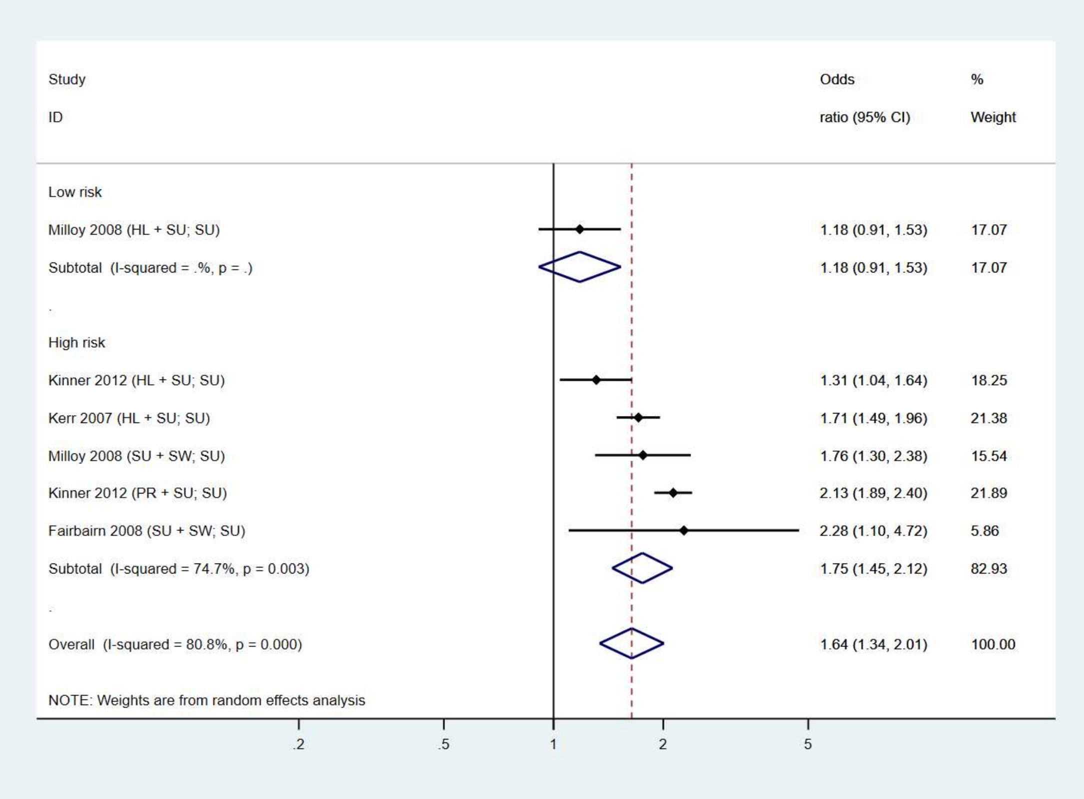

Insufficient data available to permit stratification by gender.

Figure A4.57. Forest plot for meta-analysis of studies reporting one month prevalence of non-fatal drug overdose among people with multiple versus fewer exposures, by exposure combination

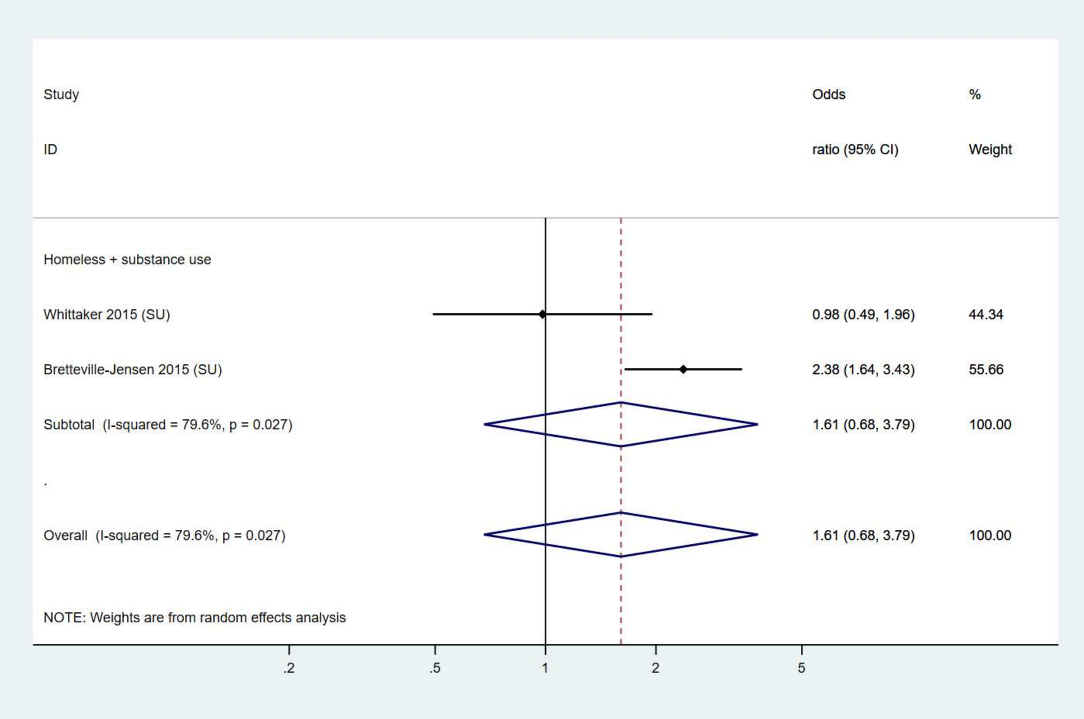

Insufficient data available to permit stratification by gender or risk of bias.

Figure A4.58. Forest plot for meta-analysis of studies reporting hazard ratios for non-fatal drug overdose among people with multiple versus fewer exposures, by exposure combination

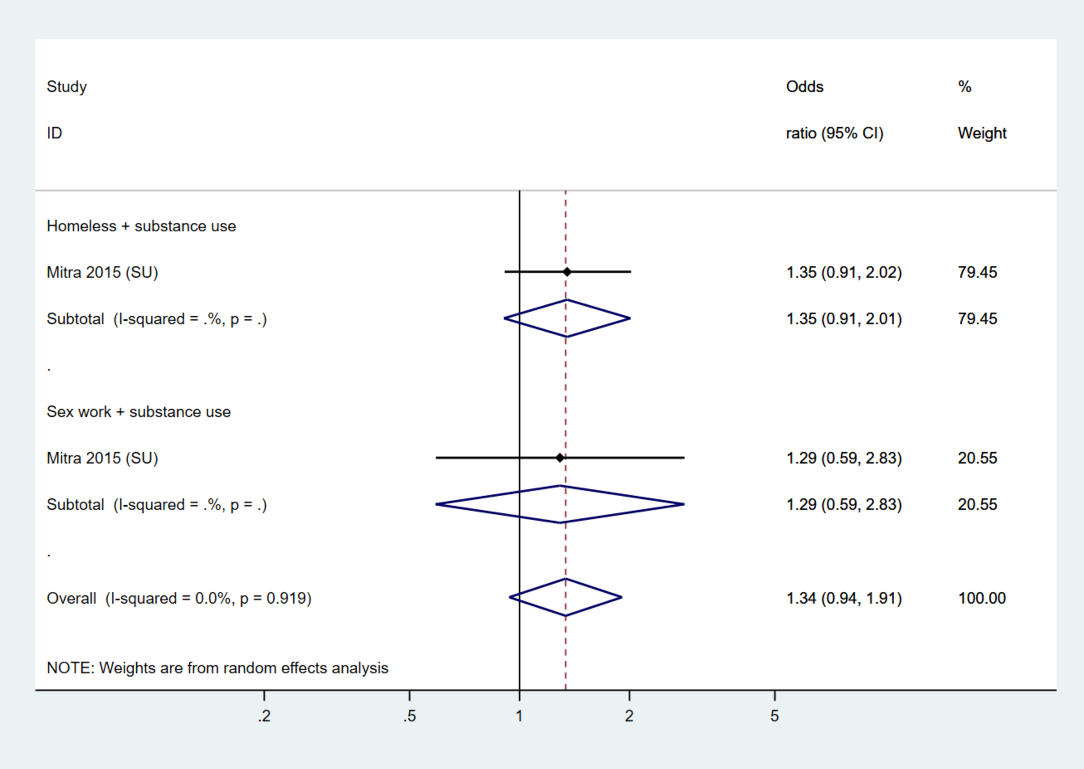

Insufficient data available to permit stratification by gender or risk of bias.

Figure A4.59. Forest plot for meta-analysis of studies reporting hazard ratios for overdose mortality among people with multiple versus fewer exposures, by exposure combination

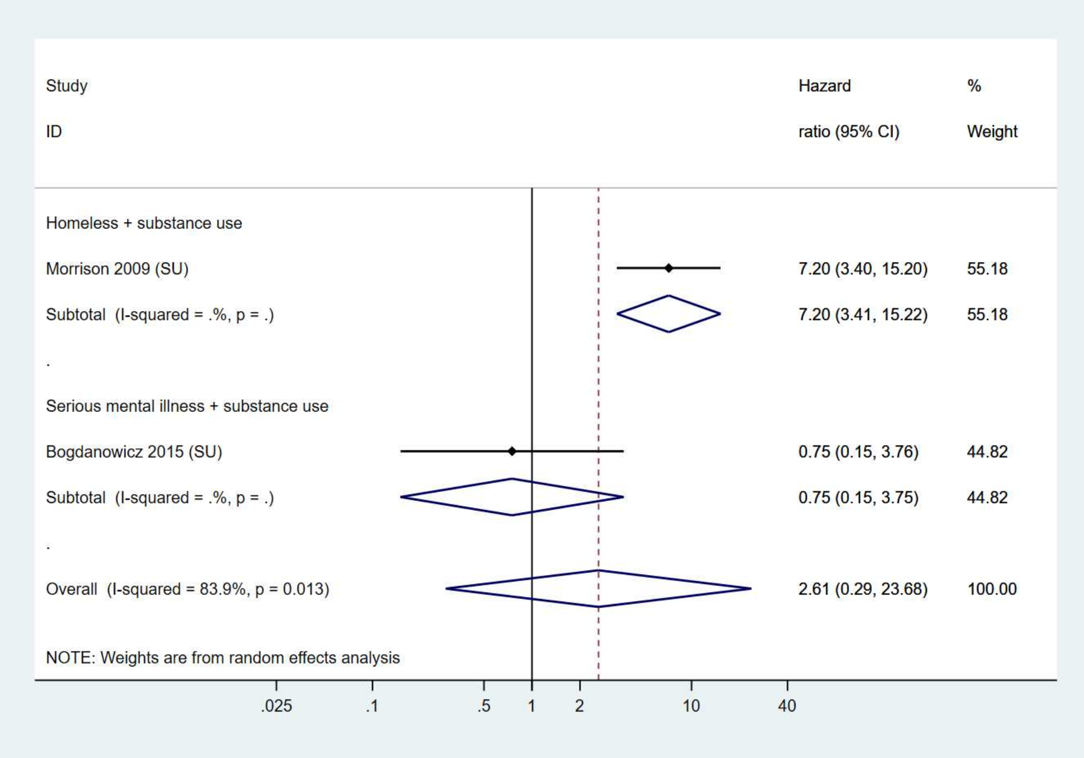

Insufficient data available to permit stratification by gender or risk of bias.

Figure A4.60. Forest plot for meta-analysis of studies reporting odds ratios for overdose mortality among people with multiple versus fewer exposures, by exposure combination

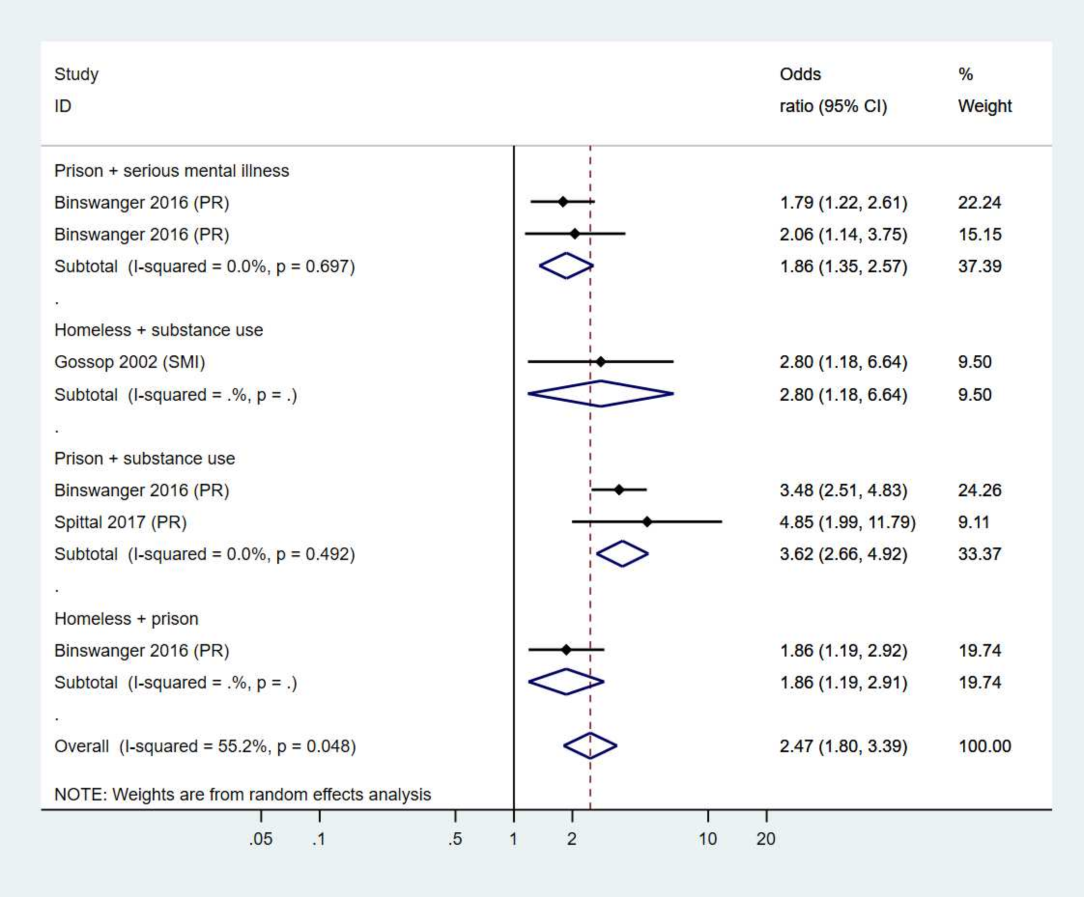

Figure A4.61. Forest plot for meta-analysis of studies reporting odds ratios for overdose mortality among people with multiple versus fewer exposures, by risk of bias

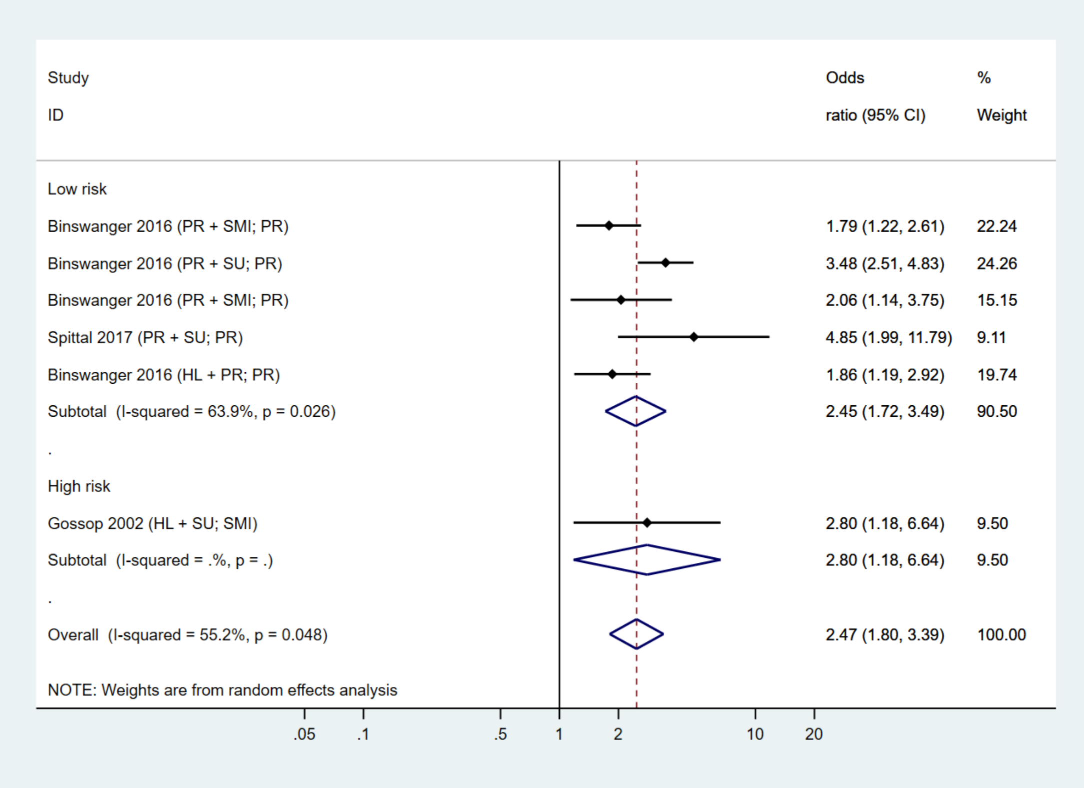

Insufficient data available to permit stratification by gender.

Figure A4.62. Forest plot for meta-analysis of studies reporting relative risks for overdose mortality among people with multiple versus fewer exposures, by exposure combination

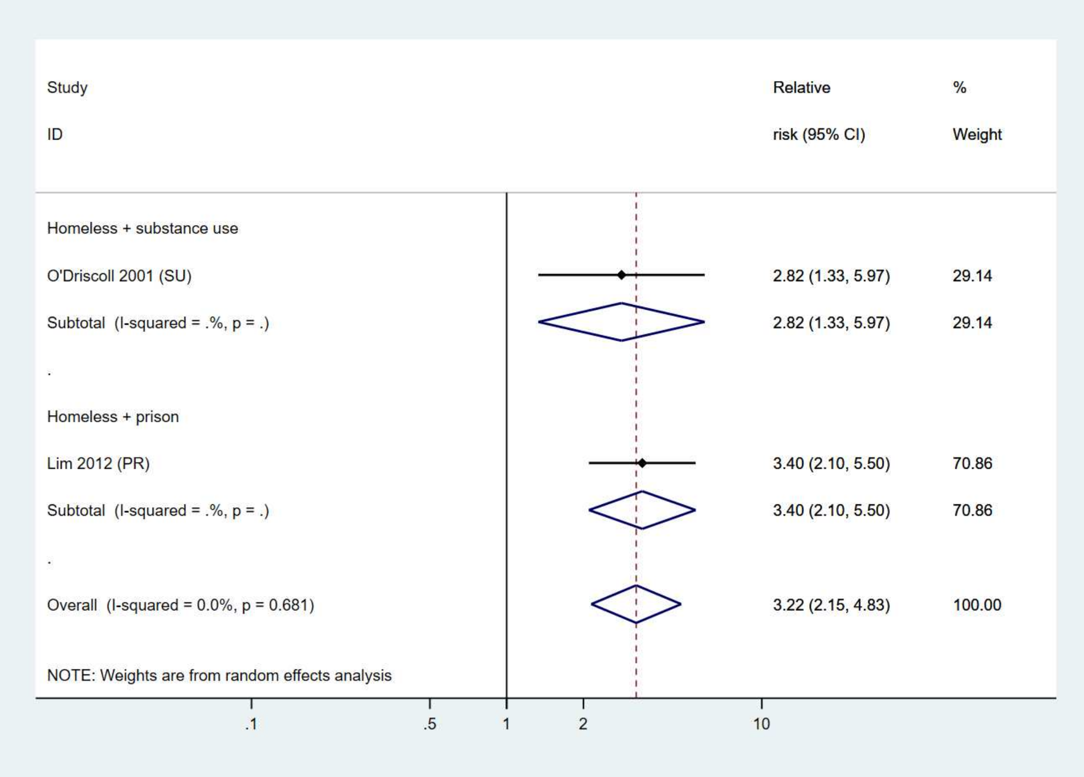

Insufficient data available to permit stratification by gender or risk of bias.

Figure A4.63. Forest plot for meta-analysis of studies reporting lifetime prevalence of head injury among people with multiple versus fewer exposures, by exposure combination

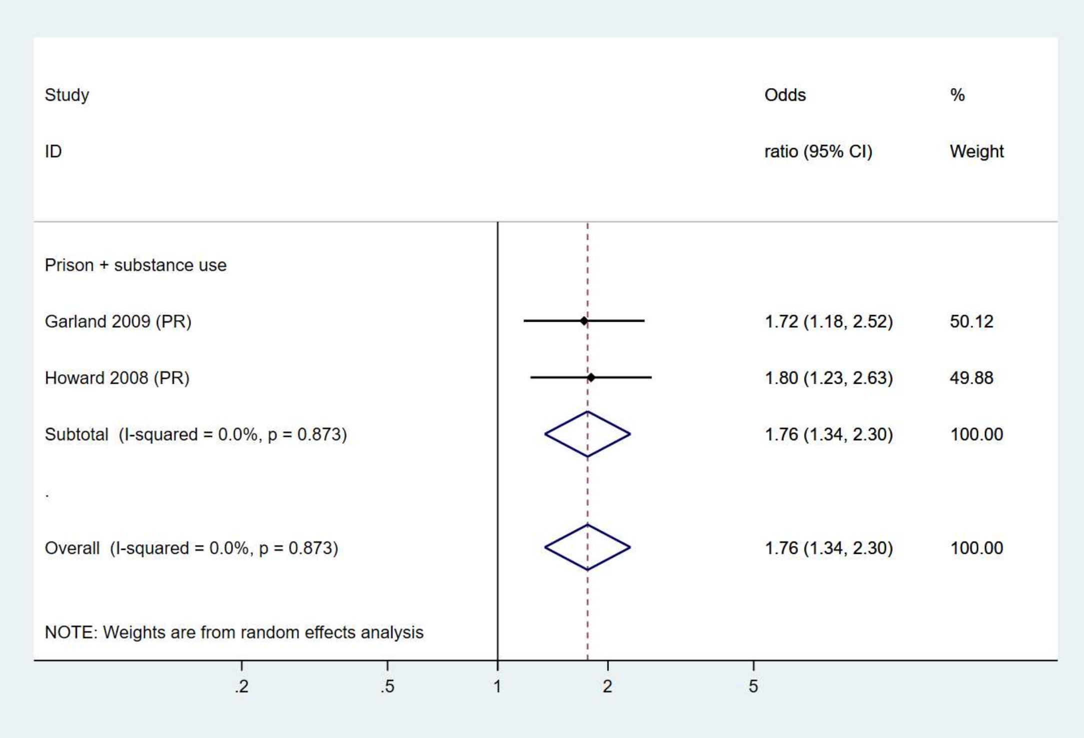

Insufficient data available to permit stratification by gender or risk of bias.

Figure A4.64. Forest plot for meta-analysis of studies reporting lifetime prevalence of brain injury among people with multiple versus fewer exposures, by exposure combination

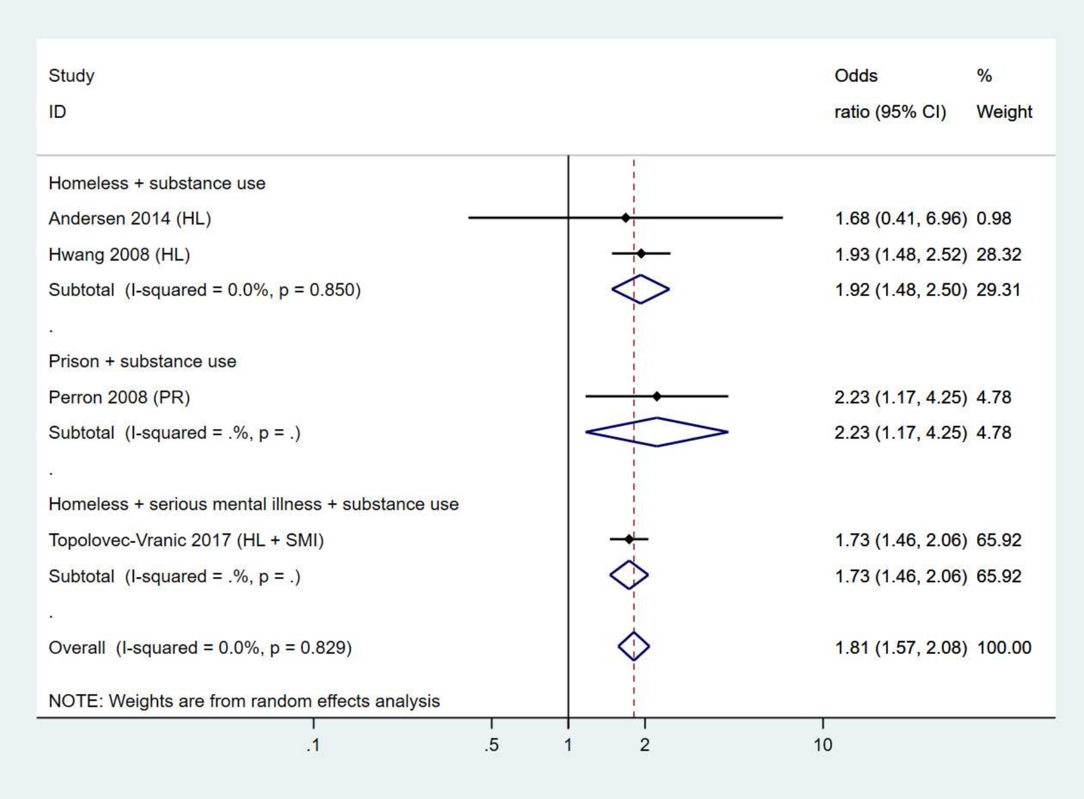

Figure A4.65. Forest plot for meta-analysis of studies reporting lifetime prevalence of brain injury among people with multiple versus fewer exposures, by risk of bias

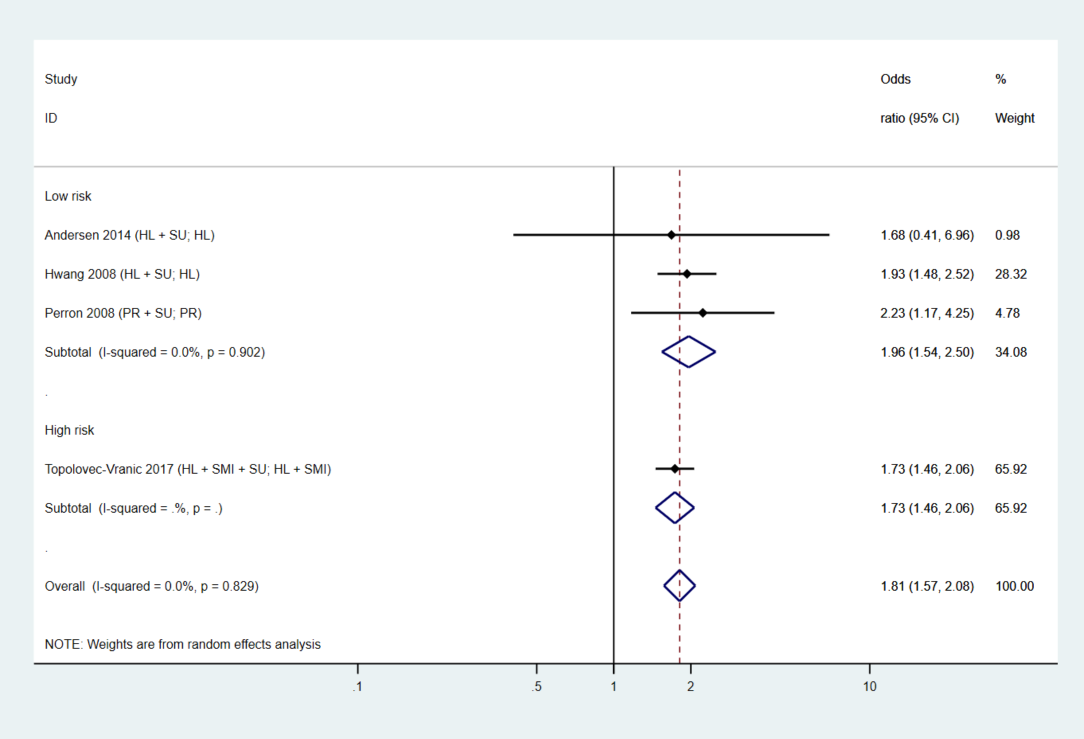

Insufficient data available to permit stratification by gender.

Figure A4.66. Forest plot for meta-analysis of studies reporting hazard ratios for injury mortality among people with multiple versus fewer exposures, by exposure combination

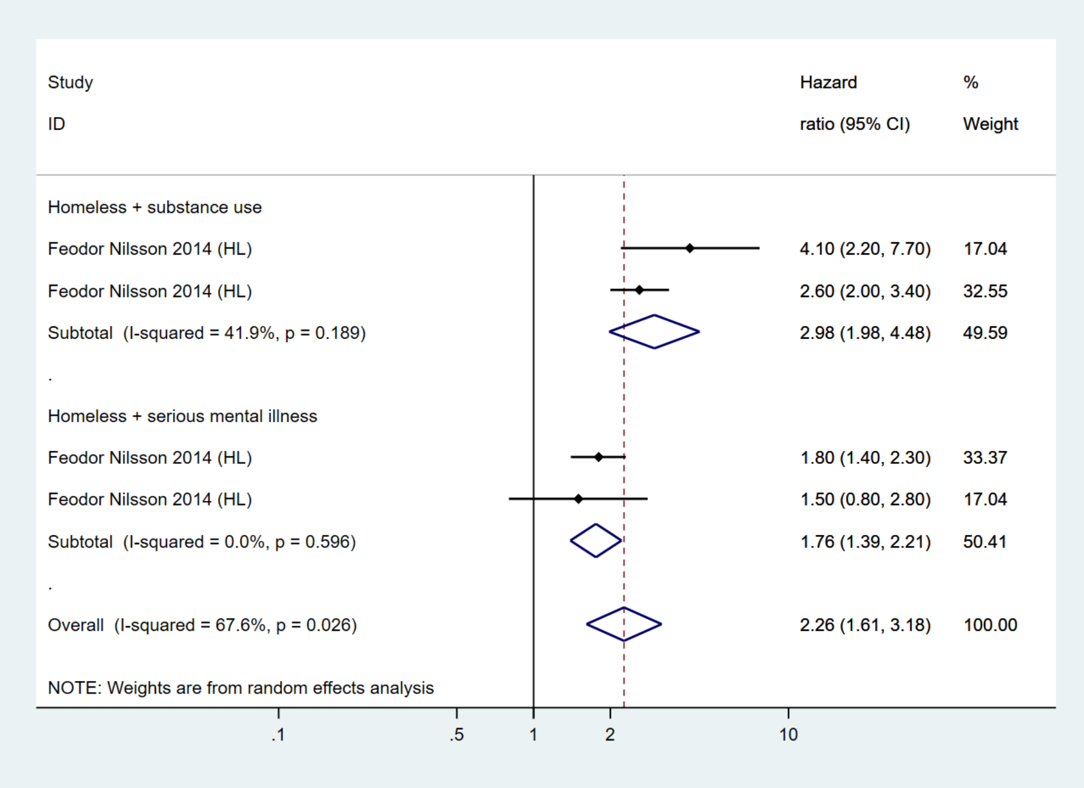

Figure A4.67. Forest plot for meta-analysis of studies reporting injury mortality among people with multiple versus fewer exposures, by gender (where reported separately)

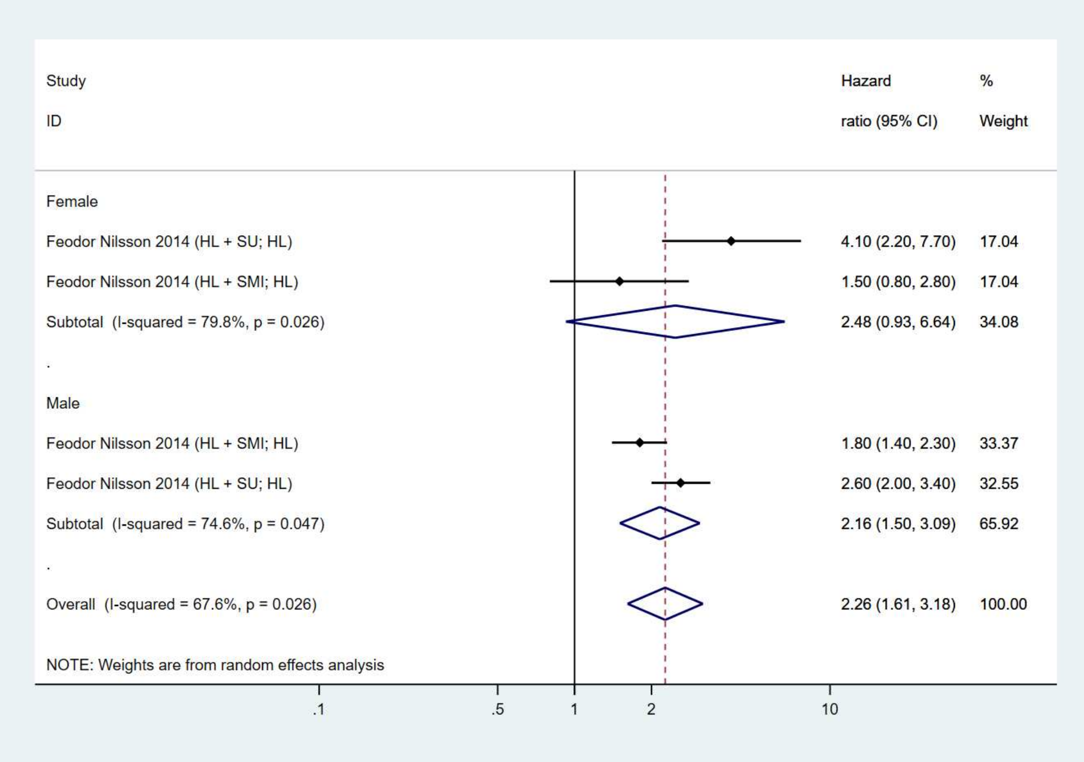

Insufficient data available to permit stratification by risk of bias.

Figure A4.68. Forest plot for meta-analysis of studies reporting lifetime prevalence of deliberate self-harm among people with multiple versus fewer exposures, by exposure combination

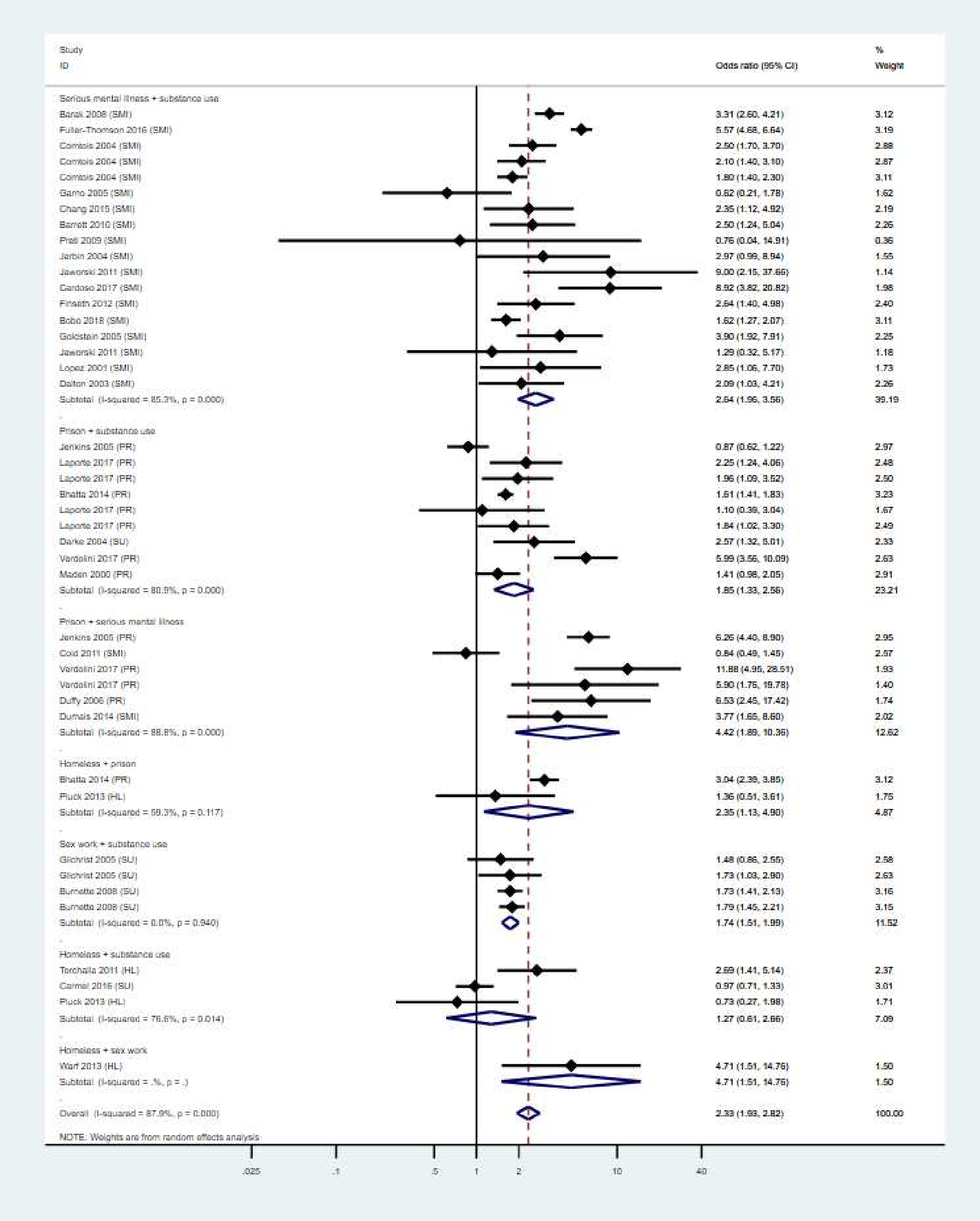

Figure A4.69. Forest plot for meta-analysis of studies reporting lifetime prevalence of deliberate self-harm among people with multiple versus fewer exposures, by risk of bias

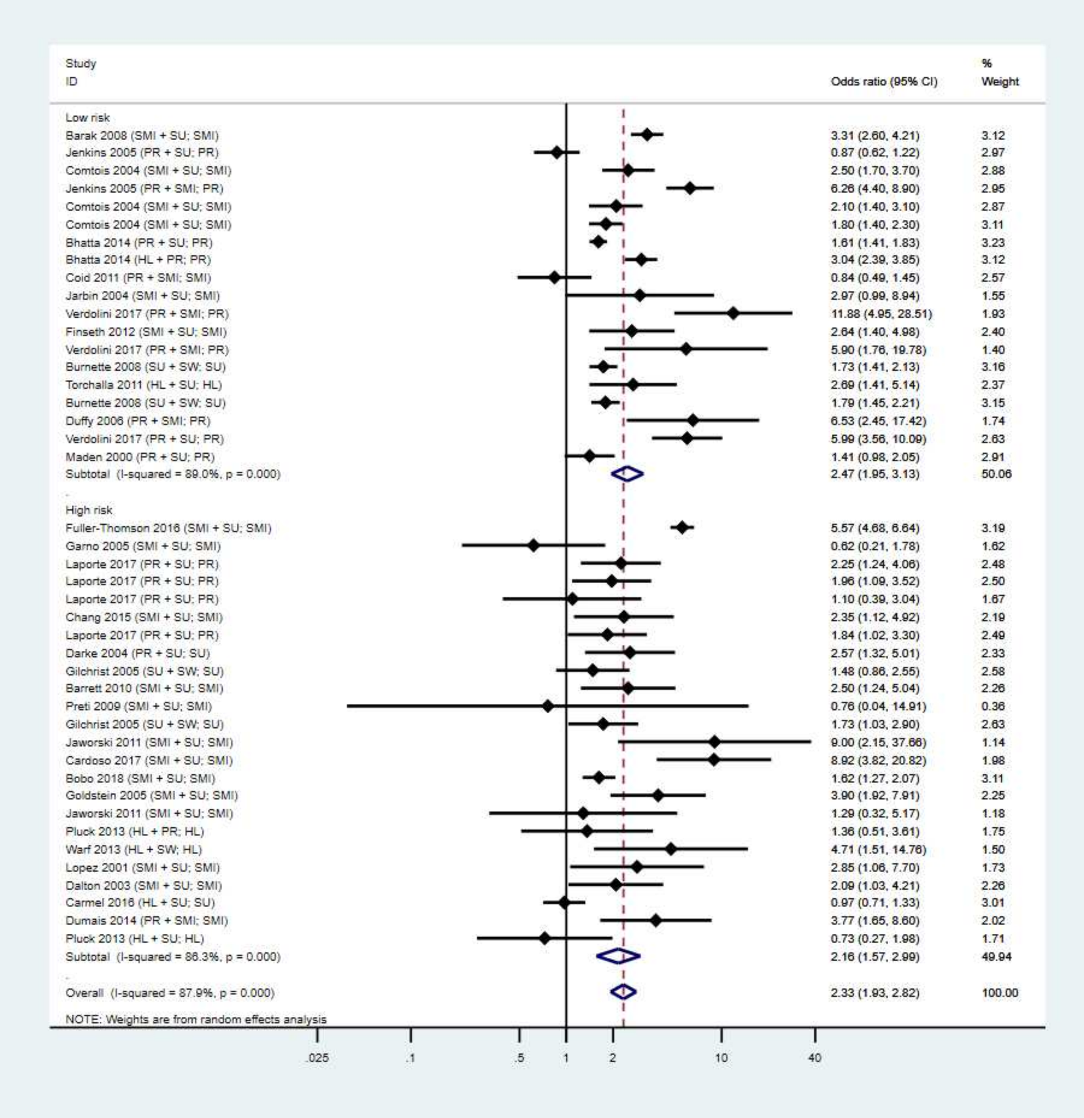

Figure A4.70. Forest plot for meta-analysis of studies reporting lifetime prevalence of deliberate self-harm among people with multiple versus fewer exposures, by gender (where reported separately)

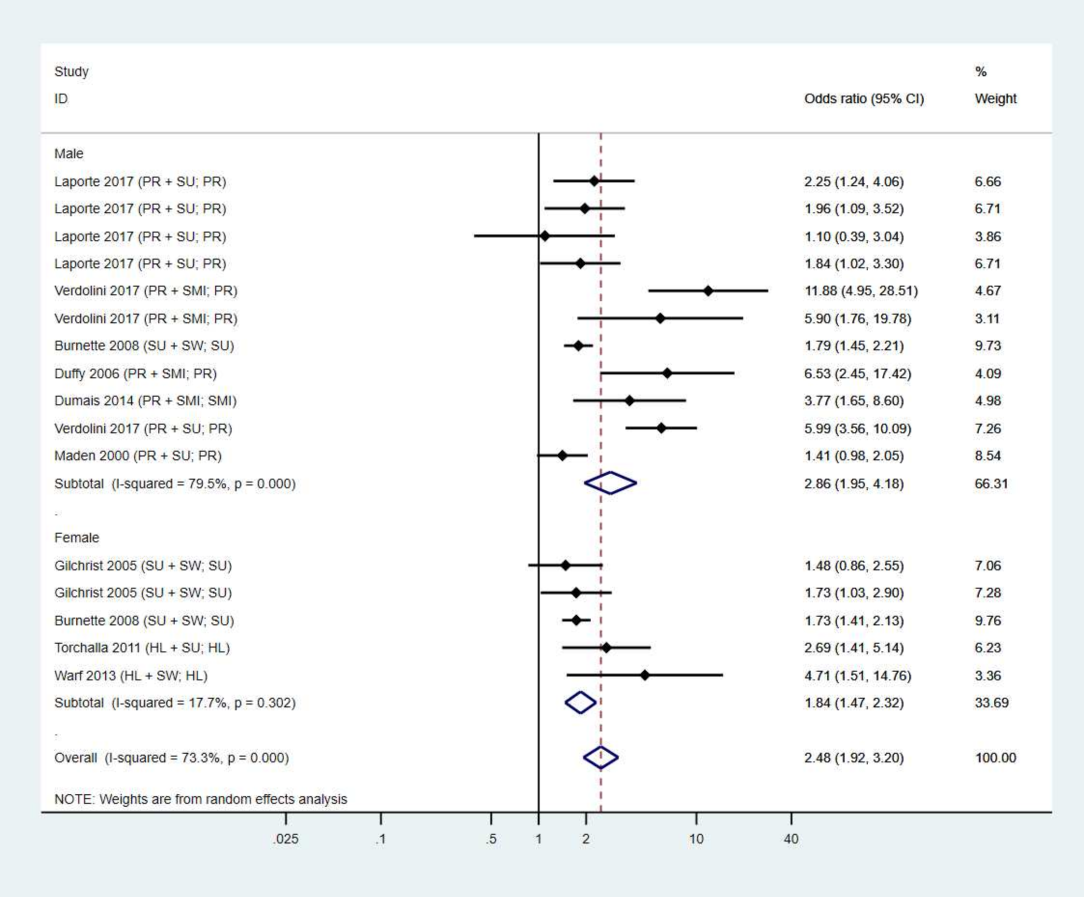

Figure A4.71. Forest plot for meta-analysis of studies reporting past month prevalence of deliberate self-harm among people with multiple versus fewer exposures, by exposure combination

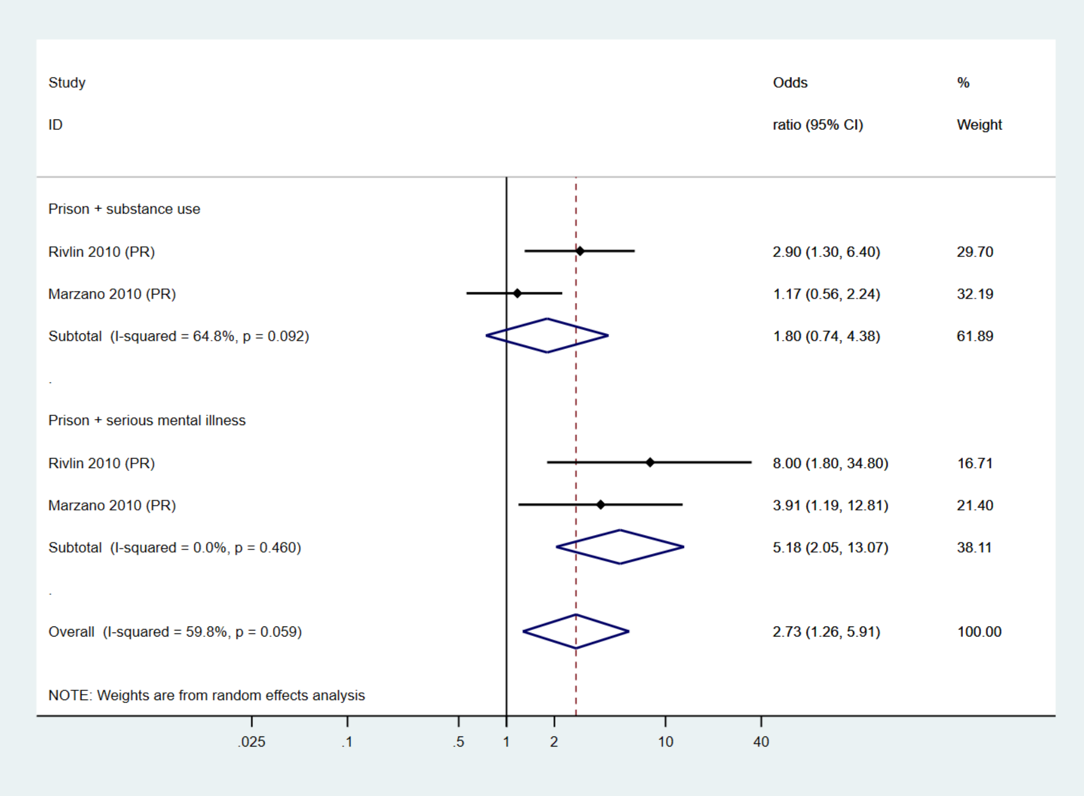

Figure A4.72. Forest plot for meta-analysis of studies reporting past month prevalence of deliberate self-harm among people with multiple versus fewer exposures, by risk of bias

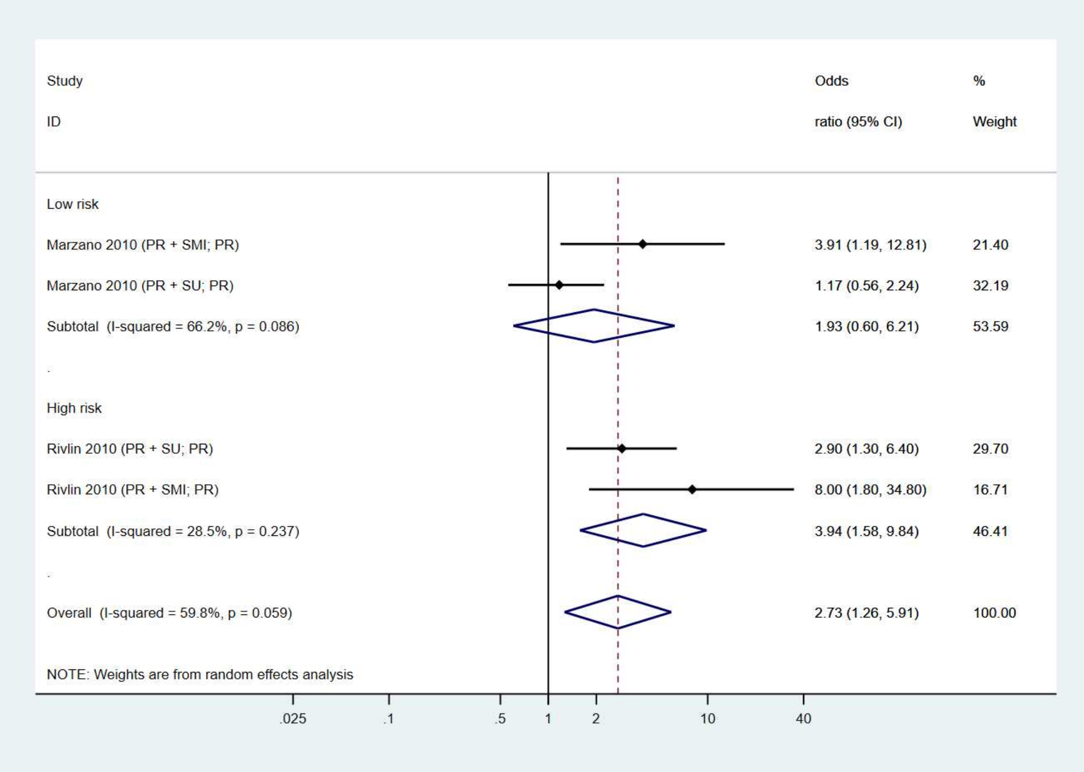

Figure A4.73. Forest plot for meta-analysis of studies reporting past month prevalence of deliberate self-harm among people with multiple versus fewer exposures, by gender (where reported separately)

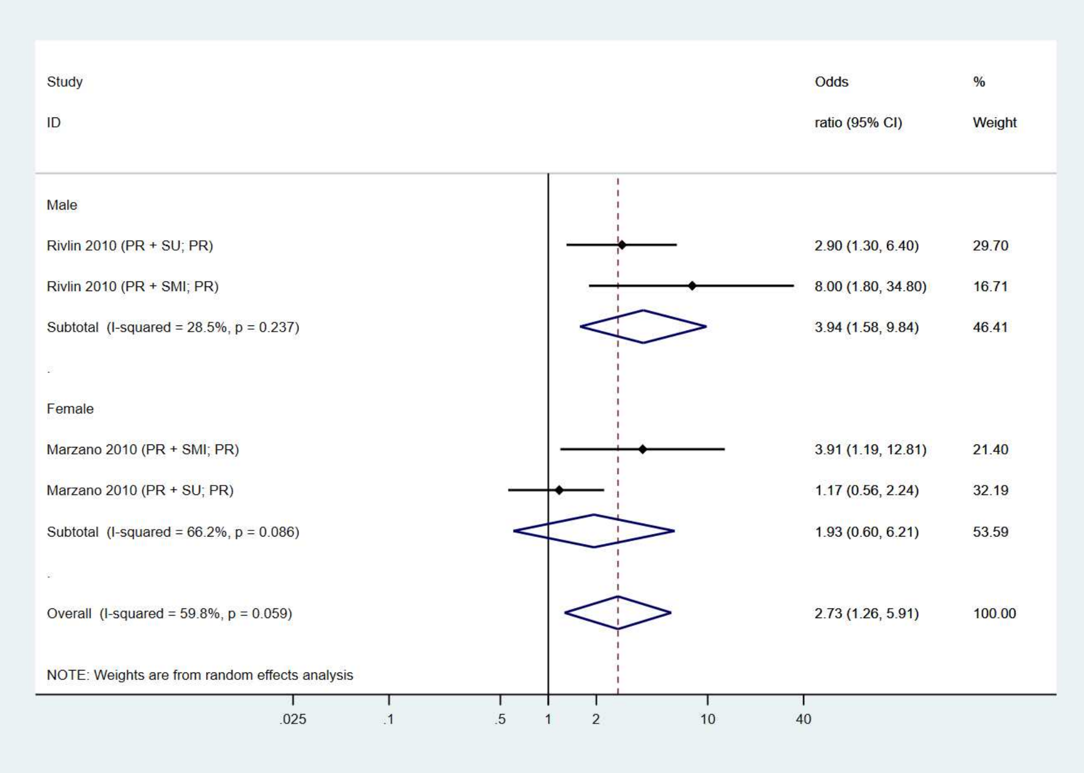

Figure A4.74. Forest plot for meta-analysis of studies reporting subhazard ratios for accident mortality among people with multiple versus fewer exposures, by exposure combination

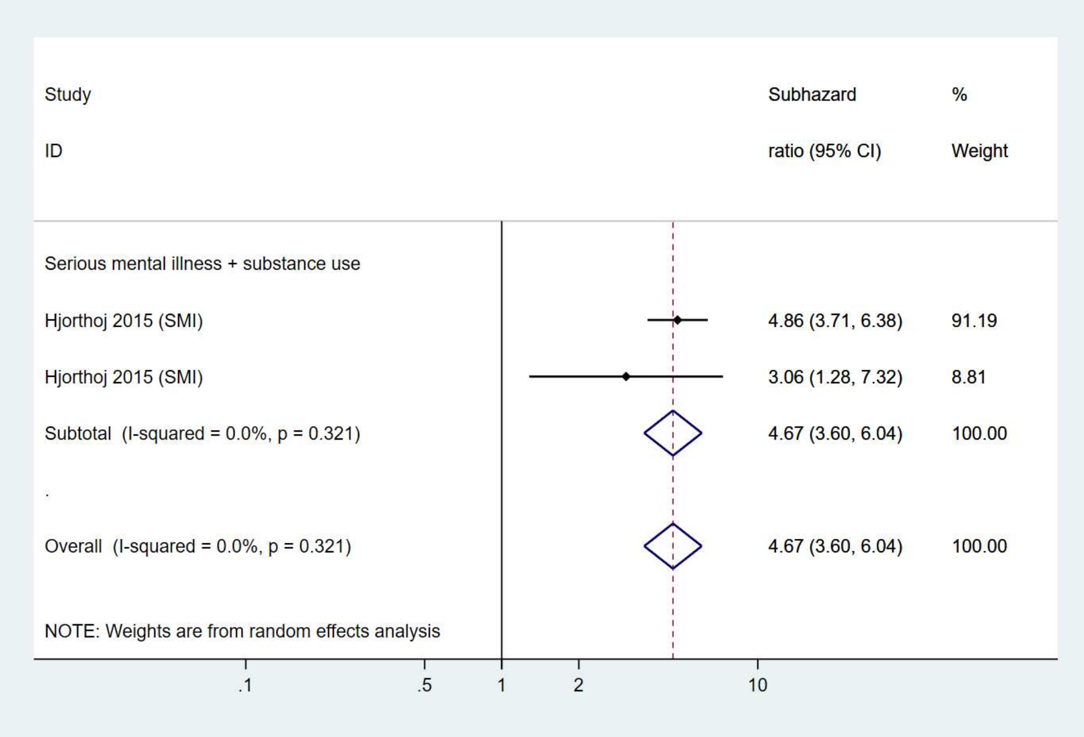

Insufficient data available to permit stratification by gender or risk of bias.

Figure A4.75. Forest plot for meta-analysis of studies reporting hazard ratios for suicide mortality among people with multiple versus fewer exposures, by exposure combination

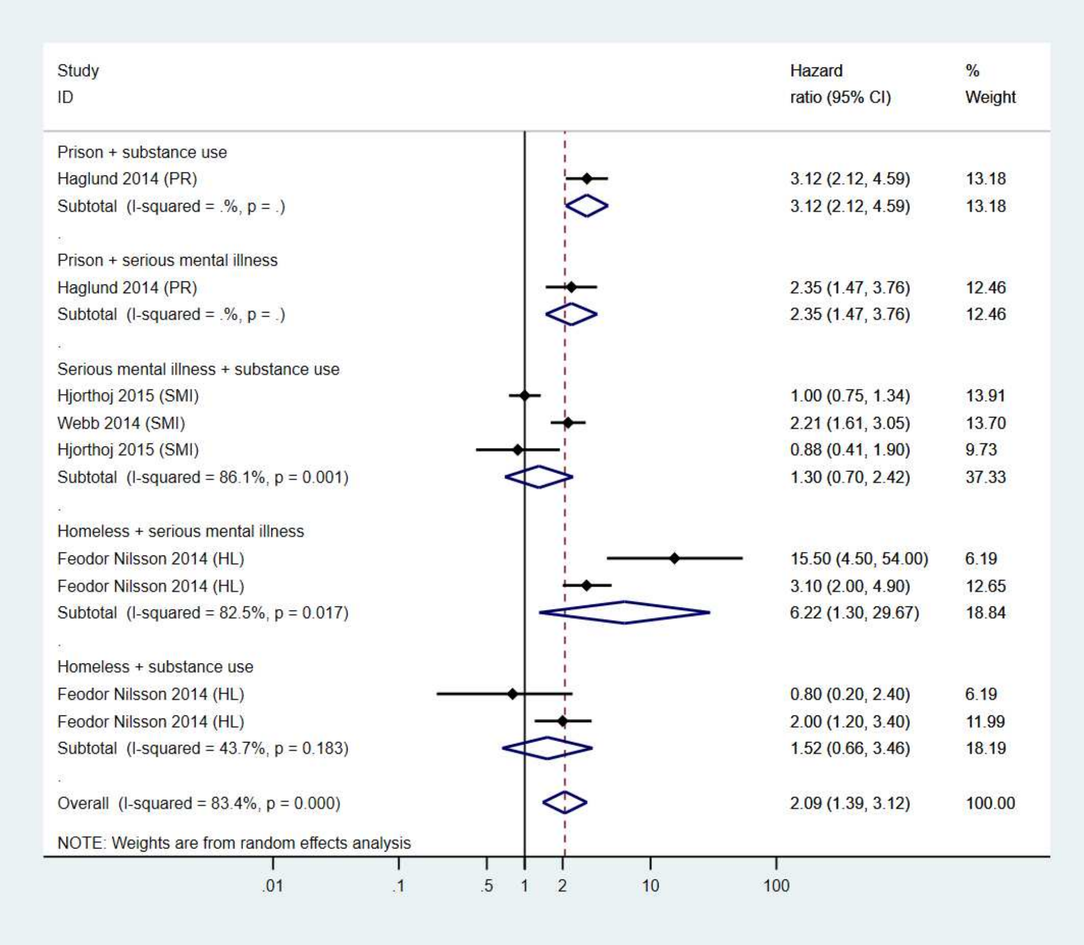

Figure A4.76. Forest plot for meta-analysis of studies reporting hazard ratios for suicide mortality among people with multiple versus fewer exposures, by gender

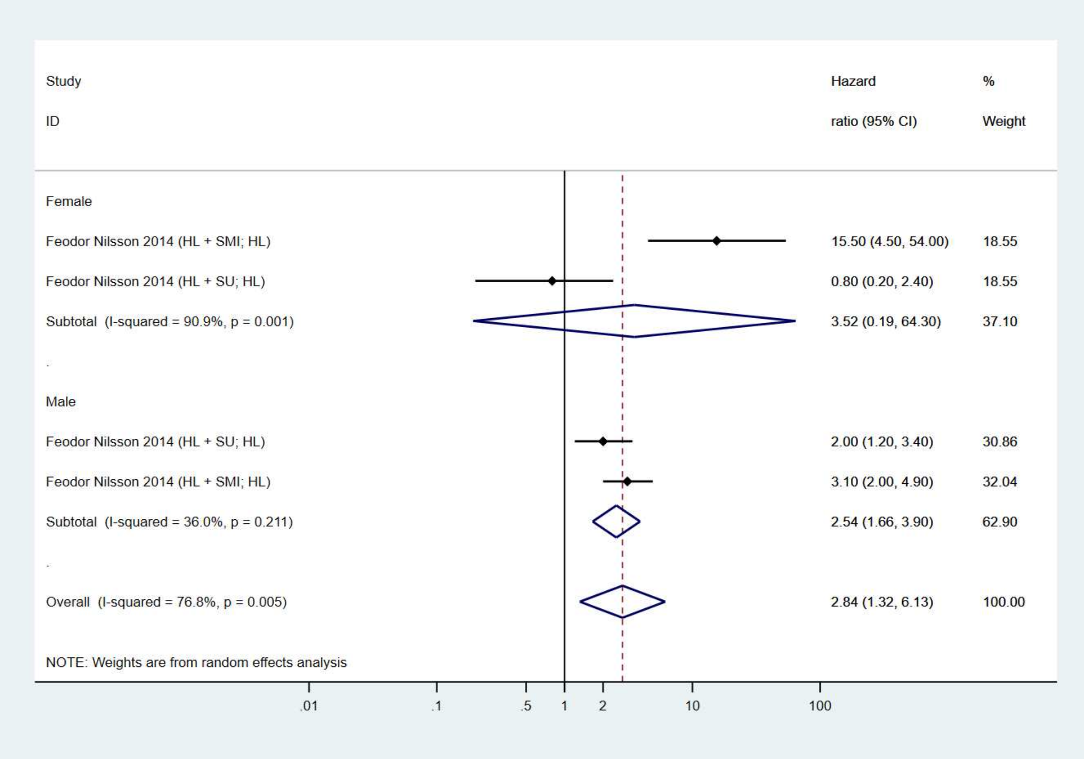

Insufficient data available to permit stratification by risk of bias.

Figure A4.77. Forest plot for meta-analysis of studies reporting odds ratios for suicide mortality among people with multiple versus fewer exposures, by exposure combination

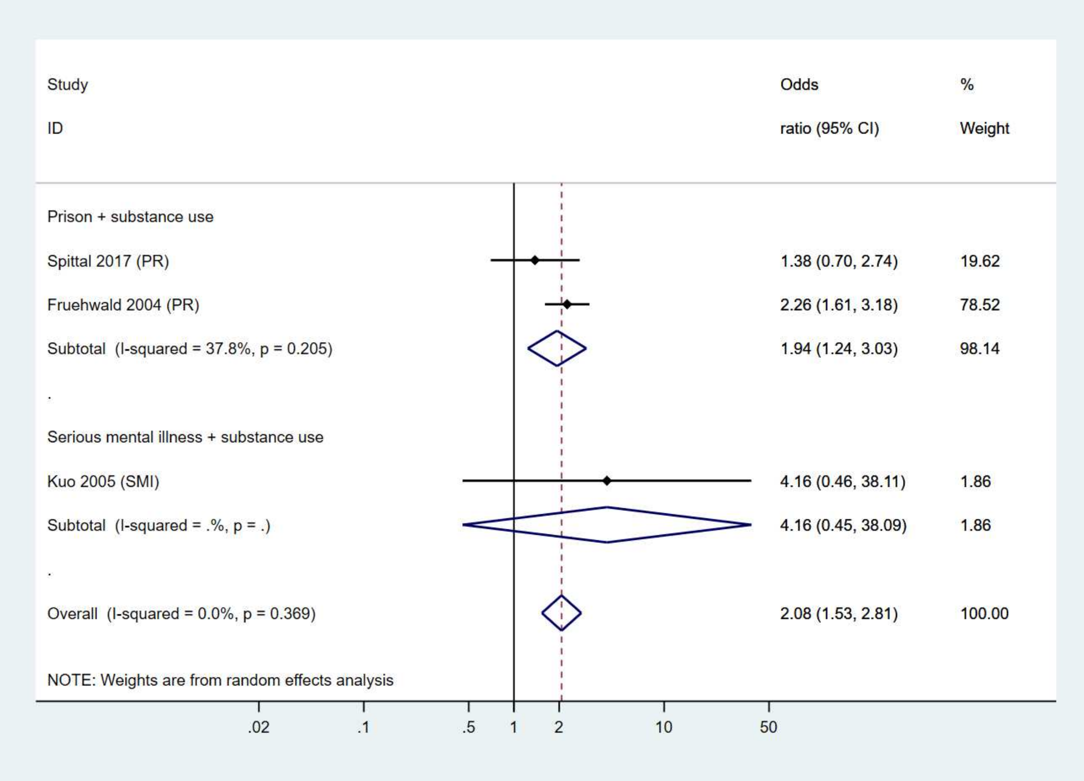

Figure A4.78. Forest plot for meta-analysis of studies reporting odds ratios for suicide mortality among people with multiple versus fewer exposures, by risk of bias

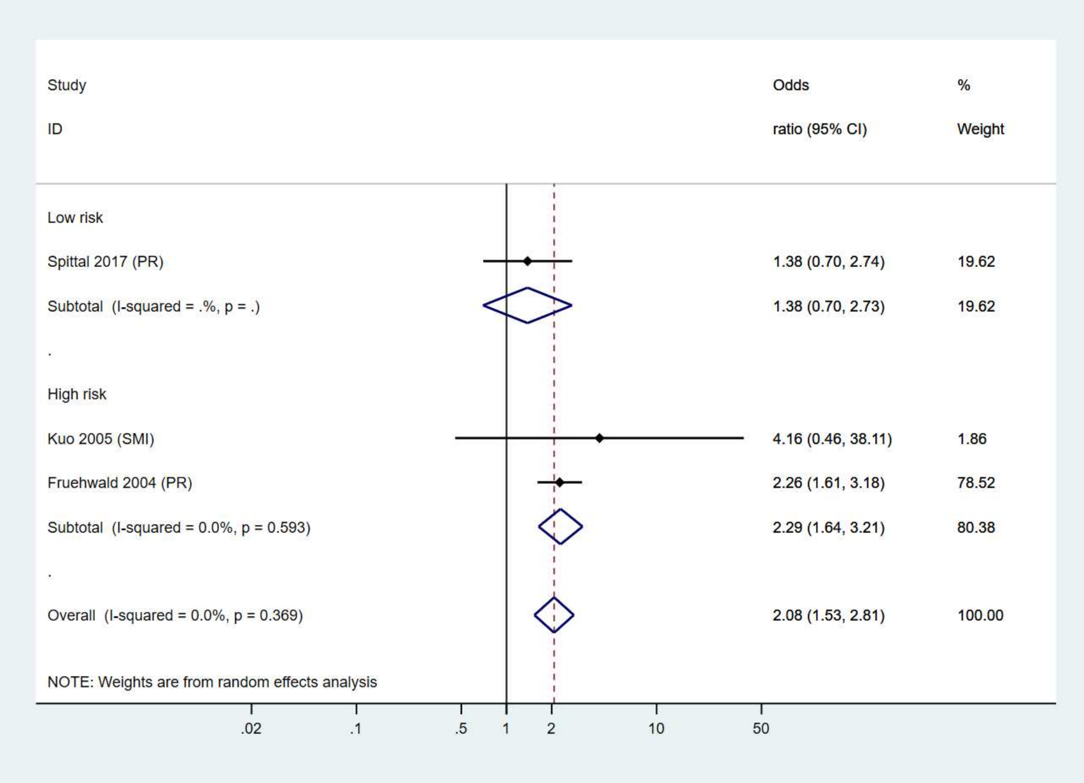

Insufficient data available to permit stratification by gender.

Figure A4.79. Forest plot for meta-analysis of studies reporting incidence rate ratios for suicide mortality among people with multiple versus fewer exposures, by exposure combination

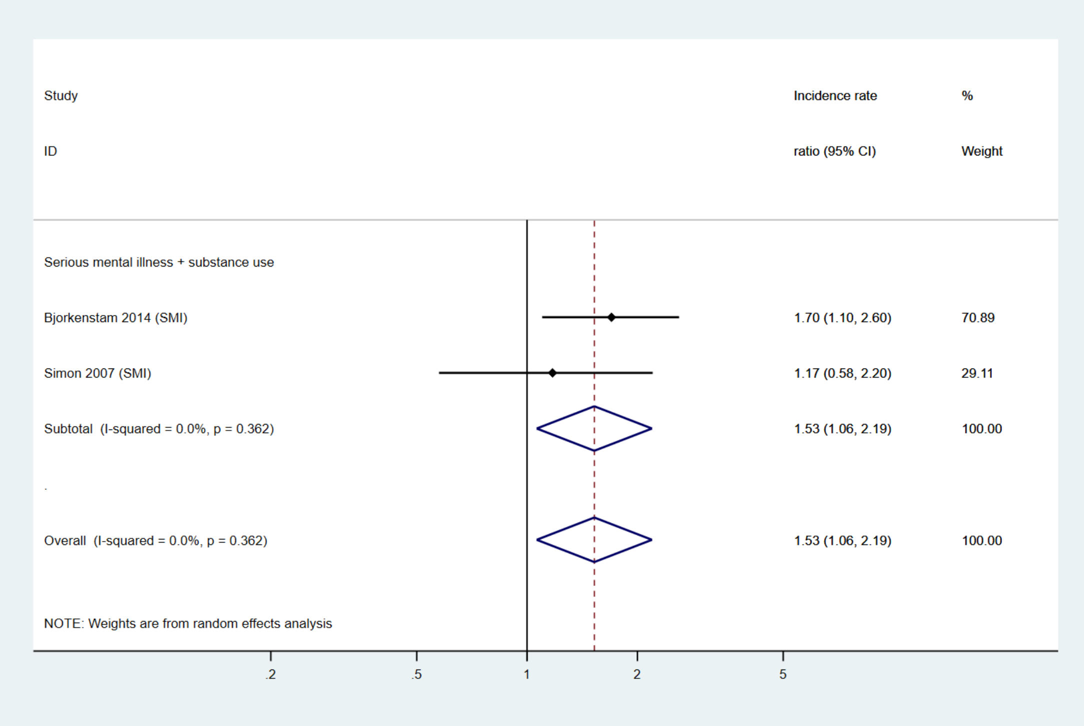

Insufficient data available to permit stratification by gender or risk of bias.

Figure A4.80. Forest plot for meta-analysis of studies reporting hazard ratios for all causes of external mortality (ICD-10 chapters 19 and 20) among people with multiple versus fewer exposures, by exposure combination

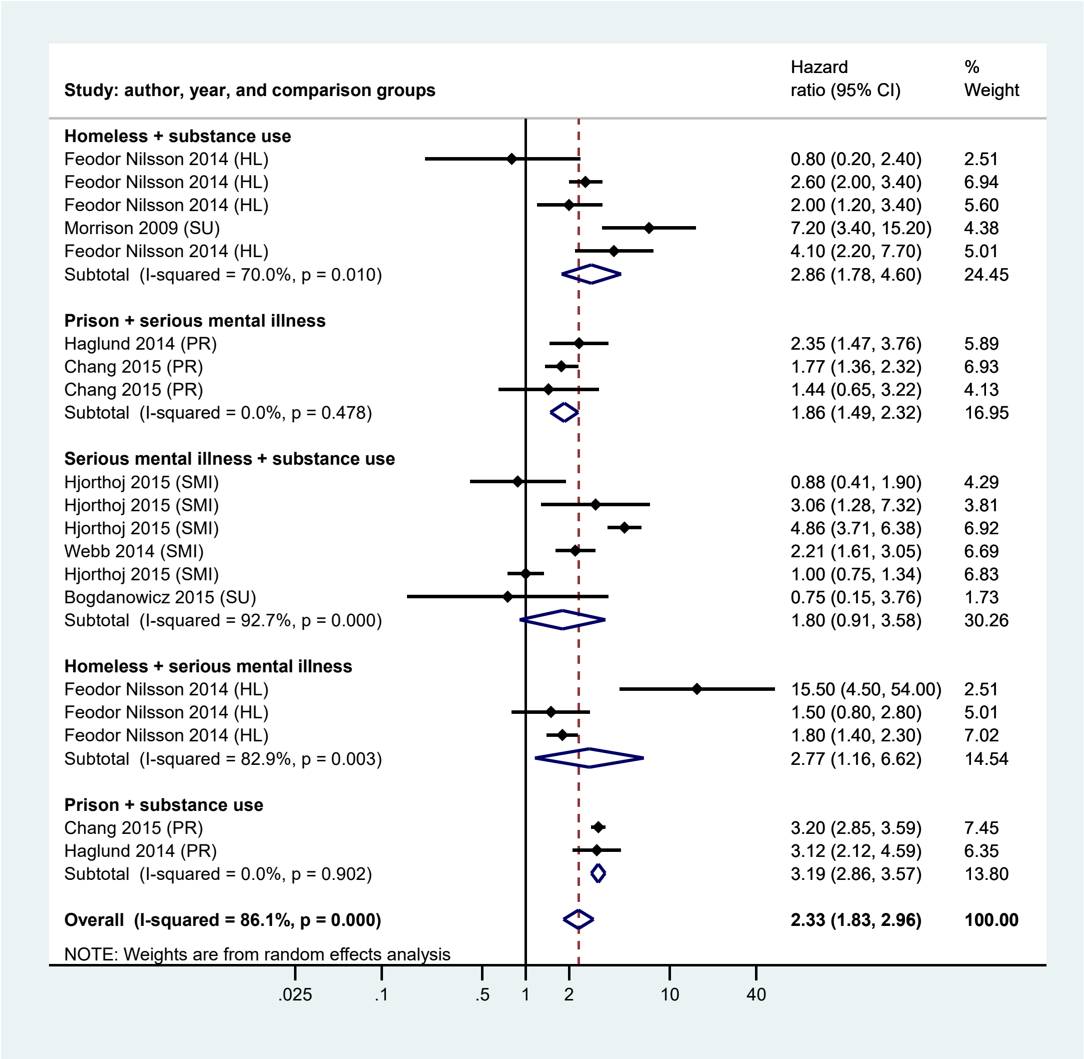

Figure A4.81. Forest plot for meta-analysis of studies reporting hazard ratios for all causes of external mortality (ICD-10 chapters 19 and 20) among people with multiple versus fewer exposures, by risk of bias

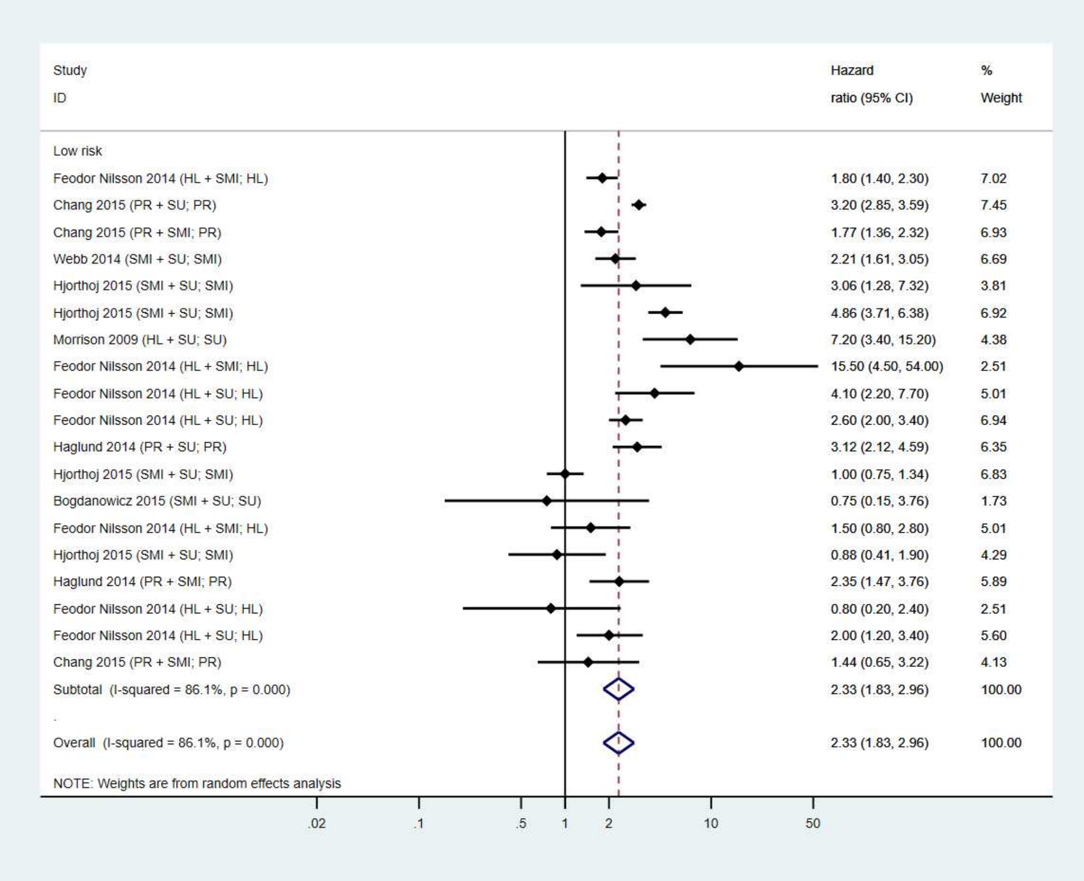

Figure A4.82. Forest plot for meta-analysis of studies reporting hazard ratios for all causes of external mortality (ICD-10 chapters 19 and 20) among people with multiple versus fewer exposures, by gender (where reported separately)

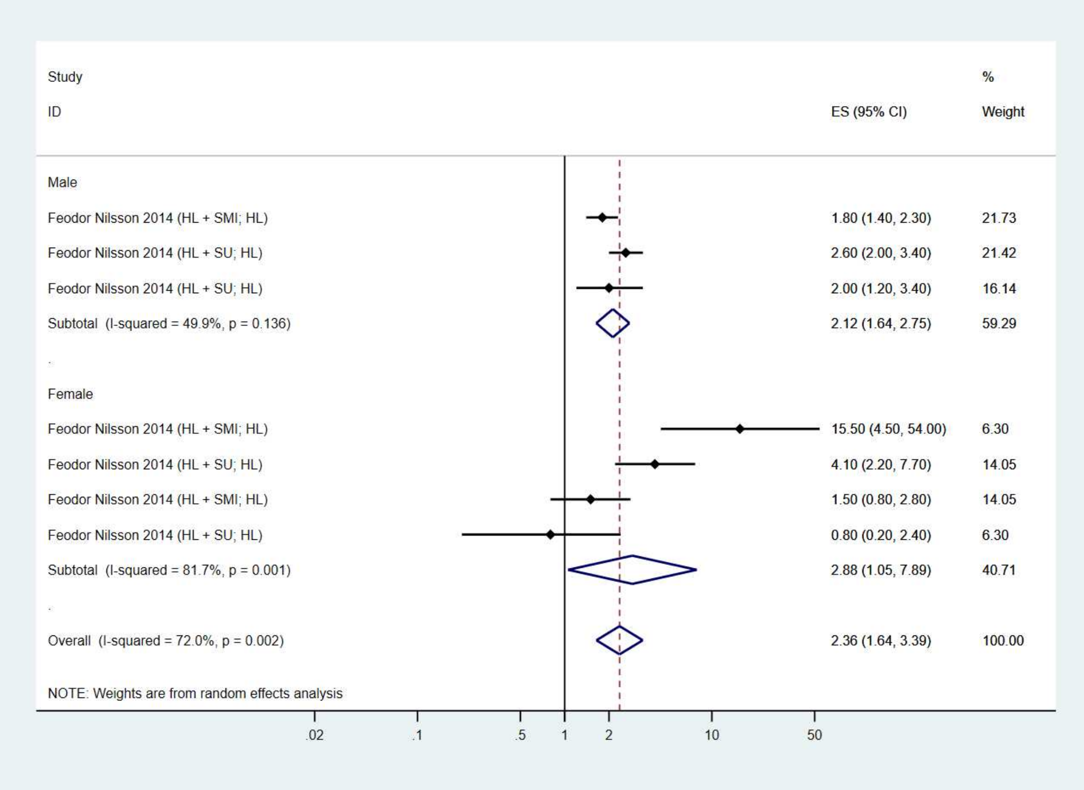

Figure A4.83. Forest plot for meta-analysis of studies reporting odds ratios for all causes of external mortality (ICD-10 chapters 19 and 20) among people with multiple versus fewer exposures, by exposure combination

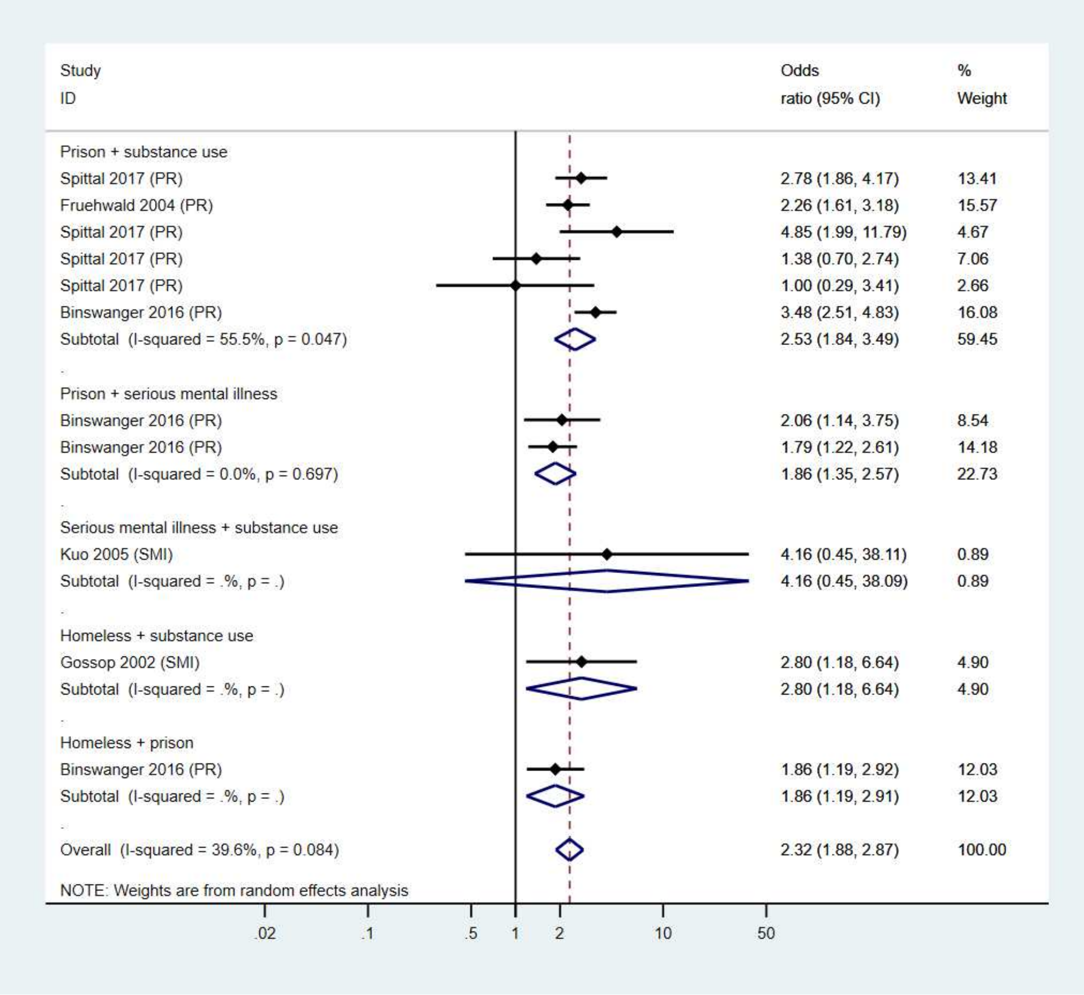

Figure A4.84. Forest plot for meta-analysis of studies reporting odds ratios for all causes of external mortality (ICD-10 chapters 19 and 20) among people with multiple versus fewer exposures, by risk of bias

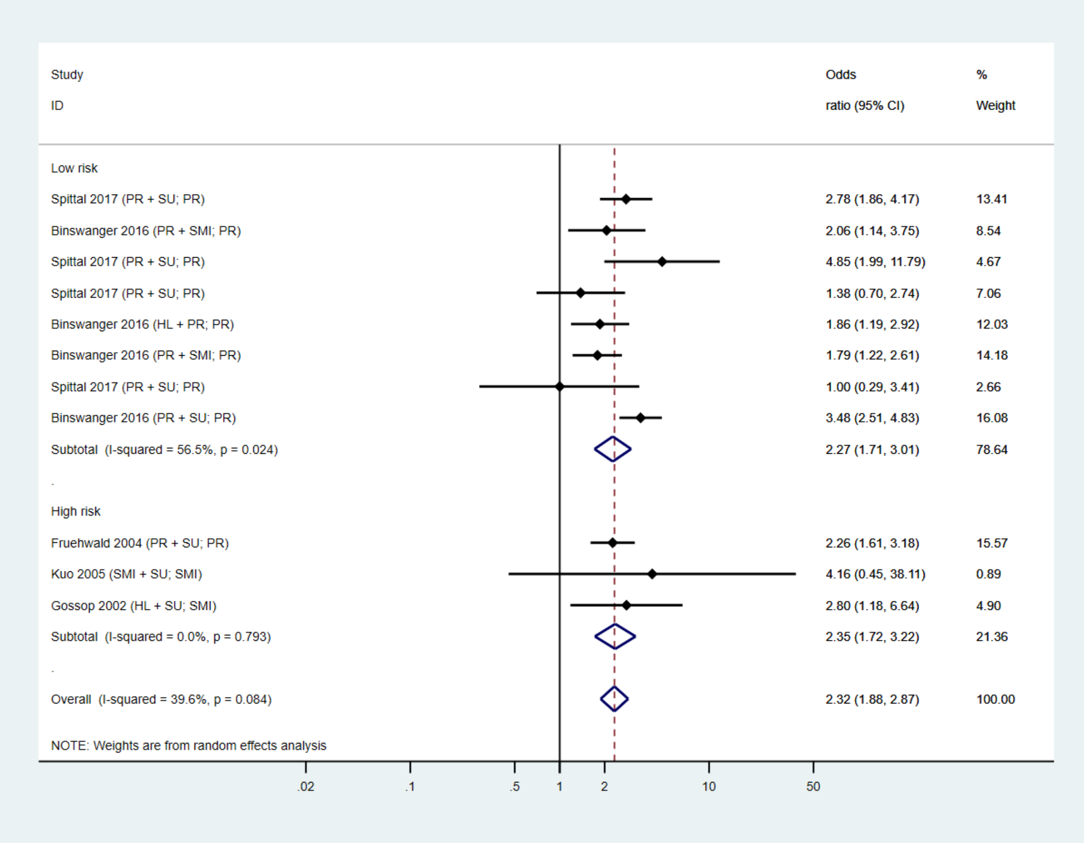

Figure A4.85. Forest plot for meta-analysis of studies reporting rate ratios for all causes of external mortality (ICD-10 chapters 19 and 20) among people with multiple versus fewer exposures, by exposure combination

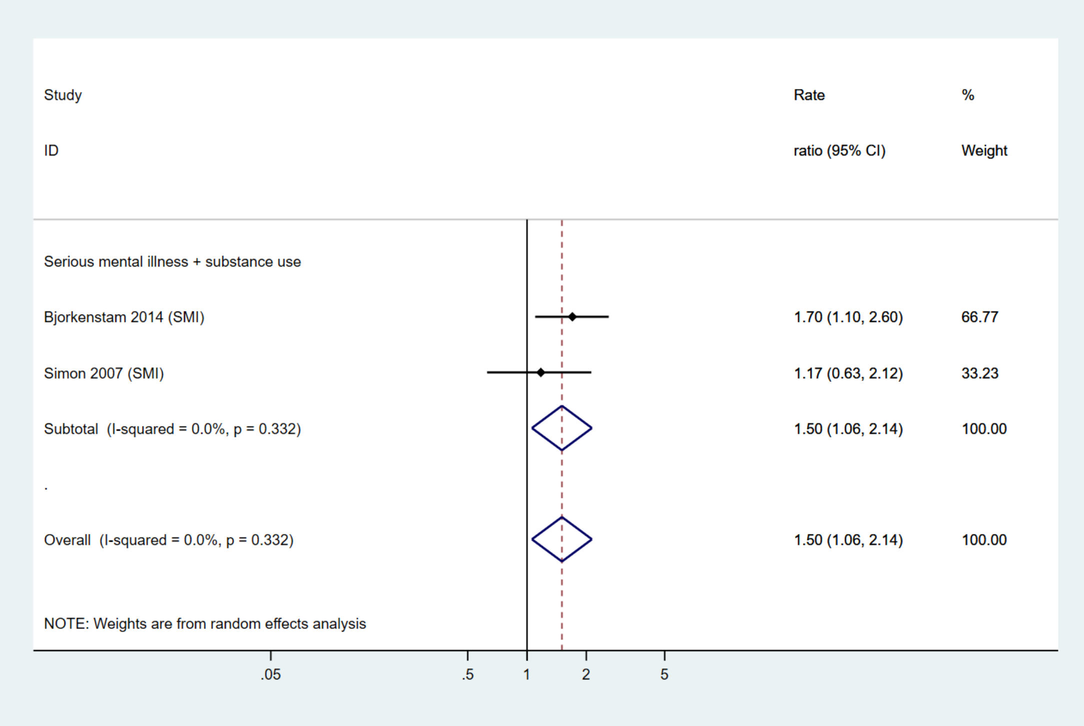

Figure A4.86. Forest plot for meta-analysis of studies reporting rate ratios for all causes of external mortality (ICD-10 chapters 19 and 20) among people with multiple versus fewer exposures, by risk of bias

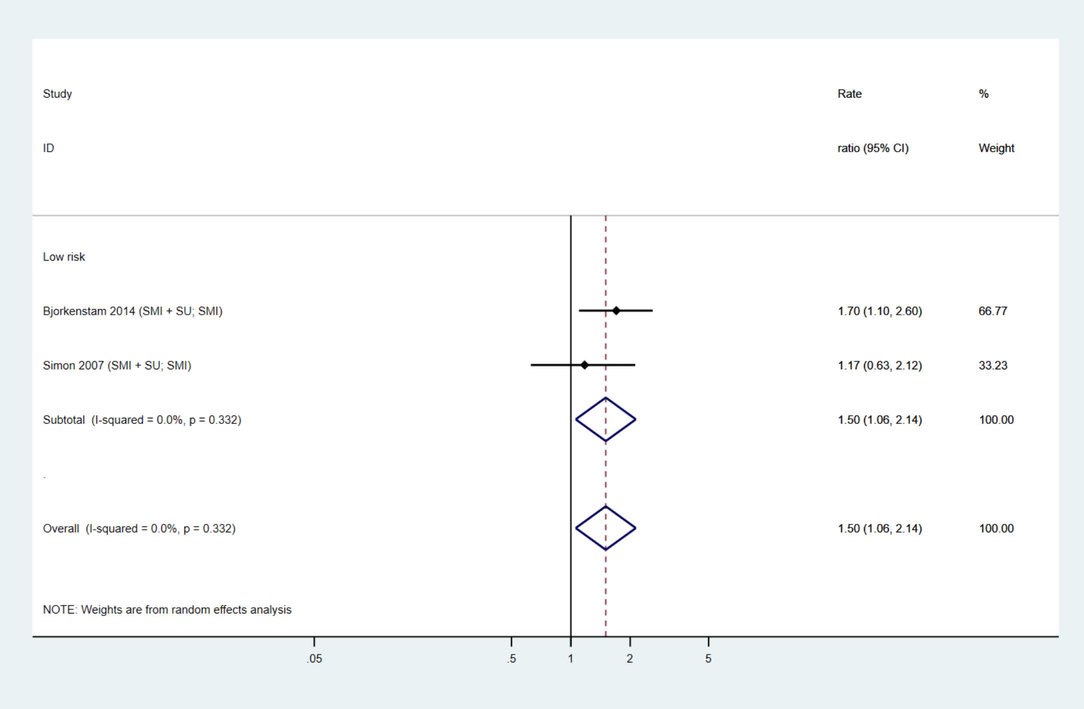

Figure A4.87. Forest plot for meta-analysis of studies reporting risk ratios for all causes of external mortality (ICD-10 chapters 19 and 20) among people with multiple versus fewer exposures, by exposure combination

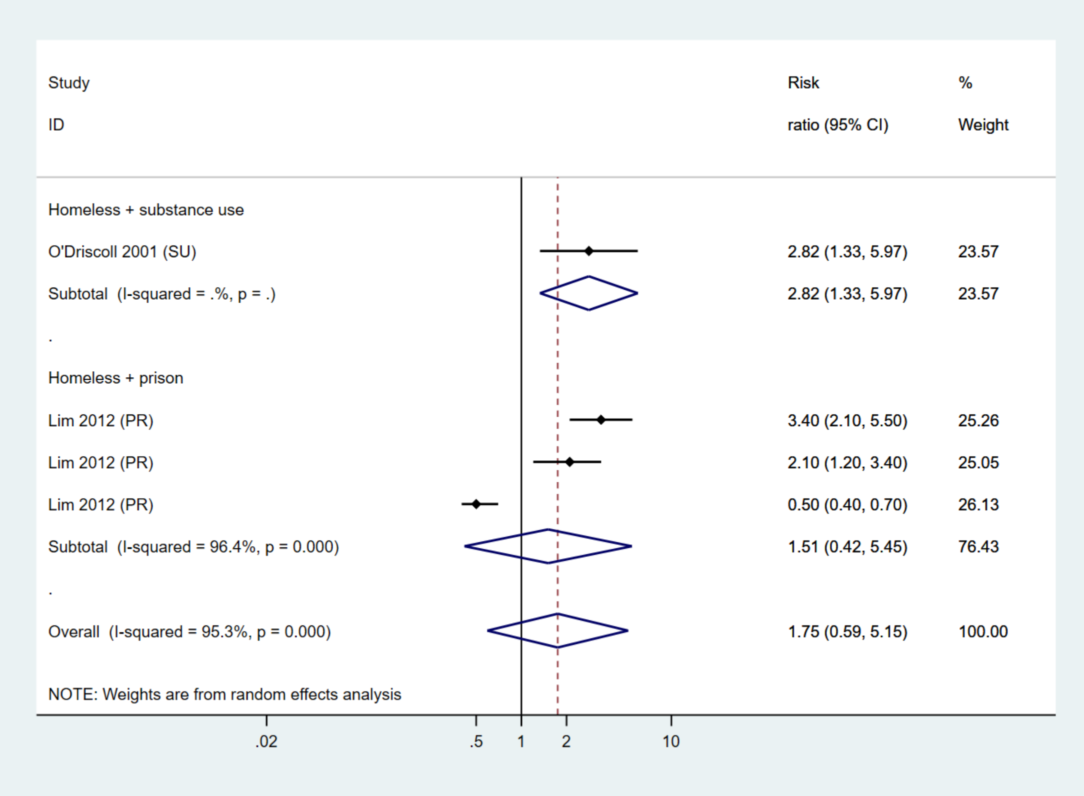

Figure A4.88. Forest plot for meta-analysis of studies reporting risk ratios for all causes of external mortality (ICD-10 chapters 19 and 20) among people with multiple versus fewer exposures, by risk of bias

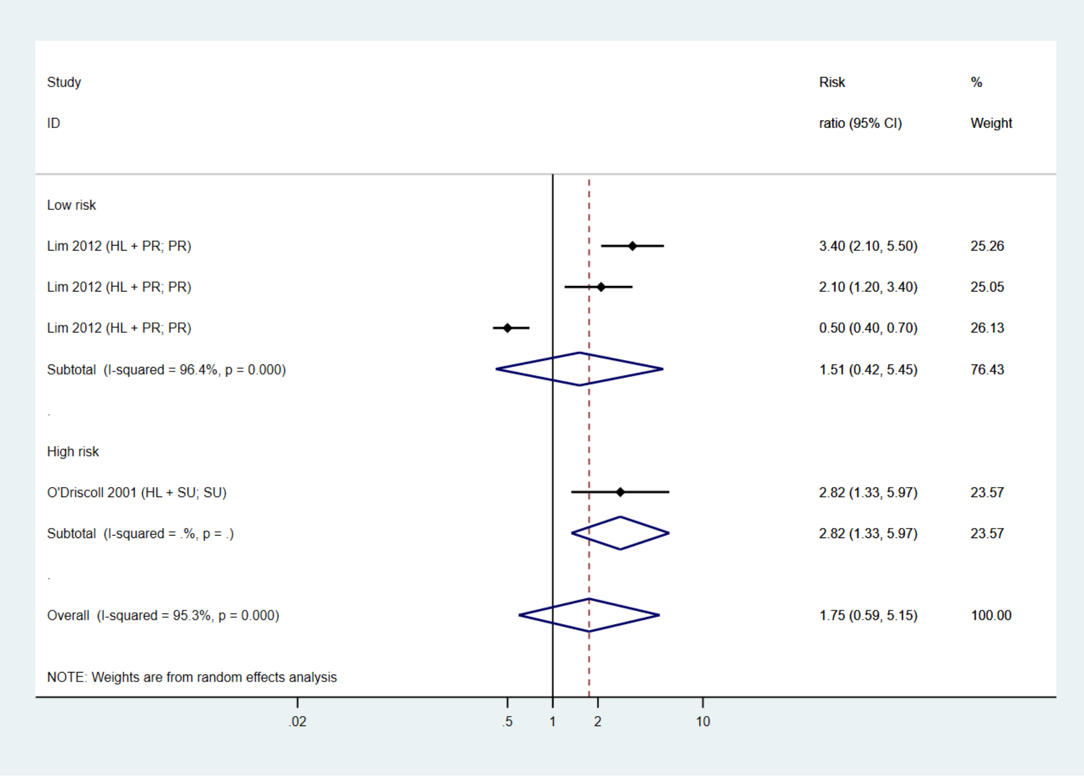

Figure A4.89. Forest plot for meta-analysis of studies reporting lifetime prevalence of selected non-communicable diseases among people with multiple versus fewer exposures, by exposure combination

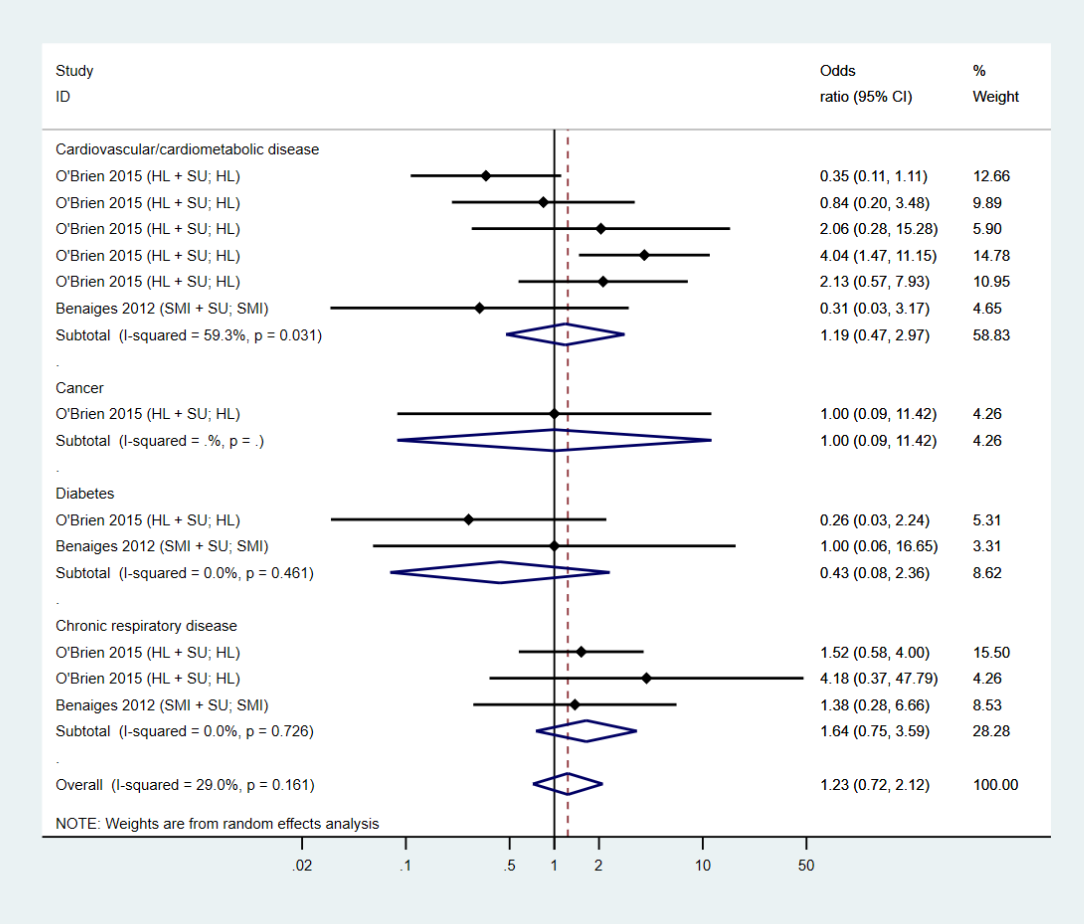

Figure A4.90. Forest plot for meta-analysis of studies reporting lifetime prevalence of selected non-communicable diseases among people with multiple versus fewer exposures, by risk of bias

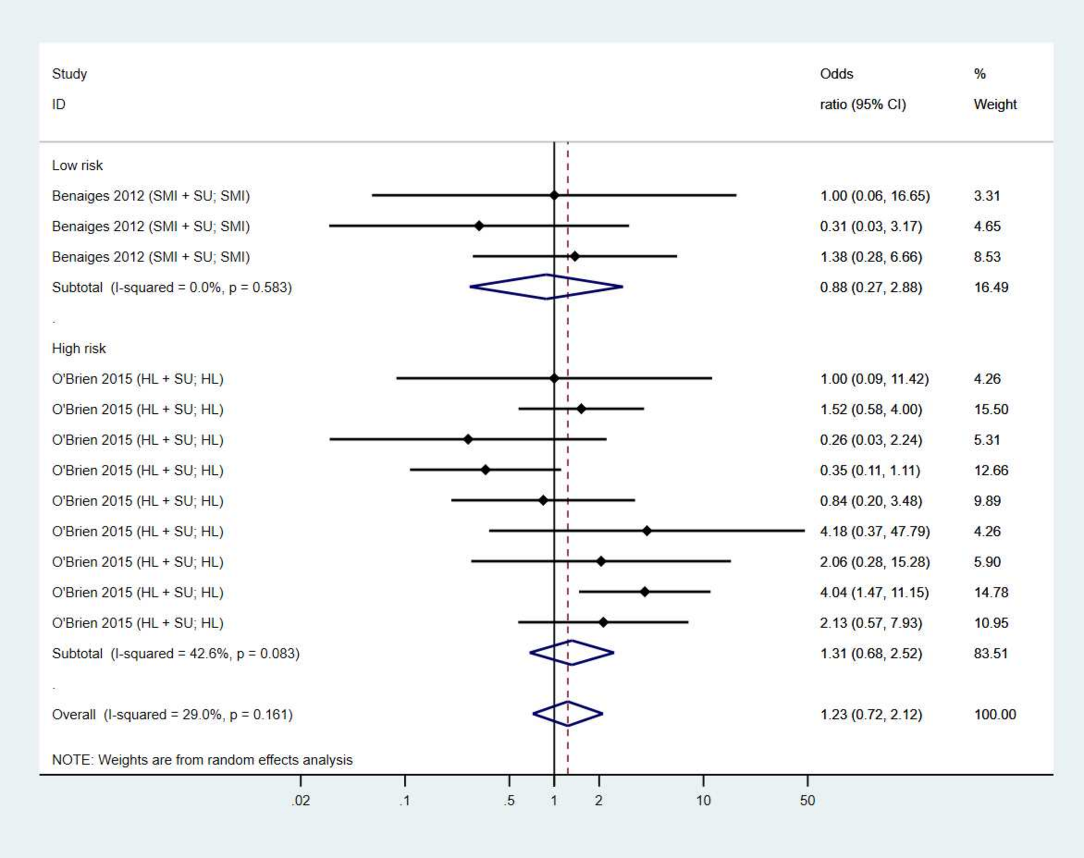

Figure A4.91. Forest plot for meta-analysis of studies reporting past year prevalence of selected non-communicable diseases among people with multiple versus fewer exposures, by exposure combination

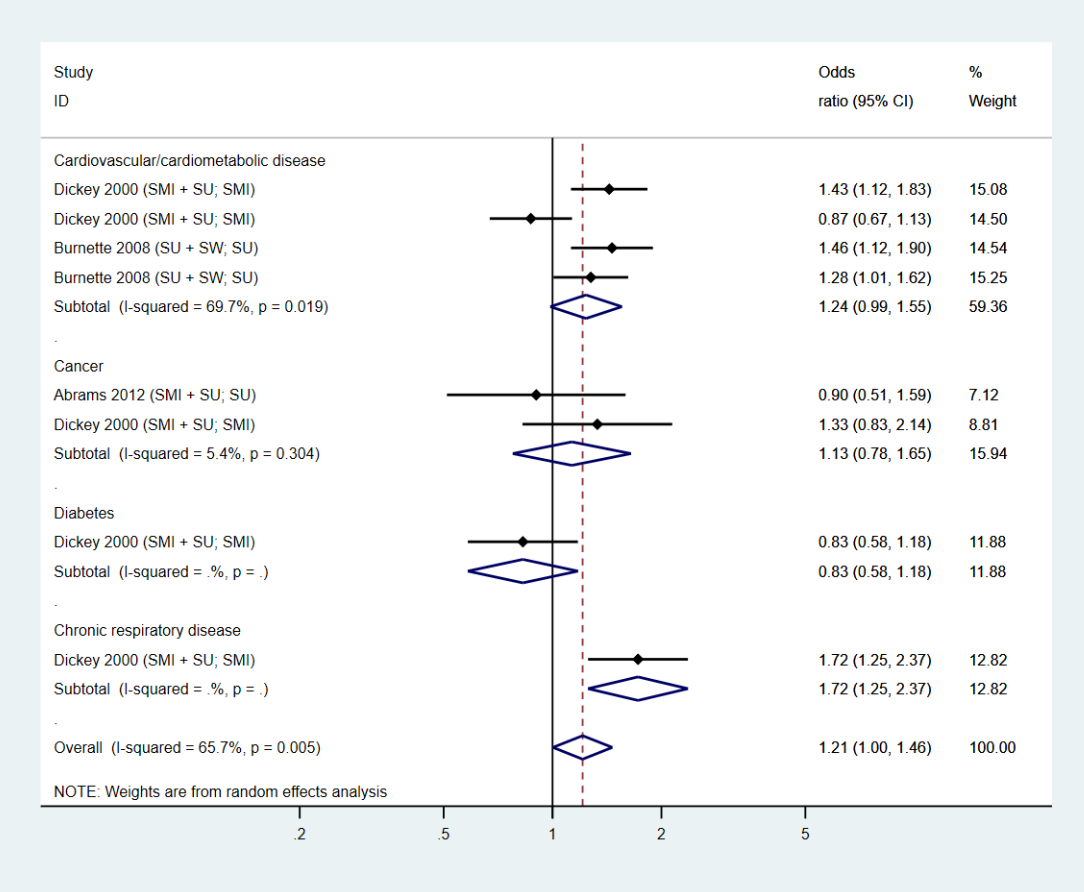

Figure A4.92. Forest plot for meta-analysis of studies reporting past year prevalence of selected non-communicable diseases among people with multiple versus fewer exposures, by gender (where reported separately)

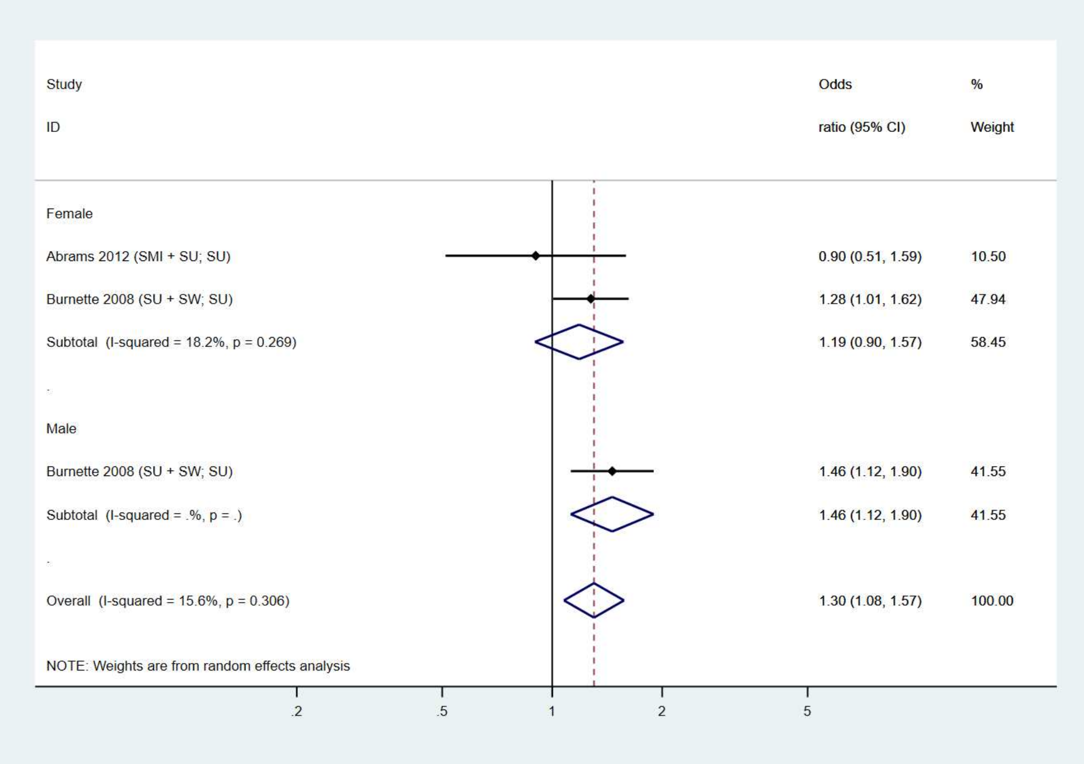

Figure A4.93. Forest plot for meta-analysis of studies reporting current prevalence of selected non-communicable diseases among people with multiple versus fewer exposures, by exposure combination

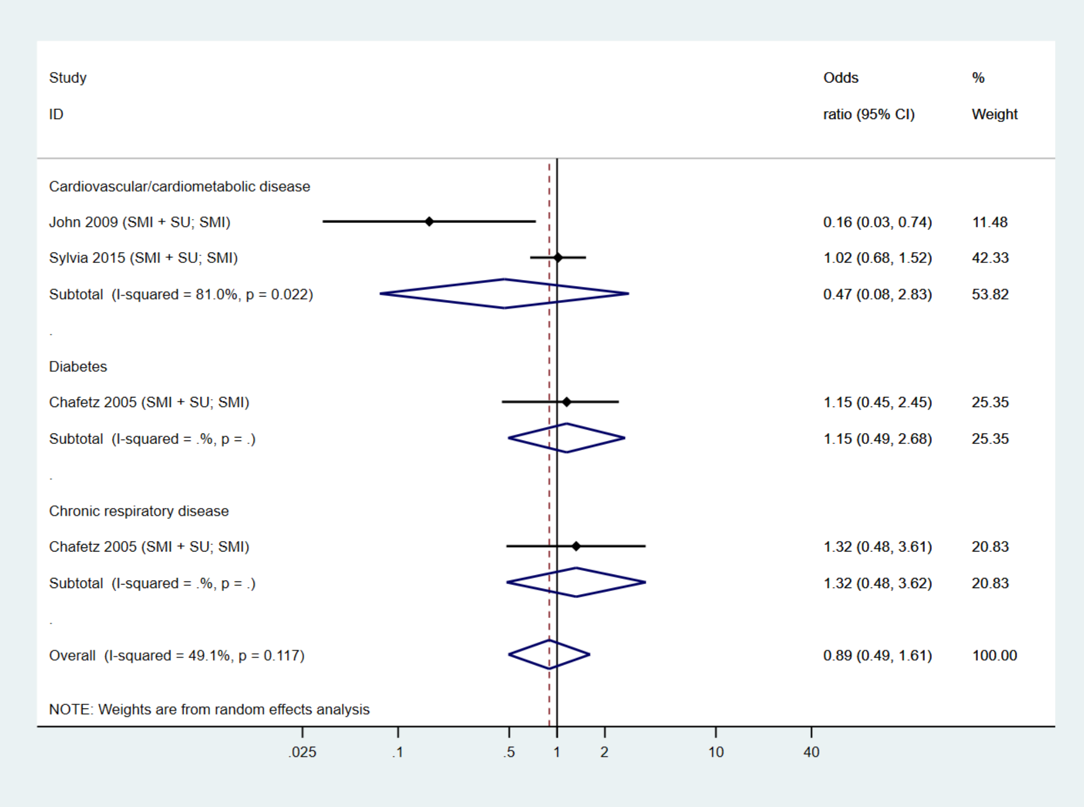

Figure A4.94. Forest plot for meta-analysis of studies reporting current prevalence of selected non-communicable diseases among people with multiple versus fewer exposures, by risk of bias

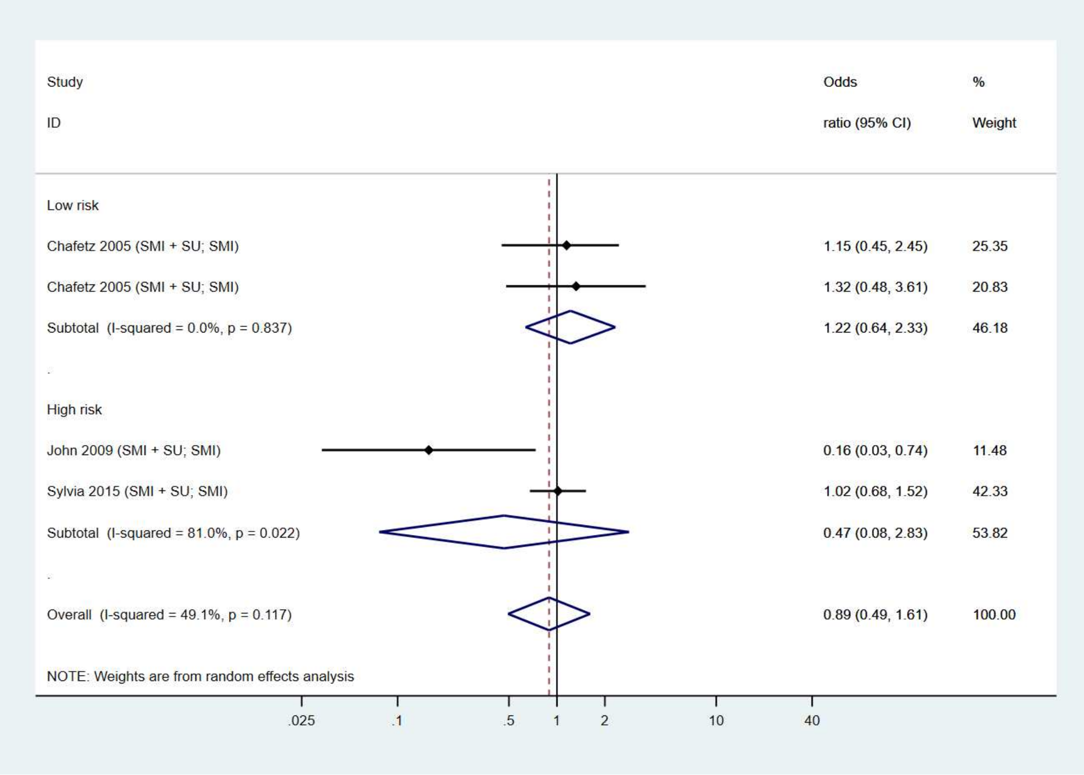

Figure A4.95. Forest plot for meta-analysis of studies reporting hazard ratios for mortality from selected non-communicable diseases among people with multiple versus fewer exposures, by exposure combination

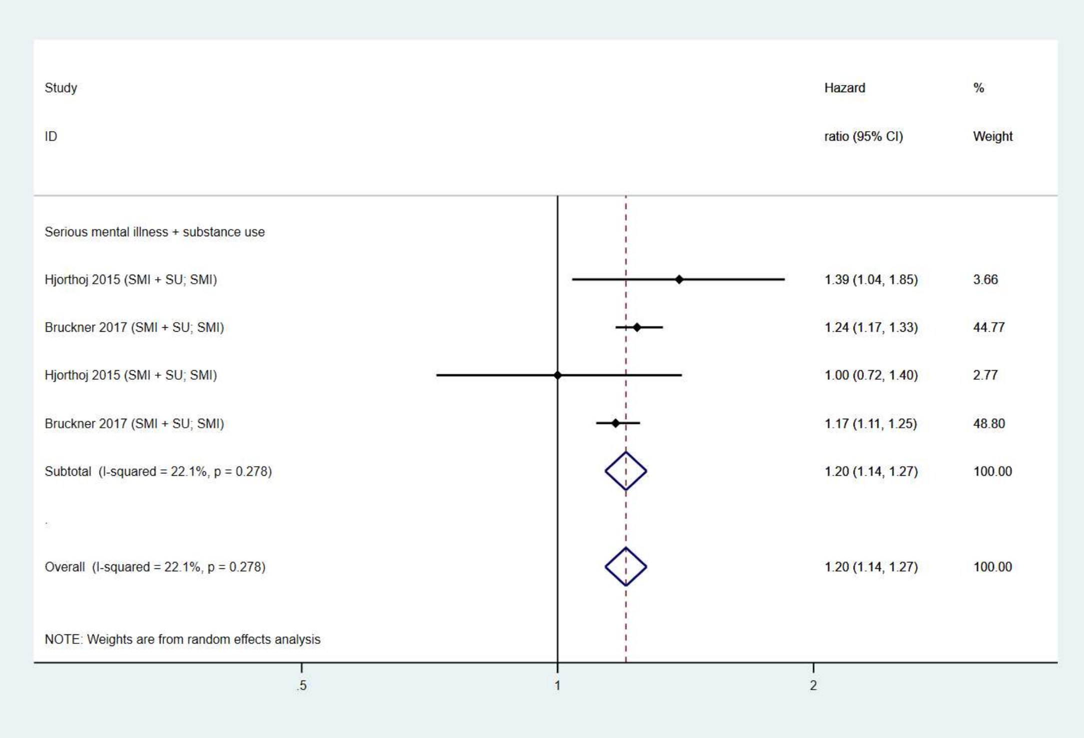

Figure A4.96. Forest plot for meta-analysis of studies reporting cumulative mortality from selected non-communicable diseases among people with multiple versus fewer exposures, by exposure combination

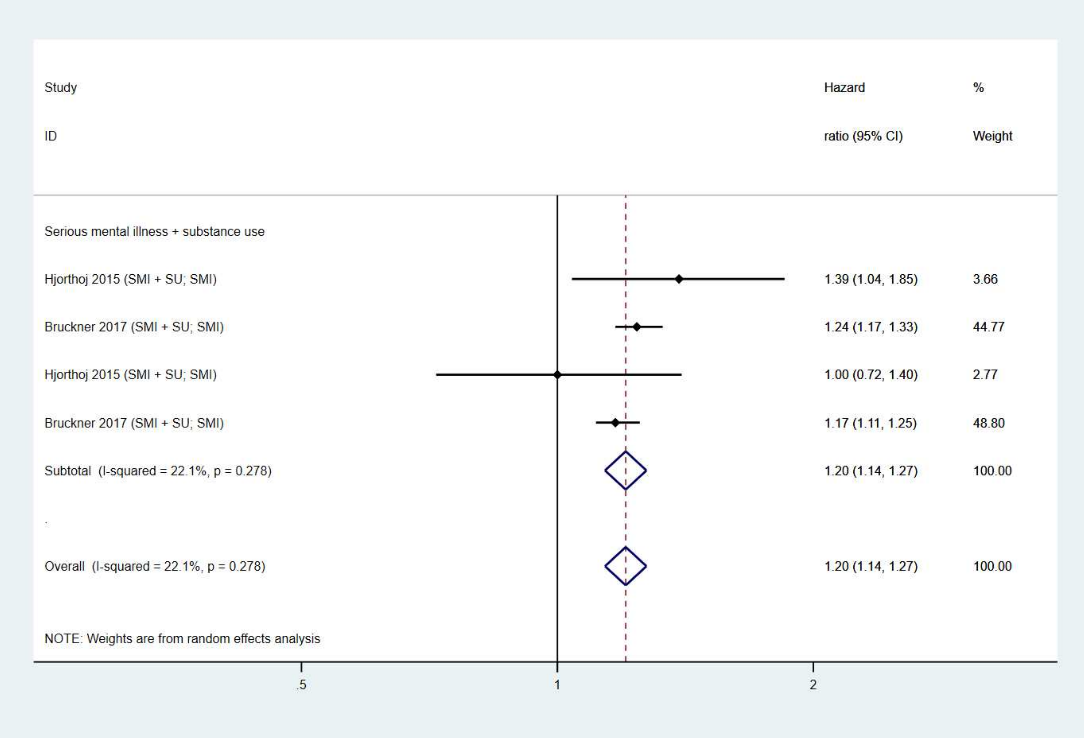

Figure A4.97. Forest plot for meta-analysis of studies reporting rate ratios for mortality from selected non-communicable diseases among people with multiple versus fewer exposures, by exposure combination

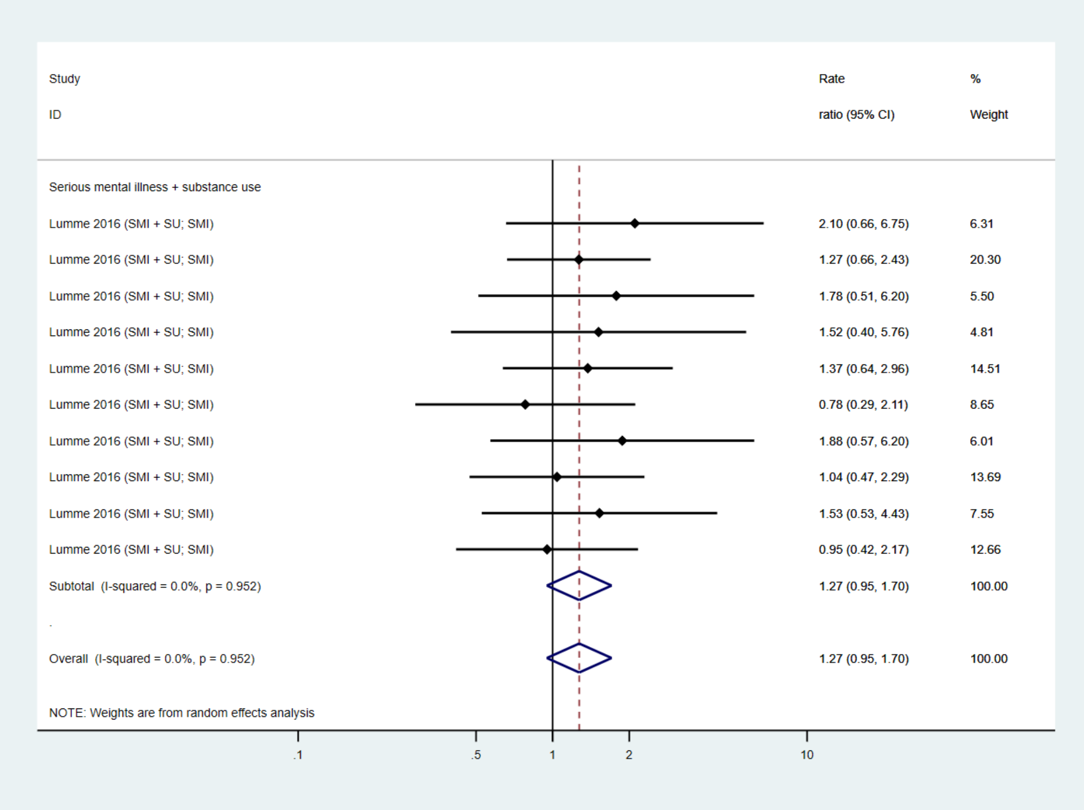

Figure A4.98. Forest plot for meta-analysis of studies reporting rate ratios for mortality from selected non-communicable diseases among people with multiple versus fewer exposures, by gender (where reported separately)

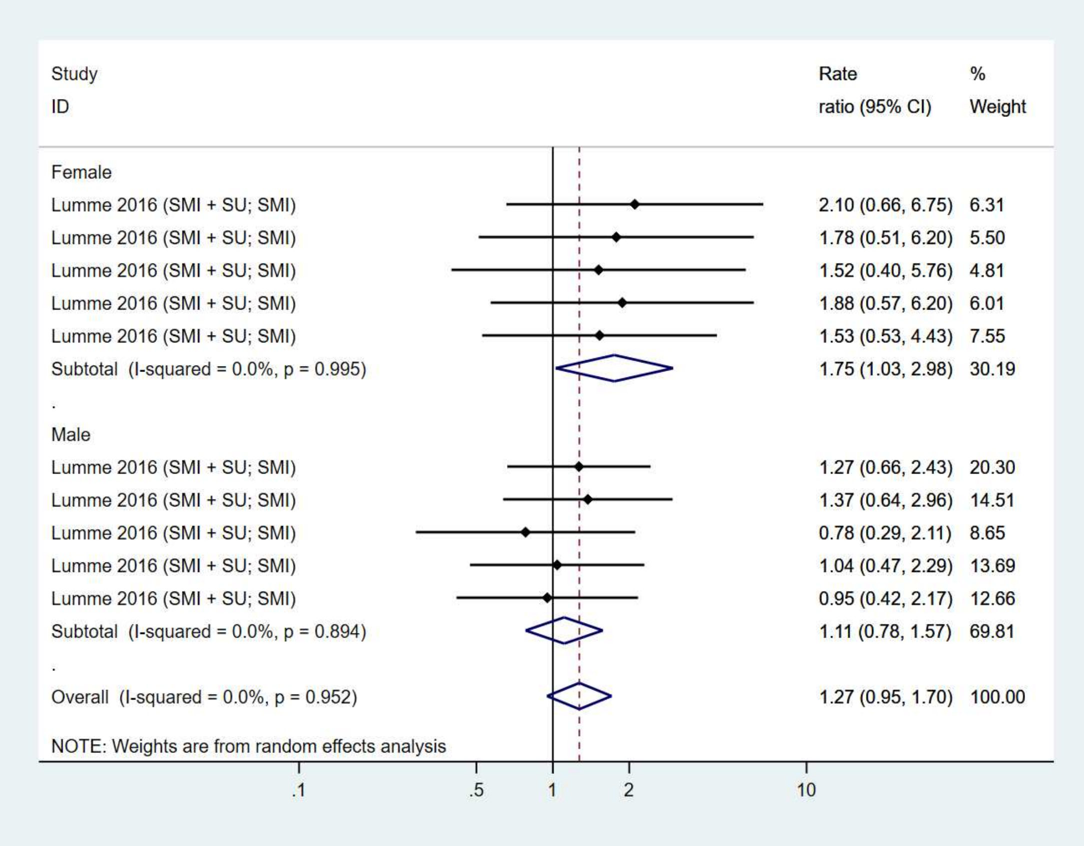

Appendix 5. Funnel plots for key outcomes described in manuscript for which meta-analysis feasible:

- All-cause mortality
- Mortality from external causes
- Blood-borne viruses
    - HIV
    - Hepatitis B
    - Hepatitis C
- Non-communicable diseases

Figure A5.1. Funnel plot for meta-analysis of all-cause mortality among people with multiple versus fewer exposures, by outcome measure.

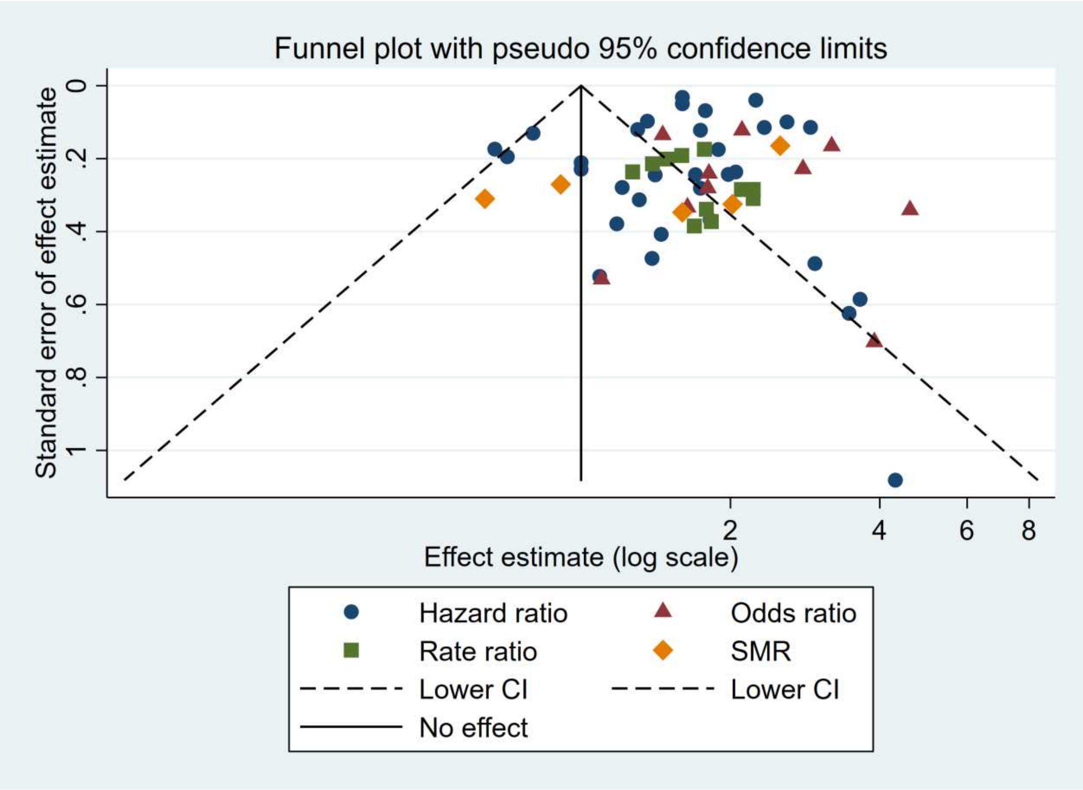

Figure A5.2. Funnel plot for meta-analysis of external cause mortality among people with multiple versus fewer exposures, by outcome measure.

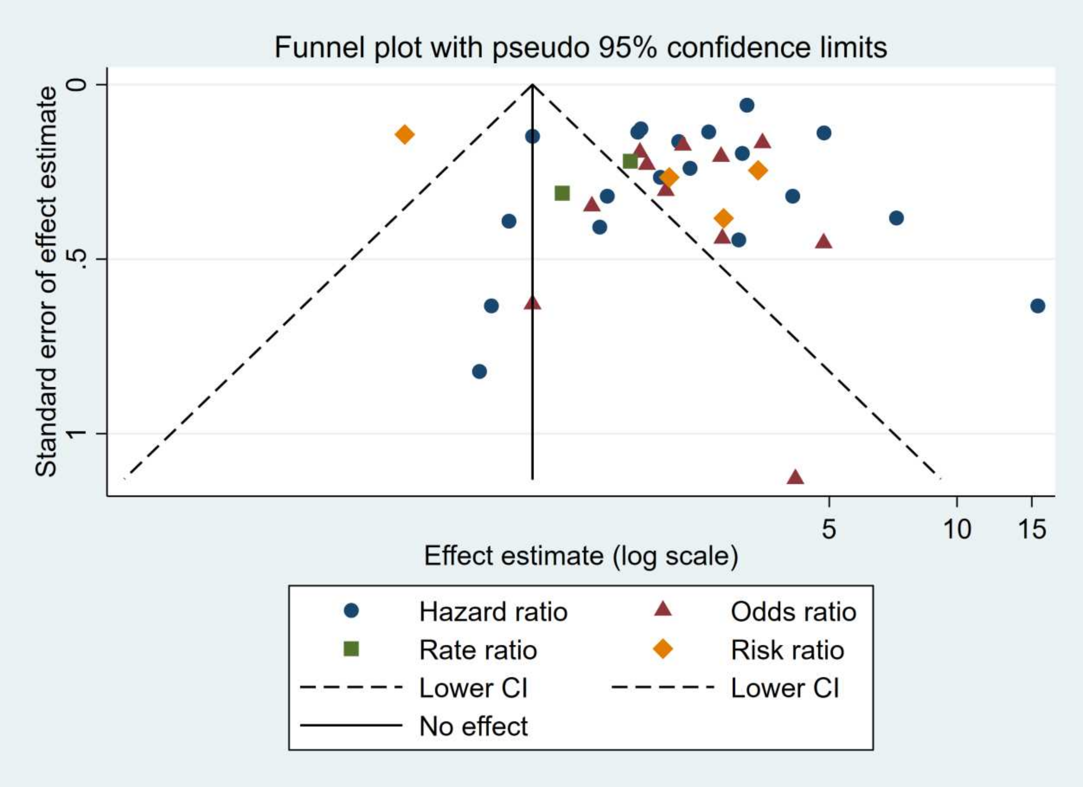

Figure A5.3. Funnel plot for meta-analysis of lifetime hepatitis C prevalence among people with multiple versus fewer exposures.

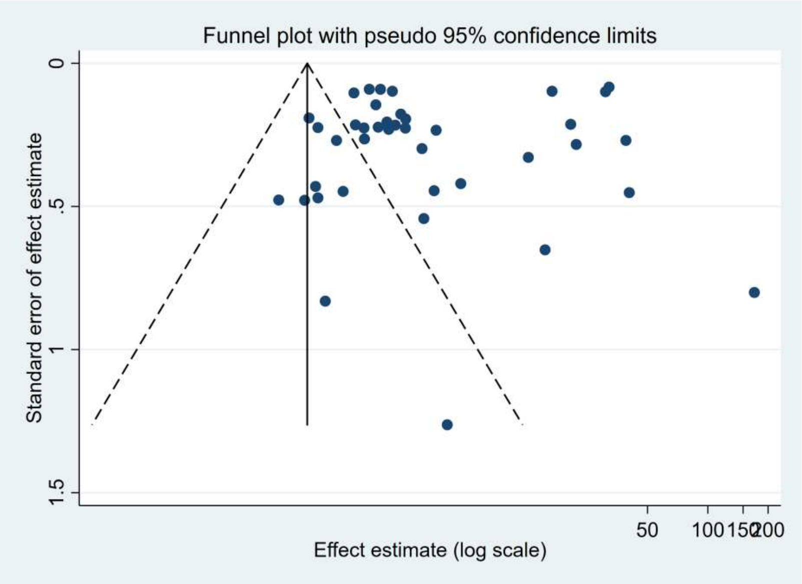

Figure A5.4. Funnel plot for meta-analysis of lifetime non-communicable disease prevalence among people with multiple versus fewer exposures.

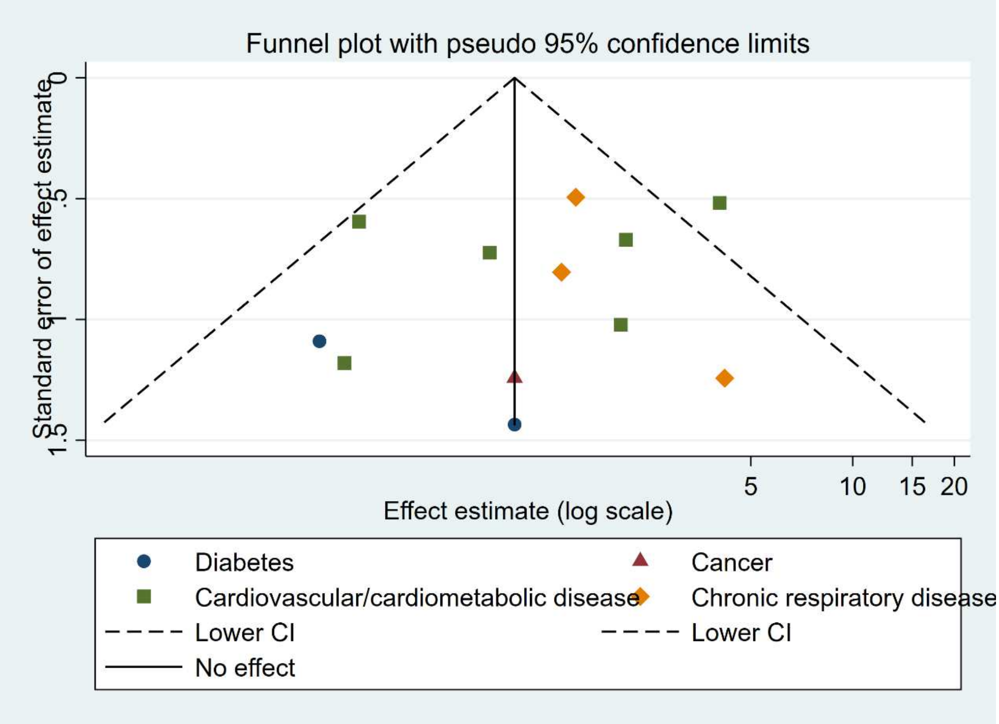

Figure A5.5. Funnel plot for meta-analysis of past year non-communicable disease prevalence among people with multiple versus fewer exposures.

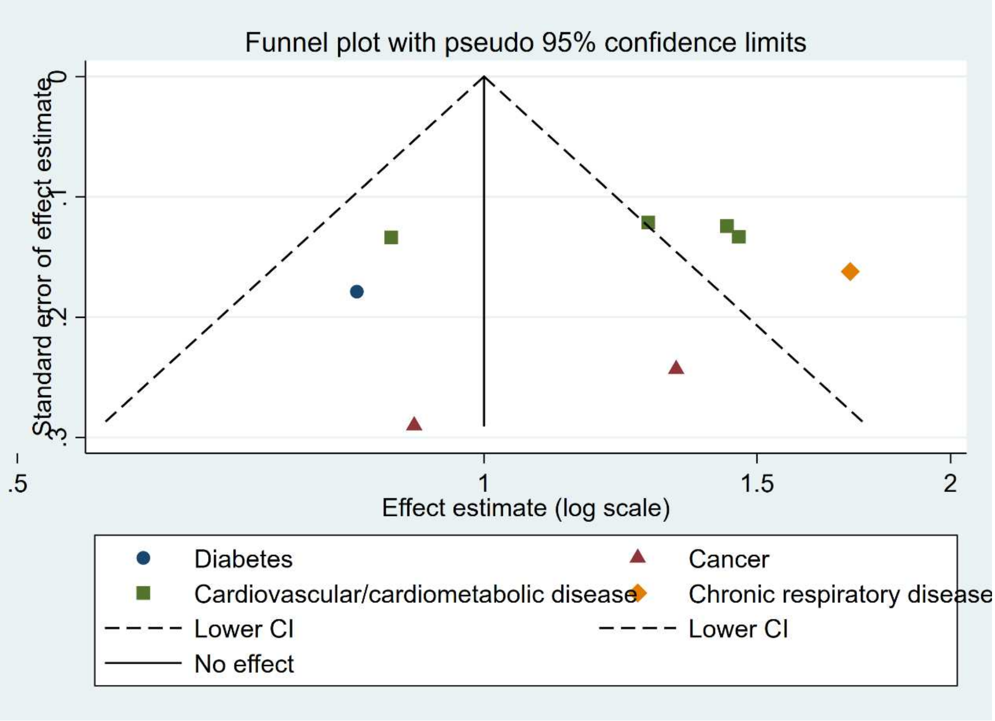

Figure A5.6. Funnel plot for meta-analysis of current non-communicable disease prevalence among people with multiple versus fewer exposures.

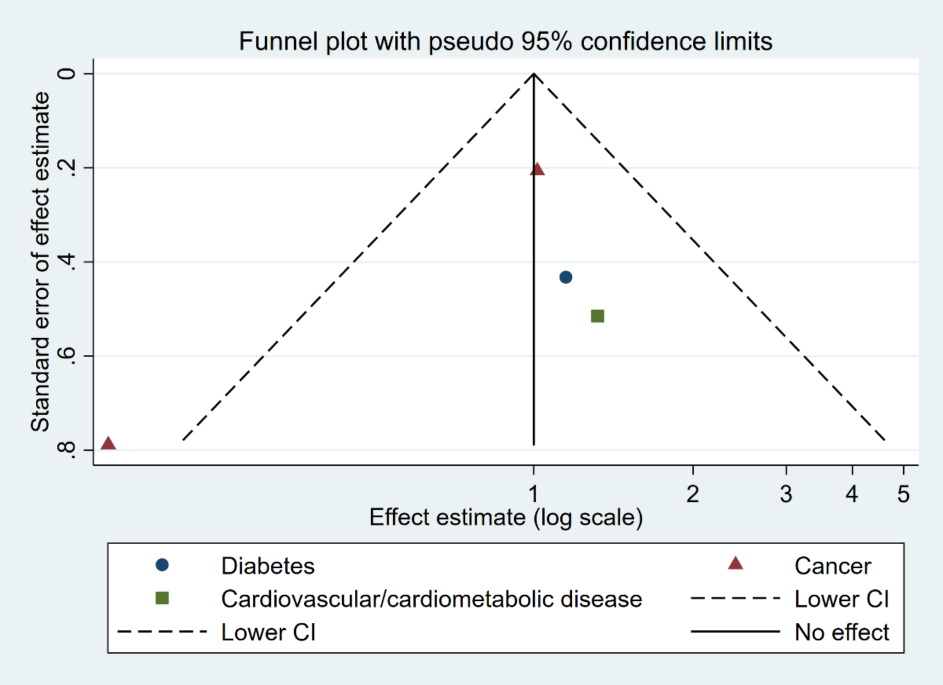

Figure A5.7. Funnel plot for meta-analysis of non-communicable disease mortality among people with multiple versus fewer exposures.

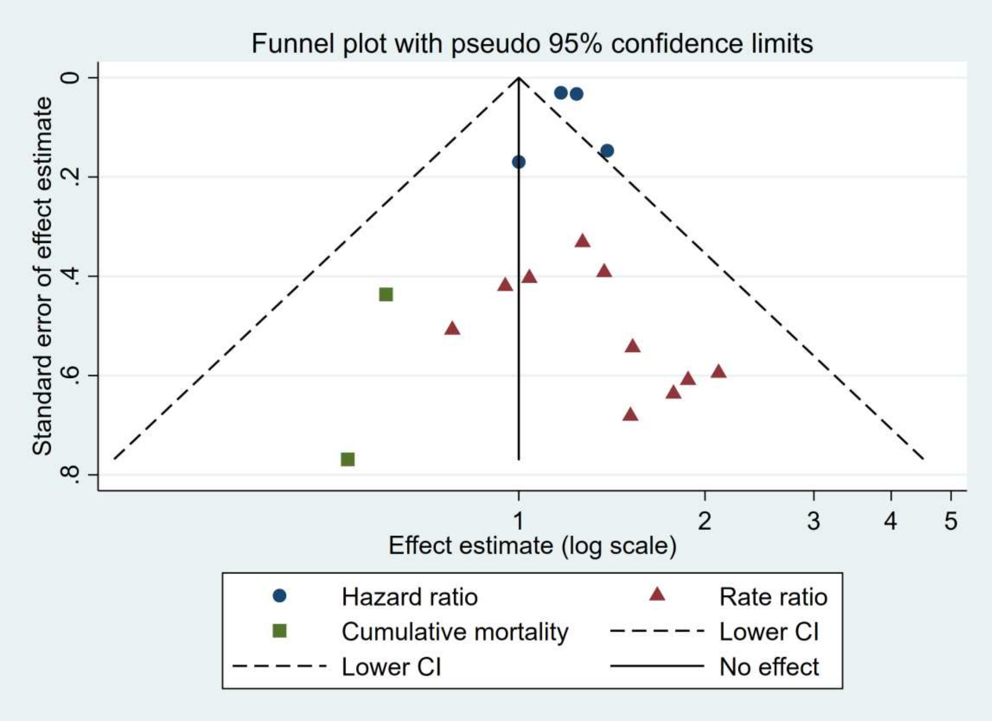

Supplement: Supplementary data [file jech-2020-215975supp002.pdf]
