# Supplementary material for: Health of people experiencing co-occurring homelessness, imprisonment, substance use, sex work and/or severe mental illness in high-income countries: a systematic review and meta-analysis
Source: J Epidemiol Community Health. 2021 Apr 23;75(10):1010–8. doi: 10.1136/jech-2020-215975 (PMC8458085; doi:10.1136/jech-2020-215975)
Supplement: Supplementary data [file jech-2020-215975supp001.pdf]

Contents

Appendix 1. Methods 2
Section 1.1. Search strategies 2
Section 1.3. Data extraction form exemplar: data points 8
Section 1.4. Data extraction form exemplar: risk of bias assessment 10
Section 1.6. De-duplication criteria 13
Appendix 2. Included studies 15
Section 2.1. Details of studies included in narrative synthesis 15
Section 2.2. Details of studies eligible according to review inclusion criteria but excluded due to overlapping data points 57

Appendix 1. Methods

Section 1.1. Search strategies

MEDLINE/EMBASE

String number	String
1	Exp Prisons/
2	Exp Prisoners/
3	Incarcerat*.mp
4	Prison*.mp
5	Jail*.mp
6	Imprison*.mp
7	Ex-prison*.mp
8	Or/1-8
9	Exp Homeless Persons/
10	Homeless*.mp
11	Or/9-10
12	exp Substance-Related Disorders/
13	(substance adj2 use*).mp
14	(substance adj2 abuse*).mp
15	(substance adj2 dependen*).mp
16	(substance adj2 misuse*).mp.
17	Drug-involved.mp
18	(inject* adj2 drug*).mp
19	(people who adj3 drugs).mp
20	addict*.mp
21	Or/12-21
22	Exp Sex Work/
23	Exp Sex Workers/
24	Prostitut*.mp
25	(sex adj2 work*).mp
26	Or/22-25
27	Exp Schizophrenia/
28	Exp Bipolar disorder/
29	psychosis.mp
30	psychotic.mp
31	(enduring mental illness).mp

32	(major mental illness).mp
33	(severe adj3 mental illness).mp
34	Or/27-33
35	8 and 11
36	8 and 21
37	8 and 26
38	8 and 34
39	11 and 21
40	11 and 26
41	11 and 34
42	21 and 26
43	21 and 34
44	26 and 34
45	(inclusion health).mp
46	(multiple exclusion homelessness).mp
47	(severe adj2 multiple disadvantage).mp
48	(multiple adj2 complex needs).mp
49	(complex adj2 co-occurring disorder*).mp
50	(dual diagnos#s).mp
51	Or/35-50
52	Exp mortality/
53	Mortality.mp
54	Incidence.mp
55	Prevalence.mp
56	Exp health status/
57	(Health status).mp
58	(Health outcome*).mp
59	Exp Morbidity/
60	Morbidit*.mp
61	Exp Hospitalization/
62	Hospitali#ation.mp
63	(Hospital adj2 admission*).mp
64	(admission* adj4 hospital).mp
65	healthcare.mp
66	(health services).mp
67	Multi#morbid*
68	Or/41-62
69	51 and 68
70	Editorial/

71	Comment/
72	Letter/
73	Historical article/
74	(Case report).mp
75	(Case series).mp
76	(Case study).mp
77	Review.pt
78	News.pt
79	Or/67-75
80	69 not 79
81	Limit 80 to (english language and yr="1998-current")

Additional searches for quality of life/self-reported health

String number	String
82	Exp quality of life/
83	Quality of life.mp
83	HRQoL.mp
84	Self#reported health.mp
85	Self#rated health.mp
86	Self#assessed health.mp
87	Subjective health.mp
88	Or/82-87
89	51 and 88
90	89 not 79
91	Limit 90 to (english language and yr="1998-current")

PSYCINFO

String number	String
1	DE "Prisoners"
2	TI Incarcerat* OR AB Incarcerat*
3	TI Prison* or AB Prison*
4	TI Jail* OR AB Jail*
5	TI Imprison* OR AB Imprison*
6	TI Ex-prison* OR AB Ex-prison*
7	DE "Legal detention"
8	DE "Incarceration"

9	DE “Homeless”
10	TI Homeless* OR AB Homeless*
11	DE “Drug Addiction”
12	DE “Drug dependency”
13	DE “Drug abuse”
14	DE “Intravenous drug usage”
15	DE “Substance use disorder”
16	DE “Addiction”
17	TI substance N2 use* OR AB substance N2 use*
18	TI Substance N2 abuse* OR AB Substance N2 abuse*
19	TI Substance N2 dependen* OR AB Substance N2 dependen*
20	TI Substance N2 misus* OR AB Substance N2 misus*
21	TI inject* N2 drug* OR AB inject* N2 drug*
22	TI Addict* OR AB Addict*
23	DE “Prostitution”
24	TI Sex N1 work* OR AB Sex N1 work*
25	TI Prostitut* OR AB Prostitut*
26	DE “Psychosis”
27	DE “Acute psychosis”
28	DE “Affective psychosis”
29	DE “Chronic psychosis”
30	DE “Paranoia (Psychosis)”
31	DE “Schizophrenia”
32	DE “Bipolar disorder”
33	TI Psychosis OR AB Psychosis
34	TI Psychotic OR AB Psychotic
35	TI severe N3 mental illness OR AB severe N3 mental illness
36	TI “major mental illness” OR AB “major mental illness”
37	Or/1-8
38	Or/9-10
39	Or/11-22
40	Or/23-25
41	Or/26-36
42	37 AND 38
43	37 AND 39
44	37 AND 40
45	37 AND 41
46	38 AND 39
47	38 AND 40

48	38 AND 41
49	39 AND 40
50	39 AND 41
51	40 AND 41
52	TI “inclusion health” OR AB “inclusion health”
53	TI “multiple exclusion homeless*” OR AB “multiple exclusion homeless*”
54	TI “severe N2 multiple disadvantage” OR AB “severe N2 multiple disadvantage”
55	TI “complex N2 co-occurring disorders” OR AB “complex N2 co-occurring disorders”
56	TI “multiple N2 complex needs” OR AB “multiple N2 complex needs”
57	DE “Dual diagnosis”
58	DE “Homeless Mentally Ill”
59	Or/42-58
60	DE “Mortality rate”
61	TI mortality OR AB mortality
62	TI Incidence OR AB Incidence
63	TI Prevalence OR AB Prevalence
64	TI Health status OR AB Health status
65	TI Health outcome* OR AB Health outcome*
66	DE “Morbidity”
67	DE “Health Care Utilization”
68	DE “Hospitalization”
69	DE “Hospital Admission”
70	DE “Hospital discharge”
71	TI Hospitali#ation OR AB Hospitali#ation
72	TI Hospital N2 admission OR AB Hospital N2 admission
73	TI Admission N4 hospital OR AB Admission N4 hospital
74	TI “health services” OR AB “health services”
75	TI “health*care” OR AB “health*care*
76	OR/59-75
77	59 and 75
78	77 Limited to 1998-2018, English, academic journals

Additional searches for quality of life/self-reported health

String number	String

82	DE “quality of life”
83	Quality of life
83	HRQoL
84	Self#reported health
85	Self#rated health
86	Self#assessed health
87	Subjective health
88	OR/82-87
89	51 and 88
90	89 not 79
91	90 Limited to 1998-2018, English, academic journals

Section 1.2. Data extraction form exemplar: study information

Study id	
Source	
First author	
Year published	
Second checker name	
Title	
Country	
Study design	
Larger study?	
Larger study name	
Recruitment strategy	
Study years	
Homeless definition	
Substance use definition	
Prison definition	
SMI definition	
Sex work definition	
Other selection criteria	
Total participants	
Average age	
% male	
Follow up length	
Study notes	

Section 1.3. Data extraction form exemplar: data points

Author year	
Study id	
Data point ID	
Data point number	
Title	
Second checker name	
Exposure combination	
Exposure notes	
Substance use definition Y/N	
SMI definition Y/N	
Outcome	
ICD chapter	
ICD subchapter	
Outcome type	
Ascertainment of outcome	

Gender	
Time period	
Outcome measure	
Point estimate	
Numerator	
Denominator	
Denominator type	
Other denominator	
Lower CI	
Upper CI	
SD	
Comparator description	
Comparator code	
Comparator point estimate	
Comparator numerator	
Comparator denominator	
Comparator denominator type	
Comparator other denominator	
Comparator lower CI	
Comparator upper CI	
Comparator SD	
Effect direction code	
Data point notes	
Adjusted	
Overlap flags	
Comparator 2 description	
Comparator 2 code	
Comparator 2 point estimate	
Comparator 2 numerator	
Comparator 2 denominator	
Comparator 2 lower CI	
Comparator 2 upper CI	
Comparator 2 SD	

Section 1.4. Data extraction form exemplar: risk of bias assessment

Study ID	
Datapoint ID	
Author date	
Title	
Study design	
Exposure combination	
Second checker name	
Outcome	
Agreement by category	
Reconciled by (names)	
Reconciled outcome	
ET score	
Second checker score	
Representativeness	
Ascertainment of exposure	
Ascertainment of outcome/case definition	
Response rate	
Selection of comparator	
Outcome of interest not present at start of study	
Long enough follow-up	
Adequacy of follow-up	
Control definition	
Same method of ascertainment for cases & controls	

Section 1.5. Modified version of Newcastle Ottawa Scale used for risk of bias assessment

ALL STUDIES

	**Representativeness**
1	a) good representativeness of target population (e.g. random sampling, complete sample)
1	b) selection process does not ensure representativeness (e.g. snowball sample) but is clearly described and unlikely to select high/low morbidity/mortality
0	c) selected group, with potential for selection bias affecting likelihood of morbidity/mortality
0	d) no description or unclear

	**Ascertainment of exposure**
1	a) from structured interview or medical/social records: clear description of how exposure defined
0	b) from structured interview or medical/social records: partial or unclear description of how exposure defined
1	c) based on location specific to the exposure of interest (e.g. prison)
0	d) based on location which may not be specific to exposure (e.g. generic MH or addiction service)
0	e) no description or unclear

	**Ascertainment of outcome/case definition**
1	a) Independent clinical assessment or validated tool
1	b) Record linkage or medical/administrative records
1	c) Self-report: question described and consistently applied
0	d) Self-report: question not described or not consistently applied
0	e) No description, or other inadequate

**Non-response rate**	
1	a) Comparability between respondents/non-respondents established and/or response rate >=70%
0	b) Comparability between respondents/non-respondents unsatisfactory/not described or response rate <70%
0	c) No description or unclear
0	d) Not applicable

	**Selection of comparator group**
1	a) Drawn from same community as exposed/cases
0	b) Drawn from a different source (e.g. hospital inpatients, general population)
0	c) no description

COHORT ONLY

	**Outcome of interest not present at the start**
1	a) Demonstrated this or not applicable
0	b) Not demonstrated this

	**Long enough follow-up for outcomes to occur**
1	a) Yes
0	b) No or unclear

	**Adequacy of follow-up**
1	a) Complete follow-up - all subjects accounted for
1	b) Subjects lost to follow up <20% or unlikely to introduce bias
0	c) Loss to follow-up substantial (>=20%) and/or likely to introduce bias
0	d) Not described

CASE-CONTROL ONLY

	**Definition of controls**
1	a) No history of disease/endpoint, or not applicable
0	b) No description or unclear

	**Same method of ascertainment for cases and controls**
1	a) Yes
0	b) No or unclear

Section 1.6. De-duplication criteria

Duplicate studies

i.e. those looking at same population, exposure combination, outcomes, and during overlapping time period:

1. Choose largest (or most representative)
2. If two studies with same size, choose the most recent
3. May be instances of duplicate data points but if some data points are unique between the two studies, overall study should be retained: individual duplicate data points will be removed at next stage

Duplicate data points:

- Three options
    - Keep in all analyses
    - Include only in narrative synthesis but exclude from meta-analyses
    - Exclude from all analyses
- Multiple comparison groups
    - Keep all for narrative synthesis
    - Select largest comparator for meta-analysis, unless this would affect comparability of results within studies, in which case choose most common/unifying comparison group
- If same exposure combination, outcome, and outcome measure
    - Review any exposure subgroups
        - Keep all for narrative synthesis
        - For meta-analysis, only keep those fitting our exposure definitions AND
            - Where no clear over-arching SU subgroup e.g. different drug classes which are not mutually exclusive and all meet our exposure definitions – for meta-analysis, select:
                - Injecting drug use
                - If IDU not available, heroin/opioids
                - If heroin/opioids not available, cocaine/crack
                - If not, dependence/abuse/problem use in favour of use
                - If not, largest group
            - Where both prison/jail reported and not mutually exclusive, select for meta-analysis the prison subgroup
            - Sex work – if multiple types (e.g. exchange for drugs/money) and not mutually exclusive, select for meta-analysis the ‘exchange for money’ subgroup
    - Outcome measures
        - For narrative synthesis, keep absolute measure if able to derive relative measure (e.g. prevalence with raw numbers that allows OR to be calculated)
            - E.g. mortality rate with raw numbers preferred over OR/HR
        - If not, prefer those with confidence intervals
        - Where longitudinal data available and both longitudinal/baseline data reported, mark as incidence/prevalence in meta-analysis category – to allow synthesis separately by measure

- Varying time points of follow up or prevalence periods
  - Keep all for narrative synthesis
  - For meta-analysis, stratify analyses by prevalence period
- Gender
  - Keep all for narrative synthesis
  - For meta-analysis, if reported separately, use both of the stratified data points; if reports combined and for one gender, use only the combined estimate
- Composite outcomes
  - Keep all for narrative synthesis
  - For meta-analysis, where there are overlapping composite outcomes e.g. one data point is CVD death, another is CVD or diabetes death – exclude narrower category unless can be reported separately in stratified analysis
- Different exposure periods
  - Keep all for narrative synthesis
  - For meta-analysis, select lifetime in preference to current exposure, where available

Logs of de-duplication decisions are available on request.

Appendix 2. Included studies

Section 2.1. Details of studies included in narrative synthesis

Author & year	Title	Country	Study years	Design	Number of data points included	Outcomes	Risk of bias
Aakre 2014	Trauma exposure and PTSD in women with schizophrenia and coexisting substance use disorders: Comparisons to women with severe depression and substance use disorders	USA	not stated	Cross-sectional	1	PTSD	High
Abdel-Baki 2017	Symptomatic and functional outcomes of substance use disorder persistence 2 years after admission to a first-episode psychosis program	Canada	2005-2012	Cohort study	10	Depression; Quality of life	Low
Abraham 2014	Self-efficacy and quality of life among people with bipolar disorder	USA	2008-2011	Cross-sectional analysis of interventional study	2	Physical & mental HRQoL	High
Abram 2015	Comorbidity and continuity of psychiatric disorders in youth after detention: a prospective longitudinal study	USA	1995-2003	Cohort study	8	Alcohol disorder, antisocial personality disorder, major depression, PTSD	Low
Abrams 2012	Cervical cancer screening and acute care visits among Medicaid enrollees with mental and substance use disorders	USA	2004-2005	Cohort study	3	Genital cancer, HIV/AIDS, other STD	Low
Addington 1998	Effect of substance misuse in early psychosis	Canada	not stated	Cross-sectional	2	Quality of life	High
Akiyama 2017	Correlates of hepatitis C virus infection in the targeted testing program of the New York city jail system: Epidemiologic patterns and priorities for action	USA	2013-2014	Cross-sectional	2	Anti-HCV seropositivity	High
Allsworth 2007	The influence of stress on the menstrual cycle among newly incarcerated women	USA	2002-2003	Cross-sectional	6	Amenorrhoea, irregular menstrual cycle	Low
Allwright 2000	Prevalence of antibodies to hepatitis B, hepatitis C, and HIV and risk factors in Irish prisoners: results of a national cross sectional survey	Ireland	1998	Cross-sectional	3	HIV, anti-HCV and HBV core antigen prevalence	Low
Altice 1998	Predictors of HIV infection among newly sentenced male prisoners	USA	1993	Cross-sectional	5	HIV	Low

Author & year	Title	Country	Study years	Design	Number of data points included	Outcomes	Risk of bias
Altice 2005	Correlates of HIV infection among incarcerated women: implications for improving detection of HIV infection	USA	1994-1996	Cross-sectional	2	HIV	Low
Alvarez 2014	Prevalence and correlates of hepatitis C virus infection among inmates at two New York State correctional facilities	USA	2009-2013	Cross-sectional	1	Hepatitis C	Low
Amesty 2008	Prevalence and correlates of previous hepatitis B vaccination and infection among young drug-users in New York City	USA	2000-2004	Cross-sectional	1	Previous/current HBV infection	High
Andersen 2014	Traumatic brain injury and cognitive impairment in men who are homeless	Canada	2012	Cross-sectional	1	Traumatic brain injury	Low
Anna Gyarmathy 2009	Vulnerability to drug-related infections and co-infections among injecting drug users in Budapest, Hungary	Hungary	2005-2006	Cross-sectional	2	Syphilis, chlamydia, any hepatitis	High
Arribas-Ibar 2017	Suicidal behaviours in male and female users of illicit drugs recruited in drug treatment facilities	Spain	2012	Cross-sectional	2	Suicidal ideation or plans last 12 months	Low
Astals 2008	Impact of substance dependence and dual diagnosis on the quality of life of heroin users seeking treatment	Spain	2000-2002	Cross-sectional	4	Physical & mental HRQoL	Low
Auquier 2013	Toward meeting the needs of homeless people with schizophrenia: the validity of quality of life measurement	France	not stated	Cross-sectional analysis of interventional study	2	Physical & mental HRQoL	Low
Babidge 2001	Mortality among homeless people with schizophrenia in Sydney, Australia: a 10-year follow-up	Australia	1988-1998	Cohort study	9	All-cause mortality and cause-specific mortality (cardiovascular, gastrointestinal, infection, neoplasm, renal, respiratory, seizure, suicide)	Low
Babudieri 2005	Correlates of HIV, HBV, and HCV infections in a prison inmate population: results from a multicentre study in Italy	Italy	2001-2002	Cross-sectional	5	HIV, HBV, HCV infection	Low
Backmund 2006	Factors associated with exposure to hepatitis B virus in injection drug users	Germany	1991-1997	Cross-sectional	1	HBV infection	Low
Bacon 2006	Commercial sex work and risk of HIV infection among young drug-injecting men who have sex with men in San Francisco	USA	not stated	Cross-sectional	4	HIV	Low

Author & year	Title	Country	Study years	Design	Number of data points included	Outcomes	Risk of bias
Baethge 2005	Substance abuse in first-episode bipolar I disorder: indications for early intervention	USA	1989-1996	Cohort study	2	Anxiety disorder	Low
Baillargeon 2003	Hepatitis C seroprevalence among newly incarcerated inmates in the Texas correctional system	USA	1998-1999	Cross-sectional	2	HCV	Low
Baillargeon 2017	The changing epidemiology of HIV in the criminal justice system	USA	2014	Cross-sectional	2	HIV	Low
Bair 2005	Prevalence and risk factors for hepatitis C virus infection among adolescents in detention	USA	2000-2000	Cross-sectional	2	HCV	Low
Bakst 2010	Antecedents and patterns of suicide behavior in first-admission psychosis	USA	1989-1995	Cohort study	2	Suicidal ideation or attempt	High
Barak 2008	Suicide attempts of schizophrenia patients: a case-controlled study in tertiary care	Israel	1990-2005	Case-control study	1	Suicide attempt	Low
Barrett 2010	Suicidality before and in the early phases of first episode psychosis	Norway	2004-2008	Cross-sectional	2	Suicidal ideation or attempt	High
Barrio 2007	Prevalence of HIV infection among young adult and non-injecting heroin users in Spain in the era of harm reduction programmes	Spain	2001-2003	Cross-sectional analysis of cohort study	4	HIV	Low
Barros 2008	A survey of HIV and HCV among female prison inmates in Portugal	Portugal	2005	Cross-sectional	3	HIV, HCV	High
Barrow 1999	Mortality among homeless shelter residents in New York City	USA	1987	Cohort study	6	All-cause mortality	Low
Bauer-Staeb 2017	Prevalence and risk factors for HIV, hepatitis B, and hepatitis C in people with severe mental illness	Sweden	2010	Cross-sectional	12	HIV, HBV, HCV infection	Low
Begier 2010	Undiagnosed HIV infection among New York City jail entrants 2006	USA	2006	Cross-sectional	1	HIV	High
Beijer 2007	Mortality, mental disorders and addiction: a 5-year follow-up of 82 homeless men in Stockholm	Sweden	1995-2001	Cohort study	1	All-cause mortality	Low
Beijer 2011	Mortality and causes of death among homeless women and men in Stockholm	Sweden	1995-2005	Cohort study	2	All-cause mortality	Low
Belza 2001	Sociodemographic characteristics and HIV risk behaviour patterns of male sex workers in Madrid, Spain	Spain	1998	Cross-sectional	1	HIV	High

Author & year	Title	Country	Study years	Design	Number of data points included	Outcomes	Risk of bias
Belza 2005	Risk of HIV infection among male sex workers in Spain	Spain	2000-2002	Cross-sectional	1	HIV	Low
Benaiges 2012	Health-related quality of life in patients with dual diagnosis: clinical correlates	Spain	2010-2011	Cross-sectional	7	Asthma/allergy, diabetes, HIV, HBV, HCV, hypertension, mental HRQoL, physical HRQoL	Low
Beyer 2007	Prevalence of HIV infection in a general psychiatric outpatient population	USA	2001-2004	Cross-sectional	2	HIV	Low
Bhalla 2018	Mental health multimorbidity and poor quality of life in patients with schizophrenia	USA	2001-2004	Cross-sectional analysis of interventional study	3	Quality of life, mental HRQoL	High
Bhatta 2014	Suicidal behaviours among adolescents in juvenile detention: role of adverse life experiences	USA	2003-2007	Cross-sectional	6	Suicidal ideation or attempt	Low
Binswanger 2016	Clinical risk factors for death after release from prison in Washington State: a nested case-control study	USA	1999-2009	Case-control study	21	All-cause mortality, overdose mortality	Low
Bizzarri 2009	Comorbidity with anxiety and substance use disorders in patients with schizophrenia	Italy	2003-2006	Cross-sectional	12	Agoraphobia, generalised anxiety disorder, obsessive compulsive disorder, panic disorder, simple phobia, social phobia	Low
Bjorkenstam 2014	Suicide in first episode psychosis: a nationwide cohort study	Sweden	1973-2008	Cohort study	1	Suicide mortality	Low
Blome 2011	Minimal transmission of HIV despite persistently high transmission of hepatitis C virus in a Swedish needle exchange program	Sweden	1997-2005	Cohort study	2	HCV	1 datapoint scored as low; 1 datapoint scored as high
Bobo 2018	The relative influence of individual risk factors for attempted suicide in patients with bipolar I versus bipolar II disorder	USA	not stated - Biobank initiated in 2009	Cross-sectional analysis of cohort study	1	Suicide attempt	High

Author & year	Title	Country	Study years	Design	Number of data points included	Outcomes	Risk of bias
Bogdanowicz 2015	Double trouble: Psychiatric comorbidity and opioid addiction-all-cause and cause-specific mortality	UK	2008-2012	Cohort study	4	All-cause and cause-specific mortality (alcoholic and other liver disease, overdose, other)	Low
Bonaccorsi 2007	The prevalence of latent tuberculosis infection in social marginal groups. An experience conducted in Tuscany	Italy	not stated	Cross-sectional	1	Latent TB	High
Bonnycastle 2011	Injecting risk into prison sentences: A quantitative analysis of a prisoner-driven survey to measure HCV/HIV seroprevalence, risk practices, and viral testing at one Canadian male federal prison	Canada	2002	Cross-sectional	2	HIV, HCV	Low
Boter 2010	Generalizability of the results of efficacy trials in first-episode schizophrenia: Comparisons between subgroups of participants of the European First Episode Schizophrenia Trial (EUFEST)	Multiple	2002-2006	Cross-sectional analysis of interventional study	2	Depression, suicidality	Low
Boyer 2015	The Relationship between Methamphetamine Use and Dental Caries and Missing Teeth	USA	1998	Cross-sectional	3	Decayed surfaces, decayed teeth, missing teeth	Low
Bretteville-Jensen 2015	Illicit use of opioid substitution drugs: prevalence, user characteristics, and the association with non-fatal overdoses	Norway	2006-2013	Cross-sectional	1	Non-fatal overdose	High
Bruckner 2017	Cardiovascular Disease Mortality of Medicaid Clients with Severe Mental Illness and a Co-occurring Substance Use Disorder	USA	2002-2007	Cohort study	2	Mortality from cardiovascular disease and/or diabetes	Low
Brugal 2005	Evaluating the impact of methadone maintenance programmes on mortality due to overdose and aids in a cohort of heroin users in Spain	Spain	1992-1999	Cohort study	2	All-cause and cause-specific mortality (AIDS)	Low
Bruneau 2012	The rising prevalence of prescription opioid injection and its association with hepatitis C incidence among street-drug users	Canada	1988-2009	Cohort study	2	HCV	Low
Burgos 2000	Correlates of sexually transmitted infections among street-based female adolescent sex workers in Puerto Rico: Implications for community health	USA	not stated	Cross-sectional	1	Sexually transmitted infections	Low

Author & year	Title	Country	Study years	Design	Number of data points included	Outcomes	Risk of bias
Burnette 2008	Prevalence and health correlates of prostitution among patients entering treatment for substance use disorders	USA	1993-1995	Cross-sectional	18	Anxiety, circulatory problems, depression, HIV/AIDS, hepatitis, suicide attempt, neurological problems, respiratory problems, STDs	Low
Burt 2006	Recent condom use by arrested injection drug users in King County, Washington, USA	USA	1998-2002	Cross-sectional	2	HIV	Low
Butler 1999	Seroprevalence of markers for hepatitis B, C and G in male and female prisoners--NSW, 1996	Australia	1996	Cross-sectional	12	HBV, HCV, HGV	Low
Butler 2004	Prisoners are at risk for hepatitis C transmission	Australia	1996-2001	Cohort study	1	HCV	Low
Butler 2007	The 2004 Australian prison entrants' blood-borne virus and risk behaviour survey	Australia	2004	Cross-sectional	4	HIV, HBV, HCV infection	Low
Butterfield 2004	Racial differences in hepatitis B and hepatitis C and associated risk behaviors in veterans with severe mental illness	USA	1998-2000	Cross-sectional	8	HBV, HCV	High
Callander 2018	Rising Chlamydia and Gonorrhoea Incidence and Associated Risk Factors among Female Sex Workers in Australia: A Retrospective Cohort Study	Australia	2009-2015	Cohort study	8	Chlamydia, gonorrhoea, syphilis	Low
Calvo 2017	Young people who use drugs engaged in harm reduction programs in New York City: overdose and other risks	USA	2014-2015	Cross-sectional	1	Overdose	Low
Calzavara 2007	Prevalence of HIV and hepatitis C virus infections among inmates of Ontario remand facilities	Canada	2003-2004	Cross-sectional	3	HIV, HCV, HIV-HCV coinfection	Low
Cardoso 2017	Clinical outcomes in children and adolescents with bipolar disorder and substance use disorder comorbidity	USA	2003-2015	Cross-sectional	2	Anxiety	High
Carey 2016	Hepatitis C virus testing and treatment among persons receiving buprenorphine in an office-based program for opioid use disorders	USA	2003-2013	Cross-sectional	2	HCV	Low
Carmel 2016	Suicide risk and associated demographic and clinical correlates among primary care patients with recent drug use	USA	2009-2012	Cross-sectional analysis of interventional study	2	Suicidal ideation or attempt	High

Author & year	Title	Country	Study years	Design	Number of data points included	Outcomes	Risk of bias
Carpenter 1999	Prevalence of HIV-1 among recent arrestees in Los Angeles County, California: serial cross-sectional study, 1991-1995	USA	1991-1995	Cross-sectional	3	HIV	High
Carr 2009	Sociodemographic and clinical characteristics of patients presenting with first-episode psychosis and concurrent substance misuse	Canada	1996-2005	Cross-sectional analysis of cohort study	2	Anxiety, depression	Low
Carra 2016	Psychosocial functioning, quality of life and clinical correlates of comorbid alcohol and drug dependence syndromes in people with schizophrenia across Europe	Multiple	not stated	Cross-sectional analysis of cohort study	3	Quality of life	Low
Caton 2013	Rates and correlates of HIV and STI infection among homeless women	USA	2007-2008	Cross-sectional	2	HIV/STI	Low
Cavanaugh 2010	Sexually transmitted infections among pregnant heroin- or cocaine-addicted women in treatment: the significance of psychiatric co-morbidity and sex trade	USA	2006-2008	Cross-sectional	1	STI (gonorrhoea, syphilis, chlamydia, herpes, genital warts, trichomonas)	High
Chafetz 2005	The poor general health of the severely mentally ill: impact of schizophrenic diagnosis	USA	1997-2001	Cross-sectional	2	Diabetes, emphysema/COPD	Low
Chahua 2013	Non-fatal opioid overdose and major depression among street-recruited young heroin users	Spain	2002-2005	Cross-sectional analysis of cohort study	2	Non-fatal overdose	Low
Chahua 2015	Quality of life in a community sample of young cocaine and/or heroin users: the role of mental disorders	Spain	2001-2006	Cross-sectional analysis of cohort study	3	Health-related quality of life	2 datapoints scored as low; 1 datapoint scored as high
Chambers 2014	Factors associated with poor mental health status among homeless women with and without dependent children	Canada	2004-2005	Cross-sectional	2	Mental health problem, mental HRQoL	Low
Champion 2004	Incidence of hepatitis C virus infection and associated risk factors among Scottish prison inmates	UK	1999-2000	Cohort study	1	HCV	Low
Chang 2015	Substance use disorders, psychiatric disorders, and mortality after release from prison: a nationwide longitudinal cohort study	Sweden	2000-2009	Cross-sectional analysis of cohort study	8	All-cause and cause-specific mortality (external cause)	Low

Author & year	Title	Country	Study years	Design	Number of data points included	Outcomes	Risk of bias
Chang 2015	Prevalence and risk factors for suicidal behavior in young people presenting with first-episode psychosis in Hong Kong: a 3-year follow-up study	Hong Kong	2001-2003	Cohort study	1	Attempted or completed suicide	High
Chapman 2005	Factors associated with suicide attempts in female inmates: the hegemony of hopelessness	Canada	not stated	Cross-sectional	1	Attempted suicide	High
Chapman 2007	The role of antisocial and borderline personality features in substance depenence among incarcerated females	Canada	not stated	Cross-sectional	2	Antisocial personality disorder, borderline personality disorder	High
Chen 1999	Psychiatric co-morbidity among male heroin addicts: differences between hospital and incarcerated subjects in Taiwan	Taiwan	1994-1996	Cross-sectional	13	Alcohol dependence, antisocial personality disorder, conversion disorder, cyclothymia, dysthymia, generalised anxiety disorder, hypochondriacal disorder, major depression, obsessive compulsive disorder, panic disorder, paranoid personality disorder, other personality disorder, somatisation disorder	High
Chmielewski 2007	Presence of Bartonella spp. In various human populations	Poland	Not stated	Cross-sectional	1	Bartonella henselae infection	High
Christensen 2000	Prevalence and incidence of bloodborne viral infections among Danish prisoners	Denmark	1996-1997	Cohort study	11	HAV, HBV, HCV, HDV, HIV, HTLV	3 datapoints scored as low, 8 scored as high
Christensen 2002	High prevalence of hepatitis E antibodies among Danish prisoners and drug users	Denmark	not stated	Cross-sectional	2	Anti-HEV positive	Low
Cohan 2006	Sex worker health: San Francisco style	USA	1999-2004	Cross-sectional	2	Sexually transmitted infections	High

Author & year	Title	Country	Study years	Design	Number of data points included	Outcomes	Risk of bias
Coid 2011	Prisoners with psychosis in England and Wales: diversion to psychiatric inpatient services?	UK	1997 (prisons only; other survey not stated)	Cross-sectional	12	Alcohol dependence, antisocial personality disorder, borderline personality disorder, depressive disorders, lifetime suicide attempt, narcisstic personality disorder, obsessive compulsive personality disorder, paranoid personality disorder, schizotypal personality disorder, serious injury/assault	Low
Commander 2001	A comparison of the needs of homeless and neverhomeless patients with psychotic disorders	UK	not stated	Cross-sectional	1	Alcohol problem	High
Comtois 2004	Clinicians' assessments of bipolar disorder and substance abuse as predictors of suicidal behavior in acutely hospitalized psychiatric inpatients	USA	1996-2003	Cross-sectional	3	Suicide attempt	Low
Cook 2001	Predictors of hepatitis B and C infection in injecting drug users both in and out of drug treatment	UK	1997-1999	Cross-sectional	2	HBV, HCV	High
Cooper 1999	Neurocognitive deficits in the dually diagnosed with schizophrenia and cocaine abuse	USA	not stated	Cross-sectional analysis of interventional study	1	Depression	High
Cornford 2011	Deep vein thromboses in users of opioid drugs: incidence, prevalence and risk factors	UK	2009	Cross-sectional	1	Deep vein thrombosis	High
Cotton 2012	Depressive symptoms in first episode schizophrenia spectrum disorder	Australia	1998-2000	Cohort study	4	Moderate to severe depression, persistent depression	High
Craine 2009	Incidence of hepatitis C in drug injectors: the role of homelessness, opiate substitution treatment, equipment sharing, and community size	UK	not stated	Cohort study	2	HCV	1 datapoint scored as low; 1 datapoint scored as high

Author & year	Title	Country	Study years	Design	Number of data points included	Outcomes	Risk of bias
Croxford 2015	Sex work amongst people who inject drugs in England, Wales and Northern Ireland: findings from a National Survey of Health Harms and Behaviours	UK	2011	Cross-sectional	6	HBV, HCV, HIV	Low
Cuddeback 2010	General Medical Problems of Incarcerated Persons With Severeand Persistent Mental Illness: A Population-Based Study	USA	1999-2003	Cohort study	13	Blood disorder, cancer, circulatory system, digestive system, endocrine disorder, infectious disease, injury, multiple health problems, musculoskeletal system, nervous system, respiratory system, skin disorder	Low
Cullen 2014	Factors associated with recently acquired hepatitis C virus infection in people who inject drugs in England, Wales and Northern Ireland: new findings from an unlinked anonymous monitoring survey	UK	2011	Cross-sectional	2	HCV	1 datapoint scored as low; 1 datapoint scored as high
Cuomo 2008	Aggression, impulsivity, personality traits, and childhood trauma of prisonerswith substance abuse and addiction	Italy	not stated	Cross-sectional	1	Suicide attempt	Low
Dahlman 2017	Physical pain is common and associated with nonmedical prescription opioid use among people who inject drugs	USA	2011-2013	Cross-sectional	1	Pain	Low
Daigre 2017	The role of dual diagnosis in health-related quality of life among treatment-seeking patients in Spain	Spain	2006-2016	Cross-sectional	2	Mental and physical HRQoL	High
Dalton 2003	Suicide risk in bipolar patients: The role of co-morbid substance use disorders	Canada	not stated	Cross-sectional	1	Suicide attempt	High
Darke 2004	Attempted suicide among injecting and noninjecting cocaine users in Sydney, Australia	Australia	2002-2003	Cross-sectional	1	Suicide attempt	High
Dausey 2003	Psychiatric comorbidity and the prevalence of HIV infection in a sample of patients in treatment for substance abuse	USA	1992-1997	Cross-sectional analysis of cohort study	1	HIV	High

Author & year	Title	Country	Study years	Design	Number of data points included	Outcomes	Risk of bias
Davstad 2009	An 18-year follow-up of patients admitted to methadone treatment for the first time	Sweden	1989-2003	Cohort study	1	All-cause mortality	Low
de la Fuente 2006	HTLV infection among injection and non-injection heroin users in Spain: prevalence and correlates	Spain	2001-2003	Cross-sectional analysis of cohort study	2	HTLV-2 infection	Low
de la Hoya 2011	Hepatitis C and B prevalence in Spanish prisons	Spain	2008	Cross-sectional	2	HBV, HCV	Low
Delahanty 2001	Differences in rates of depression in schizophrenia by race	USA	1988-1990	Cross-sectional	1	Quality of life	High
Dias 2015	Risk-taking behaviours and HIV infection among sex workers in Portugal: results from a cross-sectional survey	Portugal	2011	Cross-sectional	1	HIV	Low
Dibben 2011	Differences in 5-year survival after a 'homeless' or 'housed' drugs-related hospital admission: a study of 15--30-year olds in Scotland	UK	1986-2001	Cohort study	1	All-cause mortality	High
Dickey 2000	Schizophrenia, substance use disorders and medical co-morbidity	USA	1994	Cross-sectional	9	Acute respiratory disease, asthma, diabetes, gastrointestinal disease, heart disease, hypertension, skin infections, injury/poisoning, malignant neoplasms	Low
Dickey 2002	Medical morbidity, mental illness, and substance use disorders	USA	1997-1998	Cross-sectional	8	Acute respiratory disease, asthma, diabetes, gastrointestinal disease, heart disease, hypertension, skin infections, injury/poisoning, malignant neoplasms	Low
Dickey 2004	Externally caused deaths for adults with substance use and mental disorders	USA	1997-1998	Cohort study	2	Injury death	Low

Author & year	Title	Country	Study years	Design	Number of data points included	Outcomes	Risk of bias
Dickson-Spillman 2016	Rates of HIV and Hepatitis Infections in Clients Entering Heroin-Assisted Treatment between 2003 and 2013 and Risk Factors for Hepatitis C Infection	Switzerland	2003-2013	Cross-sectional	1	HCV	Low
Doran 2018	Substance use and homelessness among emergency department patients	USA	2016-2017	Cross-sectional analysis of cohort study	2	Opioid overdose	High
Duffy 2006	Psychiatric morbidity in the male sentenced Irish prisons population	Ireland	2001	Cross-sectional	1	Deliberate self-harm	Low
Dumais 2010	Clinical and sociodemographic profiles of male inmates with severe mental illness: a comparison with voluntarily and involuntarily hospitalized patients	Canada	1991-2003	Cross-sectional	4	Alcohol problem, antisocial personality disorder, anxiety disorder, alcohol abuse or dependence	High
Dumais 2014	Clinical characteristics and service use of incarcerated males with severe mental disorders: a comparative case-control study with patients found not criminally responsible	Canada	1998-2003	Cross-sectional analysis of case-control study	2	Alcohol abuse or dependence, suicide attempt	High
Enguelberg-Gabbay 2016	Methadone treatment, bruxism, and temporomandibular disorders among male prisoners	Israel	not stated	Cross-sectional	2	Awake bruxism, sleep bruxism	High
Epperson 2008	Increased HIV risk associated with criminal justice involvement among men on methadone	USA	not stated	Cross-sectional analysis of cohort study	1	HIV	High
Erlangsen 2012	Schizophrenia--a predictor of suicide during the second half of life?	Denmark	1990-2006	Cohort study	2	Suicide mortality	Low
Essock 2003	Risk factors for HIV, hepatitis B, and hepatitis C among persons with severe mental illness	USA	1997-1998	Cross-sectional	6	HBV, HCV, HIV	High
Estebanez 1998	A demographic and health survey of Spanish female sex workers	Spain	1989-1991	Cross-sectional	4	HIV	Low
Estebanez 2000	Women, drugs and HIV/AIDS: Results of a multicenter European study	Multiple	1995	Cross-sectional	3	HIV	Low
Estebanez 2001	Determinants of HIV prevalence amongst female IDU in Madrid	Spain	1995-1996	Cross-sectional	3	HIV	Low

Author & year	Title	Country	Study years	Design	Number of data points included	Outcomes	Risk of bias
Evans 2015	Heterogeneity of Mental Health Service Utilization and High Mental Health Service Use Among Women Eight Years After Initiating Substance Use Disorder Treatment	USA	2000-2002	Cross-sectional analysis of cohort study	4	Suicidal ideation or attempt	Low
Fairbairn 2008	Crystal methamphetamine use associated with non-fatal overdose among a cohort of injection drug users in Vancouver	Canada	1996-2005	Cohort study	1	Non-fatal overdose	High
Falster 2009	Hepatitis C virus acquisition among injecting drug users: A cohort analysis of a national repeated cross-sectional survey of needle and syringe program attendees in Australia, 1995-2004	Australia	1995-2004	Cross-sectional	1	HCV	Low
Feng 2012	Prevalence and knowledge of sexual transmitted infections, drug abuse, and AIDS among male inmates in a Taiwan prison	Taiwan	2008	Cross-sectional	9	Cellulitis, condyloma acuminata, diabetes mellitus, gonorrhoea, HBV, HCV, herpes simplex urethritis, hypertension, syphilis	High
Feodor Nilsson 2014	Suicide and unintentional injury mortality among homeless people: a Danish nationwide register-based cohort study	Denmark	1999-2008	Cohort study	23	Suicide mortality, unintentional injury mortality	Low
Fickenscher 2001	Women behind bars: health needs of inmates in a county jail	USA	1999	Cross-sectional	2	Fair/poor self-rated heatlh	Low
Finseth 2012	Risk factors related to lifetime suicide attempts in acutely admitted bipolar disorder inpatients	Norway	2002-2009	Cross-sectional	1	Suicide attempt	Low
Firestone Cruz 2007	Prevalence and associated factors of hepatitis C infection (HCV) in a multi-site Canadian population of illicit opioid and other drug users (OPICAN)	Canada	2002	Cross-sectional	2	HCV	Low
Folch 2016	High Prevalence and Incidence of HIV and HCV Among New Injecting Drug Users With a Large Proportion of Migrants--Is Prevention Failing?	Spain	2010-2011	Cross-sectional	12	HCV, HIV	Low
Ford 2000	HIV, hepatitis C and risk behaviour in a Canadian medium-security federal penitentiary. Queen's University HIV Prison Study Group	Canada	1998	Cross-sectional	1	HCV	High

Author & year	Title	Country	Study years	Design	Number of data points included	Outcomes	Risk of bias
Fowler 1998	Patterns of current and lifetime substance use in schizophrenia	Australia	not stated	Cross-sectional	2	Suicide attempt	High
Fox 2005	Hepatitis C virus infection among prisoners in the California state correctional system	USA	not stated	Cross-sectional	1	HCV	Low
Freedenthal 2007	Inhalant use and suicidality among incarcerated youth	USA	2003	Cross-sectional	8	Suicidal ideation or attempt	Low
Fruehwald 2004	Suicide in custody: case-control study	Austria	1975-1999	Case-control study	1	Suicide mortality	High
Fuller 2005	A comparison of HIV seropositive and seronegative young adult heroin- and cocaine-using men who have sex with men in New York City, 2000-2003	USA	2000-2004	Cross-sectional	1	HIV	High
Fuller 2011	Prevalence of liver disease in veterans with bipolar disorder or schizophrenia	USA	2001-2006	Cohort study	6	Alcohol-related cirrhosis, non-alcohol fatty liver disease, overall liver disease	High
Fuller-Thomson 2016	Schizophrenia and Suicide Attempts: Findings from a Representative Community-Based Canadian Sample	Canada	2012	Cross-sectional	1	Suicide attempt	High
Gabriel 2008	Prevalence of Chlamydia trachomatis and associated risk factors in women inmates admitted to a youth offenders institute in the UK	UK	2003-2005	Cross-sectional	5	Chlamydia	High
Gao 2008	Differential interactions between comorbid anxiety disorders and substance use disorder in rapid cycling bipolar I or II disorder	USA	up to 2005	Cross-sectional	16	Any anxiety disorder, generalised anxiety disorder, obsessive compulsive disorder, panic disorder	High
Garcia 2004	Association of Trichomonas vaginalis with sociodemographic factors and other STDs among female inmates in Lisbon	Portugal	Not stated	Cross-sectional	2	Trichomonas vaginalis infection	High
Garland 2009	Nitrous oxide inhalation among adolescents: prevalence, correlates, and co-occurrence with volatile solvent inhalation	USA	2004	Cross-sectional	13	Anxiety symptoms, depression symptoms, head injury with extended loss of consciousness, obsessive compulsive symptoms, suicidal ideation	Low

Author & year	Title	Country	Study years	Design	Number of data points included	Outcomes	Risk of bias
Garno 2005	Bipolar disorder with comorbid cluster B personality disorder features: impact on suicidality	USA	not stated	Cross-sectional	1	Suicide attempts	High
Gates 2004	Risk factors for hepatitis C infection and perception of antibody status among male prison inmates in the Hepatitis C Incidence and Transmission in Prisons Study cohort, Australia	Australia	2000-2001	Nested case-control study	2	HCV	Low
Gelberg 2009	Hospitalizations among homeless women: are there ethnic and drug abuse disparities?	USA	1997	Cross-sectional	5	Alcohol abuse/dependence, depression, fair or poor self-rated health, psychological wellbeing, sexually transmitted disease	Low
Gelberg 2012	Prevalence, distribution, and correlates of hepatitis C virus infection among homeless adults in Los Angeles	USA	2003-2004	Cross-sectional	9	HCV	Low
Gilchrist 2005	Comparison of drug use and psychiatric morbidity between prostitute and non-prostitute female drug users in Glasgow, Scotland	UK	not stated	Cross-sectional	4	Accidental drug overdose, suicide attempt, deliberate self-harm, eating disorder	High
Gilfillan 1998	A comparison of psychotic and nonpsychotic substance users in the psychiatric emergency room	USA	1993	Cross-sectional	1	Suicidal behaviour or ideation	High
Gjersing 2014	Gender differences in mortality and risk factors in a 13-year cohort study of street-recruited injecting drug users.	Norway	1997-2010	Cohort study	3	All-cause mortality	Low
Goldenberg 2017	Dual sexual and drug-related predictors of hepatitis C incidence among sexworkers in a Canadian setting: gaps and opportunities for scale-up of hepatitis C virus prevention, treatment, and care	Canada	2010-2014	Cohort study	3	HCV	Low
Goldstein 2005	History of suicide attempts in pediatric bipolar disorder: factors associated with increased risk	USA	not stated	Cross-sectional analysis of cohort study	1	Suicide attempt	High
Goldstein 2008	Substance use disorders among adolescents with bipolar spectrum disorders	USA	not stated	Cross-sectional analysis of cohort study	5	ADHD, anxiety, conduct disorder, oppositional defiant disorder, PTSD	High

Author & year	Title	Country	Study years	Design	Number of data points included	Outcomes	Risk of bias
Goldstein 2008	The specific burden of comorbid anxiety disorders and of substance use disorders in bipolar I disorder	USA	2001-2002	Cross-sectional	4	Mental HRQoL, personality disorder	Low
Goldstein 2008	Preliminary findings regarding overweight and obesity in pediatric bipolar disorder	USA	not stated	Cross-sectional	1	Overweight/obesity	High
Goldstein 2011	The burden of obesity among adults with bipolar disorder in the United States	USA	2001-2002	Cross-sectional	1	Obesity	Low
Gonzalez-Pinto 2006	Suicidal risk in bipolar I disorder patients and adherence to long-term lithium treatment	Spain	1994-2005	Cohort study	1	Suicidal acts	High
Gonzalez-Pinto 2007	Predictors of Suicide in First-Episode Affectiveand Nonaffective Psychotic Inpatients:Five-Year Follow-Up of Patients Froma Catchment Area in Vitoria, Spain	Spain	1997-1999	Cohort study	1	Suicide attempt	High
Gore 1999	Prevalence of hepatitis C in prisons: WASH-C surveillance linked to self-reported risk behaviours	UK	1994-1996	Cross-sectional	1	HCV	Low
Gossop 2002	A prospective study of mortality among drug misusers during a 4-year period after seeking treatment	UK	1995-1999	Cohort study	1	Overdose mortality	High
Graham 2017	Trends in hepatitis C antibody prevalence among Aboriginal and Torres Strait Islander people attending Australian Needle and Syringe Programs, 1996-2015	Australia	1996-2015	Cross-sectional	6	HCV	Low
Grinman 2010	Drug problems among homeless individuals in Toronto, Canada: prevalence, drugs of choice, and relation to health status	Canada	2004-2005	Cross-sectional	4	Mental and physical HRQoL	Low
Gutierrez-Rojas 2008	Quality of life in bipolar disorder patients: a comparison with a general population sample	Spain	not stated	Cross-sectional	2	Low mental HRQoL	High
Hadland 2014	Prescription opioid injection and risk of hepatitis C in relation to traditional drugs of misuse in a prospective cohort of street youth	Canada	not stated	Cohort study	5	HCV	Low
Haglund 2014	Suicide after release from prison: a population-based cohort study from Sweden	Sweden	2005-2009	Cohort study	2	Suicide mortality	Low
Hahn 2004	HIV seroconversion among the homeless and marginally housed in San Francisco: a ten-year study	USA	1990-2000	Repeated cross-sectional studies	2	HIV	Low

Author & year	Title	Country	Study years	Design	Number of data points included	Outcomes	Risk of bias
Hakansson 2013	All cause mortality in criminal justice clients with substance use problems - a prospective follow-up study	Sweden	2001-2008	Cohort study	1	All-cause mortality	Low
Hall 2000	Pathological gambling among cocaine-dependent outpatients	USA	not stated	Cross-sectional analysis of interventional study	1	Pathological gambling	High
Han 2003	Lifetime and current prevalence of mental disorders among homeless men in Korea	South Korea	1999-2000	Cross-sectional	2	Mood disorders	Low
Harrison 2008	Naturalistic follow-up of comorbid substance use in schizophrenia: The West London first-episode study	UK	not stated	Cohort study	5	Akasthisia, depression, mild dyskinesia, Parkinsonism, tardive dyskinesia,	High
Haussig 2018	A large proportion of people who inject drugs are susceptible to hepatitis B: results from a bio-behavioural study in eight German cities	Germany	2011-2014	Cross-sectional	2	HBV	High
Havens 2006	Prevalence and correlates of suicidal ideation among young injection vs noninjection drug users	USA	2000-2002	Cross-sectional	1	Suicidal ideation	High
Hellard 2004	The prevalence and the risk behaviours associated with the transmission of hepatitis C virus in Australian correctional facilities	Australia	2001	Cross-sectional	2	HCV	Low
Helleberg 2015	Associations between HIV and schizophrenia and their effect on HIV treatment outcomes	Denmark	1995-2011	Cohort study	1	HIV	Low
Hennessey 2009	Hepatitis A seroprevalence and risk factors among homeless adults in San Francisco: should homelessness be included in the risk-based strategy for vaccination?	USA	1999-2000	Cross-sectional	3	HAV	Low
Herman 2004	Neurocognitive functioning and quality of life among dually diagnosed and non-substance abusing schizophrenia inpatients	Australia	not stated	Cross-sectional	1	Quality of life	High
Hermanstyne 2012	The association between use of non-injection drug implements and hepatitis C virus antibody status in homeless and marginally housed persons in San Francisco	USA	1999-2000	Cross-sectional	6	HCV	Low

Author & year	Title	Country	Study years	Design	Number of data points included	Outcomes	Risk of bias
Heslin 2013	Explaining differences in hepatitis C between U.S. veterans and nonveterans in treatment for substance abuse: results from a regression decomposition	USA	2006-2010	Cross-sectional	1	HCV	High
Hickman 2007	Hepatitis C virus (HCV) prevalence, and injecting risk behaviour in multiple sites in England in 2004	UK	2003-2004	Cross-sectional	3	HCV	1 datapoint scored as low; 2 datapoints scored as high
Hjorthoj 2015	Association between alcohol and substance use disorders and all-cause and cause-specific mortality in schizophrenia, bipolar disorder, and unipolar depression	Denmark	1990-2012 (for mortality analyses)	Cohort study	12	All-cause mortality and cause-specific mortality (cardiovascular, suicide, accidents, malignant tumours other than respiratory organs, digestive organs, respiratory organs)	Low
Holohan 2000	Health and homelessness in Dublin	Ireland	1997	Cross-sectional	1	Psychiatric disease	High
Hope 2010	The extent of injection site infection in injecting drug users: findings from a national surveillance study	UK	2006-2008	Cross-sectional	3	Abscess, sore, or open wound	High
Hope 2012	Hepatitis C infection among recent initiates to injecting in England 2000-2008: is a national hepatitis C action plan mking a difference	UK	2000-2008	Cross-sectional	1	HCV	High
Howard 2008	Inhalant use among incarcerated adolescents in the United States: prevalence, characteristics, and correlates of use	USA	2004	Cross-sectional	3	Diagnosis of mental illness, head injury, suicide ideation	Low
Huckans 2006	Management of hepatitis C disease among VA patients with schizophrenia and substance use disorders	USA	1998-2003	Cross-sectional	1	HCV	High
Hwang 1998	Risk factors for death in homeless adults in Boston	USA	1988-1993	Case-control study	5	All-cause mortality	Low
Hwang 2006	Relationship of cosmetic procedures and drug use to hepatitis C and hepatitis B virus infections in a low-risk population	USA	2000-2001	Cross-sectional	1	HCV	High

Author & year	Title	Country	Study years	Design	Number of data points included	Outcomes	Risk of bias
Hwang 2008	The effect of traumatic brain injury on the health of homeless people	Canada	2004-2005	Cross-sectional	1	Traumatic brain injury	Low
Jablensky 2000	Psychotic disorders in urban areas: an overview of the Study on Low Prevalence Disorders	Australia	1997	Cross-sectional	1	Alcohol abuse	High
Jarbin 2004	Suicide and suicide attempts in adolescent-onset psychotic disorders	Sweden	1982-1993	Cohort study	1	Suicide attempt	Low
Jaworski 2011	Presence of co-morbid substance use disorder in bipolar patients worsens their social functioning to the level observed in patients with schizophrenia	France	2004-2005	Cross-sectional	2	Suicide attempt	High
Jenkins 2005	Psychiatric and social aspects of suicidal behaviour in prisons	UK	1997	Cross-sectional	8	Suicidal attempts	Low
Jin 2014	Differences in HIV risk behaviors among people who inject drugs by gender and sexual orientation, San Francisco, 2012	USA	2012	Cross-sectional	3	HIV	Low
John 2009	Prevalence of metabolic syndrome among Australians with severe mental illness	Australia	2005-2006	Cross-sectional	2	Metabolic syndrome	High
Johnson 2006	Concurrent drug and alcohol dependency and mental health problems among incarcerated women	Australia	not stated	Cross-sectional	1	Mental health problems	Low
Jones 2015	Mortality from treatable illnesses in marginally housed adults: a prospective cohort study	Canada	2008-2012	Cohort study	4	All-cause mortality	Low
Judd 2005	Prevalence of, and risk factors for, hepatitis C virus infection among recent initiates to injecting in London and Glasgow: cross sectional analysis	UK	2001-2002	Cross-sectional	1	HCV	High
Kaba 2010	Hepatitis E virus infection in sheltered homeless persons, France	France	2003,2005, 2006	Cross-sectional	1	HEV	High
Kamali 2000	The prevalence of comorbid substance misuse and its influence on suicidal ideation among in-patients with schizophrenia	Ireland	not stated	Cross-sectional	4	Depression, suicidal ideation	Low
Kavanagh 2004	Demographic and clinical correlates of comorbid substance use disorders in psychosis: multivariate analyses from an epidemiological sample	Australia	1997-1998	Cross-sectional	2	Alcohol abuse or dependence, cannabis abuse or dependence	High

Author & year	Title	Country	Study years	Design	Number of data points included	Outcomes	Risk of bias
Keaney 2011	Physical health problems among patients seeking treatment for substance use disorders: A comparison of drug dependent and alcohol dependent patients	UK	Not stated	Cross-sectional	2	Physical health	High
Kelly 2000	Risk behaviors and the prevalence of Chlamydia in a juvenile detention facility	USA	not stated	Cross-sectional	3	Chlamydia	Low
Kemp 1999	Injecting drug users have high rates of hepatitis C, but HIV infection remains uncommon	New Zealand	1997	Cross-sectional	1	HCV	Low
Kerfoot 2011	Substance use and schizophrenia: Adverse correlates in the CATIE study sample	USA	2001-2004	Cross-sectional analysis of interventional study	2	Depression	High
Kerr 2007	Predictors of non-fatal overdose among a cohort of polysubstance-using injection drug users	Canada	1996-2004	Cohort study	1	Non-fatal overdose	High
Kerr 2016	Sex work and HIV incidence among people who inject drugs	Canada	1996-2012	Cohort study	2	HIV	Low
Kertesz 2005	Homeless chronicity and health-related quality of life trajectories among adults with addictions	USA	1997-1999	Cross-sectional analysis of interventional study	8	Mental HRQoL, physical HRQoL, suicidal ideation or attempt	Low
Khan 2005	Ongoing transmission of hepatitis B virus infection among inmates at a state correctional facility	USA	2000-2001	Repeated cross-sectional studies	1	HBV	High
Khan 2015	STI/HIV Sexual Risk Behavior and Prevalent STI Among Incarcerated African American Men in Committed Partnerships: The Significance of Poverty, Mood Disorders, and Substance Use	USA	2011-2014	Cross-sectional analysis of cohort study	5	Sexually transmitted infections	Low
Kilbourne 2009	Co-occurring conditions and health-related quality of life in patients with bipolar disorder	USA	2004-2006	Cohort study	2	Mental or physical HRQoL	Low
Kim 2009	Use of sentinel surveillance and geographic information systems to monitor trends in HIV prevalence, incidence, and related risk behavior among women undergoing syphilis screening in a jail setting	USA	1999-2001	Cross-sectional	3	HIV	Low
Kimber 2010	Survival and cessation in injecting drug users: prospective observational study of outcomes and effect of opiate substitution treatment	UK	1980-2007	Cohort study	3	All-cause mortality	Low

Author & year	Title	Country	Study years	Design	Number of data points included	Outcomes	Risk of bias
Kinner 2012	Incidence and risk factors for non-fatal overdose among a cohort of recently incarcerated illicit drug users	Canada	1996-2010	Cohort study	2	Non-fatal overdose	High
Kottiri 2005	A community-based study of hepatitis B infection and immunization among young adults in a high-drug-use neighborhood in New York City	USA	1997-2000	Cross-sectional	1	HBV	Low
Krabbendam 2015	Personality disorders in previously detained adolescent females: A prospective study	Netherlands	2002-2010	Cohort study	3	Antisocial personality disorder, borderline personality disorder, both	Low
Krupski 2015	Comparison of Homeless and Non-Homeless Problem Drug Users Recruited from Primary Care Safety-Net Clinics	USA	2009-2012	Cross-sectional analysis of interventional study	16	Diagnosis of blood disorder, cancer, cardiovascular disease, cerebrovascular disease, diabetes, ear disorder, eye disorder, gastrointestinal disorder, genital disorder, infection, metabolic disorder, nervous system disease, pulmonary, renal, skeletal problem, skin	High
Kuo 2004	Hepatitis B virus infection and vaccination among young injection and non-injection drug users: missed opportunities to prevent infection	USA	1999-2002	Cross-sectional analysis of cohort study	3	HBV	High
Kuo 2005	Risk factors for completed suicide in schizophrenia	Taiwan	1985-2000	Case-control study	1	Suicide	High
Kuyper 2005	The prevalence and incidence of sexually transmitted infections in a prospective cohort of injection drug users in Vancouver, British Columbia	Canada	1996-2003	Cohort study	6	STI	High
Langendam 1999	Methadone maintenance treatment modalities in relation to incidence of HIV: results of the Amsterdam cohort study	Netherlands	1985-1996	Cohort study	2	HIV	High
Laporte 2017	Deliberate self-harm behavior among young violent offenders	Sweden	2010-2012	Cross-sectional	5	Deliberate self-harm	High

Author & year	Title	Country	Study years	Design	Number of data points included	Outcomes	Risk of bias
Laslett 2008	The oral health of street-recruited injecting drug users: prevalence and correlates of problems	Australia	2002	Cross-sectional	1	Dental problems	High
Latimer 2007	Prevalence and correlates of hepatitis A among adult drug users: The significance of incarceration and race/ethnicity	USA	not stated	Cross-sectional analysis of cohort study	2	HAV	Low
Latka 2001	Prevalence, incidence, and correlates of chlamydia and gonorrhoea among young adult injection drug users	USA	1997-1999	Cross-sectional analysis of cohort study	6	Chlamydia, gonorrhoea	Low
LaTorre 2007	Socio-demographic determinants of coinfections by HIV, hepatitis B and hepatitis C viruses in central Italian prisoners	Italy	1995-2000	Cross-sectional	3	HBV/HCV coinfection, HIV/HBV coinfection, HIV/HCV coinfection	Low
Lim 2012	Risks of drug-related death, suicide, and homicide during the immediate post-release period among people released from New York City jails, 2001-2005	USA	2001-2005	Cohort study	3	All-cause and cause-specific mortality (homicide, suicide, drug-related death)	Low
Lim 2015	All-cause, drug-related, and HIV-related mortality risk by trajectories of jail incarceration and homelessness among adults in New York City	USA	2001-2005	Cohort study	18	All-cause and cause-specific mortality (drug-related death, HIV)	Low
Limosin 2007	Ten-year prospective follow-up study of the mortality by suicide in schizophrenic patients	France	1993-2002	Cohort study	1	All-cause mortality	Low
Lin 2010	Prevalence and determinants of hepatitis B antigenemia in 15,007 inmates in Taiwan	Taiwan	1994	Cross-sectional	1	HBV	Low
Lize 2015	Violent Death Rates and Risk for Released Prisoners in North Carolina	USA	2006-2007	Cohort study	2	Mortality from homicide or suicide	Low
Lloyd-Smith 2005	Prevalence and correlates of abscesses among a cohort of injection drug users	Canada	1996-2004	Cross-sectional analysis of cohort study	3	Abscess	Low
Lloyd-Smith 2008	Risk factors for developing a cutaneous injection-related infection among injection drug users: a cohort study	Canada	2004-2005	Cohort study	2	Cutaneous injecting-related infection	Low
Long 2001	Prevalence of antibodies to hepatitis B, hepatitis C, and HIV and risk factors in entrants to Irish prisons: a national cross sectional survey	Ireland	1999	Cross-sectional	3	HIV, HBV, HCV	Low
Lopez 2001	Suicide attempts in bipolar patients	Spain	1994-1996	Cross-sectional	1	Suicide attempts	High

Author & year	Title	Country	Study years	Design	Number of data points included	Outcomes	Risk of bias
Lopez-Zetina 2001	Prevalence of HIV and hepatitis B and self-reported injection risk behavior during detention among street-recruited injection drug users in Los Angeles County, 1994-1996	USA	1994-1996	Cross-sectional	1	HBV	Low
Lozano 2017	Psychiatric comorbidity and severity of dependence on substance users: how it impacts on their health-related quality of life?	Spain	not stated	Cross-sectional	1	HRQoL	High
Lukasiewicz 2007	Prevalence and factors associated with alcohol and drug-related disorders in prison: a French national study	France	2003-2004	Cross-sectional	7	Alcohol abuse/dependence, antisocial personality disorder, anxiety disorder, history of suicide attempt, mood disorder	Low
Lumme 2016	Excess Mortality in Patients with Severe Mental Disorders in 1996-2010 in Finland	Finland	1990-2010	Cohort study	50	All-cause mortality and cause-specific mortality (alcohol, amenable, disease, IHD)	Low
Macalino 2004	Prevalence and incidence of HIV, hepatitis B virus, and hepatitis C virus infections among males in Rhode Island prisons	USA	1998-2000	Cohort study	12	HBV, HCV, HIV	Low
Macalino 2005	A missed opportunity: hepatitis C screening of prisoners	USA	1998-200	Cross-sectional	2	HCV	High
Mackelprang 2014	Adverse outcomes among homeless adolescents and young adults who report a history of traumatic brain injury	USA	2006 & 2009	Cross-sectional	6	Traumatic brain injury	High
Maden 2000	Deliberate self-harm in sentenced male prisoners in England and Wales: Some ethnic factors	UK	1988-1989	Cross-sectional	1	Deliberate self-harm	Low
Mahajan 2013	Hepatitis E virus among persons who inject drugs, San Diego, California, USA, 2009-2010	USA	2009-2010	Cross-sectional	3	HEV	High
Maher 2004	Risk behaviors and antibody hepatitis B and C prevalence among injecting drug users in south-western Sydney, Australia	Australia	1999-2002	Cross-sectional analysis of cohort study	1	HCV	Low
Mannocci 2014	A cross sectional study on dermatological diseases among male prisoners in southern Lazio, Italy	Italy	1995-2000	Cross-sectional	3	Mycosis, non-specific dermatitis, psoriasis	Low

Author & year	Title	Country	Study years	Design	Number of data points included	Outcomes	Risk of bias
Mansson 2000	Continued transmission of hepatitis B and C viruses, but no transmission of human immunodeficiency virus among intravenous drug users participating in a syringe/needle exchange program	Sweden	1990-1993	Cohort study	1	HBV, HCV	High
March 2007	Factors associated with reported hepatitis C and HIV among injecting drug users in ten European cities	Multiple	1998-2000	Cross-sectional	8	HCV, HIV	4 data points scored as low risk; 4 data points scored as high
Marco 2012	Prevalence of latent tuberculosis infection in inmates recently incarcerated in a men's prison in Barcelona	Spain	2009	Cross-sectional	2	Latent TB	Low
Marco 2014	Incidence of hepatitis C infection among prisoners by routine laboratory values during a 20-year period	Spain	1992-2012	Cohort study	1	HCV	Low
Margolese 2004	Drug and alcohol use among patients with schizophrenia and related psychoses: levels and consequences	Canada	not stated	Cross-sectional analysis of cohort study	1	Depression	Low
Margolese 2006	A 12-month prospective follow-up study of patients with schizophrenia-spectrum disorders and substance abuse: changes in psychiatric symptoms and substance use	Canada	not stated	Cross-sectional	1	Depression	High
Marshall 2008	High prevalence of HIV infection among homeless and street-involved Aboriginal youth in a Canadian setting	Canada	2005-2006	Cross-sectional analysis of cohort study	3	HIV	High
Martin 2005	Drug use and risk of bloodborne infections: a survey of female prisoners in British Columbia	Canada	2001	Cross-sectional	3	HCV	2 datapoints scored as low; 1 datapoint scored as high
Marzano 2010	Psychiatric disorders in women prisoners who have engaged in near-lethal self-harm: case-control study	UK	2007-2008	Case-control study	2	Near-lethal suicide attempt	Low
Mathei 2005	Prevalence of hepatitis C in drug users in Flanders: determinants and geographic differences	Belgium	1999-2000	Cross-sectional	1	HCV	Low

Author & year	Title	Country	Study years	Design	Number of data points included	Outcomes	Risk of bias
Matthews 2008	Hepatitis C testing and infection rates in bipolar patients with and without comorbid substance use disorders	USA	1998-2004	Cross-sectional	1	HCV	High
Mazza 2009	Clinical features, response to treatment and functional outcome of bipolar disorder patients with and without co-occurring substance use disorder: 1-year follow-up	Italy	2006-2007	Cross-sectional analysis of cohort study	12	Antisocial personality disorder, anxiety, avoidant personality disorder, borderline personality disorder, dependent personality disorder, histrionic personality disorder, narcisstic personality disorder, obsessive compulsive personality disorder, paranoid personality disorder, quality of life, schizoid personality disorder, schizotypal personality disorder	High
McDonnell 1998	Health risk profile of prostitutes in Dublin	Ireland	1991-1997	Cross-sectional	3	HBV, HCV, HIV	Low
McDonnell 2009	Risk factors for Chlamydia among young women in a northern california juvenile detention facility: implications for community intervention	USA	2002-2005	Cross-sectional	5	Chlamydia	High
McIntyre 2007	Substance use disorders and overweight/obesity in bipolar I disorder: preliminary evidence for competing addictions	Canada	2002	Cross-sectional	1	Overweight/obesity	Low
Miller 2002	Opportunities for prevention: hepatitis C prevalence and incidence in a cohort of young injection drug users	Canada	1996-?	Cohort study	1	HCV	High
Miller 2006	Binge drug use independently predicts HIV seroconversion among injection drug users: implications for public health strategie	Canada	1996-2003	Cohort study	1	HIV	Low
Miller 2007	The social epidemiology of HIV transmission among African American women who use drugs and their social network members	USA	2002-2004	Cross-sectional analysis of cohort study	3	HIV	High

Author & year	Title	Country	Study years	Design	Number of data points included	Outcomes	Risk of bias
Miller 2007	Factors associated with premature mortality among young injection drug users in Vancouver	Canada	1996-2004	Cohort study	2	All-cause mortality	Low
Miller 2008	HIV, the clustering of sexually transmitted infections, and sex risk among African American women who use drugs	USA	2003-2005	Cross-sectional	2	HIV	High
Miller 2009	Markers and risk factors for HCV, HBV and HIV in a network of injecting drug users in Melbourne Australia	Australia	2005-2007	Cross-sectional	9	HBV, HCV, HIV	4 data points scored as low risk; 5 data points scored as high
Miller 2013	Quality of life among patients with bipolar disorder in primary care versus community mental health settings	USA	not stated	Cross-sectional analysis of interventional study	4	Mental and physical HRQoL	High
Milloy 2008	Non-fatal overdose among a cohort of active injection drug users recruited from a supervised injection facility	Canada	2003-2005	Cohort study	2	Non-fatal overdose	1 data point scored as low; 1 data point scored as high
Milloy 2010	Recent incarceration linked to cutaneous injection-related infections among active injection drug users in a Canadian setting	Canada	2004-2006	Cohort study	1	Cutaneous injecting-related infection	Low
Mitra 2015	Drug use patterns predict risk of non-fatal overdose among street-involved youth in a Canadian setting	Canada	2005-2012	Cohort study	2	Non-fatal overdose	Low
Mohamed 2013	Dual diagnosis among intensive case management participants in the veterans health administration: Correlates and outcomes	USA	2000-2009	Cross-sectional analysis of interventional study	6	Quality of life, suicidal act, suicidal ideation	High
Mooney 2002	Perceived quality of life and mental health status of Irish female prisoners	Ireland	not stated	Cross-sectional	1	Quality of life	High
Moore 2013	Non-fatal overdose among adult prisoners with a history of injecting drug use in two Australian states	Australia	2008-2010	Cross-sectional	2	Non-fatal overdose	Low
Moran 2004	The correlates of comorbid antisocial personality disorder in schizophrenia	Multiple	not stated	Cross-sectional	1	Antisocial personaltiy disorder	High

Author & year	Title	Country	Study years	Design	Number of data points included	Outcomes	Risk of bias
Morrison 2009	Homelessness as an independent risk factor for mortality: results from a retrospective cohort study	UK	2000-2005	Cohort study	1	Drug-related death	High
Mueser 2004	Interpersonal trauma and post-traumatic stress disorder in patients with severe mental illness: demographic, clinical, and health correlates	USA	not stated	Cross-sectional	8	PTSD	Low
Murray 2003	Prevalence of hepatitis C virus infection and risk factors in an incarcerated juvenile population: a pilot study	USA	1999-2001	Cross-sectional	1	HCV	High
Naeem 2005	Cognitive behaviour therapy for schizophrenia in patients with mild to moderate substance misuse problems	UK	not stated	Cross-sectional analysis of interventional study	4	Anxiety, depression	High
Nambiar 2015	Mortality in the Melbourne injecting drug user cohort study (MIX)	Australia	2008-2012	Cohort study	3	All-cause mortality	Low
Neale 2004	Measuring the health of Scottish drug users	UK	2001-2002	Cross-sectional analysis of cohort study	16	Self-rated health	High
Neria 2002	Trauma exposure and posttraumatic stress disorder in psychosis: findings from a first-admission cohort	USA	not stated	Cross-sectional analysis of cohort study	1	PTSD	High
Nerlander 2017	Exchange Sex and HIV Infection Among Women Who Inject Drugs-20 US Cities, 2009	USA	2009	Cross-sectional	2	HIV	Low
Ng 2016	Posttraumatic stress disorder symptoms and social and occupational functioning of people with schizophrenia	USA	1999-2002	Cross-sectional	3	PTSD	High
Nielsen 2011	Psychiatric disorders and mortality among people in homeless shelters in Denmark: a nationwide register-based cohort study	Denmark	1999-2009	Cohort study	6	All-cause mortality	Low
Nijhawan 2012	Prevalence and predictors of trichomonas infection in newly incarcerated women	USA	2009-2011	Cross-sectional	8	Trichomonas vaginalis infection	High
Nikoo 2017	Incidence and Associated Risk Factors of Traumatic Brain Injury in a Cohort of Homeless and Vulnerably Housed Adults in 3 Canadian Cities	Canada	2009-2012	Cohort study	1	Traumatic brain injury	Low
Noell 2001	Incidence and prevalence of chlamydia, herpes, and viral hepatitis in a homeless adolescent population	USA	1994-1997	Cohort study	4	Chlamydia, herpes simplex virus 2 infection, HBV, HCV	Low

Author & year	Title	Country	Study years	Design	Number of data points included	Outcomes	Risk of bias
Nordentoft 2003	10 Year follow up study of mortality among users of hostels for homeless people in Copenhagen	Denmark	1992-2002	Cohort study	4	All-cause mortality	Low
Noska 2016	History of sex exchange in women with a history of incarceration	USA	2009-2011	Cross-sectional	2	Sexually transmitted infections	Low
Nower 2015	Problem gambling and homelessness: results from an epidemiologic study	USA	not stated	Cross-sectional analysis of cohort study	3	Problem gambling	Low
Nyamathi 2002	Risk factors for hepatitis C virus infection among homeless adults	USA	1995-1999	Cross-sectional	2	HCV	Low
Nyamathi 2006	Hepatitis C virus infection among homeless men referred from a community clinic	USA	2002-2003	Case-control study	5	HCV	Low
Nyhlen 2011	Substance abuse and psychiatric co-morbidity as predictors of premature mortality in Swedish drug abusers: a prospective longitudinal study 1970-2006	Sweden	1970-2007	Cohort study	1	Drug-related death	Low
O'Brien 2015	Health, perceived quality of life and health services use among homeless illicit drug users	Ireland	2011	Cross-sectional	46	AIDS, accidental overdose, anaemia, angina, anxiety, athritis, asthma, back problems, better quality of life, COPD, cancer, chest infection or bronchitis, DVT, dental problems, depression and/or anxiety, diabetes, eczema, epilepsy, eye problems, foot problems, good/excellent self-rated physical health, HIV, heart attack, heart disease, HBV, HCV, hypertension, joint problems, kidney problems, migraine/regular severe headaches, other GI	High

Author & year	Title	Country	Study years	Design	Number of data points included	Outcomes	Risk of bias
						tract disorder, pulmonary embolism, peptic ulcer disease, problem alcohol drinking, psoriasis, sexually transmitted infection, skin problems, skin ulcers, stroke, suicidal feelings, suicide attempt, tuberculosis	
Ochoa 2005	Heroin overdose among young injection drug users in San Francisco	USA	2000-2001	Case-control study	5	Overdose	High
O'Driscoll 2001	Predictors of accidental fatal drug overdose among a cohort of injection drug users	USA	1994-1997	Cohort study	1	Overdose mortality	High
Ogilvie 1999	Hepatitis infection among adolescents resident in Melbourne Juvenile Justice Centre: risk factors and challenges	Australia	1996	Cross-sectional	3	HAV, HBV, HCV	Low
Onyeka 2016	Association between routes of drug administration and all-cause mortality among drug users	Finland	1997-2010	Cohort study	1	All-cause mortality	Low
Ortiz 2014	Oral human papillomavirus infection among drug users in Puerto Rico	USA	2008-2010	Cross-sectional	3	Oral HPV infection	High
Ouellet-Plamondon 2017	Specific impact of stimulant, alcohol, and cannabis use disorders on first-episode psychosis: 2-year functional and symptomatic outcomes	Canada	2005-2010	Cross-sectional analysis of cohort study	4	Depression, quality of life	Low
Painter 2018	High inpatient utilization among Veterans Health Administration patients with substance-use disorders and co-occurring mental health conditions	USA	2011-2012	Cross-sectional analysis of cohort study	1	Chronic pain	Low

Author & year	Title	Country	Study years	Design	Number of data points included	Outcomes	Risk of bias
Pallas 1999	Risk factors for monoinfections and coinfections with HIV, hepatitis B and hepatitis C viruses in northern Spanish prisoners	Spain	1992-1994	Cross-sectional	4	HBV, HCV, HBV-HCV coinfection, HIV-HBV-HCV coinfection	Low
Palmateer 2017	Association between universal hepatitis b prison vaccination, vaccine uptake and hepatitis b infection among people who inject drugs	UK	1993-2014	Cross-sectional	2	HBV	High
Pan 2016	Three-year mortality in relation to early hospitalization and number of outpatient clinic visits in people with newly diagnosed bipolar disorder	Taiwan	2008-2011	Cohort study	1	All-cause mortality	Low
Pavarin 2008	Cocaine consumption and death risk: a follow-up study on 347 cocaine addicts in the metropolitan area of Bologna	Italy	1989-2006	Cohort study	2	All-cause mortality	Low
Pena-Orellana 2011	Prevalence of HCV risk behaviors among prison inmates: tattooing and injection drug use	USA	2004-2005	Cross-sectional	2	HCV	Low
Peng 2011	A case-control study of HIV infection among incarcerated female drug users: impact of sharing needles and having drug-using sexual partners	Taiwan	2007	Case-control study	1	HIV	Low
Perron 2008	Prevalence and correlates of traumatic brain injury among delinquent youths	USA	2003	Cross-sectional	3	Traumatic brain injury	Low
Perugi 2013	Attention deficit/hyperactivity disorder symptoms in Italian bipolar adult patients: a preliminary report	Italy	not stated	Cross-sectional	3	ADHD	High
Perugi 2015	General medical conditions in 347 bipolar disorder patients: clinical correlates of metabolic and autoimmune-allergic diseases	Italy	not stated	Cross-sectional	2	Autoimmune/allergic diseases, metabolic disease	High
Piselli 2009	Co-occurring psychiatric and substance use disorders among male detainees in Italy	Italy	2005-2006	Cross-sectional	4	Suicide attempt, HBV, HCV	High
Piselli 2015	Psychiatric needs of male prison inmates in Italy	Italy	2010-2011	Cross-sectional	4	Anxiety disorder, mood disorder, personality disorder	1 data point scored as low; 3 data points scored as high

Author & year	Title	Country	Study years	Design	Number of data points included	Outcomes	Risk of bias
Platt 2011	Risk of sexually transmitted infections and violence among indoor-working female sex workers in London: the effect of migration from Eastern Europe	UK	2008-2009	Cross-sectional	1	HIV, syphilis, Chlamydia, gonorrhoea	Low
Pliszka 2000	Affective disorder in juvenile offenders: A preliminary study	USA	not stated	Cross-sectional	4	Alcohol dependence, conduct disorder, major depressive disorder, marijuana dependence	Low
Pluck 2013	Self-harm and homeless adults	UK	not stated	Cross-sectional	2	Self-harm	High
Ponizovksy 2015	Trends in dual diagnosis of severe mental illness and substance use disorders, 1996-2010, Israel	Israel	1996-2010	Cross-sectional	1	Suicide attempt	High
Potvin 2006	Increased extrapyramidal symptoms in patients with schizophrenia and a comorbid substance use disorder	Canada	not stated	Cross-sectional	1	Extrapyramidal symptoms	High
Poulin 2007	Prevalence of HIV and hepatitis C virus infections among inmates of Quebec provincial prisons	Canada	2003	Cross-sectional	4	HCV, HIV	Low
Poulin 2018	Tattooing in prison: a risk factor for HCV infection among inmates in the Quebec's provincial correctional system	Canada	not stated	Cross-sectional	4	HCV	Low
Preti 2009	Risk of suicide and suicidal ideation in psychosis: Results from an Italian multi-modal pilot program on early intervention in psychosis	Italy	not stated	Cross-sectional analysis of cohort study	2	Suicide attempt	High
Prigerson 2003	Suicidal ideation and suicide attempts in homeless mentally ill persons: age-specific risks of substance abuse	USA	1994-1996	Cross-sectional analysis of interventional study	2	Suicidal ideation, suicide attempt	High
Rash 2016	A retrospective and prospective analysis of trading sex for drugs or money in women substance abuse treatment patients	USA	not stated	Cross-sectional	1	HIV	High
Reekie 2014	Trends in prevalence of HIV infection, hepatitis B and hepatitis C among Australian prisoners - 2004, 2007, 2010	Australia	2004-2010	Repeated cross-sectional studies	4	HBV, HCV, HIV	Low
Reininghaus 2015	Mortality in schizophrenia and other psychoses: a 10 year follow-up of the AESOP first-episode cohort	UK	end 2012, start not stated	Cohort study	3	All-cause mortality, mortality from "natural causes" (ICD A00-Q99), mortality from	Low

Author & year	Title	Country	Study years	Design	Number of data points included	Outcomes	Risk of bias
						"unnatural causes" (U50.9, V01-Y89)	
Reyes 2006	Prevalence and correlates of hepatitis C virus infection among street-recruited injection drug users in San Juan, Puerto Rico	USA	not stated	Cross-sectional	2	HCV	Low
Reyes-Uruena 2015	Cross sectional study of factors associated to self-reported blood-borne infections among drug users	Spain	not stated	Cross-sectional	1	HIV	High
Reynolds 2003	Using the quality of well-being scale to assess quality of life in out-of-treatment drug users	USA	Not stated	Cross-sectional	1	Quality of life	High
Rhoades 2011	The social context of homeless men's substance use	USA	not stated	Cross-sectional	1	Binge drinking	Low
Rhoades 2014	Prescription drug misuse among homeless youth	USA	2012-2013	Cross-sectional analysis of cohort study	3	Depression, PTSD, suicidal ideation	High
Ribe 2015	Long-term Risk of Dementia in Persons With Schizophrenia: A Danish Population-Based Cohort Study	Denmark	1995-2013	Cohort study	1	Dementia	Low
Rich 1999	Prevalence and incidence of HIV among incarcerated and reincarcerated women in Rhode Island	USA	1992-1996	Nested case-control study	2	HIV	High
Riddell 2006	The relationship between amphetamine use, crime, and psychiatric disorder among prisoners in New South Wales	Australia	not stated	Cross-sectional	2	Anxiety, depression	High
Rivlin 2010	Psychiatric disorders in male prisoners who made near-lethal suicide attempts: case-control study	UK	not stated	Case-control study	3	Near-lethal suicide attempt	High
Robinson 2009	Prevalence and predictors of suicide attempt in an incidence cohort of 661 young people with first episode psychosis	Australia	1998-2000	Cohort study	2	Suicide attempt	High
Rodriguez 2008	GC-MS confirmation of xylazine (Rompun), a veterinary sedative, in exchanged needles	USA	2005-2007	Cross-sectional	1	Skin uclers	Low
Rondinelli 2009	Young adult injection drug users in the United States continue to practice HIV risk behaviors	USA	2002-2004	Cross-sectional analysis of interventional study	3	HIV	Low

Author & year	Title	Country	Study years	Design	Number of data points included	Outcomes	Risk of bias
Rosen 2009	Characteristics and behaviors associated with HIV infection among inmates in the North Carolina prison system	USA	2004-2006	Cross-sectional	2	HIV	High
Rosenberg 2005	Hepatitis C virus and HIV co-infection in people with severe mental illness and substance use disorders.	USA	1997-1998	Cross-sectional	1	HIV-HCV co-infection	High
Rotily 1999	Prevalence of HCV and HIV antibodies and related risk factors among entrants to the main southeastern French prison	France	1995	Cross-sectional	2	HCV, HIV	Low
Rotily 2001	Surveillance of HIV infection and related risk behaviour in European prisons. A multicentre pilot stdy	Multiple	1996-1997	Cross-sectional	3	HCV, HIV, jaundice/hepatitis	Low
Roux 2014	HCV seropositivity in inmates and in the general population: an averaging approach to establish priority prevention interventions	France	2004-2010	Cross-sectional	3	HCV	Low
Roy 2007	High hepatitis C prevalence and incidence among Canadian intravenous drug users	Canada	1997-2003	Cohort study	1	HCV	Low
Roy 2011	Temporal changes in risk factors associated with HIV seroconversion among injection drug users in eastern central Canada	Canada	1995-2009	Cohort study	2	HIV	Low
Russi 2003	Sexual transmission of hepatitis B virus, hepatitis C virus, and Human Immunodeficiency Virus type 1 infections among male transvestite comercial sex workers in Montevideo, Uruguay	Uruguay	1999	Cross-sectional	6	HBV, HCV, HIV	Low
Ruutel 2014	Prevalence of IGRA-positivity and risk factors for tuberculosis among injecting drug users in Estonia and Latvia	Multiple	2007	Cross-sectional	1	Latent TB	High
Saddichha 2014	Homeless and incarcerated: an epidemiological study from Canada	Canada	2009	Cross-sectional	1	Depressive disorder	High
Sailas 2006	The mortality of young offenders sentenced to prison and its association with psychiatric disorders: a register study	Finland	1984-2002	Cohort study	2	All-cause mortality	Low
Sakelliadis 2010	Self-injurious behaviour among Greek male prisoners: prevalence and risk factors	Greece	not stated	Cross-sectional	1	Self-injurious behaviour	Low

Author & year	Title	Country	Study years	Design	Number of data points included	Outcomes	Risk of bias
Salmon 2009	Injecting-related injury and disease among clients of a supervised injecting facility	Australia	2001-2007	Cross-sectional	2	Injecting related injury or disease (abscesses or skin infections, endocarditis, septicaemia, thrombosis)	Low
Sara 2013	Differences in associations between cannabis and stimulant disorders in first admission psychosis	Australia	2005-2012	Cross-sectional	1	Alcohol disorder	Low
Sara 2014	Stimulant and other substance use disorders in schizophrenia: prevalence, correlates, and impacts in a population sample	Australia	2000-2011	Cohort study	6	Cardiac disease, infectious disease, self-harm and injury	Low
Sato 2004	Cincinnati criteria for mixed mania and suicidality in patients with acute mania	Germany	1980-1997	Cross-sectional	1	Suicidal ideation	High
Savilla 2008	Relationships between cognitive deficits, symptoms and quality of life in schizophrenia	Australia	not stated	Cross-sectional	2	Quality of life	High
Scheller-Gilkey 2002	Increased early life stress and depressive symptoms in patients with comorbid substance abuse and schizophrenia	USA	not stated	Cross-sectional	1	Depression	High
Scheller-Gilkey 2003	Relationship of clinical symptoms and substance use in schizophrenia patients on conventional versus atypical antipsychotics	USA	not stated	Cross-sectional	2	Depression, extra-pyramidal symptoms	High
Scheller-Gilkey 2004	Early life stress and PTSD symptoms in patients with comorbid schizophrenia and substance abuse	USA	1999-2000	Cross-sectional	1	Depression	High
Scherbaum 2005	Prevalence and risk factors of syphilis infection among drug addicts	Germany	2000-2001	Cross-sectional	2	Syphilis	Low
Schinka 2017	Predictors of Mortality in Older Homeless Veterans	USA	2000-2011	Cohort study	1	All-cause mortality	Low
Schladweiler 2009	Factors associated with substance use problem among Maryland Medicaid enrollees affected by serious mental illness	USA	1993-2000	Cohort study	1	Alcohol problem	Low
Schmidt 2011	The impact of substance use disorders on the course of schizophrenia--a 15-year follow-up study: dual diagnosis over 15 years	Denmark	1993-2008	Cohort study	1	All-cause mortality	Low

Author & year	Title	Country	Study years	Design	Number of data points included	Outcomes	Risk of bias
Seal 2001	Predictors and prevention of nonfatal overdose among street-recruited injection heroin users in the San Francisco Bay Area, 1998-1999	USA	1998-1999	Cohort study	3	Non-fatal overdose	High
Sethi 2006	HIV, sexually transmitted infections, and risk behaviours in male sex workers in London over a 10 year period	UK	1994-2003	Cross-sectional analysis of cohort study	1	HIV	High
Shah 2000	Correlates of enrollment in methadone maintenance treatment programs differ by HIV-serostatus	USA	1994-1998	Cross-sectional analysis of cohort study	1	HIV	High
Shannon 2007	Sexual and drug-related vulnerabilities for HIV infection among women engaged in survival sex work in Vancouver, Canada	Canada	2004	Cross-sectional	6	HIV	High
Shannon 2010	Survival sex work involvement as a primary risk factor for hepatitis C virus acquisition in drug-using youths in a canadian setting	Canada	1996-2007	Cohort study	2	HCV	Low
Sherman 2007	Prevalence and correlates of opiate overdose among young injection drug users in a large US city	USA	1999-2002	Cross-sectional analysis of cohort study	1	Non-fatal overdose	Low
Sherriff 2003	A survey of hepatitis C prevalence amongst the homeless community of Oxford	UK	2002	Cross-sectional	2	HCV	Low
Shewan 2000	Fatal drug overdose after liberation from prison: A retrospective study of female ex-prisoners from Strathclyde region (Scotland)	UK	1993-1995	Cohort study	5	HIV, HBV, HCV	2 data points scored as low; 3 scored as high
Shoptaw 2003	Psychiatric and substance dependence comorbidities, sexually transmitted diseases, and risk behaviors among methamphetamine-dependent gay and bisexual men seeking outpatient drug abuse treatment	USA	1997-2000	Cross-sectional	5	Chlamydia, genital gonorrhoea, oral gonorrhoea, HIV, syphilis	High
Shoval 2006	Substance use, suicidality, and adolescent-onset schizophrenia: an Israeli 10-year retrospective study	Israel	1994-2004	Cross-sectional	6	Suicide attempt	Low
Shoval 2007	A 10-year retrospective study of inpatient adolescents with schizophrenia/schizoaffective disorder and substance use	Israel	1994-2004	Cross-sectional	5	Anxiety disorder, depression, mood disorder, self-injurious	High

Author & year	Title	Country	Study years	Design	Number of data points included	Outcomes	Risk of bias
						behaviour, suicidal ideation	
Shuter 1998	Rates of and risk factors for Trichmoniasis among pregntnant inmates in New York City	USA	1996-1997	Cross-sectional	3	Trichomoniasis	Low
Simon 2007	Risk of suicide attempt and suicide death in patients treated for bipolar disorder	USA	1994-2001	Cohort study	3	Suicide attempt	Low
Smereck 1998	Prevalence of HIV infection and HIV risk behaviors associated with living place: on-the-street homeless drug users as a special target population for public health intervention	USA	1992-1994	Cross-sectional	18	HIV	Low
Smyth 2003	Retrospective cohort study examining incidence of HIV and hepatitis C infection among injecting drug users in Dublin	Ireland	1992-1998	Cohort study	2	HCV	High
Sorensen 2005	Drug-use pattern, comorbid psychosis and mortality in people with a history of opioid addiction	Denmark	1984-1999	Cohort study	1	All-cause mortality	Low
Spittal 2012	The Cedar Project: high incidence of HCV infections in a longitudinal study of young Aboriginal people who use drugs in two Canadian cities	Canada	2003-2009	Cohort study	3	HCV	Low
Spittal 2017	Modifiable risk factors for external cause mortality after release from prison: a nested case-control study	Australia	1994-2007	Case-control study	5	Mortality from drug overdose, suicide, transport accidents, external causes	Low
Stephens 2016	Correlates of African American female adolescent offenders 3, 4-Methylenedioxymethamphetamine (MDMA or 'Ecstasy') use and sexually transmitted infection morbidity	USA	not stated	Cross-sectional	5	Chlamydia, genital warts, gonorrhoea, herpes, trichomoniasis	High
Stoove 2008	Mortality among injecting drug users in Melbourne: a 16-year follow-up of the Victorian Injecting Cohort Study (VICS)	Australia	1990-2006	Cohort study	2	All-cause mortality	Low
Strehlow 2012	Hepatitis C among clients of healthcare for the homeless primary care clinics	USA	not stated	Cross-sectional	11	HCV	Low
Surratt 2007	Sex work in the Caribbean Basin: patterns of substance use and HIV risk among migrant sex workers in the US Virgin Islands	USA	2004	Cross-sectional	1	Sexually transmitted infections	High

Author & year	Title	Country	Study years	Design	Number of data points included	Outcomes	Risk of bias
Sutton 2012	Estimating the variability in the risk of infection for hepatitis C in the Glasgow injecting drug user population	UK	1990-2007	Repeated cross-sectional studies	1	HCV	Low
Swofford 2000	Double jeopardy: schizophrenia and substance use	USA	not stated	Cross-sectional	1	Tardive dyskinesia	High
Sylvia 2015	Medical burden in bipolar disorder: findings from the Clinical and Health Outcomes Initiative in Comparative Effectiveness for Bipolar Disorder study (Bipolar CHOICE)	USA	not stated	Cross-sectional analysis of interventional study	1	Cardiometabolic disorder	High
Sylvia 2017	Poor quality of life and functioning in bipolar disorder	USA	not stated	Cross-sectional analysis of interventional study	1	Quality of life	High
Sypsa 2017	Rapid Decline in HIV Incidence Among Persons Who Inject Drugs During a Fast-Track Combination Prevention Program After an HIV Outbreak in Athens	Greece	2012-2013	Cohort study	3	HIV	High
Taylor 2008	Prevalence and determinants of hepatitis C virus infection among female drug injecting sex workers in Glasgow	UK	1999	Cross-sectional	2	HCV	High
Taylor 2013	Low incidence of hepatitis C virus among prisoners in Scotland	UK	2010-2011	Cross-sectional	1	HCV	Low
Thompson 2012	Psychiatric disorders and treatment among newly homeless young adults with histories of foster care	USA	2007-2008	Cross-sectional	1	Any psychiatric disorder	High
Toftdahl 2016	Prevalence of substance use disorders in psychiatric patients: a nationwide Danish population-based study	Denmark	1969-2013	Cohort study	4	Mortality from "unnatural" causes - accidents, suicides, homicides	Low
Topolovec-Vranic 2017	The high burden of traumatic brain injury and comorbidities amongst homeless adults with mental illness	Canada	2009-2011	Cross-sectional analysis of interventional study	1	Traumatic brain injury	High
Torchalla 2011	Substance use and predictors of substance dependence in homeless women	Canada	2009	Cross-sectional	1	Suicide attempt	Low
Torchalla 2014	Posttraumatic stress disorder and substance use disorder comorbidity in homeless adults: Prevalence, correlates, and sex differences	Canada	2009	Cross-sectional	1	PTSD	Low

Author & year	Title	Country	Study years	Design	Number of data points included	Outcomes	Risk of bias
Torres 2015	Bipolar disorder with comorbid attention-deficit and hyperactivity disorder. Main clinical features and clues for an accurate diagnosis	Spain	2008-2013	Cross-sectional	3	ADHD	High
Treso 2012	Prevalence and correlates of HCV, HVB, and HIV infection among prison inmates and staff, Hungary	Hungary	2007-2009	Cross-sectional	2	HBV, HCV	High
Truszkowska 2015	Case-control study of risks and causes of death amongst opioid dependent patients on methadone maintenance treatment	Canada	2005-2012	Case-control study	2	All-cause mortality	1 data point scored as low risk; 1 data point scored as high
Tsui 2014	Association of opioid agonist therapy with lower incidence of hepatitis C virus infection in young adult injection drug users	USA	2000-2012	Cohort study	2	HCV	Low
Turkington 2009	Impact of persistent substance misuse on 1-year outcome in first-episode psychosis	UK	2003-2004	Cross-sectional analysis of cohort study	1	Depression	Low
Tyler 2007	Sexual health of homeless youth: prevalence and correlates of sexually transmissible infections	USA	not stated	Cross-sectional	2	Sexually transmitted infections	Low
Tyndall 2006	HIV seroprevalence among participants at a Supervised Injection Facility in Vancouver, Canada: Implications for prevention, care and treatment	Canada	2003-2005	Cross-sectional analysis of cohort study	2	HIV	Low
Upshur 2017	Prevalence and predictors of substance use disorders among homeless women seeking primary care: An 11 site survey	USA	2015	Cross-sectional	1	Alcohol use disorder	Low
Urbanoski 2007	Substance abuse and quality of life among severely mentally ill consumers: a longitudinal modelling analysis	Canada	1998-2004	Cross-sectional analysis of interventional study	1	Quality of life	High
Uuskula 2008	A study on HIV and hepatitis C virus among commercial sex workers in Tallinn	Estonia	2005-2006	Cross-sectional	2	HIV	High
Van Dooren 2014	A comparison of risk factors for hepatitis C among young and older adult prisoners	Australia	not stated	Cross-sectional analysis of interventional study	2	HCV	High

Author & year	Title	Country	Study years	Design	Number of data points included	Outcomes	Risk of bias
Van Ness 2004	Socioeconomic marginality and health services utilization among Central Harlem substance users	USA	1998-1999	Cross-sectional	3	HIV	Low
van Veen 2010	HIV and sexual risk behavior among commercial sex workers in the Netherlands	Netherlands	2002-2005	Cross-sectional	1	HIV	Low
VanBeek 1998	Infection with HIV and hepatitis C virus among injecting drug users in a prevention setting: retrospective cohort study	Australia	1992-1995	Cohort study	1	HCV	High
VanBeek 2004	The Sydney Medically Supervised Injecting Centre: reducing harm associated with heroin overdose	Australia	2001-2002	Cross-sectional	3	Heroin overdose	Low
vanderPlas 2012	Perceptions of quality of life and disability in homeless persons with schizophrenia and persons with schizophrenia living in non-institutional housing	Netherlands	2000-2001	Cross-sectional	5	Quality of life	High
Verdolini 2017	The aggressor at the mirror: Psychiatric correlates of deliberate self-harm in prison inmates	Italy	2010-2011	Cross-sectional	9	Deliberate self-harm	Low
Verdoux 2001	Predictors and outcome characteristics associated with suicidal behaviour in early psychosis: a two-year follow-up of first-admitted steps	France	not stated	Cohort study	1	Self-harm or suicide	Low
Verneuil 2009	Prevalence and risk factors of the whole spectrum of sexually transmitted diseases in male incoming prisoners in France	France	2000-2003	Cross-sectional	2	HCV, any sexually transmitted disease or blood-borne virus	High
Viitanen 2011	Hepatitis A, B, C and HIV infections among Finnish female prisoners--young females a risk group	Finland	2006	Cross-sectional	2	HCV	High
Vik 2007	Methamphetamine use by incarcerated women: comorbid mood and anxiety problems	USA	not stated	Cross-sectional	10	Depression, dysthymia, generalised anxiety disorder, PTSD, panic disorder	High
Vincenti 2010	Characteristics and clinical changes during hospitalization in bipolar and psychotic disorder patients with versus without substance-use disorders	USA	2004-2009	Repeated cross-sectional studies	8	Anxiety disorder, generalised anxiety disorder, obsessive compulsive disorder, post-traumatic stress disorder, panic disorder, personality	Low

Author & year	Title	Country	Study years	Design	Number of data points included	Outcomes	Risk of bias
						disorder, social phobia, suicide attempts	
Vioque 1998	Prospective cohort study of female sex workers and the risk of HIV infection in Alicante, Spain (1986-96)	Spain	1986-1996	Cohort study	1	HIV	High
Vreugdenhil 2003	Substance use, substance use disorders, and comorbidity patterns in a representative sample of incarcerated male Dutch adolescents	Netherlands	1998-1999	Cross-sectional	2	Anxiety and affective disorders, disruptive behaviour disorders	Low
Ward 2000	Health issues associated with increasing use of "crack" cocaine among female sex workers in London	UK	1989-1996	Repeated cross-sectional studies	3	HBV, HCV	High
Warf 2013	Coming of age on the streets: survival sex among homeless young women in Hollywood	USA	2006-2008	Cross-sectional	2	Sexually transmitted infections, suicide attempt	High
Webb 2014	Suicide, hospital-presenting suicide attempts, and criminality in bipolar disorder: examination of risk for multiple adverse outcomes	Sweden	1973-2009	Cohort study	2	Suicide or attempted suicide	Low
Weild 2000	Prevalence of HIV, hepatitis B, and hepatitis C antibodies in prisoners in England and Wales: a national survey	UK	1997-1998	Cross-sectional	6	HBV, HCV, HIV	Low
Weinstock 2016	Demographic and clinical characteristics associated with comorbid cannabis use disorders (CUDs) in hospitalised patients with bipolar disorder	USA	2010	Cross-sectional	1	Cannabis use disorder	Low
Weiss 2005	Does recovery from substance use disorder matter in patients with bipolar disorder?	USA	1999-2001	Cross-sectional	10	ADHD, anxiety disorder, eating disorder, quality of life, suicide attempt	High
Wenger 2014	Assessment of hepatitis C risk factors and infection prevalence in a jail population	USA	2012-2013	Cross-sectional	3	HCV	Low
Whittaker 2015	Multiply disadvantaged: Health and service utilisation factors faced by homeless injecting drug consumers in Australia	Australia	2012	Cross-sectional	4	Mental HRQoL, mental health problem, non-fatal overdose, physical HRQoL	High

Author & year	Title	Country	Study years	Design	Number of data points included	Outcomes	Risk of bias
Willers 2008	Prevalence and predictors of sexually transmitted infection among newly incarcerated females	USA	2003-2003	Cross-sectional	3	Sexually transmitted infection	2 data points scored as low; 1 scored as high
Williams 2014	Pain behind bars: the epidemiology of pain in older jail inmates in a county jail	USA	2012	Cross-sectional	3	Severe frequent pain	Low
Winter 2013	Hepatitis B virus exposure and vaccination in a cohort of people who inject drugs: what has been the impact of targeted free vaccination?	Australia	2005-2008	Cohort study	2	HBV	1 data point scored as low risk; 1 data point scored as high
Winter 2015	Incidence and predictors of non-fatal drug overdose after release from prison among people who inject drugs in Queensland, Australia	Australia	2008-2010	Cross-sectional analysis of interventional study	1	Non-fatal overdose	High
Wolff 2011	Health problems among detainees in Switzerland: a study using the ICPC-2 classification	Switzerland	2007	Cross-sectional	1	HCV	Low
Wood 2005	Prevalence and correlates of hepatitis C infection among users of North America's first medically supervised safer injection facility	Canada	2003-2004	Cross-sectional analysis of cohort study	2	HCV	1 data point scored as low; 1 data point scored as high
Wright 2006	Psychiatric morbidity among women prisoners newly committed and amongst remanded and sentenced women in the Irish prison system	Ireland	2002	Cross-sectional	1	Personality disorder	Low
Yen 2012	HIV infection risk among injection drug users in a methadone maintenance treatment program, Taipei, Taiwan 20072010	Taiwan	2007-2010	Cross-sectional	1	HIV	High
Yen 2014	Prevalence and factors associated with HIV infection among injection drug users at methadone clinics in Taipei, Taiwan	Taiwan	2012-2013	Cross-sectional	1	HIV	Low
Yen 2015	Factors associated with health-related quality of life among injection drug users at methadone clinics in Taipei, Taiwan	Taiwan	2012-2013	Cross-sectional	4	Physical HRQoL, qualtiy of life	High

Author & year	Title	Country	Study years	Design	Number of data points included	Outcomes	Risk of bias
Yim 2015	Prevalence of mental illness among homeless people in Hong Kong	Hong Kong	2011-2012	Cross-sectional	1	Suicide attempt	High
Young 2018	Dual diagnosis of mental illness and substance use disorder and injury in adults recently released from prison: a prospective cohort study	Australia	2008-2010	Cross-sectional analysis of interventional study	1	Physical HRQoL	Low
Zabransky 2006	Hepatitis C virus infection among injecting drug users in the Czech Republic -- prevalence and associated factors	Czech Republic	2002-2003	Cross-sectional	2	HCV	High
Zavitsanou 2010	Seroepidemiology of human herpesvirus 8 (HHV-8) infection in injecting drug users	Greece	not stated	Cross-sectional	1	HHV-8	High

Section 2.2. Details of studies eligible according to review inclusion criteria but excluded due to overlapping data points

Author & year	Title	Country	Design	Study years	Name of larger study of which this was a part
Blouin 2016	Sex work as an emerging risk factor for Human Immunodeficiency Virus Seroconversion Among People Who Inject Drugs in the SurvIDU network	Canada	Cohort study	2004-2014	SurvIDU
Hankins 2002	Continuing HIV transmission among injection drug users in Eastern Central Canada: The SurvUDI study, 1995 to 2000	Canada	Cohort study	1995-2001	SurvIDU
Hatzakis 2015	Design and baseline findings of a large-scale rapid response to an HIV outbreak in people who inject drugs in Athens, Greece: the ARISTOTLE programme	Greece	Cross-sectional study	2012-2013	ARISTOTLE
Kemp 2006	Homelessness among problem drug users: prevalence, risk factors and trigger events	United Kingdom	Repeated cross-sectional studies	2001-2003	DORIS
Kim 2009	Unstable housing and hepatitis C incidence among injection drug users in a Canadian setting	Canada	Cohort study	1996-2007	VIDUS and SEOSI
Lagerberg 2010	Excessive substance use in bipolar disorder is associated with impaired functioning rather than clinical characteristics, a descriptive study	Norway	Cross-sectional	2003-2007	TOP study - Thematically Organised Psychosis Research
Miller 2002	Females experiencing sexual and drug vulnerabilities are at elevated risk for HIV infection among youth who use injection drugs	Canada	Cross-sectional analysis of cohort study	1996-2000	VIDUS
Miller 2006	Elevated rates of HIV infection among young Aboriginal injection drug users in a Canadian setting	Canada	Cohort study	1996-2003	VIDUS
Puri 2014	Gender influences on hepatitis C incidence among street youth in a Canadian setting	Canada	Cohort study	2005-2011	ARYS
Schechter 1999	Do needle exchange programmes increase the spread of HIV among injection drug users?: an investigation of the Vancouver outbreak	Canada	Cohort study	1996-1998	VIDUS
Simon 2004	Anxiety disorder comorbidity in bipolar disorder patients: data from the first 500 participants in the Systematic Treatment Enhancement Program for Bipolar Disorder (STEP-BD)	United States	Cross-sectional analysis of cohort study	Not stated	STEP-BD
Spittal 2006	Drastic elevations in mortality among female injection drug users in a Canadian setting	Canada	Cohort study	1996-2002	VIDUS
Swartz 2006	Substance use and psychosocial functioning in schizophrenia among new enrollees in the NIMH CATIE study	United States	Cross-sectional analysis of interventional study	2001-2004	CATIE - Clinical Antipsychotic Trials of Intervention Effectiveness
Taylor 2000	Prevalence of hepatitis C virus infection among injecting drug users in Glasgow 1990-1996: are current harm reduction strategies working?	United Kingdom	Repeated cross-sectional studies	1990-1996	Not named - precursor to NESI study
Tyndall 2002	Risky sexual behaviours among injection drugs users with high HIV prevalence: Implications for STD control	Canada	Cohort study	1996-2000	VIDUS - Vancouver Injection Drug Users Study

Author & year	Title	Country	Design	Study years	Name of larger study of which this was a part
Tyndall 2003	Intensive injection cocaine use as the primary risk factor in the Vancouver HIV-1 epidemic	Canada	Cohort study	1996-2000	VIDUS
Ward 1999	Risky business: health and safety in the sex industry over a 9 year period	United Kingdom	Cohort study	1985-1994	Praed Street Project cohort study
Wood 2007	Sex trade involvement is associated with elevated HIV incidence among injection drug users in Vancouver	Canada	Cohort study	1996-2004	VIDUS
Verdoux 1999	Suicidality and substance misuse in first-admitted subjects with psychotic disorder	France	Cohort study	1996-1997	Not named
